# Ethics of randomized trials in a public health emergency

**DOI:** 10.1371/journal.pntd.0006313

**Published:** 2018-05-17

**Authors:** Alex John London, Olayemi O. Omotade, Michelle M. Mello, Gerald T. Keusch

**Affiliations:** 1 Department of Philosophy, Carnegie Mellon University, Pittsburgh, Pennsylvania, United States of America; 2 Institute of Child Health, University of Ibadan, Ibadan, Nigeria; 3 School of Law and School of Medicine, Stanford University, Stanford, California, United States of America; 4 National Emerging Infectious Diseases Laboratory, Boston University, Boston, Massachusetts, United States of America; George Washington University School of Medicine and Health Sciences, UNITED STATES

The 2014–2015 outbreak of Ebola in West Africa claimed over 11,000 lives and laid bare the challenges of responding to a large-scale, swiftly evolving public health emergency. Prominent among these difficulties was disagreement about the ethics of conducting clinical research during epidemics and whether clinical trials of vaccines and therapeutics should employ randomization and concurrent controls. To facilitate rapid, well-coordinated responses to future public health emergencies, the United States National Academies of Sciences, Engineering, and Medicine established a committee to assess the clinical trials conducted in Guinea, Sierra Leone, and Liberia during the outbreak. The key findings and conclusions regarding ethical issues raised about conducting research during public health crises are briefly described here and are fully evaluated in the Committee’s report [1].

First, the Committee concluded that “research is an essential component in epidemic response, as it is the only way to learn how to improve care for current and future patients and to potentially prevent an epidemic from occurring again” [1]. For diseases like Ebola, outbreaks provide the only setting in which clinical trials can be conducted to determine efficacy and safety of investigational products for treating or preventing infection, because results from animal models cannot be reliably extrapolated to humans, [2] and human challenge studies are not possible. Although some clinicians perceived conflict between their clinical obligations and the mission of research, conducting clinical trials during outbreaks is indispensable to determining which interventions actually advance the humanitarian mission of minimizing mortality and morbidity.

Second, to learn how to improve care, research must be designed to generate evidence that can support reliable inferences about safety and efficacy. The Committee concluded that “randomized controlled trials (RCTs) are the most reliable way to identify the relative benefits and risks of investigational products, and, except [in] rare circumstances… every effort should be made to implement them during epidemics” [1].

Third, the committee rejected the claim made by some stakeholders that due to Ebola’s high mortality rate, equipoise would not exist for studies of therapeutic interventions that included the possibility of randomization to a standard-of-care control arm [3]. In part, such claims reflect the mistaken idea that equipoise refers to a state of uncertainty in the mind of the individual researcher, in which each intervention is equally likely to be superior to the others. Thirty years ago, Freedman rejected this view because it would prohibit studies in situations in which researchers have a hunch that one intervention is superior to the others but in which the information on which that hunch is based is not sufficient to persuade other reasonable experts [4]. Similarly, it would prohibit research in cases where fully informed experts have conflicting judgments about which intervention is likely to prove superior for a particular indication [5]. The appropriate standard, known as clinical equipoise, holds that randomization is permissible when a state of conflict or uncertainty exists in the expert medical community about the relative clinical merits of a set of health interventions. Even if some expert clinicians have a preference for investigational interventions over the standard of care, clinical equipoise persists as long as other fully informed and expert clinicians would continue to treat patients with the standard of care [6].

In addition, the claim that randomized designs of interventions during the 2014 Ebola outbreak would have entailed the unethical withholding of potentially beneficial interventions from people in desperate need also rested on the unwarranted assumption that interventions in the early stages of development were more likely to be highly efficacious than to worsen participants’ already fragile condition. Available preclinical data did not support such enthusiasm. Even if it had, 90% of novel interventions fail to prove effective for any indication [7], a statistic that does not reflect the fact that even drugs eventually approved for some indication are often tested in a range of indications for which they are ineffective or even harmful. Absent this unwarranted presumption, randomization is ethically permissible.

Fourth, effectively communicating reliable scientific information to local communities—including uncertainty about the efficacy and safety of investigational interventions, a cornerstone of respectful community engagement—is an essential component of ethically responsible research. Public health emergencies are contexts of heightened uncertainty and mistrust. Public resistance to randomized trials in some Ebola-affected regions was fueled by a misconception that the interventions were highly efficacious “secret serums” [8]. This arose partly because of the higher survival rate for expatriates receiving investigational interventions who were also evacuated to their home countries, where they also received the highest level of supportive care in addition to any other treatment modality, compared to patients in West Africa [9]. In the face of initial opposition to randomized studies, some researchers and humanitarian organizations quickly concluded local communities would never accept such trials. But in fact, with effective community engagement and information sharing, one randomized therapeutic trial and three randomized vaccine studies were conducted in the waning stages of the outbreak in West Africa. The lesson—that informed communities that are engaged appropriately may indeed be willing to accept randomized studies—is crucial for future outbreaks.

This engagement, however, cannot be initiated late in the game. The Committee’s report includes several recommendations for increasing planning and preparedness during interepidemic periods so that reliable, ethically acceptable research can be organized, reviewed, and launched expeditiously when the next outbreak strikes.

Finally, to frontline caregivers facing overwhelming clinical need and acute shortages of supplies and manpower in the early stages of the outbreak, research felt like an unjustifiable diversion of scarce resources. The question of whether rigorous clinical trials of novel therapeutics and vaccines should or can be implemented during epidemic emerging infectious diseases has been affirmatively answered during the West Africa Ebola outbreak. The National Academies of Sciences, Engineering, and Medicine report emphasizes that sustained, coordinated international support for health systems in low- and middle-income countries is now of paramount importance. This includes investing in their medical infrastructure, enhancing their capacity to conduct public health surveillance and research, and ensuring that collaborations provide lasting benefits to affected communities.

## Supporting information

S1 File (PDF)
