# Supplementary material for: Ethics of randomized trials in a public health emergency
Source: PLoS Negl Trop Dis. 2018 May 17;12(5):e0006313. doi: 10.1371/journal.pntd.0006313 (PMC5957337; doi:10.1371/journal.pntd.0006313)
Supplement: S1 File — (PDF) [file pntd.0006313.s001.pdf]

The National Academies of SCIENCES ENGINEERING MEDICINE

THE NATIONAL ACADEMIES PRESS

This PDF is available at http://www.nap.edu/24739

SHARE

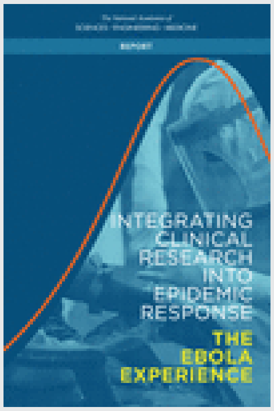

Integrating Clinical Research into Epidemic Response: The Ebola Experience

GET THIS BOOK

FIND RELATED TITLES

DETAILS

342 pages | 6 x 9 | PAPERBACK
ISBN 978-0-309-45776-7 | DOI: 10.17226/24739

CONTRIBUTORS

Gerald Keusch, Keith McAdam, Patricia A. Cuff, Michelle Mancher, and Emily R. Busta, Editors; Committee on Clinical Trials During the 2014-2015 Ebola Outbreak; Board on Global Health; Board on Health Sciences Policy; Health and Medicine Division; National Academies of Sciences, Engineering, and Medicine

Visit the National Academies Press at NAP.edu and login or register to get:

- Access to free PDF downloads of thousands of scientific reports
- 10% off the price of print titles
- Email or social media notifications of new titles related to your interests
- Special offers and discounts

Distribution, posting, or copying of this PDF is strictly prohibited without written permission of the National Academies Press. (Request Permission) Unless otherwise indicated, all materials in this PDF are copyrighted by the National Academy of Sciences.

Copyright © National Academy of Sciences. All rights reserved.

INTEGRATING CLINICAL RESEARCH INTO EPIDEMIC RESPONSE

THE EBOLA EXPERIENCE

Gerald Keusch, Keith McAdam, Patricia A. Cuff,
Michelle Mancher, and Emily R. Busta, *Editors*

Committee on Clinical Trials During the 2014–2015 Ebola Outbreak

Board on Global Health

Board on Health Sciences Policy

Health and Medicine Division

A Report of

The National Academies of

SCIENCES · ENGINEERING · MEDICINE

THE NATIONAL ACADEMIES PRESS
Washington, DC
www.nap.edu

Copyright © National Academy of Sciences. All rights reserved.

THE NATIONAL ACADEMIES PRESS 500 Fifth Street, NW Washington, DC 20001

This activity was supported by Contract No. HHSP233201400020B/HHSP23337042 with the Assistant Secretary for Preparedness and Response, U.S. Department of Health and Human Services. Any opinions, findings, conclusions, or recommendations expressed in this publication do not necessarily reflect the views of any organization or agency that provided support for the project.

International Standard Book Number-13: 978-0-309-45776-7
International Standard Book Number-10: 0-309-45776-9
Digital Object Identifier: https://doi.org/10.17226/24739
Library of Congress Control Number: 2017941808

Additional copies of this publication are available for sale from the National Academies Press, 500 Fifth Street, NW, Keck 360, Washington, DC 20001; (800) 624-6242 or (202) 334-3313; http://www.nap.edu.

Copyright 2017 by the National Academy of Sciences. All rights reserved.

Printed in the United States of America

Cover credit: Photo by Rebecca E. Rollins/Partners in Health.

Suggested citation: National Academies of Sciences, Engineering, and Medicine. 2017. *Integrating clinical research into epidemic response: The Ebola experience*. Washington, DC: The National Academies Press. doi: https://doi.org/10.17226/24739.

Copyright © National Academy of Sciences. All rights reserved.

The National Academies of

SCIENCES • ENGINEERING • MEDICINE

The **National Academy of Sciences** was established in 1863 by an Act of Congress, signed by President Lincoln, as a private, nongovernmental institution to advise the nation on issues related to science and technology. Members are elected by their peers for outstanding contributions to research. Dr. Ralph J. Cicerone is president.

The **National Academy of Engineering** was established in 1964 under the charter of the National Academy of Sciences to bring the practices of engineering to advising the nation. Members are elected by their peers for extraordinary contributions to engineering. Dr. C. D. Mote, Jr., is president.

The **National Academy of Medicine** (formerly the Institute of Medicine) was established in 1970 under the charter of the National Academy of Sciences to advise the nation on medical and health issues. Members are elected by their peers for distinguished contributions to medicine and health. Dr. Victor J. Dzau is president.

The three Academies work together as the **National Academies of Sciences, Engineering, and Medicine** to provide independent, objective analysis and advice to the nation and conduct other activities to solve complex problems and inform public policy decisions. The National Academies also encourage education and research, recognize outstanding contributions to knowledge, and increase public understanding in matters of science, engineering, and medicine.

Learn more about the National Academies of Sciences, Engineering, and Medicine at **www.national-academies.org**.

Copyright © National Academy of Sciences. All rights reserved.

The National Academies of
SCIENCES • ENGINEERING • MEDICINE

Reports document the evidence-based consensus of an authoring committee of experts. Reports typically include findings, conclusions, and recommendations based on information gathered by the committee and committee deliberations. Reports are peer reviewed and are approved by the National Academies of Sciences, Engineering, and Medicine.

Proceedings chronicle the presentations and discussions at a workshop, symposium, or other convening event. The statements and opinions contained in proceedings are those of the participants and have not been endorsed by other participants, the planning committee, or the National Academies of Sciences, Engineering, and Medicine.

For information about other products and activities of the National Academies, please visit nationalacademies.org/whatwedo.

Copyright © National Academy of Sciences. All rights reserved.

COMMITTEE ON CLINICAL TRIALS DURING THE 2014–2015 EBOLA OUTBREAK

GERALD T. KEUSCH (*Co-Chair*), Professor of Medicine and Global Health, Boston University Schools of Medicine and Public Health
KEITH McADAM (*Co-Chair*), Emeritus Professor of Clinical and Tropical Medicine, London School of Hygiene & Tropical Medicine
ABDEL BABIKER, Professor of Epidemiology and Medical Statistics, Medical Research Council Clinical Trials Unit at University College London
MOHAMED BAILOR BARRIE, Co-Founder, Chief Strategic Officer, The Wellbody Alliance
JANICE COOPER, Country Representative, Liberia Mental Health Initiative, The Carter Center
SHEILA DAVIS, Chief Nursing Officer, Partners In Health
KATHRYN EDWARDS, Professor of Pediatrics, Vanderbilt University School of Medicine, Vanderbilt Research Program
SUSAN ELLENBERG, Professor of Biostatistics, Perelman School of Medicine, University of Pennsylvania
ROGER LEWIS, Professor and Chair of the Department of Emergency Medicine, Harbor–University of California, Los Angeles, Medical Center
ALEX JOHN LONDON, Professor of Philosophy; Director, Center for Ethics and Policy, Carnegie Mellon University
JENS LUNDGREN, Professor of Infectious Diseases, University of Copenhagen
MICHELLE M. MELLO, Professor of Law, Stanford University School of Medicine, School of Law
OLAYEMI OMOTADE, Professor of Pediatrics and Child Health, Institute of Child Health, University College Hospital, University of Ibadan
DAVID H. PETERS, Professor, Johns Hopkins Bloomberg School of Public Health
FRED WABWIRE-MANGEN, Associate Professor of Epidemiology and Public Health, Makerere University School of Public Health
CHARLES D. WELLS, Head of Development and Associate Vice President, Infectious Diseases Unit, Sanofi-U.S.

Study Staff

PATRICIA A. CUFF, Study Co-Director
MICHELLE MANCHER, Study Co-Director

Copyright © National Academy of Sciences. All rights reserved.

EMILY R. BUSTA, Associate Program Officer
MICHAEL J. BERRIOS, Senior Program Assistant
OLIVIA YOST, Research Assistant
ELLEN K. KIMMEL, Senior Research Librarian
ANNE B. CLAIBORNE, Senior Program Officer
JULIE PAVLIN, Director, Board on Global Health
ANDREW M. POPE, Director, Board on Health Sciences Policy

Consultants

JANET DARBYSHIRE, Emeritus Professor of Epidemiology, University College London
ERIN HAMMERS FORSTAG, Science Writer

Copyright © National Academy of Sciences. All rights reserved.

Reviewers

This report has been reviewed in draft form by individuals chosen for their diverse perspectives and technical expertise. The purpose of this independent review is to provide candid and critical comments that will assist the institution in making its published report as sound as possible and to ensure that the report meets institutional standards for objectivity, evidence, and responsiveness to the study charge. The review comments and draft manuscript remain confidential to protect the integrity of the deliberative process. We wish to thank the following individuals for their review of this report:

Dennis Carroll, U.S. Agency for International Development
Geneviève Chêne, Université de Bordeaux
I. Glenn Cohen, Harvard Law School
Patricia Fast, International AIDS Vaccine Initiative
Thomas R. Fleming, University of Washington
Peter Barton Hutt, Covington & Burling LLP
Mohammad Bailor Jalloh, FOCUS 1000
Elaine L. Larson, Columbia University
Heidi J. Larson, London School of Hygiene & Tropical Medicine
Adam C. Levine, Brown University
Susan A. Murphy, University of Michigan
John-Arne Røttingen, Harvard T.H. Chan School of Public Health
Janet Wittes, Statistics Collaborative, Inc.

Copyright © National Academy of Sciences. All rights reserved.

Although the reviewers listed above provided many constructive comments and suggestions, they were not asked to endorse the report's conclusions or recommendations, nor did they see the final draft of the report before its release. The review of this report was overseen by **Georges Benjamin,** American Public Health Association, and **Lawrence D. Brown,** University of Pennsylvania. They were responsible for making certain that an independent examination of the report was carried out in accordance with the institutional procedures and that all review comments were carefully considered. Responsibility for the final content of this report rests entirely with the authoring committee and the institution.

Copyright © National Academy of Sciences. All rights reserved.

Preface

In the beginning there was one—a 2-year-old infant from a small village near the town of Guéckédou in the Forest Region of Guinea who, after a brief and dramatic illness, died on December 28, 2013. During January 2014, several immediate family members developed similar symptoms and died too, followed by some of the midwives, traditional healers, and health care workers who attended them. Soon the numbers affected in Guinea were in the tens, then the hundreds, and by the middle of the year in the thousands, now not only in Guinea but also in neighboring Sierra Leone and Liberia. And so the Ebola outbreak of 2014–2015 began and spread, not unnoticed but rather unidentified, and grew to a magnitude never before seen since the virus had first been discovered in 1976. Ultimately the outbreak affected more than 28,600 individuals during 2014 and 2015, with at least 11,310 deaths recorded. This is far more than the total from every outbreak in the 40 years we have known of the Ebola virus.

Clearly the world was not prepared; while some aspects of the response worked, and the outbreak was ultimately brought to a close, many things did not work well at all. In part this was due to the nearly four decades of prior experience with Ebola outbreaks, which were all in relatively remote and isolated communities in central Africa, affecting a few to a few hundred individuals, albeit with a high case fatality rate, before coming to an end as effective public health measures to stop transmission were put into place. With this in mind as the typical pattern, many in the global public health community could not—or would not—believe that 2014 was really different. Health care and public health, like science itself, are built on the cumulative experience of the past, which serves as the basis for our expec-

Copyright © National Academy of Sciences. All rights reserved.

tations and the foundation for new knowledge generation. In this instance, however, expectations gained from knowing the past prevented the experts, or at least most of them, from seeing that something different was really happening; these experts failed the vision test—to match what was known with what was happening and see both similarities and differences. That difference from previous outbreaks lay in the habitual movement of people across the porous borders where Guinea, Liberia, and Sierra Leone abut one another, as the roads and available transportation made it easy for people to travel to the larger towns and the capital cities and on to nearby countries in the region—and, in a few instances, bringing the virus with them. That was all it took, and this ability transformed what had always been a short, limited outbreak in the past, to an epidemic spiraling out of control; this was especially true where the epidemic had originally begun, where it was rapidly becoming a major public health disaster, quickly outpacing the limited health and public health systems and overwhelming any surge capacity there was. Although the global humanitarian organization Médecins Sans Frontières (MSF) became involved early on, its capacity too was quickly overwhelmed by the magnitude of the outbreak. While MSF recognized what was happening, its calls for urgent international action were essentially disregarded, in part because of the reluctance of the affected countries to admit there was a crisis and, in turn, the reluctance of the World Health Organization (WHO) to believe this was really different, making WHO unable to focus more on global public health than on politics and take the necessary action only it could take.

The rest is now history: an outbreak of unprecedented magnitude in a setting of limited capacity, with political systems that were fragile after many years of civil war, plagued by violence, and the virus itself that killed health care workers, further decimating the indigenous capacity to care for patients and limit further dissemination of infection. It was a perfect storm. On top of this, there was a sluggish and contentious international emergency response, especially early on, often carried out with overworked, well-intentioned but relatively untrained international volunteer health workers. When the almost inevitable instances of infection occurred among them, they were repatriated to their countries of origin for state-of-the-art supportive clinical care, often raising the fear levels about the importation of Ebola among the general public in the United States and other developed countries. Some of these individuals received experimental drugs that were being slowly advanced through preclinical research in their countries, and when they survived it fanned the rumors circulating in the affected West African countries of a magic serum that cured the expatriates but that was not being made available to the local African population. This fed into conspiracy theories about the origin of the virus, and when there was the possibility of actually doing clinical research to establish safety and efficacy

Copyright © National Academy of Sciences. All rights reserved.

of drugs and vaccines for Ebola, the conspiracy morphed into the view that now the West was using Africans as guinea pigs to study these experimental products. To make it worse, the various groups capable of mounting and overseeing these clinical trials could not agree on the right study design, the ethics of using controls and randomization, or whose patients would be offered the opportunity to be enrolled. With this background it is not surprising there was community backlash against the health care workers, the proposed studies, the health and political leadership in the countries, and the researchers, both local and international.

The challenge this committee has taken on is to step back and review the clinical research conducted during 2014–2015, specifically in Guinea, Liberia, and Sierra Leone, to better understand what happened, the nature of the constraints that affected the design and implementation of human clinical trials, and what knowledge was actually gained on the safety and efficacy of the tested drugs and vaccines, most of which had never been given to a human before. All of this took place in the setting of an exploding humanitarian disaster where the provision of effective baseline clinical care was beyond the reach of many of the emergency treatment centers that were ultimately set up, at least in the early months of the outbreak, and where both caregivers and researchers would be constrained by the personal protective equipment they had to use and the limited time they could spend at the bedside, given the ambient conditions. And so in February 2016 we were asked by the National Academies of Sciences, Engineering, and Medicine to co-chair a committee that would make recommendations to the three U.S. sponsors, the Office of the Assistant Secretary for Preparedness and Response, the National Institute of Allergy and Infectious Disease, and the U.S. Food and Drug Administration of the U.S. Department of Health and Human Services, to help them to do better the next time an outbreak like this occurs. The specific charge we took on is presented in Chapter 1, and the rest of the report presents our findings, conclusions, and recommendations to the study sponsors and, beyond them, to the greater global community. Our goal was not to cast blame but rather to find ways to improve what should be done the next time.

However, the statement of task was cognizant of the fact that conducting clinical trials requires "collaborative investment to achieve long-term ethical and scientific gains" and "planning activities during the inter-epidemic period." To effectively launch trials in the context with which they were carried out during the Ebola outbreak, multiple issues of a scientific, political, cultural, social, ethical, and economic nature impacted the clinical research agenda and what it could produce; and there were both national and international implications that required consideration. We watched the efforts to build capacity in the three epicenter countries with support from the United States (and many other international donors and

Copyright © National Academy of Sciences. All rights reserved.

scientific institutions) and then saw funds allocated to this effort by the U.S. Congress tapped to meet the new challenge of Zika virus, the outbreak du jour, before the local and global benefits of the investments in Ebola, those undertaken and those planned and still unfolding, could be reaped for the global community, not the least of which for the United States as well. While we do not provide specific recommendations regarding the sources of the necessary investments to build a better global system to address emerging infectious diseases in the future, we do address what we believe to be the critical issues to tackle and some of the actions necessary to do that. But we also know that funding will determine how far the world can improve on the status of things as they were in January 2014, when the Ebola outbreak in West Africa began, and how quickly that improvement might happen. It is clear that preparedness to respond to the next outbreak and preparedness to pursue clinical research on therapeutic products and vaccines during an outbreak are of the highest priority and that they will require sustained and flexible funding sources, free from political whim and pressure, to develop and reach the necessary functionality. We note here new efforts by the World Bank Group to engage with the WHO and its newfound partners, including the Food and Agriculture Organisation, the World Organization for Animal Health, and a number of United Nations agencies. And we welcome the growing engagement of these key players with foundations and charities to consult and collaborate more effectively on emerging infectious diseases, and to identify what needs to be done and how to find the resources from the global community to make that happen today and in the future.

The costs for the U.S. government and its many partners around the world to respond to the epidemic were enormous, as were the costs borne by the three affected countries. The economic effects of the epidemic outbreak will be felt for many years to come in Guinea, Liberia, and Sierra Leone. It seems amazing that despite the 40-year head start that we had for Ebola we were not adequately prepared and that nearly 12,000 deaths later we still do not have licensed therapeutic agents or vaccines. Rather than expecting that the swarm of wealthy, powerful, and knowledgeable experts would rapidly develop and implement effective plans to control the epidemic, we learned that community engagement takes time and skill to reach common ground on what needs to be done, that communication science requires considerable investment, and that strengthening capacity in clinical care, public health, and health research systems is now an urgent and necessary requirement if this sort of epidemic is to be prevented and controlled in the future. The global costs of failure are devastating; the price of effective preparedness is certainly worth the investment. Many highly motivated individuals and institutions can turn this Ebola outbreak into a

Copyright © National Academy of Sciences. All rights reserved.

global good, if they are charged with implementing the international learnings from the experience.

What we do not know is whether the needs, both in terms of capacity strengthening and the requisite financial support, can be met by an often fragmented global system of governance and engagement. We thank the brave people of Guinea, Liberia, and Sierra Leone, who persevered through the ordeal and have emerged more committed than ever to the success of their countries, and all of those who attended the open meetings of the committee and gave us the benefit of their knowledge, experience, and passion to help in every way they could to improve the response to such a calamity in the future—and perhaps to be able to prevent such outbreaks. This is the intent of this report, to move the dial forward to reach such a day. As we look back at the work by a remarkable group of committee members, our consultant Janet Darbyshire, and our project staff at the National Academies of Sciences, Engineering, and Medicine with whom we worked so closely over the past 10 months, we can identify one rather critical feature of a global community we know and believe in: from those who have much, much is expected.

Gerald T. Keusch and Keith McAdam, *Co-Chairs*
Committee on Clinical Trials During the 2014–2015 Ebola Outbreak

Copyright © National Academy of Sciences. All rights reserved.

Copyright © National Academy of Sciences. All rights reserved.

Acknowledgments

This report reflects contributions from a number of individuals and groups. The committee takes this opportunity to recognize those who so generously gave their time and expertise to inform its deliberations. To begin the committee would like to thank the individuals who attended and presented at their open-session meetings (see Appendix A). The committee greatly benefited from the opportunity for discussion with these individuals and is thankful for their many contributions.

The committee could not have done its work without the support of the many clinical trial teams who surmounted innumerable challenges to successfully conduct clinical trials in the midst of an unprecedented outbreak, specifically Ebola ça Suffit (Ana Maria Henao-Restrepo, Initiative for Vaccine Research and World Health Organization; Mandy Kader Konde, University of Conakry, Guinea Ebola Research Commission, and Center of Research on Diseases), STRIVE (Anne Schuchat, Centers for Disease Control and Prevention; Barbara Mahon, Centers for Disease Control and Prevention; Mohamed Samai, College of Medicine and Allied Health Sciences and Ministry of Health and Sanitation, Freetown, Sierra Leone), EBOVAC–Salone (Johan Van Hoof, Janssen Pharmaceuticals R&D; Christopher McShane, Janssen Research & Development, LLC), PREVAIL (Richard T. Davey, National Institutes of Allergy and Infectious Diseases [NIAID], National Institutes of Health [NIH]; John Beigel, Leidos Biomedical Research, Inc., in support of Clinical Research Section, Liberian Institute of Biomedical Research, NIAID, NIH; Mike Proschan, NIAID, NIH; Lori Dodd, NIAID, NIH; Jerome F. Pierson, NIAID, NIH; James Neaton, University of Minnesota; Stephen B. Kennedy, Incident Manage-

Copyright © National Academy of Sciences. All rights reserved.

ment System, Liberia; Barthalomew Wilson, Partnership for Research on Ebola Virus in Liberia), JIKI (France Mentre, Université Paris Diderot, Paris, France; Denis Malvy, University Hospital of Bordeaux; Abdoul Habib Beavogui, National Center for Training and Research in Rural Health "Jean SENECAL" of Maferinyah, Republic of Guinea), RAPIDE–Ebola (Trudie Lang, University of Oxford; John Whitehead, Lancaster University; Peter Horby, University of Oxford and International Severe Acute Respiratory and Emerging Infections Consortium), and Ebola-Tx (Johan van Griensven, Institute of Tropical Medicine–Antwerp).

The committee is grateful to the people of Guinea, Liberia, and Sierra Leone for hosting us and generously sharing their expertise, experiences, and personal stories. In particular, we would like to thank the representatives of the ministries of health Hon. Zulianatu Cooper (Sierra Leone), Hon. Alie Wurie (Sierra Leone), Hon. Yah Zolia (Liberia), and Hon. Tolbert Nyenswah (Liberia). Special thanks to Ambassador Juli Endee (Liberia Crusaders for Peace) and Chief Zanzan Kawa (Council of Chiefs of Liberia) for graciously welcoming us to Liberia and sharing with us the stories of their communities. We also thank Mosoka Fallah (Action Contre la Faim), Vuyu Kanda Golakai (University of Liberia), and Fatorma Bolay (Liberia Institute of Biomedical Research) for their assistance in arranging on-site visits and providing the committee the opportunity to experience Monrovia.

Many others provided support to this project in various ways. We thank Alexander M. Capron, University of Southern California, for his thoughtful review. We are grateful to Dana Vigue for her research assistance and to Elena Ovaitt Weiss for her creative efforts in our graphic design process, and finally, Robert Pool is to be recognized for his editorial assistance in preparing this report.

Copyright © National Academy of Sciences. All rights reserved.

Contents

ACRONYMS AND ABBREVIATIONS **xxiii**

SUMMARY **1**
Study Charge and Approach, 2
Assessment of Ebola Clinical Trials, 3
Ethics of Clinical Research During an Epidemic, 6
Recommendations, 8
Being Prepared: Launching Clinical Trials in an Epidemic, 18

1 INTRODUCTION **19**
Charge to the Committee and Study Scope, 22
Study Methodology, 24
Context of the 2014–2015 Ebola Epidemic, 24
Organization of the Report, 32
References, 33

2 CONDUCTING CLINICAL RESEARCH DURING AN EPIDEMIC **37**
Early Debates About Use of Products, 39
Planning Clinical Trials, 43
Ethical Perspectives, 52
Conclusions, 72
References, 75

Copyright © National Academy of Sciences. All rights reserved.

3 **ASSESSMENT OF THERAPEUTIC TRIALS** **83**
Discussion, 96
References, 108

4 **ASSESSMENT OF VACCINE TRIALS** **113**
Assessment of Trials, 117
Overall Assessment of Vaccines, 139
References, 151

5 **STRENGTHENING CAPACITY FOR RESPONSE AND RESEARCH** **155**
Capacity Challenges to Conducting Clinical Research, 158
Integration of Research into Health Systems, 183
References, 190

6 **ENGAGING COMMUNITIES IN RESEARCH AND RESPONSE** **199**
Engaging Communities in Response, 202
Engaging Communities in Research, 205
The Role of Communication, 214
Summary, 217
References, 219

7 **FACILITATING INTERNATIONAL COORDINATION AND COLLABORATION** **223**
Inter-epidemic Period, 225
During an Epidemic, 236
Embedding Research into Response, 246
Summary, 247
References, 248

APPENDIXES
A Study Approach and Methods 251
B Clinical Trial Designs 287
C Ethical Principles for Research with Human Subjects 297
D Biographical Sketches of Committee Members and Staff 301

Copyright © National Academy of Sciences. All rights reserved.

Boxes, Figures, and Tables

BOXES

1-1 Ebola Virus Disease, 20
1-2 Statement of Task, 22

2-1 Adaptive Trial Design, 48
2-2 Statistical Guidelines for Early Termination of Clinical Trials, 50
2-3 Moral Framework for Research, 54
2-4 Inclusion of Pregnant Women and Children in Clinical Trials, 58
2-5 Special Situations in Which Uncontrolled Phase 3 Trials May Be Warranted, 64
2-6 The Standard of Care Owed to Research Participants, 66

4-1 WHO Requirements of Vaccine Trials, 116

5-1 Council on Health Research for Development (COHRED), 177

6-1 Purposes of Community Engagement During an Epidemic, 201

7-1 Global Health Security Agenda, 243

FIGURES

S-1 A timeline of critical components: Launching clinical trials in an epidemic, 17

Copyright © National Academy of Sciences. All rights reserved.

1-1 Weekly reported Ebola cases during the 2014–2015 Ebola outbreak, 29

3-1 Clinical trial enrollment dates during the 2014–2015 Ebola outbreak—therapeutic trials, 86

4-1 Clinical trial enrollment start dates during the 2014–2015 Ebola outbreak—vaccine trials, 121
4-2 Timeline of PREVAIL Trial, 132

5-1 Timeline to implement a clinical trial, 180

6-1 Organizational structure of the Liberian–U.S. research partnership, 207

7-1 A timeline of critical components: Launching clinical trials in an epidemic, 226
7-2 A visual representation of how the international coalition of stakeholders and rapid research response workgroup relate to one another, as suggested in Recommendations 7a and 7b, 236

TABLES

S-1 Investigational Ebola Therapeutic Agents in Formal Clinical Trials During the 2014–2015 Ebola Outbreak, 4
S-2 Investigational Ebola Vaccines in Formal Clinical Trials During the 2014–2015 Ebola Outbreak, 5

1-1 Data Depicting the Deficit of Medical Doctors and Hospital Beds in the Ebola-Affected Countries at the Time of the Ebola Outbreak in Comparison to Higher-Income Countries, 25

3-1 Timeline of Therapeutic Trials, 85
3-2 Available Data on Investigational Therapeutic Agents for Ebola When Trials Were Being Planned (November 2014), 88
3-3 Summary of Therapeutic Trials Conducted During 2014–2015 Ebola Outbreak, 100

4-1 October 2015 WHO Summary of the Phase 1 Ebola Vaccine Trials, 118
4-2 Timeline of Vaccine Trials, 120
4-3 Calculations of Vaccine Efficacy and Vaccine Effectiveness Based on Different Study Populations—Guinea Ring Vaccine Trial, 123

Copyright © National Academy of Sciences. All rights reserved.

4-4 Effect of Vaccine on Cases of Ebola Virus Disease in Different Study Populations—Guinea Ring Vaccine Trial, 126
4-5 Preliminary Antibody Responses Following Vaccination—PREVAIL I Trial, 135
4-6 Summary of Ebola Vaccine Trials, 142

5-1 Challenges and Solutions of Implementing Sierra Leone Trial to Introduce a Vaccine Against Ebola, 170

6-1 Determining Research Benefits for Study Participants and/or Communities Involved in Research, 210

B-1 Brief Summary of the Advantages and Disadvantages of Various Clinical Trial Designs, 287

C-1 Basic Ethical Principles That Should Govern Research with Human Subjects, 297

Copyright © National Academy of Sciences. All rights reserved.

Copyright © National Academy of Sciences. All rights reserved.

Acronyms and Abbreviations

Ad26	recombinant adenovirus serotype 26
AIDS	acquired immune deficiency syndrome
ASPR	Office of the Assistant Secretary for Preparedness and Response
AZT	azidothymidine, also known as Zidovudine (ZDV)
BBC	British Broadcasting Corporation
CCC	community care center
CDC	U.S. Centers for Disease Control and Prevention
CEPI	Coalition for Epidemic Preparedness Innovations
ChAd3	recombinant chimpanzee adenovirus type 3 vector vaccine
CIOMS	Council for International Organizations of Medical Sciences
CMV	cytomegalovirus
COE	Council of Europe
COHRED	Council on Health Research for Development
CP	convalescent plasma
Ct	cycle threshold (PCR)
DNA	deoxyribonucleic acid
DSMB	data and safety monitoring board

Copyright © National Academy of Sciences. All rights reserved.

EBOVAC	Ebola vaccine projects
EC50	half maxima effective concentration
ECOWAS	Economic Community of West African States
ELISA	enzyme-linked immunosorbent assay
ENHR	essential national health research
ETU	Ebola treatment unit
EVD	Ebola virus disease
FDA	U.S. Food and Drug Administration
GCP	good clinical practice
GDP	gross domestic product
GHSA	Global Health Security Agenda
GP	glycoprotein
GSK	GlaxoSmithKline
H1N1	influenza A virus subtype H1N1
HCW	health care worker
HHS	U.S. Department of Health and Human Services
HIV	human immunodeficiency virus
IC50	half maximal inhibitory concentration
ICS	international coalition of stakeholders
IDA	International Development Association component of the World Bank Group
IDSR	integrated disease surveillance and response
IgG	immunoglobulin-G
IHR	International Health Regulations
IMC	International Medical Corps
Inserm	Institut national de la santé et de la recherche médicale (French National Institute of Health and Medical Research)
IOM	Institute of Medicine
IV	intravenous
JIKI	means “Hope” in “Kissi” language
LIBR	Liberian Institute of Biomedical Research
MERS-CoV	Middle East respiratory syndrome coronavirus
MOU	memorandum of understanding
MSF	Médecins Sans Frontières (Doctors Without Borders)
MVA	modified vaccinia virus

Copyright © National Academy of Sciences. All rights reserved.

NGO	nongovernmental organization
NHP	nonhuman primate
NIAID	National Institute of Allergy and Infectious Diseases, NIH
NIH	U.S. National Institutes of Health
oSOC	optimized standard of care
PCR	polymerase chain reaction
PEF	Pandemic Emergency Financing Facility
PHEIC	public health emergencies of international concern
PK	pharmacokinetics
PO	per os (oral administration)
PPE	personal protective equipment
PREVAIL	Partnership for Research on Ebola Virus in Liberia, NIH
R^{3}W	rapid research response workgroup
R&D	research and development
RAPIDE	Rapid Assessment of Potential Interventions & Drugs for Ebola
RCT	randomized controlled trial
REDISSE	Regional Disease Surveillance Systems Enhancement Program
RHInnO	Rapid Health Innovation
RNA	ribonucleic acid
rVSV	recombinant vesicular stomatitis virus
SAE	serious adverse event
SARS	severe acute respiratory syndrome
SBCC	social and behavior change communication
SOC	standard of care
STAC-EE	Scientific and Technical Advisory Committee on Ebola Experimental Interventions
STRIVE	Sierra Leone Trial to Introduce a Vaccine against Ebola, CDC
TB	tuberculosis
UN	United Nations
UNESCO	United Nations Educational, Scientific and Cultural Organization
USAID	U.S. Agency for International Development

Copyright © National Academy of Sciences. All rights reserved.

WAHO	West African Health Organization
WHO	World Health Organization
WMA	World Medical Association
ZEBOV	Zaire Ebola virus

Copyright © National Academy of Sciences. All rights reserved.

Summary[1]

The 2014–2015 Ebola epidemic in western Africa was the longest and most deadly Ebola epidemic in history, resulting in 28,616 cases and 11,310 deaths in Guinea, Liberia, and Sierra Leone. The Ebola virus, which causes fever, vomiting, diarrhea, impaired kidney and liver functions, and internal and external bleeding, has been known since 1976, when two separate outbreaks were identified in the Democratic Republic of Congo (then Zaire) and South Sudan (then Sudan). However, because all Ebola outbreaks prior to that in West Africa in 2014–2015 were relatively isolated and of short duration, little was known about how to best manage patients to improve survival, and there were no approved therapeutics or vaccines. There were a few potentially useful agents in 2014 that had been tested on animals, including nonhuman primates, and some very limited Phase 1 studies of the safety of vaccine candidates in humans. Given the nature of Ebola and its high mortality rate (ranging from 25 to 90 percent), it was not feasible to perform further testing of the safety or efficacy of these agents until the emergence of a natural outbreak of sufficient size and duration. The 2014–2015 Ebola epidemic presented such a situation.

The epidemic began in December 2013 when one child in Guinea was infected, likely from contact with bats. The child died in late December, and soon several family members and health care workers also became ill and died. By February 2014, the illness had spread to Conakry, the capital of Guinea, and in March 2014 Médecins Sans Frontières (MSF) was asked

[1] This summary does not include references. Citations for the discussion presented in the summary appear in the subsequent report chapters.

Copyright © National Academy of Sciences. All rights reserved.

to help identify the nature of the outbreak. MSF arranged for samples to be tested in Lyon, France, these samples came back positive for Ebola, and the World Health Organization (WHO) soon announced that the outbreak was caused by the Zaire species of the Ebola family. At the time, the WHO confirmed 49 cases of Ebola in Guinea, with 29 deaths. Soon, Ebola cases were confirmed in Liberia and Sierra Leone, and by June 2014 the epidemic was officially the largest in history, with 759 confirmed, probable, and suspected cases, including 467 deaths. The affected countries struggled to deal with the rapidly escalating epidemic and the growing number of patients, and MSF, which was providing the frontline treatment and infection control, warned that the epidemic was "out of control" and that ending the epidemic would require a massive international response.

In the summer of 2014, several international aid workers contracted Ebola and were evacuated to medical facilities in the United States and Europe, given unproven therapeutic agents, such as ZMapp and brincidofovir, and they appeared to survive at a higher rate than did African patients who contracted the virus. While the aid workers' survival was most likely due to the state-of-the-art supportive care that they received in the countries they were evacuated to, the use of these therapeutic agents sparked a call to make potential therapeutics available to the thousands of African patients suffering from Ebola. The WHO declared the epidemic a public health emergency of international concern on August 8, 2014, and shortly thereafter researchers and stakeholders began discussing whether and how to conduct clinical trials on potential Ebola therapeutics and vaccines; these discussions ultimately resulted in several teams conducting formal clinical trials in the Ebola affected countries during the outbreak. In October 2015, the National Academies of Sciences, Engineering, and Medicine (the National Academies) was asked by the Office of the Assistant Secretary for Preparedness and Response, the National Institute of Allergy and Infectious Disease, and the U.S. Food and Drug Administration of the U.S. Department of Health and Human Services to review and analyze the clinical trials that were conducted during the epidemic.

STUDY CHARGE AND APPROACH

The National Academies was charged with convening an expert committee to assess the value of the trials and to make recommendations about how the conduct of trials could be improved in the context of a future international emerging or reemerging infectious disease event (see Chapter 1 for the full Statement of Task). Over the course of 10 months, the 16-member committee held meetings in the United States, the United Kingdom, and Liberia, and developed seven recommendations about how to improve the clinical research response in an outbreak situation. The committee's recom-

Copyright © National Academy of Sciences. All rights reserved.

mendations focus on both the inter-epidemic period—the time before and between infectious disease events—and the epidemic period itself.

The committee deliberated from February to November 2016, during which time it held three 3-day public workshops in Washington, DC, London, and Monrovia; one 2-hour public webinar; and three 2-day closed meetings. The committee also solicited and considered written statements from stakeholders and members of the public as well as soliciting information regarding the clinical trials conducted by responsible clinical trial teams. Furthermore, the committee conducted an extensive literature review on relevant topics. (See Appendix A for more information on methodology.)

ASSESSMENT OF EBOLA CLINICAL TRIALS

The clinical trials that took place during the 2014–2015 Ebola epidemic were conducted in an atmosphere and on a timeline entirely different from most clinical trials. The fact that the trials were conducted at all is a demonstration of the ability of researchers, regulators, review boards, and communities to quickly work together when the need is pressing—but it was not easy, and there was avoidable conflict along the way. The trial teams should be praised for overcoming the immense logistical obstacles encountered while trying to design and implement trials in West Africa in the midst of a rapidly spreading, highly dangerous contagious disease. The limited health and health research infrastructure, fear, rumors, lack of trust, and supply chain hurdles were just some of the barriers that had to be addressed and overcome. Despite the successes, however, the overall scientific harvest of the therapeutic trials was described as "thin" in a special report in *Science*. None of the therapeutic trials ended with conclusive results on product efficacy, although the limited evidence from the ZMapp trials did trend toward a possible benefit. Given the resources, time, and effort put into these trials, they were not as successful as they could have been. While the research did yield some new information about Ebola, none of the trials were able to reach definitive conclusions about efficacy, and some of the inconclusive trials may have actually set back the search for safe and effective therapeutics. (See Table S-1 for further detail.)

The results of the vaccine trials were more fruitful. There are two Ebola vaccine candidates that current data suggest may be safe and immunogenic, though further data on safety and efficacy are needed (see Table S-2 for more detail). The Guinea ring vaccination study (this trial was also named; Ebola ça Suffit) showed suggestive efficacy, however, the trial was not designed to document long-term safety and efficacy because all participants were ultimately immunized and the protocol only followed participants out to day 84. The results of the PREVAIL trial, when available, will provide information on the long-term immunogenicity of the two vaccines studied,

Copyright © National Academy of Sciences. All rights reserved.

TABLE S-1 Investigational Ebola Therapeutic Agents in Formal Clinical Trials During the 2014–2015 Ebola Outbreak

Investigational Therapeutic	Trial Design	Results
Convalescent plasma	• Nonrandom, open-label<br>• Historical controls	The transfusion of up to 500 ml of convalescent plasma with unknown levels of neutralizing antibodies in 84 patients with confirmed Ebola virus disease (EVD) was not associated with a significant improvement in survival.
Favipiravir	• Multicenter proof-of-concept noncomparative trial<br>• Nonrandom, open-label<br>• Single-arm, historical controls	Efficacy and tolerance inconclusive.
Brincidofovir	• Multistage trial design with boundaries based on historical/contemporary controls with results guiding subsequent trial design<br>• Nonrandom, open-label<br>• Single-arm, historical controls	Efficacy and tolerance inconclusive due to small sample size.
TKM-130803	• Multistage trial design with boundaries based on historical/contemporary controls with results guiding subsequent trial design<br>• Nonrandom, open-label<br>• Single-arm, historical controls	Early results from the study, demonstrated that TKM-130803 was not effective in increasing the survival fraction above 50 percent; unlikely to demonstrate an overall therapeutic benefit to patients.
ZMapp	• Randomized, open-label<br>• Two arms: ZMapp + optimized standard of care (oSOC) versus oSOC only<br>• oSOC includes Favipiravir in Guinea	ZMapp showed promise as a possible effective treatment agent for EVD, but there were insufficient data to determine definitively whether it is a better treatment for EVD than supportive care alone.

Copyright © National Academy of Sciences. All rights reserved.

TABLE S-2 Investigational Ebola Vaccines in Formal Clinical Trials During the 2014–2015 Ebola Outbreak

Investigational Vaccine	Trial Design	Results
rVSV-ZEBOV	Trial 1 (Guinea Ring Vaccine Trial)<br>• open-label, cluster-randomized ring vaccination trial<br>• vaccines are "rings" (contacts/contacts of contacts) of confirmed Ebola cases<br>• Immediate versus deferred (21 days) vaccination<br>Trial 2 (CDC–STRIVE)<br>• Individually randomized, open-label<br>• Immediate versus deferred vaccination (18–24 weeks after enrollment)<br>Trial 3 (NIH PREVAIL)<br>• Randomized, double-blind, placebo-controlled<br>• 2 treatment arms—randomized 1:1:1 to ChAd3-EBO-Z, VSVDG-ZEBOV, or saline placebo	Overall results from the three trials:<br><br>While the ring vaccination study provided some evidence of efficacy, the trial was not designed to document long-term safety and efficacy because all participants were ultimately immunized and the protocol only followed participants out to day 84.<br><br>From preliminary results obtained from the PREVAIL I trial results, the antibody response peaked 1 month after vaccination and was sustained over the next 11 months, without any clear evidence of decline for the rVSΔG group; 70 to 80 percent of the cohort responded to the vaccination with an antibody response.<br><br>When the final immunogenicity data become available, the results of the PREVAIL trial will provide information on the long-term immunogenicity of the vaccines, including the one used in the ring vaccination study.
ChAd3-EBOZ	Trial – NIH PREVAIL<br>• Randomized, double-blind, placebo-controlled<br>• 2 treatment arms—randomized 1:1:1 to ChAd3-EBO-Z, VSVDG-ZEBOV, or saline placebo	Vaccine was well tolerated. At 1 month, 87 percent of the volunteers who received the cAd3-EBOZ vaccine candidate had measurable Ebola antibodies; the results show a robust antibody response to the vaccine that is maintained over a 12-month follow-up period and without evidence of adverse drug reactions other than the expected local injecting site reactions.

continued

Copyright © National Academy of Sciences. All rights reserved.

TABLE S-2 Continued

Investigational Vaccine	Trial Design	Results
Ad26.ZEBOV and MBA-BN-Filo	Trial – EBOVAC-Salone<br>• Staged Phase 3 study to gather information on the safety and immunogenicity of a heterologous prime-boost regimen. In this regimen, the immune system will be primed with the candidate vaccine Ad26.ZEBOV and later boosted with the candidate vaccine MVA-BN-Filo.	Initial Phase 1 studies suggest no adverse events. Phase 2 and 3 studies are ongoing.

including the one used in the ring vaccination study. These differences in the study designs and the value of the information generated highlight the importance of collaboration in future trials (see Chapter 4 for additional detail).

ETHICS OF CLINICAL RESEARCH DURING AN EPIDEMIC

Planning and conducting clinical research during the Ebola epidemic required confronting a number of ethical issues. First and foremost, stakeholders debated whether it was ethical to conduct clinical trials at all in the midst of a public health emergency. Many, including the members of the WHO Ethics Working Group, argued that there was an ethical obligation to conduct research during the epidemic. On the other hand, humanitarian organizations providing care in the treatment units were skeptical of activities that drew effort away from their mission of providing clinical care to the most people possible. Properly designed clinical research is essential for answering questions about disease processes and for evaluating the safety and efficacy of potential therapeutics and vaccines; indeed, for diseases such as Ebola, an outbreak or epidemic presents the only opportunity to conduct such research. The high mortality of Ebola and the uncertainty about how the epidemic would progress produced a sense of urgency to quickly identify effective therapeutics or vaccines. Despite this sense of urgency, research during an epidemic is still subject to the same core scientific and ethical requirements that govern all research on human subjects. The committee identified seven moral requirements that should guide all clinical research including research conducted during epidemics: scientific

Copyright © National Academy of Sciences. All rights reserved.

and social value, respect for persons, community engagement, concern for participant welfare and interests, a favorable risk–benefit balance, justice in the distribution of benefits and burdens, and post-trial access.

There was a great deal of disagreement among researchers over how clinical trials should be designed during the Ebola epidemic, particularly over whether trials should use randomization and concurrent control groups. Randomized controlled trials (RCTs) are the preferred research design because they allow researchers to directly compare the outcomes of similar groups of people who differ only in the presence or absence of the investigational agent. However, many stakeholders argued that RCTs would be unethical in the context of the Ebola epidemic. The arguments against RCTs were varied, but most were primarily based on one central assumption: that it was unethical and unacceptable to deprive patients of an agent that could potentially prevent or treat Ebola, given the high mortality rate and lack of known and available treatment options.

This committee found, however, that the RCT was an ethical and appropriate design to use, even in the context of the Ebola epidemic. First, at the beginning of the epidemic it was unknown whether any of the potential agents were safe or effective. This position of "equipoise"—genuine uncertainty in the expert medical community over whether a treatment will be beneficial—is the ethical basis for assigning only some participants to receive the agent. If the relative risks and benefits of an agent are unknown, participants who receive the experimental agent may receive a benefit or may be made worse off. Providing the experimental agent to all would expose all participants to potentially harmful effects. Second, some stakeholders argued that communities would not understand or accept RCTs. However, the committee found that while there was a great deal of mistrust and fear within the affected communities, early, respectful, appropriate communication and engagement could, and did, result in community buy-in and acceptance of RCTs. Finally, the committee found that using a randomized control group as a comparator to the group receiving the experimental agent is the most reliable way to determine whether an agent is effective. Other methods of comparison that were proposed—such as using historical data—are unlikely to produce reliable results because of issues with varying mortality rates and differences in supportive care over time. The committee concluded that randomized, controlled trials are the most reliable way to identify the relative benefits and risks of investigational products and, except when rare circumstances are applicable, every effort should be made to implement them during epidemics. The committee notes that randomization can take many forms (i.e., not just simple randomization) and that trial teams will need to assess the context in which they are implementing trials to determine the best form of randomization (further discussed in Chapter 2).

Copyright © National Academy of Sciences. All rights reserved.

RECOMMENDATIONS

The mobilization of a rapid and robust research response during the next epidemic will depend not just on what happens during the epidemic, but on what happens before or between epidemics. The committee's recommendations cover both the epidemic and inter-epidemic periods and focus on three main areas: strengthening capacity, engaging communities, and facilitating international coordination and collaboration. Focusing on these three areas will improve the national and international response to the next epidemic. The degree of improvement in the response will be largely dependent on the investments made in research and development (R&D) on diagnostics (which we do not discuss further), therapeutic agents, and vaccines and on the success in identifying promising candidates in these areas to bring forward to human clinical trials when an outbreak strikes. For a disease like Ebola, where experimental human infections cannot be used to facilitate the conduct of clinical trials of investigational products, an outbreak provides the only opportunity to assess the efficacy of drug candidates in patients and assess the protection capability of vaccines.

Strengthening Capacity

The three countries most affected by the Ebola epidemic—Guinea, Liberia, and Sierra Leone—were among the countries that were perhaps the least equipped to respond to an epidemic or to support clinical research during an epidemic. They did not have the infrastructure, human resources, or experience to deal with the public health and health care demands of the epidemic, let alone to facilitate research. The committee found that there were six major capacity challenges that hindered and slowed the research response to the Ebola epidemic: (1) lack of clinical experience with Ebola; (2) poor surveillance and laboratory capacity; (3) deficiency of crucial health systems infrastructure and health care workers; (4) small pool of clinical research experts and very limited prior experience in the conduct of clinical research; (5) ethics review boards in the countries that lacked the resources, experience, training, and information management systems that were needed to evaluate a sudden onslaught of clinical research proposals; and (6) lack of experience and expertise in completing the various and complex legal and bureaucratic steps in clinical trial conduct, e.g., contract negotiations.

First, the affected countries lacked experience with Ebola; although there is some evidence that Ebola virus was present in the region before 2014, the countries had not experienced a prior outbreak and certainly not an epidemic of such magnitude and duration. Second, the countries did not have the surveillance systems and laboratory capacity necessary to quickly

Copyright © National Academy of Sciences. All rights reserved.

identify the source of the illness at the beginning of the outbreak, and, once the epidemic was under way, the lack of surveillance and laboratory capacity continued to impede attempts to monitor and control the epidemic. In order to address this deficiency, the committee recommends that during the inter-epidemic period funders and development agencies should provide resources and assistance for the development of core capacities in low- and middle-income countries. Because clinical research is dependent on a functioning health care system, it is not enough to invest in the research enterprise in the absence of improving the quality of the health care workforce and the facilities in which care is provided. When international assistance to strengthen capacity is involved, it will likely require a combination of sources from the research and the international development/assistance communities.

Recommendation 1
Support the development of sustainable health systems and research capacities—Inter-epidemic
To better prepare low-income countries to both respond to future outbreaks and conduct foundational research, during the inter-epidemic period (as covered in 2005 International Health Regulations [IHR 2005]), major research funders and sponsors (e.g., U.S. National Institutes of Health and comparable public and private research funders) and development agencies (e.g., U.S. Agency for International Development [USAID] and comparable public and private development funders) should collaborate with the World Health Organization (WHO) and regional centers of excellence to

1. **Assist in monitoring and evaluating the development of national and regional core capacities under IHR 2005 and**
2. **Provide financial and technical assistance to the extent possible or establish a financing mechanism to help build sustainable core capacities at the intersection of health systems and research (e.g., diagnostics, surveillance, and basic epidemiology).**

Third, health infrastructures were poor, and there was a major shortage of health care personnel, which was exacerbated when personnel became infected and died as the epidemic progressed. The shortage of workers hindered the countries' ability to care for patients and to implement infection control measures, especially in the setting of containment and the need to wear personal protective equipment, and to collect patient-level data that could be used to inform treatment protocols in real time. The committee concluded that, while recognizing the challenges of collecting and recording patient data, it is critical to do so in order to document the natural history of the evolving epidemic and to provide clues to better patient management.

Copyright © National Academy of Sciences. All rights reserved.

The committee developed two recommendations aimed at facilitating data collection during an epidemic.

Recommendation 2a
Develop memoranda of understanding[2] to facilitate data collection and sharing—Inter-epidemic
Research funders, sponsors, national governments, and humanitarian organizations should work together with the World Health Organization (WHO) to develop memoranda of understanding during the inter-epidemic period to improve capacity to collect and share clinical data, with all necessary provisions to protect the privacy of individuals and anonymize data for epidemiological research.

Recommendation 2b
Provide resources to enable data collection and sharing—Epidemic
At the start of an outbreak, developed countries, research funders, and sponsors should work together with national and international health care providers responding to an outbreak, to provide the additional resources and personnel needed to enable systematic data collection on routine care practices and outcomes. Data collection should begin as soon as possible, and data should be shared and coordinated in a central database to advance an understanding of the natural history of the disease and of the best practices for standard of care. This information should also be used to inform protocols for clinical trials.

The final three capacity challenges that the committee identified are distinct but interrelated issues. The three countries had a small pool of clinical research experts and very limited prior experience in the conduct of clinical research. Ethics review boards in the countries lacked the resources, experience, training, and information management systems that were needed to evaluate a sudden onslaught of clinical research proposals. Finally, the countries' lack of clinical research experience and expertise meant that completing bureaucratic and legal requirements took time and delayed the beginning of trials. To address these hurdles, the committee recommends that stakeholders work with low- and middle-income countries during the inter-epidemic period in order to help these countries develop the capacity to quickly negotiate legal agreements and complete

[2] Memoranda of Understanding: Documents whereby parties entering into a partnership agree to an intended common purpose or set of goals. This is sometimes seen as more of a moral agreement rather than a legally binding agreement, and thus it is usually not intended to have the enforceability of a legal document. Although useful as an overarching agreement that sets out the working principles between parties, other written agreements are necessary to create binding commitments.

Copyright © National Academy of Sciences. All rights reserved.

ethics reviews when an epidemic strikes. In addition to the necessary human capacity, there is also a need to develop clinical trial templates because even a well-resourced country would be challenged if it needed to solve all the design issues necessary to launch clinical trials in the middle of a rapidly evolving and perhaps rapidly concluding epidemic.

Recommendation 3
Facilitate capacity for rapid ethics reviews and legal agreements—Inter-epidemic
Major research sponsors should work with key stakeholders in low- and middle-income countries to

- **Build relationships between local ethics boards and entities that could provide surge capacity for ethics review in the event of an emergency situation. Such efforts would include strengthening networks of ethics boards in a region or connecting local and outside ethics boards, agencies, or experts. Memoranda of understanding setting forth who will provide what services and how decisions will be made should be executed in the inter-epidemic period.**
- **Establish banks of experts in negotiation of clinical trial and material transfer agreements, and other essential components of collaboration, who are willing to offer pro bono advice and support to counterparts in countries affected by outbreaks.**
- **Develop template clinical trial agreements reflecting shared understandings about key issues such as data sharing, post-trial access to interventions, storage and analysis of biospecimens, and investments to build local capacity.**

In addition to the potential sources of experts in ethical review and the negotiation of clinical trial and material transfer agreements within schools of medicine and public health with extensive experience conducting clinical trials in low-resource settings, the nongovernmental organization Public Interest Intellectual Property Advisors, which provides pro bono legal advice to low- and middle-income countries regarding health research and contracts, and the Council on Health Research for Development, through its program on Fair Research Contracting, can be engaged to assist in these efforts, but will themselves require funding resources to participate.

Although the committee focused its capacity recommendations specifically on capacity for research, it acknowledges that public health, clinical care, and clinical research are all important and interconnected components of a strong health system. Building capacity for research cannot—and should not—be separated from building health systems capacity in general, and efforts to strengthen research capacity without improving the general public health and clinical care infrastructure may negatively affect the per-

Copyright © National Academy of Sciences. All rights reserved.

Integrating Clinical Research into Epidemic Response: The Ebola Experience

ception of clinical research activities and undermine their impact. With this in mind, the committee recommends that during an epidemic—and, more effectively, in an inter-epidemic period—building capacity for research be partnered with building capacity in the larger health system in general. This includes strengthening the educational institutions for health care professionals, from physicians, nurses, and midwives to laboratory technicians and public health professionals.

Recommendation 4
Ensure that capacity-strengthening efforts benefit the local population—Epidemic
When the health care services of a population need to be enhanced or augmented in order to support the conduct of research, development organizations (e.g., USAID), international bodies, and other stakeholders should partner with national governments to ensure that capacity-strengthening efforts are not limited to services that solely benefit study participants.

Finally, research systems should be incorporated into these countries' emergency preparedness and response systems. This committee's set of recommendations for actions to strengthen capacity for response and research is intended to provide the basis for cooperative initiatives and a rational partition of primary responsibility among national health authorities, the WHO, and other supranational and international partners involved in health care, public health, and R&D for therapeutics and vaccines, including the academic and private sectors; it is now up to these entities to seize the moment to engage and to invest the critical resources needed to strengthen capacity in low- and middle-income countries for the benefit of all in terms of creating national, regional, and global public goods. There is no doubt that a considerable investment in a sustainable manner will be required and that low-income countries have very limited ability to contribute their own funds to the effort; however, these countries still need to be investing partners and to claim co-ownership.

Recommendation 5
Enable the incorporation of research into national health systems—Inter-epidemic
National governments should strengthen and incorporate research systems into their emergency preparedness and response systems for epidemic infectious diseases. The multilateral institutions (the World Health Organization [WHO] and the World Bank Group), regional and international development agencies, and foundations working in

Copyright © National Academy of Sciences. All rights reserved.

global health should support national efforts by providing expertise and financing.

Engaging Communities

During the Ebola epidemic, there was a great deal of fear, mistrust, and misunderstanding between the affected communities and the national and international response and research staff. Community members feared going to health care facilities for the treatment of Ebola, rumors spread that Ebola was deliberately brought to the region by foreigners, and some people defied government edicts intended to fight the epidemic, such as quarantine. Early missteps in messaging and a lack of engagement with the communities exacerbated the preexisting mistrust and hindered the response to the epidemic. Initial response efforts tended to be top down and did not take into account community traditions and beliefs—for example, mandatory cremation policies countered deeply held religious beliefs. Over the course of the epidemic, communication and community engagement improved, and this resulted in an improved acceptance of and participation in infection control and research efforts. The committee found that the success of clinical research is dependent on the community's understanding of, engagement in, and sense of involvement and respect in the process of planning and conducting research. The committee recommends that community engagement be prioritized during epidemic responses and that engagement be a continuous and evolving effort that begins at the outset of the epidemic.

Recommendation 6a
Prioritize community engagement in research and response—Epidemic
International and national research institutions, public health agencies, and humanitarian organizations responding to an outbreak should engage communities in the research and response by

1. **Identifying social science experts in community engagement and communications to lead their efforts to effectively engage and connect with communities affected by the epidemic.**
2. **Consulting with key community representatives from the outset of an outbreak to identify a range of local leaders who can participate in planning research and response efforts, help to map community assets, articulate how to infuse cultural and historical context into presentations, and identify gaps and risks in developing public health measures and designing research protocols. Consultations should be continued throughout the implementation phase by relevant actors to provide information as the outbreak evolves, provide feedback about progress and results, and inform and recommend changes to strategies based on feedback from the community.**

Copyright © National Academy of Sciences. All rights reserved.

3. **Coordinating within and across sectors, with national authorities and with each other to ensure alignment of social mobilization and communication activities with the overall response and research strategies, and that there is sufficient support and training to local leaders and organizations to engage communities in research and response.**

This process would no doubt be easier—and less fraught with problems of trust—if, during inter-epidemic periods, stakeholders invested more time, training, research, and funding into developing frameworks and strategies for community engagement and communication about health and public health that could be translated to the circumstances of an epidemic.

Recommendation 6b
Fund training and research into community engagement and communication for research and response—Inter-epidemic
The World Health Organization (WHO), international research institutions, governments, public health agencies, and humanitarian organizations should actively collaborate together to fund training and research for developing frameworks, networks, strategies, and action plans for community engagement and communication on public health and research that could inform and be mobilized during an epidemic.

Facilitating International Coordination and Collaboration

Events on a global scale generally require a global solution, which in turn necessitates international coordination and cooperation. There are no events for which this is more applicable than emerging infectious disease outbreaks, for even when they are in the beginning apparently localized, they can quickly become globalized. During the Ebola epidemic, research and response efforts were greatly affected by the relationships between international stakeholders and their ability to coordinate and collaborate. For example, there were a number of therapeutic candidates available at the beginning of the outbreak that required evaluation for safety and efficacy before they could gain regulatory approval, but the research conducted on these candidates was scattered and disjointed, with no agreed-upon approach for prioritizing the candidate agents, no infrastructure in place to rapidly implement trials, no consensus about trial design, and no coordination of trial locations. As a result, little more is known about the candidates now than before the trials began. If the international community had coordinated its research efforts and research could have been implemented sooner, there would have been a possibility that the trials would have identified a safe and effective therapeutic that might have been

Copyright © National Academy of Sciences. All rights reserved.

deployed during the epidemic, but more likely would have been available at the outset of the next one.

The R&D of therapeutics and vaccines is a long and expensive process. The process of drug development from bench to bedside is estimated to, on average, take at least 10 years and cost $2.6 billion,[3] with the likelihood of eventual licensing at less than 12 percent. Given the length of a typical infectious disease outbreak (weeks to months) and the length of time it takes to conduct drug discovery and assess efficacy and safety (years to decades), the odds that a new compound will be discovered and evaluated during an outbreak is vanishingly small. Therefore, making progress on the R&D of products—including therapeutics, vaccines, assays, and diagnostic tests—during the inter-epidemic period is the only way to ensure that promising candidates are ready for trials once an outbreak occurs. To this end, the committee recommends that an international coalition of stakeholders work during the inter-epidemic period to advise on and invest in priority pathogens to target for R&D, develop generic clinical trial design templates, and identify teams of clinical research experts who could be deployed to assist with research during an outbreak. The international coalition could also discuss and agree on methods to address administrative requirements that would rapidly become high priority during an emerging infectious disease outbreak, such as the location and management of a central data repository.

Recommendation 7a

Coordinate international efforts in research and development for infectious disease pathogens—Inter-epidemic

An international coalition of stakeholders with representation from governments, foundations, academic institutions and researchers, pharmaceutical companies, humanitarian organizations, and the World Health Organization (WHO) (such as the Coalition for Epidemic Preparedness Innovations) should work on the following planning activities to better prepare for and improve the execution of clinical trials conducted during infectious disease events:

1. **Advise on and invest in priority pathogens to target for research and development, and promote a process to ensure that, whenever possible, interventions should be brought through Phase 1 or Phase 2 trials prior to an outbreak.**
2. **Develop generic clinical trial design templates for likely outbreak scenarios. The reasoning and rationale behind the designs and the situations in which each would be best utilized should be discussed with representatives of ethics review boards, major humanitarian**

[3] The cost for developing a licensed product.

Copyright © National Academy of Sciences. All rights reserved.

organizations, and at-risk local communities to promote buy-in from stakeholders in advance of an outbreak.

3. **Develop a list of key experts in clinical research from different agencies and organizations who could be rapidly seconded to the coalition of stakeholders and deployed anywhere in the world when an outbreak is first identified.**

In addition to cooperating and collaborating in the preparation for an epidemic, it is essential that the international community coordinate its research efforts once an outbreak begins. Outbreaks of infectious disease can evolve, move, and end quickly; it is critical that well-designed trials of the most promising agents be implemented as soon as possible in order to maximize the likelihood of finding a safe and effective therapeutic or vaccine. To that end, the committee recommends that in the event of an emerging epidemic, an independent rapid research response workgroup should be convened by the international coalition of stakeholders. This workgroup would have the requisite expertise in order to appraise and prioritize products for trial, determine which trial designs are best suited for the circumstances, and monitor and evaluate the trials.

Recommendation 7b

Establish and implement a cooperative international clinical research agenda—Epidemic

In the event of an emerging epidemic the international coalition of stakeholders (in Recommendation 7a) should designate an independent multistakeholder rapid research response workgroup with expertise in the pathogen of concern, research and development of investigational interventions, clinical trial design, and ethics and regulatory review, and including representatives from the affected communities, to

1. **Rapidly appraise and prioritize a limited set of vaccine and therapeutic products with the most promising preclinical and clinical data for clinical trials;**
2. **Select a portfolio of trial designs that are best suited to the investigational agent(s) and the manifestation of the epidemic;**
   a. **The trial designs used should lead to interpretable safety and efficacy data in the most reliable and fastest way;**
   b. **Randomized trials are the preferable approach, and unless there are compelling reasons not to do so, every effort should be made to implement randomized trial designs; and**
3. **Monitor and evaluate clinical trials conducted during an outbreak to enhance transparency and accountability.**

Copyright © National Academy of Sciences. All rights reserved.

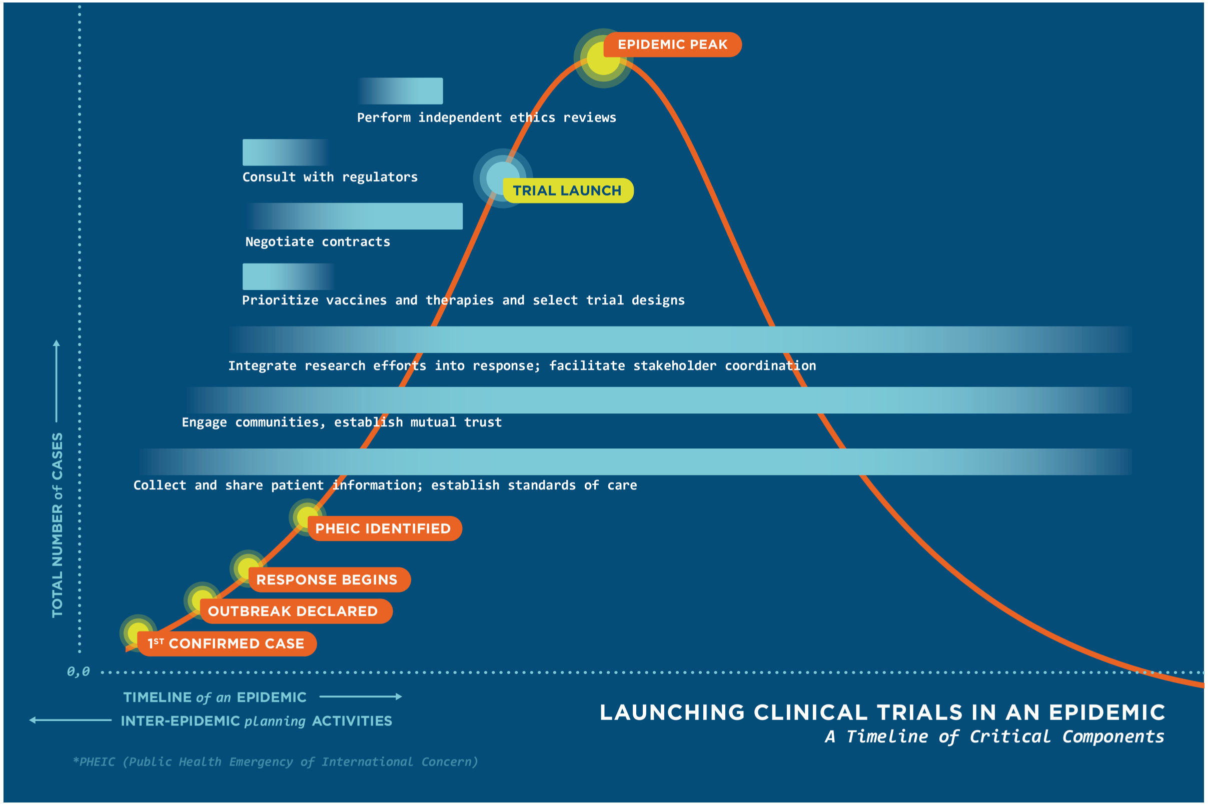

FIGURE S-1 A timeline of critical components: Launching clinical trials in an epidemic.

Copyright © National Academy of Sciences. All rights reserved.

There will be a need to connect the international coalition of stakeholders and its rapid research response workgroup with the other international response agencies during an epidemic and also with the leadership of national governments affected by an outbreak from the very onset of that outbreak in order to ensure that the affected population has a partnership position in the response. The responsibilities for the rapid research response workgroup should include making sure that resources for research are allocated efficiently and effectively, that the goals of the response and research activities are clear and agreed upon, and that community engagement and communication strategies are aligned. There should be thoughtful consideration given in the inter-epidemic period to developing an epidemic response stakeholder engagement strategy that includes a process for rapid mapping of key stakeholders at multiple levels (i.e., national to international and national to local leaders and opinion formers) at the onset of an epidemic. The goal is to encourage an open dialogue among all relevant stakeholders to achieve a better understanding of the nature of the crisis, each stakeholders' interests, and resources available for addressing the epidemic, inclusive of the potential for research in the response.

BEING PREPARED: LAUNCHING CLINICAL TRIALS IN AN EPIDEMIC

Through targeted exploration and analysis of scientific and ethical issues related to clinical trial design, conduct, and reporting during the 2014–2015 Ebola epidemic in West Africa, the committee learned key lessons that could be applied to future research conducted in settings where there is limited health care and research infrastructure. These lessons were then applied to developing the seven recommendations previously stated. Figure S-1 incorporates these recommendations into a visual representation of an idealized timeline of activities necessary to launch a clinical trial within the course of outbreak—represented as a standard epidemic curve.

The timeline is made up of seven key components that, if done in an efficient, coordinated, and timely manner, would enable trials to be launched before reaching the peak of the epidemic. However, attaining such a goal is unlikely without careful inter-epidemic planning and execution through a well-coordinated and collaborative effort from all involved parties. This includes national, international, and local representatives who each play a critical role in ensuring the global community is prepared to answer challenging questions through the conduct of research. It is through the development and implementation of sound clinical trials that best practices can be identified for improving clinical care for future populations both during and between public health emergencies.

Copyright © National Academy of Sciences. All rights reserved.

1

Introduction

Ebola has been known since 1976 when two outbreaks occurred, one in the Democratic Republic of the Congo, then known as Zaire, and the other in what is now South Sudan (Johnson et al., 1977). Ebola is a serious illness, transmitted from person to person by direct contact with infected body fluids, with a high mortality rate even with good clinical care (see Box 1-1). Until 2014, however, previous outbreaks had been limited in size and duration, occurring in relatively isolated communities in Central Africa and Uganda, with at most a few hundred cases and deaths in each outbreak but no cross-border or international spread (CDC, 2016c). Much was learned about the virus from previous outbreaks, but there was little knowledge of case management or the clinical sequelae among survivors because these outbreaks affected a limited number of individuals and were contained in isolated settings. The opportunity to make clinical observations was therefore restricted. Research on drugs and vaccines for Ebola was accordingly also limited in support and scope; the research was primarily focused on early preclinical development and was not a particularly high priority for the major international medical research organizations, including military health research institutes in the United States and elsewhere, or for the pharmaceutical industry (Burki, 2011). There was, nonetheless, some steady progress, including nonhuman primate challenge studies on investigational therapeutic agents and vaccines and also some very limited human Phase 1 studies of vaccine candidates at the beginning of 2014 (Gebre et al., 2014). Because of the nature of the disease, there was no way to create an experimental human infection model to test the efficacy of these products. Testing these products would require that a natural outbreak of

Copyright © National Academy of Sciences. All rights reserved.

BOX 1-1
Ebola Virus Disease

Ebola virus disease is a serious, acute illness characterized by fever, vomiting, diarrhea, impaired kidney and liver functions, and internal and external bleeding. Ebola is introduced to humans through contact with the blood, bodily fluids, or organs of infected animals, such as chimpanzees, gorillas, fruit bats, antelope, and a variety of bush mammals that are often consumed as food. It then spreads person to person through direct contact with the bodily fluids (e.g., blood, secretions) of infected persons, including those who are already deceased and harbor a high viral load. The time between infection and onset of symptoms ranges from 2 to 21 days. The initial symptoms of Ebola—fever, fatigue, muscle pain, sore throat, and headache—are similar to the symptoms of many common infections such as malaria, typhoid fever, and meningitis. Mortality from Ebola is variable, ranging from 25 to 90 percent, depending on the strain of the virus, the condition of the host (e.g., age; children of age <6 years are at higher risk of death), and the availability of expert supportive care, including fluid and electrolyte management. Those who survive the disease may continue to harbor the virus in immunologically protected sites such as the central nervous system, eyes, and testes and can still transmit it.

SOURCES: CDC, 2015b; WHO, 2016a.

sufficient size and duration occurred and that researchers could quickly mobilize products, protocols, and participants and implement clinical trials before the outbreak came under control and transmission was halted. And no one knew when, where, or whether this situation would present itself—until the epidemic of 2014–2015.

Conducting clinical research can often seem secondary to addressing the immediate health needs of patients, if not a distraction and unnecessary impediment to public health control activities and patient care in the midst of a public health emergency. However, research is an essential component to epidemic response, as it is the only way to learn how to improve care for current and future patients and to potentially prevent an epidemic from occurring again (Lurie et al., 2013). This is especially true for a disease like Ebola because there were no proven safe and effective therapeutic products or vaccines when the epidemic began. An epidemic, despite the often chaotic environment, is an opportunity to test the efficacy of vaccines and therapeutics that are currently in development. If research quickly reveals a safe and effective therapeutic agent or one that is superior to any available at the time, current patients could reap the benefits as soon as the interven-

Copyright © National Academy of Sciences. All rights reserved.

tion could be approved, made available, and delivered to those in need. The discovery of a safe and effective vaccine could protect people who are at immediate risk of infection as well as prevent future generations from contracting the disease.

There are established principles for conducting scientifically sound, ethical clinical research. For many years these have been reviewed and refined for the international context, in particular when research is sponsored by high-income countries for conduct in low-income countries. For example, the Declaration of Helsinki states that researchers must obtain the "freely given informed consent" of research subjects and that medical research "must conform to generally accepted scientific principles" and "be preceded by careful assessment of the predictable risks and burdens to the individuals and groups involved" (WMA, 2013, p. 2192). During an epidemic some of the standard practices of research may need to be accelerated or modified in order to work in the specific context of the community and disease. For example, informed consent procedures may need to be sped up or abbreviated, or consent by proxy may be deemed appropriate in situations in which patients are not able to give consent. However, as discussed in more detail in Chapter 2, the core ethical and scientific principles of research must undergird efforts even in the midst of an epidemic. Doing so helps to ensure that research can "quickly and definitively determine the safety and efficacy of interventions and thus provide access for the greatest number of patients to the most effective therapies in the shortest possible time" (Lane et al., 2016, p. 2). In addition, adhering to ethical principles such as "respect for volunteers and study communities, the value of informed consent, and the need for collaborative partnership with affected communities" helps to ensure that affected communities are not exploited and that the researchers gain the trust and buy-in of the community (Lane et al., 2016, p. 2).

Every epidemic is different in terms of the communities affected, the transmission and mortality rates, and the availability of potential treatments (KFF, 2014). The 2014–2015 Ebola epidemic was different from all previous Ebola outbreaks—it was unpredictable and fast moving, crossed borders, affected large numbers of people, was highly deadly, and was exacerbated by the lack of local experience, resources, infrastructure, and the limited number of experienced researchers (Heymann and Wertheimer, 2014). However, the issues raised by conducting research in the midst of the epidemic were not unique to Ebola. The same scientific and ethical questions have arisen in various other epidemics, ranging from the HIV/AIDS epidemic in the early 1980s to the current Zika epidemic (Deloffre, 2016; Wainberg et al., 2014). Much can be learned from prior debates about conducting research during public health emergencies, in resource-poor settings, or among a population of desperately ill patients. Researchers should not consider each epidemic to be sui generis; rather, the response to

Copyright © National Academy of Sciences. All rights reserved.

each new epidemic should take advantage of the fact that various epidemics have common elements and build upon the knowledge gained from previous experiences.

CHARGE TO THE COMMITTEE AND STUDY SCOPE

In October 2015, the Office of the Assistant Secretary for Preparedness and Response, the National Institute of Allergy and Infectious Diseases, and the U.S. Food and Drug Administration of the U.S. Department of Health and Human Services charged the National Academies of Sciences, Engineering, and Medicine (the National Academies) with developing a consensus report that explores and analyzes scientific and ethical issues related to clinical trial design, conduct, and reporting. The sponsors stipulated that particular emphasis be given to clinical trials for investigational therapeutic and vaccine candidates for Ebola conducted by the international community in settings where there is limited health care and research infrastructure, focusing on research conducted in Guinea, Liberia, and Sierra Leone during the 2014–2015 epidemic. The information and analysis presented in this report is meant to inform guidelines and best practices for clinical trials of therapeutics and vaccines conducted in response to future outbreaks and epidemics in low-resource settings (see Box 1-2 for the full Statement of Task).

BOX 1-2
Statement of Task

An ad hoc committee of the National Academies of Science, Engineering, and Medicine will review and conduct an analysis of the clinical trials conducted during the 2014–2015 Ebola virus disease outbreak in West Africa. The final deliverable from this committee will be a consensus report that explores and analyzes the scientific and ethical issues related to clinical trial design, conduct, and reporting. Particular emphasis will be given to clinical trials for vaccine and therapeutic candidates for Ebola conducted by the international community in settings where there is limited health care and research infrastructure.

The committee will achieve its objectives by organizing and conducting meetings, interviews, and discussions with key informants in the international research community and in affected countries, including perspectives from government agencies, nongovernmental entities, and others. The final report of the committee will be based on the information gathered at these meetings and key informant interviews and review of the literature and other relevant documentation and communications.

Copyright © National Academy of Sciences. All rights reserved.

BOX 1-2 Continued

In summary the report will

- Assess the scientific value of the different Ebola vaccine and therapeutic clinical trials conducted in Guinea, Liberia, and Sierra Leone and the data derived from them.
    - Describe the specific context (such as disease pathogenicity, high case fatality, political and health system context, and public opinion) and the ethical and scientific practices and considerations related to the design and conduct of each Ebola clinical trial.
    - To the extent possible, characterize how these considerations have been addressed in similar contexts previously, drawing on case studies of clinical trials conducted during prior disasters to identify transferable best practices.
    - Compare pragmatic, ethical, and scientific practices and considerations made in the context of each Ebola clinical trial against existing best practices to identify additional best practices and make recommendations on opportunities for improving future clinical research conducted during public health emergencies.
- Make recommendations for how, in the context of an international emerging or reemerging infectious disease event, clinical trials can best be prioritized and conducted to (1) speed data collection; (2) inform clinical management of patients; (3) assess the safety, efficacy, and effectiveness of therapeutics and vaccines; and/or (4) improve/augment outbreak control efforts.
    - Where possible, identify scientific and ethical principles and practical guidance for clinical trial practices and protocols that balance the rapid need for new, useful information with ethical considerations and scientific rigor amid an emerging and rapidly evolving infectious disease event.
    - Where possible, identify new ideas for innovative approaches to research in emergency contexts and to alternative methods to evaluate treatments and vaccines.
    - Such guidance should include options that facilitate a more flexible and accelerated approach.
- Address whether adjustments to scientific or ethical standards are appropriate in the conduct of research in outbreak settings and, if so, under what circumstances. If such adjustments are found to be appropriate, specify
    - How those adjustments should be decided and implemented; and
    - Current and/or future consequences of any such adjustments to patients themselves or to prevention and treatment strategies.
- Identify opportunities for collaborative investment to achieve long-term ethical and scientific gains from clinical trials conducted during emerging infectious disease events.
- Develop recommendations for planning activities during the inter-epidemic period to better prepare for and improve the execution of clinical trials during future infectious disease public health emergencies.

Copyright © National Academy of Sciences. All rights reserved.

Integrating Clinical Research into Epidemic Response: The Ebola Experience

To respond to this charge, the National Academies convened a 16-member ad hoc committee composed of experts from a range of disciplines. Members of the committee have expertise in clinical trial conduct and design, biostatistics, infectious disease, public health, health systems, and bioethics as well as considerable experience working in low- and middle-income countries. The committee included members from Africa, Europe, and North America.

STUDY METHODOLOGY

The committee deliberated from February to November 2016, during which time it held three 3-day public workshops in Washington, DC; London; and Monrovia; one 2-hour public webinar; and three 2-day closed meetings. The committee also solicited and considered written statements from stakeholders and members of the public, as well as soliciting information regarding the clinical trials conducted from responsible clinical trial teams. Furthermore, the committee conducted an extensive literature review on relevant topics. (See Appendix A for more information.)

CONTEXT OF THE 2014–2015 EBOLA EPIDEMIC

The 2014–2015 Ebola epidemic, the largest in history, began in December 2013 when a toddler in Guinea became ill, likely as a result of contact with a bat (WHO, 2015d). He died on December 28, 2013, and, soon after, members of his family and several health care workers who treated them also became ill and died. By the end of February 2014, the illness had spread to Conakry, the capital, as well as to other villages and cities. On March 22, the cause was confirmed to be the Zaire species of the Ebola family, and the following day the World Health Organization (WHO) publicly announced the outbreak. The WHO's announcement provided an official count of 49 cases with 29 deaths and noted that reports of suspected cases in Liberia and Sierra Leone, which share a common border with Guinea, were being investigated (WHO, 2014c). Within a few days, Ebola cases were confirmed in both countries (MSF, 2015). By the time the epidemic was nearing its end in early 2016, the epidemic was responsible for 28,616 cases of Ebola, with 11,310 deaths (WHO, 2016a). The WHO declared the emergency phase of the epidemic to be over on March 29, 2016, though flare-ups continued to occur through April (WHO, 2016a).

The three countries at the epicenter of the 2014–2015 epidemic—Guinea, Liberia, and Sierra Leone—were all ill equipped to handle such a serious and quick-moving epidemic. Given its size and rapid spread, the epidemic would have been a challenge for any country to contain. Before the epidemic Liberia and Sierra Leone were in the process of rebuilding

Copyright © National Academy of Sciences. All rights reserved.

Integrating Clinical Research into Epidemic Response: The Ebola Experience

after long civil wars that had spilled over into Guinea, and all three countries suffered from political and social instability, weak health care systems, extreme poverty, and poor infrastructure (International Crisis Group, 2015). In the 2014 United Nations Human Development Index, which ranks 187 countries on the basis of life expectancy, income per capita, and years of schooling, Guinea was ranked 179th, Liberia 175th, and Sierra Leone 183rd (UNDP, 2014). The health systems of each country were weak, with critical shortages of medical doctors and hospital beds (see Table 1-1). Health care facilities were unevenly distributed, inadequately staffed, and lacked the supplies necessary to treat patients and protect health care workers (CIA, 2016; International Crisis Group, 2015). The 2014–2015 Ebola epidemic in the three countries moved quickly, was difficult to contain, and lasted longer than any previous outbreak. In contrast, outbreaks of Ebola in Nigeria, Senegal, and Mali in, respectively, July, August, and October 2014 were contained relatively quickly due to a high state of alert, more robust health systems and public health capacity, and the ability to mobilize and deploy the necessary human and laboratory resources rapidly from within and outside of these countries (WHO, 2015e).

The Ebola epidemic in Guinea, Liberia, and Sierra Leone exposed and strained those countries' already fragile health care and public health systems, and the situation quickly deteriorated: the shortage of staff was exacerbated when workers became infected or, in some instances, refused

TABLE 1-1 Data Depicting the Deficit of Medical Doctors and Hospital Beds in the Ebola-Affected Countries at the Time of the Ebola Outbreak in Comparison to Higher-Income Countries

Country	Medical Doctor Density	Hospital Bed Density
Guinea	0.1 physicians/1,000 population (2005)	0.3 beds/1,000 population (2011)
Liberia	0.01 physicians/1,000 population (2008)	0.8 beds/1,000 population (2010)
Sierra Leone	0.02 physicians/1,000 population (2010)	0.4 beds/1,000 population (2006)
United States	2.45 physicians/1,000 population (2011)	2.9 beds/1,000 population (2011)
United Kingdom	2.81 physicians/1,000 population (2013)	2.9 beds/1,000 population (2011)
France	3.19 physicians/1,000 population (2013)	6.4 beds/1,000 population (2011)

SOURCE: CIA, 2016.

Copyright © National Academy of Sciences. All rights reserved.

to report to work due to fear of infection; health facilities closed due to a lack of staff or could offer only the most basic care; and, as the number of cases increased, Ebola patients were denied treatment and turned away from facilities (MSF, 2015). As the epidemic progressed, the inability of the countries' systems to control the epidemic became clear. Foreign medical staff and aid organizations stepped in to provide support and direct care. Médecins Sans Frontières (MSF), as one of the few agencies with direct experience responding to Ebola outbreaks in the past, had staff with established expertise in treating Ebola. As a result of its experience, MSF and its staff was quickly at the epicenter of the outbreak (Hofman and Au, 2017). As the affected area and number of infected patients grew MSF staff and resources were soon spread thin (MSF, 2015). Health care facilities often lacked personal protective equipment to prevent transmission to health care workers, and as workers became infected, the facilities acted as amplifiers of the virus (WHO, 2015b). In addition, the treatment units lacked the staff and the equipment to provide the necessary supportive care—particularly intravenous fluids and electrolyte management—and patient demand far outpaced the availability of treatment beds (WHO, 2015b). The opening of treatment centers was delayed by a lack of funding, and patients traveled for miles over poor roads in attempts to get care.

In addition to the lack of facilities, staff, and equipment, the response to the Ebola outbreak was made more difficult because of such issues as stigma, fear, rumors, traditional practices, mistrust of authorities and foreign response workers, and mistakes made in engaging communities and community leaders. Stigma took on different forms in different communities, but it complicated the response efforts in all three countries. For example, in Guinea, Ebola initially spread among the Forestièrs (people from the Forest region), a marginalized and suppressed ethnic group. Guineans from the rest of Guinea initially saw Ebola as a disease of "immoral" people (Fairhead, 2015). As a result, Guineans were slow to admit that Ebola could infect their communities and resisted taking measures that could prevent the spread of the virus (Taddonio, 2015). Also in Guinea, rumors spread that foreign response workers were deliberately spreading the virus through disinfection campaigns or that they were killing people in order to harvest their organs (WHO, 2015b). Traditional burial practices in the region—which include kissing, touching, and washing the body—were responsible for many secondary infections (Manguvo and Mafuvadze, 2015). One traditional healer's funeral, which drew hundreds of mourners from miles around, may have been the source of as many as 365 subsequent Ebola deaths (WHO, 2016c).

The mistrust of authorities—which was exacerbated by a heavy-handed government response which included quarantine, mass cremations, and the use of military force—resulted in community resistance to response

Copyright © National Academy of Sciences. All rights reserved.

workers (Pellecchia et al., 2015). Authorities often failed to establish effective communication with community members and could not answer their concerns, and the community sometimes did not comply with the infection control efforts of authorities and actually avoided contact with health care facilities or workers (RAS, 2015). Patients who entered Ebola treatment units (ETUs) were sequestered away from the outside world and all too often never emerged except for being cremated and buried safely without the involvement of family. The process, while understandable from a public health perspective, served to drive a wedge between the health system and the community, and the result was disastrous. Patients ran from authorities, or families hid them away, and burials took place in secrecy and without precautions—and transmission of Ebola increased (WHO, 2015b). Community resistance even took the form of violence. For example, in Guinea, treatment facilities and equipment were vandalized, foreign epidemiologists were run out of town by an angry mob, and, in one instance, an eight-member response team was murdered in a village (McCoy, 2014; WHO, 2015b). Nearly 1 year into the epidemic, new cases continued to emerge in both new areas and areas that had already been affected, while communities were overwhelmed by unmet needs—bodies lay uncollected on the streets, patients were dying outside of full treatment units, and orphaned children were left to die (WHO, 2015b).

"Unprecedented" and "Out of Control"

The first victim of the outbreak contracted Ebola in the village of Meliandou in December 2013, and the virus soon spread to the nearby towns of Guéckédou and Macenta and beyond as contacts dispersed to other locations. But it was not until early March 2014 that the Ministry of Health seriously confronted the mysterious spreading illness, and not until March 14 that MSF was asked for help (Baize et al., 2014). Upon learning of the outbreak, MSF quickly sent emergency teams into the field, with the first team arriving in Guéckédou, Guinea, on March 18 (MSF, 2015). Concerned about the likelihood that it was Ebola, MSF arranged to obtain and ship samples for diagnosis to the Inserm laboratory in Lyon, France, where the diagnosis was confirmed and announced by MSF on March 22. In the WHO's first official outbreak report on March 23, the organization reported that the Ministry of Health, WHO, and other partners were taking steps to control the outbreak and that teams had been deployed to search for and manage cases, while MSF actually began to mobilize the capacity to receive affected patients (WHO, 2014b). The WHO country office in Guinea classified the outbreak as a grade 2 emergency: "a single or multiple country event with moderate public health consequences that requires a moderate WHO country office response and/or moderate international

Copyright © National Academy of Sciences. All rights reserved.

WHO response" (WHO, 2013). However, in late March, a case was confirmed in the capital city of Conakry, 400 miles away from the initial index cases (MSF, 2015). By March 31, MSF declared that the outbreak was unprecedented because of the geographic spread of the cases (MSF, 2014b). One day later, despite the appearance of confirmed or probable cases in Sierra Leone and Liberia, WHO spokesman Gregory Hartl in Geneva downplayed the outbreak, saying that it was relatively small, neither an epidemic nor unprecedented, and that the appearance of Ebola in a capital city was not a new phenomenon (Samb, 2014). MSF continued to warn about the very real potential for a humanitarian disaster, saying that the distribution of cases in Guinea and Liberia showed that the epidemic was already of a magnitude never seen before and characterizing the response of state authorities and international organizations as minimal (Samb, 2014).

By mid-May 2014 the outbreak seemed to be waning: there was a slight decline in cases in Guinea, Liberia had not reported a new case since April 9, and there were no confirmed cases in Sierra Leone (WHO, 2015b). However, the hope that the three-country outbreak was resolving proved to be wishful thinking. On May 26, the first case was confirmed in Sierra Leone, and it soon became clear that the disease had already been present in the country for some time (Williams, 2015). Within 3 days, from May 27 to May 30, the cases of Ebola reported in Sierra Leone tripled from 16 to 50 (Boston Children's Hospital and Harvard Medical School, 2016). There was also an escalation of new cases in Liberia and Guinea, and by late June, Ebola patients were identified in more than 60 separate locations across the three countries (MSF, 2014a). On June 21, MSF declared that the epidemic was "out of control," and it warned that it was reaching the limit of what it could do alone; MSF said it could no longer respond or send teams to new outbreak areas and argued that containment would require massive assistance from local governments and international organizations (MSF, 2014a). On later reflection, MSF director of operations Bart Janssens said that it was like "shouting into a desert" as their appeal for help went unanswered (MSF, 2015). The WHO, the U.S. Centers for Disease Control and Prevention (CDC), and other international organizations sent small numbers of experts to help in the response (CDC, 2016a; WHO, 2014a), but the vast majority of the day-to-day treatment of patients was being provided by local health workers and volunteers from private aid organizations such as MSF as well as by smaller groups such as Samaritan's Purse and SIM (Serving in Mission) (MSF, 2015).

By late June 2014, the West Africa outbreak was officially the largest in history, with 759 confirmed, probable, and suspected cases, including 467 deaths (WHO, 2014d). (See Figure 1-1 for a graphic display of the progression of the epidemic in the three countries.) On July 24, the director-general of the WHO declared the outbreak to be a grade 3 emergency (WHO, 2014c). International attention to the Ebola epidemic was

Copyright © National Academy of Sciences. All rights reserved.

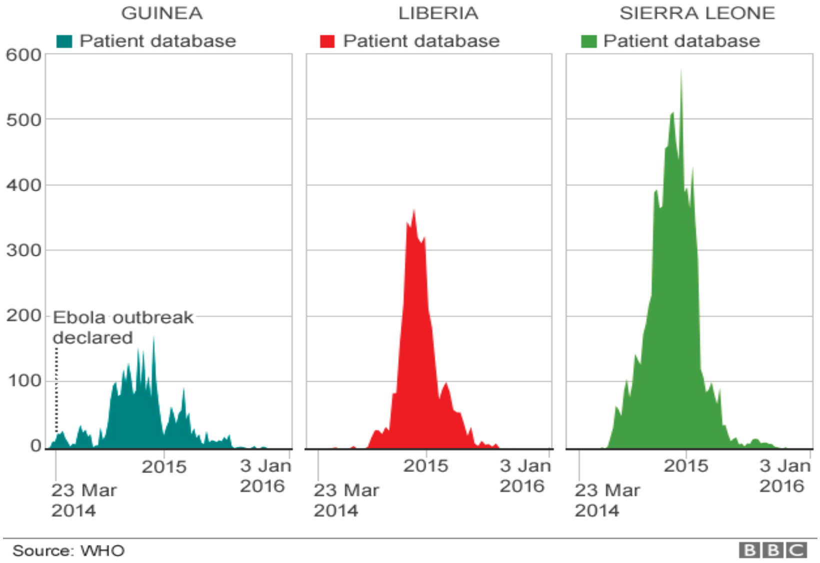

FIGURE 1-1 Weekly reported Ebola cases during the 2014–2015 Ebola outbreak.
SOURCE: BBC, 2016.

heightened when people from outside the African continent were infected: an American citizen died of Ebola in Nigeria on July 25, shortly after arriving from Liberia; two American aid workers were infected in Liberia in late July and transported back to the United States for treatment; and a Spanish priest died in Madrid in early August after treating patients in Liberia. These cases sparked fears that Ebola could become an international pandemic and spread far beyond the affected region. Yet it was not until August 6 that the WHO director-general convened an Emergency Committee for Ebola under the International Health Regulations (IHR 2005), and it was not until August 8 that WHO declared the Ebola outbreak a public health emergency of international concern (PHEIC) (WHO, 2014e).

International Response

For the past decade, since the adoption of the updated IHR in 2007,[1] there has existed a mechanism to engage the international community in a global public health response. This mechanism resides in the responsibility of the director-general to convene an emergency committee to advise on

[1] The IHR (2005) entered into force, generally, on June 15, 2007, and are currently binding on 194 countries (States Parties) across the globe, including all 193 Member States of WHO (WHO, 2007).

Copyright © National Academy of Sciences. All rights reserved.

the need to declare a PHEIC, the most urgent designation for a potentially pandemic infectious diseases threat. A PHEIC is an event that constitutes a "public health risk through the international spread of disease" and that "potentially require[s] a coordinated international response" (Murthy, 2016). Four public health crises have been designated PHEICs since PHEIC was defined in IHR 2005: swine flu (2009), polio (2014), Ebola (2014), and Zika virus (2016) (CDC, 2016b).

The declaration of a PHEIC made clear the need for a coordinated international response, and the global community gathered to aid the individuals in the affected communities and countries (WHO, 2015c). The international response was multifaceted, including pledges of money from foreign governments to help build containment facilities to care for the infected and to build laboratory capacity for diagnosis, and the mobilization of foreign volunteers, including health care workers and public health outbreak control experts to provide patients with the best possible level of care and to stop the epidemic from spreading further. However, not much happened until mid-September, when the UN Security Council passed Resolution 2177 (UN, 2014), calling for the creation of a UN Mission for Ebola, and governments began to mobilize the logistics for support. Shortly afterwards, at the High-level Meeting on Response to the Ebola Disease Outbreak at the United Nations on September 25, 2014, Joanne Liu, international president of MSF, said, "Generous pledges of aid and unprecedented UN resolutions are very welcome. But they will mean little, unless they are translated into immediate action. The reality on the ground today is this: the promised surge has not yet delivered" (Liu, 2014). The WHO coordinated outbreak response efforts through the Global Outbreak Alert and Response Network, which "deployed a multidisciplinary workforce of 895 experts in the current Ebola outbreak response operation in West Africa, including doctors, nurses, infection control specialists, logisticians, laboratory specialists; communication, anthropology and social mobilization experts, emergency management and public health professionals among others" (WHO, 2016b).

Clinical Trials

Shortly after the declaration of a PHEIC, the WHO began to convene meetings to discuss the use of potential Ebola therapeutics and vaccines that were in various stages of development. ETUs had little to offer patients because treatments such as supportive care with fluids and electrolytes, monitoring blood pressure and kidney function, and medications for associated secondary infections were unavailable, rudimentary, or limited. The lack of targeted antiviral therapeutics meant that ETUs were limited to providing supportive care for patients, and mortality rates were high

Copyright © National Academy of Sciences. All rights reserved.

(CDC, 2015a). Some patients—primarily Westerners—had been treated with unproven Ebola therapeutics[2] and survived, giving hope that a safe and effective therapeutic could be found in time to stem the tide of the epidemic, while rumors circulated in West Africa that there was a magic or secret serum cure that was not being made available for them (Seay, 2014). The WHO convened several meetings during the fall of 2014, during which stakeholders discussed the scientific, ethical, and regulatory issues involved in conducting clinical trials on these therapeutics and vaccines. By December 2014 the first clinical trials began in the region, as the death toll from Ebola neared 8,000 with over 20,000 reported cases (Dunning et al., 2016; WHO, 2014c).

Key players involved in developing and conducting the clinical trials in West Africa included the WHO; research organizations such as the U.S. National Institutes of Health; public health organizations such as CDC and Institut national de la santé et de la recherche médicale[3] (Inserm); academic centers including the University of Oxford, the London School of Hygiene and Tropical Medicine, and the Institute of Tropical Medicine at Antwerp, Belgium; humanitarian groups such as MSF, International Medical Corps, and GOAL International; pharmaceutical companies including GlaxoSmithKline, Merck, and Johnson & Johnson (Janssen Pharmaceuticals); and international funders such as the Wellcome Trust and the Bill & Melinda Gates Foundation (WHO, 2015f). There were many hurdles to overcome, and international research groups and researchers worked in partnership with the ministries of health in Guinea, Liberia, and Sierra Leone to implement their trials.

Conducting the trials took immense effort, from selecting investigational medicinal products to identifying trial sites and setting up appropriate infrastructure to implement trials in the midst of a public health emergency. The success of these groups in launching clinical trials on a compressed time frame, in countries that were unfamiliar with clinical research, and for products that had largely never before been tested in humans, was groundbreaking. However, this success was not without setbacks, which included administrative delays (Lang, 2015) and various disputes regarding the selection of vaccine and therapeutic candidates, trial designs, and other issues. The disparate goals and missions of international partners were displayed when conflict arose over two different perspectives regarding the goals of the clinical trials and how best to design them (Presidential Commission for the Study of Bioethical Issues, 2015). Research organizations

[2] Unproven investigational therapeutics used under expanded access (also known as compassionate use) included ZMapp, brincidoforvir, TKM-Ebola, favipiravir, and convalescent plasma (WHO, 2015a).

[3] France's National Institute for Health and Medical Research.

Copyright © National Academy of Sciences. All rights reserved.

said that the aim of conducting clinical trials should be to identify safe and effective interventions as efficiently and reliably as possible and that randomized, controlled trials were the best way to achieve this goal. Others said that trials should be conducted in order to provide access to the potential benefits of experimental interventions to as many participants as possible. These stakeholders promoted the use of research designs without randomization or concurrent controls. The conflict between these two perspectives became a central point of contention between stakeholders. These protracted arguments hindered the implementation of robust clinical trials during the 2014–2015 epidemic. (The conflict between researchers and its impact on trial implementation is further discussed in Chapter 2.)

ORGANIZATION OF THE REPORT

This report is organized into six chapters, which follow this introductory chapter. Chapter 2, Conducting Clinical Research During an Epidemic, explores the arguments that arose around clinical trial designs, discusses the ethics and moral principles of conducting clinical research during an epidemic, and examines the ethical arguments made during the 2014–2015 Ebola epidemic. Chapter 3, Assessment of Therapeutic Trials, reviews the formal clinical trials on investigational therapeutic agents conducted in West Africa during the Ebola epidemic, specifically looking at the scientific value of the data generated as a result of the trials. Chapter 4, Assessment of Vaccine Trials, similarly assesses the formal Ebola-specific vaccine trials conducted in West Africa during the Ebola epidemic. Chapter 5, Strengthening Capacity for Research and Response, examines the underlying health systems in West Africa and how a lack of clinical and research capacity influenced clinical research and epidemic response, examines logistical considerations that impacted the conduct of trials, and makes recommendations on how to strengthen capacity to be better prepared for the next epidemic. Chapter 6, Engaging Communities in Research and Response, discusses the social and community context that surrounded the Ebola outbreak and how this influenced clinical trials and explores best practices for community engagement in the event of a future public health emergency. Finally, Chapter 7, Facilitating International Coordination and Collaboration, discusses the need for a coalition of international stakeholders to establish a mechanism that will encourage relationship building and participation of the global research and development and epidemic response communities in addressing key concerns prior to the next epidemic.

Copyright © National Academy of Sciences. All rights reserved.

REFERENCES

Baize, S., D. Pannetier, L. Oestereich, T. Rieger, L. Koivogui, N. F. Magassouba, B. Soropogui, M. S. Sow, S. Keïta, H. De Clerck, A. Tiffany, G. Dominguez, M. Loua, A. Traoré, M. Kolié, E. R. Malano, E. Heleze, A. Bocquin, S. Mély, H. Raoul, V. Caro, D. Cadar, M. Gabriel, M. Pahlmann, D. Tappe, J. Schmidt-Chanasit, B. Impouma, A. K. Diallo, P. Formenty, M. Van Herp, and S. Günther. 2014. Emergence of Zaire Ebola virus disease in Guinea. *New England Journal of Medicine* 371(15):1418–1425.

BBC (British Broadcasting Corporation). 2016. *Ebola: Mapping the outbreak*. http://www.bbc.com/news/world-africa-28755033 (accessed January 11, 2017).

Boston Children's Hospital and Harvard Medical School. 2016. *Ebola outbreaks: Map*. http://www.healthmap.org/ebola/#timeline (accessed January 11, 2017).

Burki, T. K. 2011. USA focuses on Ebola vaccine but research gaps remain. *The Lancet* 378(9789):389.

CDC (U.S. Centers for Disease Control and Prevention). 2015a. *Ebola (Ebola virus disease): Treatment*. https://www.cdc.gov/vhf/ebola/treatment (accessed January 11, 2017).

CDC. 2015b. *Q&As on transmission*. https://www.cdc.gov/vhf/ebola/transmission/qas.html (accessed January 11, 2017).

CDC. 2016a. *Ebola (Ebola virus disease): CDC's role*. https://www.cdc.gov/vhf/ebola/outbreaks/2014-west-africa/what-cdc-is-doing.html (accessed January 11, 2017).

CDC. 2016b. *Global health security: International Health Regulations (IHR)*. https://www.cdc.gov/globalhealth/healthprotection/ghs/ihr (accessed January 11, 2017).

CDC. 2016c. *Outbreaks chronology: Ebola virus disease*. https://www.cdc.gov/vhf/ebola/outbreaks/history/chronology.html (accessed December, 2016).

CIA (Central Intelligence Agency). 2016. *The world factbook*. https://www.cia.gov/library/publications/the-world-factbook/wfbExt/region_afr.html (accessed December 20, 2016).

Deloffre, M. Z. 2016. Three lessons from Ebola can help us fight the Zika virus. *Washington Post*, February 14. https://www.washingtonpost.com/news/monkey-cage/wp/2016/02/14/heres-why-zika-and-ebola-are-more-than-public-health-crises/?utm_term=.31c928a82973 (accessed January 11, 2017).

Dunning, J., C. Longuet, and A. Salam. 2016. Ebola research: An encounter between science and humanitarian action. *Alternatives Humanitaires/Humanitarian Alternatives* 1(1):81–93.

Fairhead, J. 2015. Understanding social resistance to Ebola response in Guinea: An anthropological perspective. *African Studies Review* 59(3):7–31.

Gebre, Y., T. Gebre, and A. Peters. 2014. The Ebola virus: A review of progress and development in research. *Asian Pacific Journal of Tropical Biomedicine* 4(12):928–936.

Heymann, D., and L. Wertheimer. 2014. Why this Ebola outbreak is different than earlier versions. In *Weekend edition Sunday: NPR*. http://www.npr.org/2014/08/17/341083551/why-this-ebola-outbreak-is-different-than-earlier-versions (accessed January 11, 2017).

Hofman, M., and S. Au. 2017. *The politics of fear: Médecins Sans Frontières and the West African Ebola epidemic*. New York: Oxford University Press.

International Crisis Group. 2015. *The politics behind the Ebola crisis*. Africa report no. 232. Brussels, Belgium: International Crisis Group. https://d2071andvip0wj.cloudfront.net/232-the-politics-behind-the-ebola-crisis.pdf (accessed January 11, 2017).

Johnson, K. M., J. V. Lange, P. A. Webb, and F. A. Murphy. 1977. Isolation and partial characterisation of a new virus causing acute hæmorrhagic fever in Zaire. *The Lancet* 310(8011):569–570.

KFF (Kaiser Family Foundation). 2014. *Ebola characteristics and comparisons to other infectious diseases*. http://kff.org/infographic/ebola-characteristics-and-comparisons-to-other-infectious-diseases (accessed January 11, 2014).

Copyright © National Academy of Sciences. All rights reserved.

Lane, H. C., H. D. Marston, and A. S. Fauci. 2016. Conducting clinical trials in outbreak settings: Points to consider. *Clinical Trials* 13(1):92–95.

Lang, T. 2015. Ebola: Embed research in outbreak response. *Nature* 524(7563):29–31.

Liu, J. 2014. *MSF president urges UN General Assembly to act now*. http://www.msf.org/en/article/msf-president-urges-un-general-assembly-act-now (accessed January 11, 2017).

Lurie, N., T. Manolio, A. P. Patterson, F. Collins, and T. Frieden. 2013. Research as a part of public health emergency response. *New England Journal of Medicine* 368(13):1251–1255.

Manguvo, A., and B. Mafuvadze. 2015. The impact of traditional and religious practices on the spread of Ebola in West Africa: Time for a strategic shift. *Pan African Medical Journal* 22(Suppl 1):9.

McCoy, T. 2014. Why the brutal murder of several Ebola workers may hint at more violence to come. *Washington Post*, September 19. https://www.washingtonpost.com/news/morning-mix/wp/2014/09/19/why-the-brutal-murder-of-eight-ebola-workers-may-hint-at-more-violence-to-come/?utm_term=.7b84f596959b (accessed January 11, 2017).

MSF (Médecins Sans Frontières). 2014a. *Ebola in West Africa: Epidemic requires massive deployment of resources*. http://www.msf.org/article/ebola-west-africa-epidemic-requires-massive-deployment-resources (accessed January 11, 2017).

MSF. 2014b. *Guinea: Mobilisation against an unprecedented Ebola epidemic*. http://www.msf.org/en/article/guinea-mobilisation-against-unprecedented-ebola-epidemic (accessed December 20, 2016).

MSF. 2015. *Ebola: Pushed to the limit and beyond: A year into the largest ever Ebola outbreak*. Geneva, Switzerland: Médecins Sans Frontières. http://www.msf.org/sites/msf.org/files/msf1yearebolareport_en_230315.pdf (accessed January 11, 2017).

Murthy, N. 2016. *Public health experts urge World Health Organization to ramp up Zika response*. http://news.medill.northwestern.edu/chicago/public-health-experts-urge-world-health-organization-to-ramp-up-zika-response/ (accessed January 11, 2017).

Pellecchia, U., R. Crestani, T. Decroo, R. Van den Bergh, and Y. Al-Kourdi. 2015. Social consequences of Ebola containment measures in Liberia. *PLoS ONE* 10(12):e0143036.

Presidential Commission for the Study of Bioethical Issues. 2015. *Ethics and Ebola: Public health planning and response*. Washington, DC: Presidential Commission for the Study of Bioethical Issues. http://bioethics.gov/sites/default/files/Ethics-and-Ebola_PCSBI_508.pdf (accessed January 18, 2017).

RAS (Royal African Society). 2015. *Ebola: An epidemic of mistrust*. http://www.royalafricansociety.org/analysis/ebola-epidemic-mistrust (accessed December 20, 2016).

Samb, S. 2014. *WHO says Guinea Ebola outbreak small as MSF slams international response*. http://www.reuters.com/article/us-guinea-ebola-idUSBREA301X120140401 (accessed December 20, 2016).

Seay, L. 2014. Ebola, research ethics, and the ZMapp serum. *Washington Post*, August 6. https://www.washingtonpost.com/news/monkey-cage/wp/2014/08/06/ebola-research-ethics-and-the-zmapp-serum/?utm_term=.826de62c2cd9 (accessed January 11, 2017).

Taddonio, P. 2015. Outbreak: Inside the troubled early days of Guinea's Ebola response. *PBS Frontline*, May 5. http://www.pbs.org/wgbh/frontline/article/watch-inside-the-troubled-early-days-of-guineas-ebola-response (accessed January 11, 2017).

UN (United Nations). 2014. *With spread of Ebola outpacing response, Security Council adopts resolution 2177 (2014) urging immediate action, end to isolation of affected states*. https://www.un.org/press/en/2014/sc11566.doc.htm (accessed January 11, 2017).

UNDP (United Nations Development Programme). 2014. *Human development report 2014—Sustaining human progress: Reducing vulnerabilities and building resilience*. New York: UNDP. http://hdr.undp.org/sites/default/files/hdr14-report-en-1.pdf (accessed January 11, 2017).

Copyright © National Academy of Sciences. All rights reserved.

Integrating Clinical Research into Epidemic Response: The Ebola Experience

Wainberg, M. A., S. Kippax, M. Bras, and P. S. Sow. 2014. HIV and Ebola: Similarities and differences. *Journal of the International AIDS Society* 17(1):19896.

WHO (World Health Organization). 2007. *International Health Regulations (2005): Areas of work for implementation.* http://www.who.int/ihr/finalversion9Nov07.pdf (accessed February 16, 2017).

WHO. 2013. *Emergency response framework (ERF).* Geneva, Switzerland: WHO Press. http://www.who.int/hac/about/erf_.pdf (accessed January 11, 2017).

WHO. 2014a. *Ebola challenges West African countries as WHO ramps up response*. http://www.who.int/mediacentre/news/notes/2014/ebola-response/en (accessed December 20, 2016).

WHO. 2014b. *Ebola response roadmap: Situation report—31 December 2014*. Geneva, Switzerland: WHO Press. http://apps.who.int/ebola/en/status-outbreak/situation-reports/ebola-situation-report-31-december-2014 (accessed January 11, 2017).

WHO. 2014c. *Ebola virus disease in Guinea*. http://www.who.int/csr/don/2014_03_23_ebola/en (accessed December 20, 2016).

WHO. 2014d. *Ebola virus disease, West Africa—Update*. http://www.who.int/csr/don/2014_07_01_ebola/en (accessed December 20, 2016).

WHO. 2014e. *Statement on the 1st meeting of the IHR Emergency Committee on the 2014 Ebola outbreak in West Africa*. http://www.who.int/mediacentre/news/statements/2014/ebola-20140808/en (accessed December 20, 2016).

WHO. 2015a. *Compassionate use of experimental treatments for Ebola virus disease: Outcomes in 14 patients admitted from August to November, 2014*. http://www.who.int/medicines/ebola-treatment/outcomes_experimental_therapies/en (accessed February 17, 2017).

WHO. 2015b. *Guinea: The Ebola virus shows its tenacity*. http://www.who.int/csr/disease/ebola/one-year-report/guinea/en (accessed December 20, 2016).

WHO. 2015c. *Key events in the WHO response to the Ebola outbreak*. http://www.who.int/csr/disease/ebola/one-year-report/who-response/en (accessed January 11, 2017).

WHO. 2015d. *Origins of the 2014 Ebola epidemic*. http://www.who.int/csr/disease/ebola/one-year-report/virus-origin/en (accessed December 20, 2016).

WHO. 2015e. *Successful Ebola responses in Nigeria, Senegal and Mali*. http://www.who.int/csr/disease/ebola/one-year-report/nigeria/en (accessed January 11, 2017).

WHO. 2015f. *WHO Ebola R&D effort—Vaccines, therapies, diagnostics*. http://www.who.int/medicines/ebola-treatment/ebola_r_d_effort/en (accessed December 20, 2016).

WHO. 2016a. *Ebola virus disease—Fact sheet*. http://www.who.int/mediacentre/factsheets/fs103/en (accessed December 21, 2016).

WHO. 2016b. *Partners: Global Outbreak Alert and Response Network (GOARN)*. http://www.who.int/csr/disease/ebola/partners/en (accessed December 20, 2016).

WHO. 2016c. *Sierra Leone: A traditional healer and a funeral*. http://www.who.int/csr/disease/ebola/ebola-6-months/sierra-leone/en (accessed January 11, 2017).

Williams, C. L. 2015. Leading the charge: Médecins Sans Frontières receives the 2015 Lasker~Bloomberg Public Service Award. *Journal of Clinical Investigation* 125(10):3737–3741.

WMA (World Medical Association). 2013. World Medical Association Declaration of Helsinki: Ethical principles for medical research involving human subjects. *JAMA* 310(20):2191–2194.

Copyright © National Academy of Sciences. All rights reserved.

Copyright © National Academy of Sciences. All rights reserved.

2

Conducting Clinical Research During an Epidemic

With the Ebola epidemic rapidly spiraling upward in the summer of 2014 and the international community searching for a way to quell the tide, attention turned to the possibility of using experimental medicinal products to treat Ebola patients. There were no therapeutic treatments or vaccines for Ebola that were proven safe or effective, and the Ebola-specific agents that were furthest along in development had only reached the stage of preclinical studies in nonhuman primates. The lack of agents with demonstrated efficacy meant that there were no treatment options outside of supportive care for Ebola, and even supportive care was frequently difficult to obtain, particularly at the beginning of the outbreak. The available Ebola treatment units (ETUs) were filled to capacity and beyond, and health care workers had little to offer patients beyond a "bed, three meals, [oral] fluids, tablets, anti-malarials, [and] painkillers"—and sometimes even these were unavailable (MSF, 2015). More advanced supportive care, such as monitoring electrolytes and blood chemistry or respiratory and renal support, was often not possible, as it required nonexistent equipment and technical and laboratory support. Little information was available about the factors that allowed some patients to recover and others to succumb to the illness; mortality was high (MSF, 2015). As one frontline doctor with Médecins Sans Frontières (MSF) in Sierra Leone, Javid Abdelmoneim, stated, "I can only say you have around 50 percent chance of dying, and I can do very little about it for you" (MSF, 2015, p. 17).

Initially the World Health Organization (WHO) and some nongovernmental organizations providing care on the ground were opposed to using untested medical products due to the level of mistrust, conspiracy allega-

Copyright © National Academy of Sciences. All rights reserved.

Integrating Clinical Research into Epidemic Response: The Ebola Experience

tions, and violence toward international health workers (McCoy, 2014). As a representative from MSF noted in *Science*, "There are rumors that we are spreading disease, harvesting organs, and other horrible things. Bringing in unlicensed things to experiment on people could be very counterproductive" (Enserink, 2014a, p. 364). However, in July 2014 two infected American aid workers—Kent Brantly and Nancy Writebol—were treated with an experimental agent and the perceptions of the international community and the responders in-country changed (Enserink, 2014b). Brantly and Writebol received doses of the experimental agent ZMapp, an engineered monoclonal antibody cocktail that had been shown to be effective in rhesus macaque monkeys but had not previously been administered to humans (Qiu et al., 2014). ZMapp was shipped to Monrovia for the aid workers before they were separately evacuated for further management at Emory University Hospital in Atlanta, Georgia (Seay, 2014). Although critically ill with Ebola, both Brantly and Writebol recovered and were Ebola-free when discharged in late August 2014. Their recovery brought global attention to investigational agents, and ZMapp was soon "dubbed 'secret or magic serum' by the media," and "generated hope, suspicion, accusations of inequity, and requests for additional product" (Goodman, 2014, p. 1086). Foreigners who had been infected with Ebola were treated with other experimental therapies in addition to ZMapp, including convalescent plasma, convalescent whole blood, and the experimental antiviral drug TKM-Ebola. Despite the global publicity, however, it was unknown what effect, if any, the untested products had on the patients' recovery. As Bruce Ribner, the lead physician at Emory University Hospital, where the patients were treated, and the director of Emory's infectious disease unit, said, "Frankly we do not know if [ZMapp] helped them, made any difference, or even delayed their recovery" (Moisse, 2014).

Some of the perceptions around the effectiveness of investigational agents were influenced by the disparate clinical care international workers received. Foreigners infected with Ebola were evacuated from the region to the United States or Europe and were provided state-of-the-art supportive care. In fact, 22 out of 27 patients treated in the United States or Europe between August 2014 and December 2015 survived—a case-fatality rate far lower than in West Africa at the beginning of the epidemic (McWhirter et al., 2014; Uyeki et al., 2016). Even in the face of minimal evidence that these experimental therapies were safe or effective, the media and public focused their attention on the untested products rather than on the role of supportive care, and there were urgent calls to make the products more widely available (Singh, 2015; Wahl, 2014). Shortly after Brantly and Writebol received ZMapp, African health authorities questioned why two Americans had received the drug while no treatment was made available for the thousands of Africans infected with Ebola. The Liberian assistant

Copyright © National Academy of Sciences. All rights reserved.

health minister, Tolbert Nyenswah, said, "This is something that has made our job most difficult. The population here is asking: 'You said there was no cure for Ebola, but the Americans are curing it?'" (McWhirter et al., 2014).

EARLY DEBATES ABOUT USE OF PRODUCTS

With global attention focused on experimental therapies for Ebola—and calls to make them more widely available—it forced the question of how these agents could best be utilized in the fight against Ebola. Since early mortality rates were high and the agents offered at least the possibility of benefit some argued that experimental therapies should be given to as many patients as possible. Others argued that because so little was known about the agents, it was necessary to conduct formal clinical trials in order to quickly and efficiently identify beneficial therapies or vaccines. This tension—between those responding to the massive humanitarian crisis who desired medicinal products to treat individuals, and those who supported the use of medicinal interventions only after products had been evaluated for safety and efficacy in clinical trials—complicated the early discussions about the appropriate international response to the epidemic. Frontline humanitarian agencies, such as MSF, were overwhelmed with carrying out basic patient care and public health measures, and they considered the international community's initial response to be dangerously inadequate to meet the needs of the affected communities (MSF, 2014a). Due to the time and resource constraints of taking care of Ebola patients, some frontline providers appeared convinced that it was impossible to both provide effective clinical care and conduct useful clinical research.

Research Versus Care

This tension between research and care is ever present in public health emergencies. The urgent desire to help current patients with whatever is available may appear to be in conflict with the need to learn as much as possible about potential interventions in order to help current and future patients. During the Ebola epidemic, some caregivers may have felt that providing clinical care and conducting clinical research were mutually exclusive and that one could not be done without harming the other effort. Clinical research and clinical care are sometimes at odds because care focuses on the individual, current needs of a specific patient (Sacristán, 2015), while clinical research benefits future patients and not necessarily the specific patient enrolled in the research; however, patients who enroll in clinical trials often benefit from receiving better medical care than patients not enrolled in trials. Furthermore, in a research setting health care decisions are not based only on the interaction of one health professional and

Copyright © National Academy of Sciences. All rights reserved.

one patient, but are often controlled through the process of randomization, and adherence to a standard protocol is required. This lack of autonomy on the part of both clinician and patient can add to the tension.

Despite tensions between research and clinical care, they can also be seen as two sides of the same coin, ideally conducted in tandem (Sacristán, 2015). The Declaration of Helsinki addresses this in its guidelines for physicians; it states that research and medical care may be combined "only to the extent that the research is justified by its potential preventive, diagnostic, or therapeutic value and if the physician has good reason to believe that participation in the research study will not adversely affect the health of the patients who serve as research subjects" (WMA, 2013). In the initial stages of an outbreak or epidemic, when care centers and workers are inundated with patients, it may be appropriate to focus the limited resources available on clinical care so as not to detract from the clinical response and incorporate research later when staff is not spread as thin. However, it is critical that the possibility of integrating research into clinical care is part of the discussions and planning from the outset, because it can take considerable time to obtain necessary approvals for research and to fully train the study team, while the opportunity to initiate a trial is continually reassessed as conditions on the ground change. If the planning does not occur early in the epidemic, the epidemic may be waning—or over—by the time a study is designed, approved, and ready to be implemented. This aptly describes the Ebola outbreak in the first 6 months of 2014.

An initial question was whether it was ethical to conduct research at all, given the extreme demands already placed on frontline care providers during the epidemic. Because there were no drugs or vaccines approved to treat or prevent Ebola or ready to enter into clinical trials at the outset of the epidemic, many felt that there was an ethical imperative to conduct such research as quickly and safely as possible. Providers needed to learn how best to treat patients or prevent new Ebola infections and to assess how health systems could be configured and equipped to meet these health needs. The WHO Ethics Working Group report from October 2014 stated that there was an ethical obligation to do research during the epidemic and that research should be part of the public health response (WHO, 2014b). Only by increasing the knowledge base about Ebola and about the merits of various treatment or prevention strategies could clinicians be sure that their efforts improved patient outcomes and communities be reassured that their scarce resources were used wisely and efficiently (UNESCO, 2006).

Expanded Access

Although providing experimental therapies in the context of a clinical trial is the ideal way to monitor and minimize risks of unproven agents

Copyright © National Academy of Sciences. All rights reserved.

Integrating Clinical Research into Epidemic Response: The Ebola Experience

while maximizing the scientific information gained, in some circumstances it is appropriate to administer unproven agents outside of an approved clinical trial. This is called an expanded access exemption or "compassionate use." In the United States, a number of conditions must be met in order for a patient to be granted access to a drug under expanded access: (1) there is no comparable or satisfactory therapy available, (2) the probable risk from the investigational product is not greater than the probable risk from the disease, and (3) providing the investigational product will not interfere with the conduct of clinical trials (FDA, 2016).

The foreign aid workers, like Brantley and Writebol, received experimental therapies under an expanded access framework. In October 2014, the WHO working group report referred to compassionate use of investigational products as justifiable as long as data are collected and shared (WHO, 2015). However, most of the examples of expanded access provided were cases of foreign health workers who were evacuated from West Africa to the United States or Europe in order to ensure that they received optimal supportive care, and who, in desperation, were also offered whatever experimental intervention was available—and more than one if available (Enserink, 2014b). In this context, it would have been extremely difficult to attribute either beneficial or detrimental outcomes to any one of these investigational agents. The use of investigational agents under expanded access in these situations did not contribute to the knowledge base, but they did serve to initiate rumors that there was a cure for the foreigners that was not being made available to Africans. The belief that investigational agents in the very early stages of development were likely to be highly effective furthered the view that randomized controlled trials were unethical. For example, Caplan et al. concluded that because "all available agents have been variously deployed against infected persons treated in the United States and Europe, the case for randomization to placebo in West Africa is morally suspect" (Caplan et al., 2015, p. 6).

The assertion of a right to access an intervention without established efficacy in these circumstances is controversial at best. Any such right must be grounded in a concern for individual health, and there is no evidence that investigational products in the early stage of development will promote the health of humans treated with the agent. In fact, most agents in the early stages of development are eventually proven to be ineffective or even potentially harmful (Dresser, 2009). Additionally, a right to access interventions approved for treating another condition, but without established efficacy for another particular condition might be unbounded in its scope since there are potentially a great many interventions that have no evidence of efficacy for a particular condition but whose use might be supported on theoretical or even speculative grounds. Moreover, rights such as this do not exist in a vacuum. They can only be honored through the expenditure

Copyright © National Academy of Sciences. All rights reserved.

of time and resources for research. When limited resources have to be used to address the health needs of many individuals, proof of efficacy is a reasonable requirement for the use of resources. Absence of evidence of efficacy thus reduces the strength of the claim that scarce resources should be directed to the provision of novel interventions of unknown value.

Others argued that expanded access should be avoided because its "use exposes many patients to investigational interventions, often undermines fair access to experimental agents, compromises the collection of robust data to determine the safety and efficacy of interventions, and consumes scarce resources for uncertain clinical benefits" (Rid and Emanuel, 2014, p. 1844). Additionally, given the limited supply of experimental Ebola treatments and vaccines at the time, randomized trials may actually have been the most equitable way to distribute these products (Largent, 2016; Rettner, 2014). The strongest argument for providing expanded access to unproven therapies during the Ebola epidemic is that the high lethality of the disease tipped the ethical scales in favor of providing interventions that could be helpful, however remote that prospect of benefit may have been and even given the potential for harm. This argument springs from the principle of beneficence—the notion that medical care providers should seek to help patients. Yet even under such conditions, the social costs of providing expanded access merit consideration. Specifically, under circumstances like the Ebola epidemic, the principle of beneficence supports providing products under an expanded access exception when the following conditions are met (Darrow et al., 2015; FDA, 2016):

- A sufficient amount of the product is available after supplying the needs of clinical trials.
- Providing expanded access would not preclude or delay the initiation of more conclusive investigations of the intervention in properly designed studies. This could occur, for example, if the availability of investigational products off protocol depleted the supply of individuals willing to enroll in studies that could yield generalizable knowledge about the product's safety and efficacy.
- Existing evidence does not suggest such an unfavorable risk–benefit balance that the product would not even "make the cut" for inclusion in clinical trials.

Conclusion 2-1 *The use of unproven experimental therapies—especially those in the early phases of drug development—under an expanded access exemption to patients regardless of nationality or where they are located, not only fails to provide information on safety or efficacy, but also creates inequities with the larger affected population during an epidemic. Such uses can promote the public misconcep-*

Copyright © National Academy of Sciences. All rights reserved.

tion that a safe and effective treatment exists and may generate mistrust of researchers and research efforts that will make it more difficult to launch clinical trials when additional interventions become available.

PLANNING CLINICAL TRIALS

Given the urgency of the situation, the August 2014 WHO ethics panel concluded that there was an "ethical imperative to offer the available experimental interventions that have shown promising results," noting that the "only way of obtaining evidence on the safety and efficacy of any intervention in Ebola virus disease is during an outbreak" (WHO, 2014a, p. 4). The panel stated that compassionate use is "justified as an exceptional emergency measure" but said that it should not "preclude or delay the initiation of more conclusive investigations of the intervention(s) in properly designed clinical studies" (WHO, 2014a, pp. 5–6). The panel identified a number of conditions for the use of investigational interventions (WHO, 2014a):

- The investigations should not divert attention or resources from public health measures.
- Ethical criteria should guide the use of such interventions.
- The use of the interventions should be based on the best possible assessment of risk and benefit.
- The interventions should have been demonstrated to be safe and effective in animal models, in particular in nonhuman primates.
- Expanded access for individual use should be employed only with a shared understanding of the criteria for such exceptions, and it should not preclude or delay high-quality clinical investigations.
- The uncertainty about the safety and efficacy of the interventions should be acknowledged and communicated to all stakeholders to avoid unfounded expectations.
- Investigational therapies should be administered in concert with necessary supportive treatment, management of side effects, and monitoring the progress of treatment.
- The data generated from the use of investigational therapies should be systematically collected and shared.
- The decision to use investigational therapies should take into consideration the available standard of care and feasibility in the setting.

Not all stakeholders were in agreement with the WHO's conclusions or the focus on clinical research. Criticism was aimed at the makeup of the August WHO ethics advisory panel, as only a few of the panelists

Copyright © National Academy of Sciences. All rights reserved.

had a background in bioethics or medical ethics, women were underrepresented, and the panel included no representatives from the countries actually affected by Ebola (Schuklenk, 2014). Some public health advocates questioned the choice to focus on the treatment of individuals rather than on broad public health measures; other critics argued that the WHO's individualistic medical ethics approach "frames the issues incorrectly, imposes the wrong priorities, and uses the wrong set of values" (Dawson, 2015, p. 107). A medical advisor at Queensland Health in Australia worried that the "inappropriate focus on experimental treatments for individuals diverted attention away from infection control and other measures that would benefit everyone," and asserted that "thousands died while we argued over the wrong questions" (Gericke, 2015).

Despite these criticisms, attention soon turned toward planning clinical trials in order to identify safe and effective therapeutic(s) or vaccine(s). Discussions—and sometimes heated debates—ensued about which candidates should be tested and how the trials should be designed. These discussions were heavily influenced by stakeholders' perspectives and experiences with the early days of the epidemic. The overwhelming numbers of desperately ill patients, combined with the limited number of caregivers and the resource constraints they faced, likely contributed to the perception among frontline personnel that resource-intensive research designs would not be feasible or else would compromise patient care and therefore would be inherently unethical. Similarly, the belief that the fatality rate was very high and that the outbreak was out of control, supported by a Centers for Diseases Control and Prevention (CDC) projection that the numbers could reach over a million people in a few months (Meltzer et al., 2014), likely influenced the way stakeholders framed the response strategy to focus on expanding numbers of treatment beds rather than conducting ongoing research and evaluation. Pertaining to clinical trials in particular, this belief created a context in which some stakeholders prioritized research strategies designed to detect only highly efficacious medicinal products (i.e., a magic bullet) that could potentially be used during the epidemic at hand, while others believed that looking for something even moderately effective was equally worthy of research. Rather than considering both approaches as complementary, the desire to quickly identify an intervention that might be a game changer led some stakeholders to deprioritize efforts to conduct studies that might make real but incremental improvements to the understanding and treatment of Ebola (Branswell, 2014).

In order to implement clinical trials, researchers and stakeholders needed to answer two initial questions: Which potential therapeutic agents or vaccines should be tested? And how should the clinical trials be designed?

Copyright © National Academy of Sciences. All rights reserved.

Identifying Candidates to Research

To identify candidates for trials, researchers looked to the few Ebola-specific agents that were in early stages of development, and also explored the possibility of repurposing approved drugs for the new indication of Ebola. Although already approved for another indication, repurposed drugs would still require clinical investigation in order to determine the efficacy and safety of the drug for the new indication and patient population. Researchers sought to investigate numerous agents with limited evidence of potential value in the search for a highly efficacious medicinal product to treat Ebola. This pursuit resulted in a glut of proposals that "flooded the in-boxes of staff at the WHO and research funding agencies. Silver nanoparticles. Cholesterol-controlling statins. A breast cancer drug. Intravenous ozone. Vulture gastric fluids. An influenza antiviral. Interferon. Almost anything you can think of [was] being advocated as a potential Ebola curative, often with few or no data to support the case" (Branswell, 2014). In order to prioritize and select compounds to study in clinical trials for the treatment of Ebola, the WHO convened the Scientific and Technical Advisory Committee on Emergency Ebola Interventions (STAC-EE) in November 2014 in Geneva. Here the committee noted their inundation with an ever-increasing number of potential agents for proposed trials: "WHO and partners receive daily proposals for potential products against [Ebola] from the scientific community" (WHO, 2014d).

In order to winnow these down, the STAC-EE committee developed a set of criteria regarding the minimum levels of evidence required for an agent to be considered for clinical trials. The summary of this meeting makes clear that there was disagreement about the relative importance of the availability of an agent and the efficacy of that agent. Some participants believed that products that were readily available or easy to produce, such as brincidofovir and favipiravir, should be prioritized despite a lack of evidence that they would be effective in Ebola patients. Others thought that "availability was not a reason to study drugs with weak supporting data" and favored prioritizing drugs that had shown strong preclinical evidence of efficacy, such as monoclonal antibodies and small inhibitory RNA, even if they were less readily available (WHO, 2014d). Ultimately, the STAC-EE committee published a list of around 20 potential agents, acknowledging that "many of these have already been tested and shown to have no activity against the virus" (WHO, 2014d). Participants said that scientists and developers should "assess themselves whether further investigation is warranted" (WHO, 2014d); others later said that the list was not helpful, as it showed products "that barely worked in a mouse . . . in the same column as something that was shown in a non-human primate to be very

Copyright © National Academy of Sciences. All rights reserved.

effective."[1] This lack of prioritization of the agents to be tested may have reduced the likelihood that clinical trials would identify beneficial agents. If fewer agents were proposed it is possible that more comprehensive data would have been available on the more promising agents.

It is difficult to estimate how long an emergent epidemic will last or how many people will be affected, and therefore difficult to determine the number of people who could enroll in a trial. By limiting the number of agents studied and the number of trials allowed to proceed, the trials that do proceed will be more likely to enroll enough participants to reach conclusive results, and the likelihood of identifying effective interventions will be maximized. In order to achieve this prioritization and limiting of trials, a rapid response body that offers access to broad expertise and mechanisms to avoid conflicts of interest in decision making is needed. Such a body should have the ability to convene the expert members at short notice, and have the authority to determine which studies will actually proceed (see Chapter 7 for further discussion).

> ***Conclusion 2-2*** *In the event of a rapidly progressing outbreak it is critical to create a mechanism to prioritize investigational agents for study and limit the conduct of the clinical trials to a small number of products, focusing on those with the most promising preclinical or human clinical data, in order to maximize the likelihood that meaningful results will be generated.*

Choosing Appropriate Trial Designs

Trial design was one of the most contentious areas of debate among those participating in discussions about Ebola clinical trials. Stakeholders disagreed about the proper approach to ethical, scientific, and practical issues, and they disagreed about how these issues should inform design decisions. (A synopsis of some study designs is presented in a table in Appendix B.) The clash between humanitarian medicine and research science was also evident in these discussions. As one representative present at the meetings later offered, "The fundamental tension is between the obligation to treat patients with whatever intervention offers the best hope of success and the obligation to gather objective evidence in a scientifically rigorous manner. The stakes are high in a crisis in which time is short and consequences of treatment failure are deadly" (Dawson, 2015, p. 45). Stakeholders struggled with issues such as using randomized trials versus nonrandomized alternative designs, the use of a standard-of-care control

[1] Testimony of Anthony Fauci, Director, National Institute of Allergy and Infectious Diseases, National Institutes of Health. Committee Meeting #1, Washington, DC, February 2016.

Copyright © National Academy of Sciences. All rights reserved.

arm, and the fair distribution of limited product. Though stakeholders disagreed on all these topics, they later were in agreement on one issue: Too much time was spent debating trial design, rather than quickly implementing trials and discovering safe and effective products in time to fight the epidemic[2] (Cohen and Kupferschmidt, 2014).

Randomized Controlled Trials

The gold standard for a clinical trial continues to be the randomized controlled trial (RCT). An RCT is considered the best tool for assessing the efficacy of a treatment and is used for several reasons: it avoids selection bias, improves comparability between the experimental and control arms, and allows for valid statistical testing that permits a reliable assessment of the likelihood that observed differences in outcomes between arms could be due to chance (Suresh, 2011). In an RCT, patients are allocated by chance to an arm of the study. There are several types of arms, including (NIH, 2016)

- Experimental: A group of participants who receive the intervention that is the focus of the study, that is, the investigational treatment or vaccine.
- Control: A group of participants who do not receive the investigational treatment or vaccine.
    - Active: A group of participants who receive an intervention that is considered to be effective but that is not the investigational treatment (e.g., a vaccine for hepatitis rather than the product under investigation).
    - Placebo: A substance that does not contain active ingredients and is made to be physically indistinguishable from (i.e., it looks and tastes identical to) the investigational treatment or vaccine.
    - Sham: A procedure or device that is made to be indistinguishable from the actual procedure or device being studied but that does not contain active processes or components.
- Standard of care: A group of participants who receive standard medical care for the condition being studied. Standard of care is sometimes but not always provided in conjunction with the experimental treatment, a placebo, or sham.

[2] Testimony of Edward Cox, director, Office of Microbial Products, U.S. Food and Drug Administration. Public Webinar with International Regulators of the Committee on Clinical Trials During the 2014–2015 Ebola Outbreak, WebEx, May 2016.

Copyright © National Academy of Sciences. All rights reserved.

While most trials employ randomization through the comparison of two treatment approaches (e.g., the investigational therapy versus a control), others may compare multiple approaches, with or without a control group. For example, a factorial design randomizes each individual to two or more treatments over the course of a single trial so that multiple questions can be addressed in a single trial (Montgomery et al., 2003). When two or more known effective treatments are available, a head-to-head comparison of treatments (without a control) can be an appropriate design to compare their relative effectiveness and safety. Multiarm, multistage designs and Bayesian adaptive platform designs have also been proposed for situations in which multiple experimental agents are simultaneously available (Gurrin et al., 2000; MRC CTU, 2014). "Adaptive clinical trials are designed to take advantage of accumulating information, by allowing modification to key trial parameters in response to accumulating information and according to predefined rules" (Lewis, 2012, screen 5). (See Box 2-1 for more information on adaptive trial design.) Each of these trial designs involves randomization. There are many procedures for the random assignment of participants to treatment groups in clinical trials. Simple randomization (i.e., the investigational therapy versus a control) is just one form of randomization—some others include block, stratified, and covariate

BOX 2-1
Adaptive Trial Design

The use of adaptive clinical trial design methodology, particularly adaptive randomization permitting changes in the randomization ratio, has been advocated to cut the time and cost of clinical trials in drug development. While some types of adaptive designs may provide greater flexibility and efficiency in some circumstances, there can be operational challenges with their implementation including preplanning protocol deviations based on prior information and the need for extensive and continuous mathematical modeling (Gupta, 2012; Mahajan and Gupta, 2010). Further, adaptive designs can be less efficient than standard sequential designs that allow for early termination.

Two principal issues that must be addressed in adaptive trial designs methods are

1. "whether the adaptation process has led to design, analysis, or conduct flaws that have introduced bias that increases the chance of a false conclusion that the treatment is effective (a Type I error)," and
2. "whether the adaptation process has led to positive study results that are difficult to interpret, irrespective of having control of Type I error" (Chang and Chow, 2011; FDA, 2010).

Copyright © National Academy of Sciences. All rights reserved.

adaptive randomization—each type has its advantages and disadvantages for a given situation (large versus small trials, known prognostic factors, etc.). Clinical trial teams will need to assess the context surrounding the trial before determining which type of randomization to use; however, the benefits of randomization (as discussed above) contribute to it being an essential part of clinical trials to establish efficacy.

It should also be noted that the analysis of trial results depends in part on the design chosen for the trial. Because patient enrollment for a clinical trial is typically staggered, the regular interim analysis of trial results allows rapid identification of highly effective (or harmful) treatments, enabling researchers to terminate a study early if a treatment appears particularly beneficial (or harmful). Several statistical approaches to interim monitoring, constructed to avoid increasing the false positive rate, are widely used, as discussed in Proschan et al. (2006) (see Box 2-2 for additional information on statistical guidelines for early termination of a trial).

Alternatives to Randomization

During discussions about trial design for Ebola research, a wide variety of arguments were voiced in favor of and opposed to RCTs, with stakeholders concerned about scientific validity, safety of participants, the feasibility of conducting RCTs in the context of an epidemic, and ethical issues. Proponents of RCTs said that this design was the "most efficient and reliable way to evaluate the safety and effectiveness of candidate products" (Cox et al., 2014, p. 2350). Proponents also argued that conducting a trial without a randomized concurrent control group would be unethical because it would not be possible to determine the efficacy of the investigational treatment, with one scientist stating that such a trial "would be scientifically invalid, and a scientifically invalid study by definition cannot be ethical" (Davis, 2015). In addition, proponents of RCTs maintained that it would be unethical to give patients an unproven, potentially unsafe medication outside the controlled environment of an RCT (Dunning et al., 2016b). Clifford Lane, deputy director for clinical research and special projects at the U.S. National Institute of Allergy and Infectious Diseases, reinforced this viewpoint, saying, "The idea that there's no need for randomized, controlled trials presupposes that the drugs have zero side effects, that they are efficacious, and that there's no substantial variability from patient to patient. I don't think any of that is true" (Hayden, 2014, p. 178).

Some of the arguments against randomized trials were based on ideas about how the affected communities would perceive randomization or on the logistics of carrying out such a trial. Peter Horby, an epidemiologist at the University of Oxford, reasoned that "[t]hese trials will be conducted in a context of fear, distrust, a lack of effective care options, the admission

Copyright © National Academy of Sciences. All rights reserved.

Integrating Clinical Research into Epidemic Response: The Ebola Experience

BOX 2-2
Statistical Guidelines for Early Termination of Clinical Trials

It is widely recognized that the review of accumulating data from a clinical trial on a regular basis, with the intent of stopping the trial as soon as the comparison of outcomes in the treatment and control groups becomes statistically significant at the usual 0.05 level, will inflate the false positive rate. Since in many trials it would be unethical to refrain from monitoring the accumulating data, statistical methods have been developed to allow such monitoring and the possible early termination based on interim efficacy or safety findings, while maintaining the false positive rate at the desired low level. Some of the widely used tests include

- *O'Brien-Fleming*: Boundaries to guide early termination decisions are calculated to ensure the protection of the false positive rate by severely limiting the possibility of early termination when only a small proportion of the information has become available, but becoming less stringent as more information is accumulated, allowing the final test to be performed at close to the nominal level (often 0.05 or 0.025);
- *Pocock*: Pocock boundaries are constant across the duration of the trial. Thus, Pocock boundaries are less stringent than the O'Brien-Fleming boundaries at early stages in the trial, but the final test will be at a more stringent level; and
- *Haybittle-Peto*: Haybittle and Peto independently proposed a very simple monitoring boundary: test at the same very stringent level throughout the trial (e.g., 0.001 or 0.0001), so that early termination would be permitted with only very extreme interim results, even when the trial is close to completion. With this approach, the inflation of the false positive error is minimal, even when the interim data are reviewed multiple times during the trial, so that the final analysis can still be done at or very close to the nominal level.

of multiple family members to the same center, and sometimes violence against health-care workers. Scientific arguments cannot tell us what will work in these conditions" (Hayden, 2014, p. 178). Further, the objectors noted that the controlled conditions of a randomized trial may not have been logistically possible, given the state of the health care systems in the affected countries (Adebamowo et al., 2014). MSF was clear in its belief that randomization "might not be feasible for therapeutic trials in the context of a very deadly disease with no other therapeutic options."[3] At an October 2014 meeting, the WHO Ethics Working Group heard from

[3] Personal communication, Annick Antierens, Médecins Sans Frontières, March 25, 2015. Trial designs in epidemic emergencies: The perspective of caretakers and aid workers, based on the experience in the 2014–2015 Ebola outbreak.

Copyright © National Academy of Sciences. All rights reserved.

participants from Guinea and Liberia that in their view, communities would not accept a randomized controlled trial because it would "deny a new experimental treatment to some participants" (WHO, 2014b). Proponents of RCTs acknowledged that carrying out controlled trials in the region would be challenging but said they believed that RCTs would be acceptable to the community if public health leaders were "to articulate the rationale for conducting scientifically valid trials, to work closely with local health authorities, and to engage community leaders" (Cox et al., 2014, p. 2351).

Given the hesitations of some stakeholders about conducting RCTs, many alternative trial designs to avoid randomization were proposed at the WHO ethics advisory panel meeting on August 11, 2014. Some argued that those who pushed for RCTs were "doggedly insisting on gold standards that were developed for different settings and purposes" (Adebamowo et al., 2014, p. 1424). Participants at the meeting noted that further discussions were needed in order to determine "the trial designs that are the most appropriate for accommodating the current constraints of the international outbreak response, including use of pragmatic trial designs and exploration of innovative methods for rapid assessment of efficacy and safety" (WHO, 2014a). The proposed alternative designs used a variety of approaches to avoid randomization or to avoid a concurrent control group, including designs that would implement a control group only if a shortage of experimental treatments arose.

Several trial teams proposed using a single-arm trial design, in which study participants are given an experimental agent and their outcomes are compared to expected outcomes based on previous experiences with Ebola, i.e., historical controls. Such designs would remove the requirement for a concurrent control group while obtaining evidence about whether outcomes were better than historical controls. Detractors of this type of study design argued that comparing outcomes of study participants to previous outcomes was not meaningful, because mortality rates for Ebola varied widely and because some study participants might receive better supportive care than others, making it impossible to know if the investigational treatment was responsible for any improvement in observed mortality rates (Cox et al., 2014). This argument is particularly pertinent in an evolving epidemic where patient characteristics may be different at different times in the epidemic. For example, how early an individual seeks care and the quality of the general supportive care available at the time of presentation may change over time. According to a presentation by the U.S. Food and Drug Administration (FDA), "it is far safer to use a concurrently controlled trial than to rely on a historical control unless the effect is very large. If the effect is large, stopping rules can limit the duration and study size so that little time will be wasted" (Temple, 2013). The FDA presentation continued that there was "little reason not to make the first patient trial an RCT, with

Copyright © National Academy of Sciences. All rights reserved.

rare exception" (Temple, 2013). For Ebola, the use of historical controls to assess treatment efficacy may have been ill advised, as varying infection rates and mortality rates were observed over the course of the epidemic and by location, adding considerable risks to the use of historical controls (Nason, 2016).

ETHICAL PERSPECTIVES

While some of the debate over trial design was focused on logistical or scientific considerations, much of the conflict stemmed from disagreements over ethical issues. Planning and conducting scientifically and ethically sound research in the midst of the Ebola epidemic was a complicated task. The early stages of the Ebola epidemic were characterized by widespread uncertainty, anxiety, and mistrust among all health care and public health workers, researchers, and especially the general public and community leaders (Fairhead, 2015). Attacks on treatment facilities and aid workers enhanced the perception of social risk and instability surrounding Ebola treatment (McCoy, 2014). Reports that foreigners who were infected while working in the epidemic response were being cured by Western experimental drugs further complicated the process of engaging communities in an honest discussion of just how little was known about many of the investigational interventions being proposed for study, of why research was needed, and about the relative merits of different trial designs.

In this context, stakeholders disagreed on how to resolve a number of ethical dilemmas. However, while researchers certainly can and should take context into account when planning clinical trials, research conducted during an epidemic is still subject to the same ethical principles that guide all human subjects research. There is now broad consensus about the core requirements for ethical research with human participants and a recognition that in order to conduct research in an emerging crisis, certain standard requirements may require expedited processing or increased flexibility, or both (CIOMS, 2016; COE, 1997; HHS, 2009; National Commission for the Protection of Human Subjects of Biomedical and Behavioral Research, 1979; Nuremberg Code [1947], 1996; UNESCO, 2006; WMA, 2013). For example, in a rapidly moving epidemic where time is of the essence, ethical review may need to be accelerated so as not to unduly delay the start of a valuable study while cases are still appearing.

However, even in such circumstances, the substantive ethical requirements governing research with humans do not change (CIOMS, 2016; Curry et al., 2014). This conclusion is not new. For example, Guideline 20 of the recently revised *CIOMS International Ethical Guidelines for Health-Related Research Involving Humans* specifically addresses "research in disasters and disease outbreaks" and states: "In the conduct of research

Copyright © National Academy of Sciences. All rights reserved.

in disasters and disease outbreaks, it is essential to uphold the ethical principles embodied in these guidelines. Conducting research in these situations raises important challenges, such as the need to generate knowledge quickly, maintain public trust, and overcome practical obstacles to implementing research. These challenges need to be carefully balanced with the need to ensure the scientific validity of the research and uphold ethical principles in its conduct" (CIOMS, 2016). A similar position was stated in a 2009 WHO technical consultation, "Research Ethics in International Epidemic Response" (WHO, 2009). That report states that "even in an infectious disease emergency or other crisis situation, the principles and values embodied in international and national ethics guidelines must be upheld" (WHO, 2009).[4]

Ethics in Human Subjects Research

Since the promulgation of the Nuremburg Code in 1947, numerous efforts have been made by different organizations to codify the basic ethical principles that should govern research with human subjects (CIOMS, 2016; COE, 1997; HHS, 2009; National Commission for the Protection of Human Subjects of Biomedical and Behavioral Research, 1979; Nuremberg Code [1947], 1996; UNESCO, 2006; WMA, 2013). A vast scholarly literature has emerged over time on the central ethical questions, as new situations have been identified and thinking has evolved. The committee has identified seven moral requirements that are widely recognized in authoritative guidance documents and the scholarly literature that are of particular importance for evaluating trials conducted during the Ebola epidemic and other similar circumstances in the future, these requirements are (1) scientific and social value, (2) respect for persons, (3) community engagement, (4) concern for participant welfare and interests, (5) favorable risk–benefit balance, (6) justice in the distribution of benefits and burdens, and (7) post-trial access (see Box 2-3 and Appendix C).

[4] Additional background documents addressing relevant issues are reviewed by Research for Health in Humanitarian Crises (R2HC), a program launched in 2013 through a strategic partnership between the nongovernmental organization Enhancing Learning and Research for Humanitarian Assistance, based at Save the Children (UK), and the Wellcome Trust and the Department for International Development (UK). The aim of the program is to "increase the level and quality of collaborative research on recognised public health challenges in humanitarian crises occurring in low- and middle-income countries" in order "to improve health outcomes by strengthening the evidence base for public health interventions in humanitarian crises." R2HC has been promoting an ethical framework for the development and review of health research proposals to be conducted in the context of an international humanitarian response (R2HC, 2016; Wellcome Trust, 2013).

Copyright © National Academy of Sciences. All rights reserved.

BOX 2-3
Moral Framework for Research

There are fundamental moral requirements that apply to all clinical research, regardless of the community or context in which the research is conducted. The seven specific moral requirements discussed in this chapter (please see Ethics in Human Subjects Research) were chosen by the committee, but have been recognized as essential in authoritative guidance documents, including

1. Nuremburg Code, 1947;
2. Belmont Report, 1979;
3. Convention on Human Rights and Biomedicine, 1997;
4. UNESCO Declaration, 2005;
5. HHS Common Rule, 2009;
6. WMA Declaration of Helsinki, 2013; and
7. CIOMS Ethical Guidelines, 2016.

1. Scientific and Social Value

The value of a study depends on the scientific quality of the information that the study is designed to produce, and the relevance and significance of the information to address an important clinical or public health problem (CIOMS, 2016). In addition, the information that a trial is designed to produce must be of sufficient value to justify the various risks, burdens, and costs associated with the research, including the risks and burdens to the study participants (CIOMS, 2016; COE, 1997; Nuremberg Code (1947), 1996). In the context of a public health emergency, the value of the research conducted should also be sufficient to justify allocating scarce resources—including money, time and energy of caregivers, the use of institutional spaces, and opportunity costs—to research rather than to activities that could impact the emergency more immediately and directly. Ultimately, the value of research depends on whether the information is of sufficient quality to be used to make decisions about care and the allocation of resources. Many stakeholders rely on research data to make decisions that affect the rights and welfare of large numbers of people and that will alter the ways scarce resources are allocated; for example, regulators use data to decide whether to approve a new intervention; third-party payers rely on data to decide which interventions to use, pay for, recommend, or disseminate; and clinicians use research data to make treatment decisions (CIOMS, 2016). Together, these considerations provide strong justification for the default expectation that trials that are conducted during a public

Copyright © National Academy of Sciences. All rights reserved.

health emergency should be designed to produce data that are sufficiently reliable to guide the practice of experts in the medical and public health communities and to meet applicable regulatory standards for the approval and registration of interventions that are demonstrated to be safe and effective (CIOMS, 2016).

2. *Respect for Persons*

In order to be ethical, research with human subjects must always be conducted in ways that demonstrate respect for the individuals and communities that participate in and host the research. Showing respect includes honoring people's fundamental rights, showing genuine concern for their welfare and interests, and allowing them to make momentous decisions about their body or decisions that will affect their welfare or other life prospects. In order to facilitate informed decision making, researchers must provide prospective study participants with relevant, reliable, and understandable information about the choices that are available to them, what risks and possible benefits are associated with each option, why the research is needed, and what will happen if they choose or decline to participate (CIOMS, 2016; COE, 1997; HHS, 2009; National Commission for the Protection of Human Subjects of Biomedical and Behavioral Research, 1979; Nuremberg Code [1947], 1996; UNESCO, 2006; WMA, 2013). Individuals can only be included in a study if they (or a proxy when appropriate, for example research involving children) have voluntarily consented to participate after having understood the associated risks and benefits; if this consent is unconstrained by deception, coercion, or other forms of manipulation; and if they understand that they have the right to withdraw at any time (CIOMS, 2016; COE, 1997; HHS, 2009; National Commission for the Protection of Human Subjects of Biomedical and Behavioral Research, 1979; Nuremberg Code [1947], 1996; UNESCO, 2006; WMA, 2013). In addition to being a moral requirement of research, showing respect for people is critical for building a relationship of trust between researchers and communities; this relationship has major implications not only for the research at hand, but for future interactions between researchers and communities, including patients and their advocates.

3. *Community Engagement*

Emergency situations are often fraught with uncertainty and increased stress and strain on underlying social divisions. This context can exacerbate preexisting mistrust and complicate the process of communicating important information to communities and to prospective study participants, particularly when the circumstances involve life and death decisions and when

Copyright © National Academy of Sciences. All rights reserved.

there is a great deal of uncertainty about potential interventions. It can be challenging to communicate the potential risks and benefits of participating in research, the details of a clinical trial design, and relevant concepts such as randomization, standard of care, control arms, and individual versus societal benefits. Despite the challenges, engaging communities in dialogue about these issues and facilitating an informed decision-making process is critical to showing respect for communities (CIOMS, 2016). (See Chapter 6 for further discussion about community engagement.)

4. *Concern for Participant Welfare and Interests*

Although the goal of clinical research is to answer scientific questions and to generate new information, studies require the participation of individuals whose health and welfare are at stake. As a result, concern for study participants requires that the risks to participants be limited to those that are necessary in order to conduct sound scientific inquiry; gratuitous or unnecessary risks are never justified (CIOMS, 2016; WMA, 2013). The potential risks to participants are not just related to the intervention itself, but also include harms resulting from breaches of confidentiality, violations of privacy, or discrimination or stigma as a consequence of participation (HHS, 2009). In addition to minimizing risks, researchers should also make efforts to increase benefits to the participants (CIOMS, 2016; UNESCO, 2006). In an emergency situation, where participants are particularly vulnerable, it is paramount that research be conducted in ways that advance participant health. Many ethics documents that consider research in humanitarian crisis situations place great emphasis on ensuring benefits to participants (R2HC, 2016). However, this is not always possible, and many of the ethical disagreements about various trial designs during the Ebola epidemic reflect differing views on how to reconcile concern for the welfare of individual participants with concern for scientific and future social value.

5. *Favorable Risk–Benefit Balance*

In the conduct of research, the requirements of sound science and the requirements to respect the health and welfare of study participants may appear to conflict. To be ethically acceptable, research must be designed in a way that maximizes the benefits while minimizing the potential harms (CIOMS, 2016; National Commission for the Protection of Human Subjects of Biomedical and Behavioral Research, 1979; UNESCO, 2006). At one extreme, studies that do not generate reliable scientific information are ethically objectionable because their value does not justify the costs and burdens associated with their conduct. At the other extreme, the knowing

Copyright © National Academy of Sciences. All rights reserved.

neglect or abuse of study participants cannot be justified by advancements to the social good. The challenge for research ethics is reconciling this tension. Some ethicists argue that since people are free to accept personal risks for many different purposes (e.g., recreation), informed individuals should be permitted to voluntarily accept the risks of studies that offer the prospect of generating benefit, as long as the risks have been minimized and are not out of proportion with the value of the information likely to be generated from the trial (Veatch, 2007). However, the dominant approach to this problem holds that this tension can be reconciled when research begins in—and is designed to disturb—a state of "equipoise." Equipoise refers to a state of disagreement or uncertainty in the expert medical community about the relative therapeutic, diagnostic, or prophylactic merits of a set of interventions for a particular health problem (Freedman, 1987). The rationale behind this approach is that because there is no agreement that one of the interventions is superior to the others, then it is ethical to allow participants to be allocated at random to receive one or more of these interventions and then to observe, measure, and document the outcome. "An interpretation of equipoise that requires uncertainty on the part of the individual clinician is not ethically justifiable because it prevents studies that are likely to improve the quality of patient care without the credible expectation that this restriction will improve outcomes" (London, 2017, p. 526). Additionally, if randomized studies increase the prospect of obtaining information that will help to resolve this uncertainty or disagreement, such studies are arguably much more likely to have significant scientific and social value.

6. *Justice in the Distribution of Benefits and Burdens*

To be ethically permissible, the benefits and burdens of research must be fairly distributed. Research should not focus disproportionately on the health needs of some groups while neglecting the health needs of others, and the burdens of research participation should not be borne solely by groups of people who are unlikely to benefit from the knowledge generated. (See Box 2-4 for a discussion on the inclusion of pregnant women and children in clinical trials.) Some groups are particularly vulnerable to neglect or exploitation because of deprivation, disease, marginalization, or oppression. Thus, fairness requires that these groups not be excluded from research nor should they bear a disproportionate share of the burdens of research participation (CIOMS, 2016). Victims of public health emergencies are placed at increased risk and heightened vulnerability in many ways, but also may have unique health needs that cannot be studied outside of the emergency situation. Failing to conduct research in such situations under the guise of protecting the vulnerable would have the adverse effect of perpetuating the knowledge gap about the health condition. Conducting

Copyright © National Academy of Sciences. All rights reserved.

BOX 2-4
Inclusion of Pregnant Women and Children in Clinical Trials

In an epidemic situation, there is greater urgency for clinical trials to quickly identify effective vaccines and therapeutic agents for broad use in the general population. Determining eligibility criteria for inclusion in clinical trials may require the identification of subgroups in the general population at higher risk for infection or who suffer disproportionately severe outcomes as a result of infection. These at-risk groups may include pregnant women and children who often have inherent physiological and pharmacodynamic differences that make extrapolating dosing information gained in clinical trials in nonpregnant adults less informative. Thus, it may be particularly important to consider whether and under what circumstances these groups may be included in clinical trials.

Historically, pregnant women and children have been excluded from clinical trials because they have been considered members of "vulnerable populations," not only because the effects of some interventions could be more adverse and unpredictable for them than for nonpregnant adults, creating potential liability for the researcher, but also because of a concern they are at risk of coercion or undue influence. As a consequence, safeguards were introduced with the goal of protecting the welfare of these subjects (U.S. Code of Federal Regulations, 45 CFR 46, subparts B–D [HHS, 2009]; Declaration of Helsinki [WMA, 2013]). Unfortunately, these safeguards have led to an increasing reluctance to test products in these populations and resulted in a paucity of information on the safety and efficacy of approved products in pregnant women and children (IOM, 1994, 2004). Additionally, when no known effective treatments exist (as in the early days of the HIV epidemic), keeping any group of patients out of clinical trials deprives them and future patients of what may be their only opportunity to receive potentially effective treatment; in the HIV era, this led to the conclusion that many patients were being "protected to death" (Hentoff, 1996).

However, over the last several decades momentum has built to include pregnant women and children in clinical trials (IOM, 1994, 2004) and in fact during the Ebola epidemic, trials did or intended to include pregnant women and children to various degrees. As examples, the Guinea ring vaccination trial actively enrolled children; the EBOVAC vaccine trial planned to enroll children at a later phase of the trial; three therapeutic trials (to study brincidofovir, favipiravir, and ZMapp) enrolled children; and the convalescent plasma trial (Ebola-Tx) enrolled both pregnant women and children. Below we briefly discuss considerations for the inclusion of pregnant women and children in clinical trials.

Pregnant Women

Though policy documents frequently lump children and pregnant women together, the concept of vulnerability applies rather differently to the two groups. Pregnant women are capable of protecting themselves and making decisions about their own medical care. Instead, the hesitation to enroll pregnant women in research stems from the concern that products to be tested may cross the placenta and adversely affect fetal growth, structure, or function (Lyerly et al., 2008). One historical example is the 1961 thalidomide tragedy, in which thousands of

Copyright © National Academy of Sciences. All rights reserved.

pregnant women were prescribed this new sedative for morning sickness despite the absence of studies of its potential effects on the fetus which were, in fact, profound on the developing embryo and fetus (Kim and Scialli, 2011). Pregnant women may, however, continue to be "vulnerable," since their concern about the welfare of their child could outweigh their concern for their own welfare. The exclusion of pregnant woman from clinical trials of experimental agents may later expose the fetus to unnecessary risks from medicinal products previously approved based on studies only in adult males and nonpregnant females. Evidence gained in clinical trials would be particularly valuable because physiological changes in the pregnant woman may alter drug pharmacokinetics, making a drug's metabolism, efficacy, and optimal dosing different from that in men and women who are not pregnant (Feghali et al., 2015). Observational studies and analysis of post-licensure surveillance systems currently provide the bulk of safety information concerning immunization during pregnancy (Fulton et al., 2015). A 2014 WHO review of vaccines suggested vaccinating pregnant women with inactivated vaccines but not live attenuated virus vaccines (WHO, 2014c), due to the concern that live attenuated viruses from vaccines could cross the placenta and infect the fetus, while inactivated vaccines or toxoids would not. According to CDC this is a theoretical concern that has not been demonstrated to be generally true (CDC, 2008).

With comprehensive efforts to provide a realistic sense of the potential risks and benefits of the experimental product and available evidence for safety, pregnant women can decide about participation in a clinical trial, just as they decide about their routine medical care. Both the 2002 and 2016 CIOMS guidelines and the 1994 Institute of Medicine (IOM) report *Ethical and Legal Issues of Including Women in Clinical Studies* reached this same conclusion (CIOMS, 2002, 2016; IOM, 1994). Guideline 19 of CIOMS 2016 states, "Pregnant and breastfeeding women have distinctive physiologies and health needs. Research designed to obtain knowledge relevant to the health needs of the pregnant and breastfeeding woman must be promoted" (CIOMS, 2016, p. 71). The 1994 IOM report stated, "Pregnant women should be presumed to be eligible for participation in biomedical research" (IOM, 1994, p. 17). Yet a 2013 study found that roughly 95 percent of Phase 4 studies that potentially could have included pregnant women actually still chose to exclude them (Shields and Lyerly, 2013). Indeed as Lyerly et al. have remarked, "As with other traditionally excluded populations, progress will not happen until we shift the burden of justification from inclusion to exclusion" (Lyerly et al., 2008, p. 9).

It remains to be seen how updates to the Common Rule in 2017 that removed pregnant women as a population that is "potentially vulnerable to coercion or undue influence" will impact their inclusion in studies (CGR, 2017). An encouraging sign is the call in the 21st Century Cures Act for the establishment of a Task Force on Research Specific to Pregnant Women and Lactating Women, whose duties are to "provide advice and guidance to the Secretary regarding Federal activities related to identifying safe and effective therapies for pregnant women and lactating women" (United States Congress, 2016 [§ 2041(a)(2]).[a] These changes, however, do not currently apply to studies in children and do not eliminate concerns about risks to the fetus.

continued

Copyright © National Academy of Sciences. All rights reserved.

BOX 2-4 Continued

Children

The special concerns for treating and conducting research in children have long been recognized, evidenced by their classification as "vulnerable" subjects (Capron, 1973), originally because they are unable to protect themselves and hence must rely on someone else (typically a parent) who has the will, capacity, and legal standing to protect their interests under the circumstances. Ethical issues have also arisen in the past when children have been unwittingly experimented on in hospitals and orphanages without full disclosure of the benefits or risks (Krugman, 1986). While these ethical concerns are appropriate, they have had the effect of limiting pharmacokinetic studies in children, forcing pediatricians to calculate drug doses based only on studies conducted in adults. Children, however, are not little adults and their age-related differences in metabolism and excretion of medications make the extrapolation of pharmacokinetic data from adults to children problematic. Children are particularly vulnerable to rapidly spreading diseases due to their lack of preexisting immunity, smaller size, and risk of contagion from family members (AAP, 2002). For this reason, countries in Europe and the United States have enacted a number of legal provisions "to encourage, entice or compel pharmaceutical companies to undertake pediatric trials" (Bavdekar, 2013, p. 90).

When conducting clinical trials in children, the goal is to balance ethical concerns with the moral imperative to understand how drugs are metabolized and affect children specifically. This is done, in most cases, by minimizing risks with well-defined inclusion and exclusion criteria. For federally funded researchers in the United States, four categories of research involving children are permitted, with varying degrees of parental permission, assent of the child, where appropriate, and regulatory oversight. These are (1) not greater than minimal risk, (2) a prospect of direct benefit to the child that is at least as favorable as existing therapy and that justifies the risk, (3) slightly higher than minimal risk but holding out no potential benefit to the child where the research is likely to produce information of vital importance regarding the disorder, and (4) research that is not otherwise approvable (typically because it poses more substantial risk and holds out no prospect of direct benefit) but which the Secretary of Health and Human Services concludes, following consultation with experts, provides "a reasonable opportunity to further understanding that could prevent or alleviate a serious problem affecting the health or welfare of children" (45 CFR §§ 46.404–407; HHS, 2009). Similar criteria are set forth in numerous other international guidelines aimed at protecting children as vulnerable research subjects through differentiating risk, limiting harm, and attending to the multiple and complex characteristics of children in order to ethically include them in clinical trials (CIOMS, 2016; EC, 2008; ICH, 2000; Nuffield Council on Bioethics, 2015).

---

[a] 114th U.S. Congress. *H.R.34 - 21st Century Cures Act*. (2016). https://www.congress.gov/bill/114th-congress/house-bill/34 (accessed March 8, 2017).

Copyright © National Academy of Sciences. All rights reserved.

research in emergency situations is an important component of our ability to safely and effectively address the health needs of current and future victims of the emergency situation.

7. Post-Trial Access

When communities host and participate in clinical research on an investigational product that is shown to be effective and safe, there is an ethical obligation to provide post-trial access to the product. Post-trial access is supported by Guideline 2 of the newly released *CIOMS 2016 International Ethical Guidelines for Health-Related Research Involving Humans*, Article 15 of the UNESCO Universal Declaration on Bioethics and Human Rights, and elsewhere (CIOMS, 2016; UNESCO, 2006). While broadly accepted, the concept of post-trial access has been controversial with regard to who bears the costs of the access—the research sponsor, the manufacturer of the product, individual participants in the trial, the host nation, or some other entity. While the principles and practice guiding this aspect of the ethics of clinical trials have not been clearly defined, Nicole Lurie, Assistant Secretary for Preparedness and Response at the Department of Health and Human Services has provided an important perspective in the context of the Ebola epidemic of 2014–2015: "There was a very clear commitment that if we found anything that worked, we are making it available. I just want to be super clear about that. That was never a question."[5]

The Effect of Mortality Rate on Equipoise

During the Ebola epidemic the mortality rate was frequently discussed in deliberations about selecting appropriate trial design. Some stakeholders argued that it would be unethical to randomize patients to a standard-of-care arm, when the current standard of care "does not much affect clinical outcomes and the mortality is as high as 70 percent" (Adebamowo et al., 2014, p. 1423). They argued that in such an environment, "it is problematic to insist on randomizing patients when the intervention arm holds out at least the possibility of benefit," and they maintained that "ethical arguments are not the same for all levels of risk" (Adebamowo et al., 2014, p. 1423). In contrast, proponents of RCTs countered that there were no data to support the assumption that patients with a life-threatening dis-

[5] Testimony of Nicole Lurie, at the time the Assistant Secretary of Preparedness and Response, U.S. Department of Health and Human Services. Public Workshop of the Committee on Clinical Trials During the 2014–2015 Ebola Outbreak, February 23, 2015, Washington, DC.

Copyright © National Academy of Sciences. All rights reserved.

ease would always choose to use a first-in-human experimental product of unknown safety and efficacy (Nelson et al., 2015).

A basic tenet of clinical research is the concept of equipoise, defined as "genuine uncertainty about whether an untested treatment has benefits or risks that exceed those of conventional care" (Adebamowo et al., 2014). The underlying rationale behind this approach is that because there is no agreement that one of the interventions is superior to the others, it is permissible to allow participants to be allocated at random to receive one or more of these interventions and then to observe, measure, and document the outcome. For some, equipoise breaks down or is not applicable in contexts of extremely high mortality and where available options for care offer little benefit (Adebamowo et al., 2014). Because at the time trial designs were being considered it was estimated that Ebola had a mortality rate of 70 percent or more and it was thought that supportive care offered little benefit, the conclusion was reached by some that it was unethical to randomize participants to an investigational agent or to an arm that provided only standard-of-care treatment measures (Caplan et al., 2015; WHO Ebola Response Team, 2014).

There are several problems with this argument. First, it rests on an assumption about the benefits and risks of investigational agents and the consequences of receiving an appropriate standard of care alone that was not supported by sufficient evidence. Notably, for the investigational therapeutic interventions tested during the Ebola epidemic, the available preclinical data were insufficient to determine that a product was more likely to provide a therapeutic advantage to recipients than to worsen their already fragile condition. Given that most of these products were novel and that failure rates for novel interventions in general are in the range of 90 percent, it seems unreasonable to expect that interventions in the early stages of development would have an appreciable therapeutic advantage, let alone have sufficient efficacy to constitute the desired magic bullet (Dawson, 2015; Hay et al., 2014; Thomas et al., 2015). Additionally, in the early stages of the epidemic, some ETUs were unable to provide patients with basic support, including intravenous (IV) fluids and electrolyte management, and offered only oral fluids (MSF, 2016). It was reasonable to expect that mortality rates in patients who did not receive such supportive care would be higher than in patients who did receive necessary physiological support. This calls into question the stark perception that existed, that Ebola had a uniformly high mortality rate and, therefore, that it was futile to try to improve outcomes with the use of standard supportive treatment.

Second, this view seems to presuppose that desperately ill patients cannot be made clinically worse by the adverse effects of potent therapeutic

Copyright © National Academy of Sciences. All rights reserved.

agents or other modalities. However, this is a morally suspect assumption. Even if we assume that Ebola has a 70 percent mortality rate, being given an investigational agent in the early stages of development might lower this risk, but perhaps as likely it might increase it, thereby reducing the survival rate below 30 percent. Third, this position assumes a greater degree of certainty about relevant factors than is warranted. Overall estimates of mortality from emerging infectious diseases are often uncertain and influenced by many factors. Since subclinical cases are often missed or confused for other conditions, mortality estimates can be biased by the fact that only the sickest patients are properly diagnosed (Lipsitch et al., 2015). In retrospect it is clear that initial assumptions about mortality rates and the shape of the epidemic were incorrect. As the response to Ebola improved, the overall mortality rate in the three high-impact countries progressively dropped over the course of the epidemic, from 61.5 percent in July 2014 to 40.7 percent in July 2015; the mortality rate also differed among the three countries, from a high of 66.6 percent in Guinea to 45.1 percent in Liberia and 30.0 percent in Sierra Leone (Johnston, 2015).

Fourth, the position articulated above treats Ebola as an exceptional case. Sound and socially valuable research often takes place among gravely ill participants involving study designs in which novel agents are compared against standard therapies. Preventing such studies on the grounds that they deny sick patients the chance of receiving a potentially beneficial intervention would create or exacerbate gaps in our knowledge about how best to treat the patients with such conditions. Preventing these studies would reduce our ability to efficiently form an accurate picture of the relative merits and hazards of novel interventions. At the height of the AIDS crisis, before there were any proven treatments, the mortality rate of untreated AIDS was essentially 100 percent. While AIDS differs from Ebola in many ways (e.g., incubation period), similar arguments were made against placebo-controlled randomized trials from AIDS activists such as AIDS Coalition to Unleash Power (ACT UP) (Crimp, 2011; Dawson, 2015; KFF, 2014). In an effort to address their concerns, a group of statisticians (Byar et al., 1990) advocated for randomization in AIDS trials, but also clearly articulated a limited set of conditions for which randomization may not be appropriate. While a universally poor prognosis was one condition, it could not be the only one; rather, Byar et al. argued, all five must apply for uncontrolled trials to be warranted (see Box 2-5).

These considerations support the view that equipoise is applicable to emergency contexts and that it is therefore ethically acceptable to offer participants the chance to participate in a trial that begins in and is designed to disturb a state of equipoise (Nelson et al., 2015).

Copyright © National Academy of Sciences. All rights reserved.

BOX 2-5
Special Situations in Which Uncontrolled Phase 3 Trials May Be Warranted

All conditions must apply:

1. There must be no other treatment appropriate to use as a control.
2. There must be sufficient experience to ensure that the patients not receiving the therapy will have a uniformly poor prognosis.
3. The therapy must not be expected to have substantial side effects that would compromise the potential benefit to the patient.
4. There must be a justifiable expectation that the potential benefit to the patient will be sufficiently large to make interpretation of the results of a nonrandomized trial unambiguous.
5. The scientific rationale for the treatment must be sufficiently strong that a positive result would be widely accepted.

SOURCE: Byar et al., 1990.

Determining an Ethical Comparator

Debate about clinical trial designs during the Ebola epidemic also focused on what might constitute an ethical comparator for the evaluation of novel interventions. The choice of comparator is important because it provides the benchmark against which novel interventions are assessed. If a sick person receives a novel intervention and his or her condition improves or worsens, the benefit or harm cannot be attributed to the intervention unless we know what would have happened to that person without the intervention. Because we cannot know the answer to this counterfactual directly, we compare the effect of giving a novel intervention to some patients against a comparator group.

Substantial confusion can arise in the discussion of study comparators because some commonly used terms are themselves either misleading or are frequently used in ways that can be confusing. For example, the claim that a novel intervention will be compared against a placebo control is sometimes taken to be synonymous with the claim that it will be compared against "no treatment." In this instance potential trial participants may believe that those in the comparator arm will not receive any medical care or treatment of any kind. However, this is rarely if ever the case, as both arms will typically receive standard supportive care. Similarly, the statement that a novel intervention is being compared against a placebo is often used to describe two very different situations. The first is what might be called a "placebo-

Copyright © National Academy of Sciences. All rights reserved.

only" comparison, in which members of the investigational arm receive no therapeutic interventions other than the novel intervention being tested and members of the comparator arm receive no therapeutic intervention other than an inert substance that is delivered in the same manner and looks like the novel intervention. In a "placebo-add-on" design, each person in the investigational and comparator arms of the study receives a standardized treatment package as part of the baseline of his or her care and treatment (see Box 2-6 for further discussion on standard care) (Gupta and Verma, 2013). Members of the investigational arm then receive that investigational agent "on top of" this baseline of care, and members of the comparator arm receive a placebo on top of this same baseline package of care.

During the Ebola epidemic, some stakeholders objected to a placebo controlled design, unequivocally stating that trials "should not include a placebo: exposed and vulnerable people in Ebola-affected and low-resource settings shouldn't be led to think they are either being treated or protected when they're not" (MSF, 2014b). Objectors to the use of a placebo also argued that it could be unethical or logistically implausible to administer a placebo treatment to such sick patients, for example, "giving 12 to 24 placebo tablets to a vomiting Ebola patient or a 6-hours-lasting placebo infusion to a patient with coagulopathy."[6] While RCTs do not require the use of a placebo, placebos are used to facilitate blinding and thereby control for other factors that may influence patient outcomes, such as the conduct of caregivers[7] (Vickers and de Craen, 2000). The committee determined that such considerations do not warrant the a priori rejection of the use of a placebo but rather should be taken into consideration within the specific context of a trial.

Resistance to studies that would compare a new intervention to a placebo control may have stemmed partly from a misperception that participants in such studies would be denied all care, including such supportive interventions as aggressive rehydration and management of electrolyte abnormalities. Such a "placebo-only" design would have been unethical, but a placebo-add-on design would have been both ethically permissible and scientifically desirable.

No therapeutics trial that was conducted in the three countries in 2014–2015 used a placebo in the standard-of-care study arm (a placebo add-on); rather when a concurrent control was used, for example in the

[6] Personal communication, Annick Antierens, Médecins Sans Frontières, March 25, 2015. Trial designs in epidemic emergencies: The perspective of caretakers and aid workers, based on the experience in the 2014–2015 Ebola outbreak.

[7] An additional consideration is that of the "placebo effect." The placebo effect is a beneficial effect, produced by a placebo drug or treatment, that cannot be attributed to the properties of the placebo itself, and must therefore be due to the patient's belief in that treatment (Oxford English Dictionary, 2016).

Copyright © National Academy of Sciences. All rights reserved.

BOX 2-6
The Standard of Care Owed to Research Participants

There has been much debate in the past about what standard of care is owed to trial participants. The Nuffield Council's position is that "wherever appropriate, participants should be offered the best standard of care available in the world for the disease being studied. But this is not always appropriate or possible" (Nuffield Council on Bioethics, 2002). For example, given the setting and infrastructure, the Ebola epidemic was not a situation in which the best available standard of supportive care anywhere in the world could have been provided. The technology and staffing that would have been required was far beyond what was feasible. The local standard of care varied somewhat across treatment units. For example, some provided intravenous (IV) fluids, while some provided only oral fluids because they lacked IV tubing. In these situations, the Nuffield Council recommends, "As a minimum participants should be offered the best treatment available from the national public health system" (Nuffield Council on Bioethics, 2002). If the experimental and control arms of the study use the best care available through the national public health system, this would mean that sites that cannot meet this standard cannot be clinical trial sites, and the perception that a control arm means no care would not be supported by evidence. Standard-of-care control arms in trials often receive better care and have better outcomes than if they were given clinical care outside the research setting (Braunholtz et al., 2001).

This position differs slightly from that of the U.S. Presidential Commission, which recommends that the standard should be the best care sustainable in the community where the research is conducted and where the intervention will be used, with the reasoning that the level of supportive care that is provided during a trial needs to be locally sustainable after the trial, in part because the effectiveness of the treatment intervention may depend on it (Presidential Commission for the Study of Bioethical Issues, 2015). Experiences from the setting of reducing the risk of mother-to-child transmission of HIV provide important insights about this issue (Fleming and Ellenberg, 2016). Which standard of care to use is context dependent, however, regardless of the standard applied, the committee believes: "it must be defined in consultation with those who work within the country and must be justified to the research ethics committee" (Nuffield Council on Bioethics, 2002).

Better approaches to presenting and communicating the details of each element of the trial designs might have avoided the misunderstandings about control groups during the Ebola trial discussions and strengthened support for RCTs.

ZMapp trial, it was compared to a standard-of-care arm alone. However, it is possible that the initial discussion of including a placebo polarized the debate about randomized trials. Those who argued against RCTs may have mistakenly believed that patients randomized to the control group would receive no care, rather than the standard of care. In reality, at the

Copyright © National Academy of Sciences. All rights reserved.

beginning of the epidemic, given the capacity of the health care system and infrastructure and the conditions of working in containment in a hastily constructed facility, the standard of care that was generally available was quite limited. However, the clinical trials that used a standard-of-care arm made a concerted effort to provide the best possible supportive care available. For example, the PREVAIL II trial of ZMapp (which had the advantage of starting later when better supportive care was available) had an "optimized-standard-of-care" control arm in which patients received IV fluids, monitoring of electrolytes and key biochemical parameters, and maintenance of oxygenation and blood pressure support and treatment for other infections when they were identified (Davey, 2016). The PREVAIL II trial also demonstrated that individuals would consent to participation in an RCT when it was clearly explained to them. Nelson et al. wrote that "to build trust, all efforts should be made to improve the local standard of care to include early rapid diagnostic testing, the provision of intravenous fluids, and electrolyte management—all of which are known to be effective in reducing the mortality of Ebola. However, the provision of such resources reinforces the need for a concurrent control group, as such interventions are likely to affect mortality" (Nelson et al., 2015) (see Chapter 6 for further discussion on community engagement). While the availability of treatments remained somewhat variable based on trial site, the patients in the standard-of-care arm and the active arm received the same supportive care, with the only difference between the groups being the provision of the investigational medicinal product. Most of the Ebola trials that were conducted provided all trial participants with supportive care, including IV fluids, hemodynamic or electrolyte monitoring or both, and adjunctive medications (e.g., antimalarials, antibiotics) (Dunning et al., 2016a,c; MSF, 2015; PREVAIL II Writing Group, 2016; Sissoko et al., 2016; van Griensven et al., 2016).

Authoritative research ethics guidance documents hold that, as a general rule, research subjects in the control group of a trial of a diagnostic, therapeutic, or preventive intervention should receive an established effective intervention. However, there are some circumstances under which it may be ethically acceptable to compare a novel intervention against a placebo-only comparator. As stated in CIOMS guidelines, such a placebo may be used when

- there is no established effective intervention;
- withholding an established effective intervention would expose subjects to, at most, temporary discomfort or delay in relief of symptoms; and
- use of an established effective intervention as comparator would not yield scientifically reliable results and use of placebo would

Copyright © National Academy of Sciences. All rights reserved.

not add any risk of serious or irreversible harm to the subjects (CIOMS, 2016).

Guideline 33 of the Declaration of Helsinki (2013) also states that the benefits, risks, burdens, and effectiveness of a new intervention must be tested against those of the best proven intervention(s), except in the following circumstances:

- Where no proven intervention exists, the use of placebo, or no intervention, is acceptable; or
- Where for compelling and scientifically sound methodological reasons the use of any intervention less effective than the best proven one, the use of placebo, or no intervention is necessary to determine the efficacy or safety of an intervention and the patients who receive any intervention less effective than the best proven one, placebo, or no intervention will not be subject to additional risks of serious or irreversible harm as a result of not receiving the best proven intervention. Extreme care must be taken to avoid abuse of this option (WMA, 2013).

Given the severity of Ebola and the likely effects of leaving severe dehydration untreated, a placebo-only trial would not meet the above exceptions and, therefore, would not have been ethically acceptable. Because a placebo-add-on design would not deny any study participant access to currently accepted treatment for Ebola, such comparator arms would be ethically permissible so long as the provision of the placebo add-on would be feasible and could be performed safely. The committee determined that testing a novel intervention against a standard-of-care comparator without a placebo add-on is less desirable from a methodological point of view, but is also ethically acceptable, particularly where administering a placebo involves risk to providers or patients (e.g., because of the difficulty of injecting highly infectious patients). Whether IV fluids are actually provided is another issue. As Lamontagne et al. observed, "A common assumption is that a lack of material resources constitutes the dominant barrier to clinical care. That is not the case. Intravenous catheters, fluids, and electrolyte replacement are readily available but thus far are being used much too sparingly. . . . There is a historical bias against aggressive interventions, including intravenous cannulation, for many transmissible illnesses. Percutaneous injury to health care workers does carry substantial risk, but such risks are not specific to Ebola" (Lamontagne et al., 2014, p. 1566).

On this point, it is worth considering the implications of a standard of care or placebo control group with some level of the risk of serious harm to

Copyright © National Academy of Sciences. All rights reserved.

subjects, researchers, or caregivers. For some therapies, for example, using IVs for rehydration or administering a placebo in infected people presented risks to caregivers, as administering an IV involves potential risk from an accidental needle stick to the person doing the infusion. However, as it became common to provide IV fluids as part of standard supportive care the evidence suggests the marginal increase in risk was modest. In March 2015 Partners In Health stated that "responders started putting IVs in children more regularly to resuscitate and rehydrate them. In some cases, they used intraosseous lines (inserted into the bone) if insertion into a vein wasn't possible. . . . The more aggressive use of hydration has certainly dropped mortality rates" (Partners In Health, 2015). The result was that IV rehydration became standard care for all patients at the Maforki Ebola Treatment Unit in Port Loko, Sierra Leone. In areas where IV rehydration was already the standard of care and the health care team had experience, the additional risk to researchers and caregivers to administer an IV placebo, if deemed necessary, would be limited, although needle-stick injury is a well-described hazard for transmission of Ebola (Guardian, 2015). The greater exposure risk may actually result from the more extensive bedside monitoring and direct contact with patients or with the equipment used for supportive care, such as respirators. During the Ebola-TKM trial, monitoring occurred over the 2-hour infusion period, with additional assessment of vital signs before, during, and at 1, 2, 4, and 8 hours after the end of infusion (Dunning et al., 2016c). For these reasons the committee determined that the additional risk involved with administering an IV placebo is minimal because IV fluid replacement is considered standard of care for Ebola and all patients with dehydration should be receiving fluids, whether they are in the treatment or the control arm of a study.

It might, however, be challenging to allocate the manpower required to monitor a patient receiving a placebo or IV for an extended period of time. It can be argued that unless researchers are able to provide all of the necessary resources to conduct a trial properly and not interrupt clinical care routines, they ought not to proceed. A call for more volunteers to carry out protocol requirements would be relevant, but it may also be insensitive or naïve to simply call for more manpower. The priority is to set up enough facilities and staff to deliver a site-specific optimized standard of care. With that in place, RCTs are more palatable and readily implemented.

In principle, there is the danger that providing an enhanced standard of care even in the control arm might push people to enroll in trials (McMillan and Conlon, 2004); however, it is unclear whether this was a factor in community members' decisions to enroll in Ebola trials.

Copyright © National Academy of Sciences. All rights reserved.

Limited Product Availability

Supplies of some of the experimental therapies being considered for Ebola were limited, and this influenced perspectives on the appropriateness of RCTs under these circumstances. Objectors to RCTs believed that RCTs would deprive some patients of access to treatment. Proponents of RCTs countered that "given the scarcity of the drug, a finite number of patients will receive access regardless of what study design is used" (Joffe, 2014, p. 1300), and they maintained that RCTs would actually be a fair and ethical way to allocate resources while gathering data that could help future Ebola patients (Cox et al., 2014). Further, supporters of randomization noted that "alternative means for prioritizing access, such as first-come first-served and sickest first, are themselves ethically unsatisfactory" (Joffe, 2014, p. 1300) and would fail to generate interpretable evidence.

The argument for randomization seems especially strong in the case of a limited supply of product. If there were, for example, just 10 doses of an experimental therapy available, would it be rational to give it to the first 10 people who showed up and agreed to its administration when this may sacrifice the ability to learn something about its efficacy and safety? Would the decision be different if there were 100 doses? In contrast, if an intervention is in surplus and randomization is applied, it might appear to some observers that the researchers are "withholding" the intervention from some patients. All trial designs need to enroll enough subjects to reach interpretable endpoints and there needs to be enough available product for the patients randomized to the experimental treatment. However, even small randomized studies can provide a provisional assessment of efficacy that a first-come/first-served approach cannot, unless the effect is very dramatic. If not enough doses to conduct a suitable trial are available, the use of a randomized lottery system to distribute the available doses would still be preferential because it would be the fairest possible way to distribute scarce resources and retain the potential to generate useful information. The Ebola report of the Presidential Commission for the Study of Bioethical Issues presented two competing perspectives on obligations to Africans confronting Ebola (Presidential Commission for the Study of Bioethical Issues, 2015):

1. Identify safe, effective interventions as efficiently and reliably as possible.
2. Provide access to the potential benefits of experimental interventions to as many people as possible using scientifically valid research designs.

For scarce interventions during the epidemic, like ZMapp, there may be no conflict between these two obligations. But where the intervention

Copyright © National Academy of Sciences. All rights reserved.

Integrating Clinical Research into Epidemic Response: The Ebola Experience

may be given to a large number of people (e.g., vaccines), there may be controversy over a randomized trial because there must be a risk of infection distributed across the population to be able to assess efficacy, and some subjects will be denied the potential benefit of the product. On the other hand, they are spared the potential adverse effects of the vaccine. Many still recall the widespread administration of an experimental vaccine in the United States to protect against the emerging influenza A H1N1 New Jersey 1976 strain that provided no protection because the anticipated epidemic did not happen; however, there was a sharp increase in the number of individuals with Guillain-Barre syndrome in the weeks following immunization, and 25 deaths were attributed to the vaccine (Langmuir, 1979).

Community Trust

Some argued that mistrust of health care workers by the community would deter trial enrollment and the generation of meaningful data (CIOMS Guideline 1) (Caplan et al., 2015). They also argued that the sharing of drugs (to ensure that more people had access to the active agent, as seen in early HIV trials with AZT [Farber, 2015]) might compromise trial results. However, previous experiences in confronting the HIV epidemic revealed that controlled conditions of a randomized trial are possible even in developing country settings, and even where there is a sense of urgency about addressing an emerging epidemic (Lane et al., 2016). These concerns, in combination with the violence and mistrust the affected population had shown toward both ETUs and health care workers, led some to assume that randomization would be locally unacceptable (Adebamowo et al., 2014; McCoy, 2014). Waldman and Neiburg expanded on this, emphasizing that the confusion and volatility of the situation would make it challenging for individuals to understand what the standard of care was (Waldman and Nieburg, 2015). They worried that the foreign care providers would be perceived as providing potentially lifesaving treatments, thus enhancing therapeutic misperceptions. At the very least, this situation would make fully informed consent challenging. “RCTs will not work without community trust,” Caplan et al. wrote, “yet implementing them risks eroding that trust” (Caplan et al., 2015, p. 7).

It is critically important, however, that researchers not make assumptions about how communities perceive prospective research activities. In order to lay the groundwork for ethical research, there has to be truthful engagement with the community about the tradeoffs inherent to clinical trials so they can make an informed decision about the trial designs that can be implemented. If trial teams rush to enter a community in order to rapidly implement a trial without having a proper engagement strategy, it can backfire, as observed during the latter months of 2014. When the teams

Copyright © National Academy of Sciences. All rights reserved.

can engage the community and provide clearly articulated information about the various aspects of trial designs, community buy-in for research is possible, as documented by the experience in West Africa. For example, the PREVAIL team demonstrated their ability to propose and implement an RCT in Liberia in early 2015 (Wilson et al., 2016). Respect for communities requires that their members are engaged in a process of dialogue and exchange with the investigators about the need for research, the nature of the uncertainty to be addressed, what is known about the status of the interventions to be used, and the merits of possible trial designs. As the committee heard through testimony in their meeting in Liberia, communities, even those that were previously unexposed to clinical trials, are capable of understanding components of research when it is explained. In a context of scarcity, need, and heightened mistrust, such conversations can be challenging. But they are an indispensable component of ethically sound research and are critical to treating study communities as full partners in the effort to find the means to advance their health needs.

However, if after substantial and genuine community discussion and engagement there is still extensive opposition to trials that involve randomization, it becomes reasonable to consider alternative trial approaches. When this course is taken there must be a commitment to avoid supporting any design that is unlikely to produce sufficiently reliable evidence in order to offset the many risks, costs, and burdens associated with research. The study design proposed by Cooper et al., using a single-arm study at the outset and then moving to a randomized trial if the results are promising but neither exceptional nor limited, is an effort to address the concerns of such communities while recognizing that views about the acceptability of randomization may evolve as evidence accumulates and it is clear that an intervention is or is not highly efficacious (Cooper et al., 2015). Such multistage study designs may represent prudent options in such circumstances, when despite engagement and information exchanges, communities or the health authorities in a country will not accept the inclusion of an add-on control group in a clinical trial. Research must be conducted in a responsible and locally acceptable fashion, with attention paid to the local communities' values, beliefs, and priorities. Failing to conduct research in this manner risks more than the success of the research project; it can also jeopardize the trust and relationships that allow clinical care to be delivered to the community. (See Chapter 6 for further discussion of community engagement.)

CONCLUSIONS

The features of the early days of the Ebola epidemic—high mortality rate, rumors, fears, and uncertainty—were part of the context in which stakeholders had to evaluate the designs for clinical trials during the sum-

Copyright © National Academy of Sciences. All rights reserved.

mer of 2014, when the need for and the opportunity to conduct clinical trials became fully apparent. Specifically, the desire to find a highly effective treatment that could be deployed in the epidemic at hand, the belief that the mortality rates from Ebola were extremely high, the potential conflict between research and patient care, and the perception that communities would not agree to study designs in which they were denied access to potentially helpful investigational agents no doubt played a strong role in the support for nonrandomized, uncontrolled study designs.

In evaluating the single-arm trials that used historical data for comparison (the design of many of the therapeutics trials, described in detail in Chapter 3), there are two important questions. First, can the trial as designed answer the research questions that it is asking? In this regard, such designs seem most reasonable when there are preliminary data from preclinical or clinical trials that are highly suggestive of efficacy, the natural history of the disease is uniform and well understood, and there is a stable and high mortality rate. This type of design would then be used to address the goal of identifying a highly efficacious intervention that could effectively stop the epidemic. However, such designs cannot reliably identify moderately effective interventions, or identify any potential serious adverse events of the interventions that were distinct from those of the disease itself, particularly given the minimal natural history on Ebola. The second question is whether the questions asked by the study were the right ones (e.g., whether a single-arm trial design to find a highly efficacious medicinal product in the context of Ebola was a reasonable one), or whether it would be preferable at the outset of future outbreaks to employ study designs that are capable of generating information that can support incremental progress in understanding and addressing Ebola or another similar infectious disease.

Each issue discussed in this chapter highlights an important aspect of research involving human participants that must be addressed to ensure that the design and conduct of a study is ethically acceptable. Reconciling the demands of these requirements in specific cases can be challenging. In an emergency, research must be responsive to the particular health needs that arise in that context, while being designed and conducted so as to ensure that the rights, interests, and autonomy of study participants are respected. To reiterate, to be ethically acceptable, research must have a realistic prospect of generating information that constitutes an adequate basis for learning. In the case of an emerging infectious disease outbreak, ethically acceptable research must provide the information needed to put stakeholders in a better position to understand and make decisions regarding the use of new interventions and to address similar outbreaks in the future (CIOMS, 2016). In this regard, there is a strong, default presumption in favor of the strongest research design that is feasible to implement, considering both logistical constraints and cultural acceptance together with

Copyright © National Academy of Sciences. All rights reserved.

the highest scientific standards of excellence. The ideal output from research is to obtain maximum scientific benefit by generating valid interpretable knowledge that can be applied to the affected population in current and future outbreaks.

Given the complexity of conducting research in low-resource settings during an infectious disease outbreak, it is also essential that research designs be feasible and can be implemented under the constraints of the outbreak and the response to it. The reality is that what is feasible may change over the course of the epidemic (e.g., as caseloads rise or fall, more facilities, health care workers and resources become available, knowledge grows, and process efficiencies are realized). Similarly, the risk–benefit balance of trial designs may change over time, depending on how the epidemic, standard of care, and treatment alternatives evolve. Thus, as an ethical matter in emergencies involving great uncertainty about key parameters of the disease, trial design decisions should be subject to close monitoring and potential reconsideration or adaptation. These decisions are not restricted to a single point during the study, but must be revisited as needed as the outbreak evolves over time. In this regard, trials must be designed in ways that permit periodic reassessment of the original design decisions to ensure that they still make the most sense, both ethically and scientifically. Context matters.

While the Ebola epidemic was unique in many respects, the ethical issues raised were not unprecedented and have been encountered in previous events and epidemics, including HIV, severe acute respiratory syndrome (SARS) and tuberculosis (KFF, 2014). Much can be learned from prior debates over research involving desperately ill patients, research conducted during humanitarian or public health emergencies, research in emergency room situations and with an unconscious patient, and research in resource-poor settings (Wainberg et al., 2014). Similarly, lessons learned from the Ebola epidemic will provide insights for the future. The issues that influenced choices about trial design during the Ebola epidemic—community mistrust, the feasibility of a standard-of-care-only arm, the early high mortality rate, limited product availability, and the potential conflicts between research and care—are likely to recur in future epidemics. However, the perceived ethical or logistical hurdles that these issues present are not sufficiently compelling to override the benefits of randomized trials. Rather, RCTs may be seen as the most ethical trial design in a context such as the Ebola epidemic because they offer the fastest route to identifying beneficial treatments while minimizing the risks of exposure to potentially harmful investigational agents.

Researchers have an ethical obligation to undertake efforts to help ensure that RCTs are locally acceptable. Community engagement, in particular, is an essential element to the conduct of successful clinical trials; a

Copyright © National Academy of Sciences. All rights reserved.

Integrating Clinical Research into Epidemic Response: The Ebola Experience

community that is informed of the risks and benefits of RCTs and is engaged in the planning process from the beginning is more likely to actively participate in research efforts. Failing to conduct clinical research in a way that considers and addresses community concerns jeopardizes the success of the entire research enterprise. It can also jeopardize trust and relationships that permit clinical care to be delivered, and for vaccine trials, it can jeopardize trust in the whole immunization system. The stakes are very high.

> ***Conclusion 2-3*** *Randomized controlled trials are the most reliable way to identify the relative benefits and risks of investigational products, and, except when the rare circumstances detailed in Box 2-5 are applicable, every effort should be made to implement them during epidemics.*

REFERENCES

AAP (American Academy of Pediatrics). 2002. The youngest victims: Disaster preparedness to meeting children's needs. https://www.aap.org/en-us/Documents/disasters_youngest_victims_disaster_preparedness.pdf (accessed February 17, 2017).

Adebamowo, C., O. Bah-Sow, F. Binka, R. Bruzzone, A. Caplan, J.-F. Delfraissy, D. Heymann, P. Horby, P. Kaleebu, J.-J. M. Tamfum, P. Olliaro, P. Piot, A. Tejan-Cole, O. Tomori, A. Toure, E. Torreele, and J. Whitehead. 2014. Randomised controlled trials for Ebola: Practical and ethical issues. *The Lancet* 384(9952):1423–1424.

Bavdekar, S. B. 2013. Pediatric clinical trials. *Perspectives in Clinical Research* 4(1):89–99.

Branswell, H. 2014. *Ebola free-for-all could trigger bad science and wasted efforts*. https://www.scientificamerican.com/article/ebola-free-for-all-could-trigger-bad-science-and-wasted-efforts (accessed January 20, 2017).

Braunholtz, D. A., S. J. L. Edwards, and R. J. Lilford. 2001. Are randomized clinical trials good for us (in the short term)? Evidence for a "trial effect." *Journal of Clinical Epidemiology* 54(3):217–224.

Byar, D. P., D. A. Schoenfeld, S. B. Green, D. A. Amato, R. Davis, V. De Gruttola, D. M. Finkelstein, C. Gatsonis, R. D. Gelber, S. Lagakos, M. Lefkopoulou, A. A. Tsiatis, M. Zelen, J. Peto, L. S. Freedman, M. Gail, R. Simon, S. S. Ellenberg, J. R. Anderson, R. Collins, R. Peto, and T. Peto. 1990. Design considerations for AIDS trials. *New England Journal of Medicine* 323(19):1343–1348.

Caplan, A. L., C. Plunkett, and B. Levin. 2015. Selecting the right tool for the job. *The American Journal of Bioethics* 15(4):4–10.

Capron, A. M. 1973. Legal considerations affecting clinical pharmacological studies in children. *Clinical Research* 21:141–150.

CDC (U.S. Centers for Disease Control and Prevention). 2008. *ACIP: Guidance for vaccine recommendations for pregnant and breastfeeding women*. https://www.cdc.gov/vaccines/acip/committee/downloads/preg-principles-2008.pdf (accessed March 17, 2017).

CGR (Council on Governmental Relations). 2017. Common rule overview. http://www.cogr.edu/sites/default/files/Summary%20of%20Changes%20to%20the%20Common%20Rule_COGR.pdf (accessed February 20, 2017).

Chang, M., and S. C. Chow. 2011. *Adaptive design methods in clinical trials, second edition*, Chapman & Hall/CRC Biostatistics Series. Boca Raton, FL: Chapman & Hall/CRC Press.

Copyright © National Academy of Sciences. All rights reserved.

CIOMS (Council for International Organizations of Medical Sciences). 2002. International ethical guidelines for biomedical research involving human subjects. Geneva: CIOMS.

CIOMS. 2016. *International ethical guidelines for biomedical research involving human subjects*. http://www.cioms.ch/ethical-guidelines-2016 (accessed January 20, 2017).

COE (Council of Europe). 1997. Convention for the protection of human rights and dignity of the human being with regard to the application of biology and medicine: Convention on Human Rights and Biomedicine. *Kennedy Institute of Ethics Journal* 7(3):277–290.

Cohen, J., and K. Kupferschmidt. 2014. Tough choices ahead in Ebola vaccine trials. *Science Magazine*, October 7, 2014.

Cooper, B. S., M. F. Boni, W. Pan-ngum, N. P. J. Day, P. W. Horby, P. Olliaro, T. Lang, N. J. White, L. J. White, and J. Whitehead. 2015. Evaluating clinical trial designs for investigational treatments of Ebola virus disease. *PLoS Medicine* 12(4):e1001815.

Cox, E., L. Borio, and R. Temple. 2014. Evaluating Ebola therapies — the case for RCTs. *New England Journal of Medicine* 371(25):2350–2351.

Crimp, D. 2011. *Before occupy: How AIDS activists seized control of the FDA in 1988*. http://www.theatlantic.com/health/archive/2011/12/before-occupy-how-aids-activists-seized-control-of-the-fda-in-1988/249302/ (accessed January 20, 2017).

Curry, D., R. Waldman, and A. Caplan. 2014. *An ethical framework for the development and review of health research proposals involving humanitarian contexts: Project final report, January 2014*. http://www.alnap.org/resource/10687 (accessed January 20, 2017).

Darrow, J. J., A. Sarpatwari , J. Avorn, and A. S. Kesselheim. 2015. Practical, legal, and ethical issues in expanded access to investigational drugs. *New England Journal of Medicine* 372(3):279–286.

Davey, R. T. J. 2016. PREVAIL II: A randomized controlled trial of ZMapp™ in acute Ebola virus infection. Paper presented at CROI 2017, Seattle, WA.

Davis, M. 2015. *In rush to develop Ebola therapies, a debate over placebo control*. http://blog.bioethics.gov/2015/02/05/in-rush-to-develop-ebola-therapies-a-debate-over-placebo-control (accessed December 21, 2016).

Dawson, L. 2015. Not all RCTs are created equal: Lessons from early AIDS trials. *The American Journal of Bioethics* 15(4):45–47.

Dresser, R. 2009. First-in-human trial participants: Not a vulnerable population, but vulnerable nonetheless. *The Journal of Law, Medicine & Ethics: A Journal of the American Society of Law, Medicine & Ethics* 37(1):38–50.

Dunning, J., S. B. Kennedy, A. Antierens, J. Whitehead, I. Ciglenecki, G. Carson, R. Kanapathipillai, L. Castle, R. Howell-Jones, R. Pardinaz-Solis, J. Grove, J. Scott, T. Lang, P. Olliaro, and P. W. Horby, for the RAPIDE-BCV trial team. 2016a. Experimental treatment of Ebola virus disease with brincidofovir. *PLoS ONE* 11(9):e0162199.

Dunning, J., C. Longuet, and A. Salam. 2016b. Ebola research: An encounter between science and humanitarian action. *Alternatives Humanitaires/Humanitarian Alternatives* 1(1):81–93.

Dunning, J., F. Sahr, A. Rojek, F. Gannon, G. Carson, B. Idriss, T. Massaquoi, R. Gandi, S. Joseph, H. K. Osman, T. J. G. Brooks, A. J. H. Simpson, I. Goodfellow, L. Thorne, A. Arias, L. Merson, L. Castle, R. Howell-Jones, R. Pardinaz-Solis, B. Hope-Gill, M. Ferri, J. Grove, M. Kowalski, K. Stepniewska, T. Lang, J. Whitehead, P. Olliaro, M. Samai, and P. W. Horby, for the RAPIDE-TKM trial team. 2016c. Experimental treatment of Ebola virus disease with TKM-130803: A single-arm phase 2 clinical trial. *PLoS Medicine* 13(4):e1001997.

EC (European Commission). 2008. *Ethical considerations for clinical trials on medicinal products conducted with the paediatric population*. https://ec.europa.eu/health/sites/health/files/files/eudralex/vol-10/ethical_considerations_en.pdf (accessed March 8, 2017).

Enserink, M. 2014a. Ebola drugs still stuck in lab. *Science* 345(6195):364–365.

Copyright © National Academy of Sciences. All rights reserved.

Enserink, M. 2014b. How two U.S. patients changed the debate about using untested Ebola drugs. *Science*, August 7. http://www.sciencemag.org/news/2014/08/how-two-us-patients-changed-debate-about-using-untested-ebola-drugs (accessed January 25, 2017).

Fairhead, J. 2015. Understanding social resistance to Ebola response in Guinea: An anthropological perspective. *African Studies Review* 59(3):7–31.

Farber, C. 2015. *AIDS and the AZT scandal:* Spin's *1989 feature, "Sins of omission."* http://www.spin.com/featured/aids-and-the-azt-scandal-spin-1989-feature-sins-of-omission/ (accessed January 25, 2017).

FDA (U.S. Food and Drug Administration). 2010. Guidance for industry: Adaptive design clinical trials for drugs and biologics.

FDA. 2016. *Expanded access (compassionate use)*. http://www.fda.gov/NewsEvents/PublicHealthFocus/ExpandedAccessCompassionateUse/default.htm (accessed December 21, 2016).

Feghali, M., R. Venkataramanan, and S. Caritis. 2015. Pharmacokinetics of drugs in pregnancy. *YSPER Seminars in Perinatology* 39(7):512–519.

Fleming, T. R., and S. S. Ellenberg. 2016. Evaluating interventions for Ebola: The need for randomized trials. *Clinical Trials (London, England)* 13(1):6–9.

Freedman, B. 1987. Equipoise and the ethics of clinical research. *New England Journal of Medicine* 317(3):141–145.

Fulton, T. R., D. Narayanan, J. Bonhoeffer, J. R. Ortiz, P. Lambach, and S. B. Omer. 2015. A systematic review of adverse events following immunization during pregnancy and the newborn period. *Vaccine* 33(47):6453–6465.

Gericke, C. A. 2015. Ebola and ethics: Autopsy of a failure. *British Medical Journal* 350:h2105.

Goodman, J. L. 2014. Studying "secret serums"—Toward safe, effective Ebola treatments. *New England Journal of Medicine* 371(12):1086–1089.

Guardian. 2015. *Second UK military Ebola worker flies back home after needlestick injury*. https://www.theguardian.com/world/2015/feb/02/second-ebola-worker-uk-needlestick-royal-free-diagnosis (accessed January 20, 2017).

Gupta, S. K. 2012. Use of bayesian statistics in drug development: Advantages and challenges. *International Journal of Applied and Basic Medical Research* 2(1):3–6.

Gupta, U., and M. Verma. 2013. Placebo in clinical trials. *Perspectives in Clinical Research* 4(1):49–52.

Gurrin, L. C., J. J. Kurinczuk, and P. R. Burton. 2000. Bayesian statistics in medical research: An intuitive alternative to conventional data analysis. *Journal of Evaluation in Clinical Practice* 6(2):193–204.

Hay, M., D. W. Thomas, J. L. Craighead, C. Economides, and J. Rosenthal. 2014. Clinical development success rates for investigational drugs. *Nature Biotechology* 32(1):40–51.

Hayden, E. C. 2014. Ethical dilemma for ebola trials. *Nature* 515:177–178.

Hentoff, N. 1996. Breakthrough for HIV-positive babies. *New York Times*. https://www.washingtonpost.com/archive/opinions/1996/08/05/breakthrough-for-hiv-positive-babies/a3be3f8c-e9d3-4f1b-bcd7-eb457c581d59/?utm_term=.b504cd1631fd (accessed February 20, 2017).

HHS (U.S. Department of Health and Human Services). 2009. 45 CFR 46, *Common rule for the protection of human subjects in research*. https://www.hhs.gov/ohrp/regulations-and-policy/regulations/common-rule (accessed February 20, 2017).

ICH (International Conference on Harmonisation). 2000. *ICH harmonised tripartite guideline: Clinical investigation of medicinal products in the pediatric population - E11*. http://www.ich.org/fileadmin/Public_Web_Site/ICH_Products/Guidelines/Efficacy/E11/Step4/E11_Guideline.pdf (accessed March 7, 2017).

IOM (Institute of Medicine). 1994. *Women and health research: Ethical and legal issues of including women in clinical studies*. Washington, DC: National Academy Press.

Copyright © National Academy of Sciences. All rights reserved.

Integrating Clinical Research into Epidemic Response: The Ebola Experience

IOM. 2004. *The ethical conduct of clinical research involving children*. Washington, DC: The National Academies Press.

Joffe, S. 2014. Evaluating novel therapies during the Ebola epidemic. *JAMA* 312(13):1299–1300.

Johnston, W. R. 2015. *Statistics on the 2014–2015 West Africa Ebola outbreak*. http://www.johnstonsarchive.net/policy/westafrica-ebola.html (accessed January 20, 2017).

KFF (Kaiser Family Foundation). 2014. *Ebola characteristics and comparisons to other infectious diseases*. http://kff.org/infographic/ebola-characteristics-and-comparisons-to-other-infectious-diseases (accessed January 11, 2014).

Kim, J. H., and A. R. Scialli. 2011. Thalidomide: The tragedy of birth defects and the effective treatment of disease. *Toxicological Sciences* 122(1):1–6.

Krugman, S. 1986. The Willowbrook hepatitis studies revisited: Ethical aspects. *Reviews of Infectious Diseases* 8(1):157–162.

Lamontagne, F., C. Clément, T. Fletcher, S. T. Jacob, W. A. I. Fischer, and R. A. Fowler 2014. Doing today's work superbly well—Treating Ebola with current tools. *New England Journal of Medicine* 371(17):1565–1566.

Lane, H. C., H. D. Marston, and A. S. Fauci. 2016. Conducting clinical trials in outbreak settings: Points to consider. *Clinical Trials (London, England)* 13(1):92–95.

Langmuir, A. D. 1979. Guillain-Barré syndrome: The swine influenza virus vaccine incident in the United States of America, 1976–77: Preliminary communication. *Journal of the Royal Society of Medicine* 72(9):660–669.

Largent, E. A. 2016. Ebola and FDA: Reviewing the response to the 2014 outbreak, to find lessons for the future. *Journal of Law and the Biosciences* 3(3):489–537.

Lewis, R. J. 2012. *An overview of Bayesian adaptive clinical trial design* http://www.berryconsultants.com/wp-content/uploads/2012/09/An-Overview-of-Bayesian-Adaptive-Clinical-Trial-Design.pdf (accessed January 18, 2017).

Lipsitch, M., C. A. Donnelly, C. Fraser, I. M. Blake, A. Cori, I. Dorigatti, N. M. Ferguson, T. Garske, H. L. Mills, S. Riley, M. D. Van Kerkhove, and M. A. Hernán. 2015. Potential biases in estimating absolute and relative case-fatality risks during outbreaks. *PLoS Neglected Tropical Diseases* 9(7):e0003846.

London, A. 2017. Equipoise in research: Integrating ethics and science in human research. *JAMA* 317(5):525–526.

Lyerly, A. D., M. O. Little, and R. Faden. 2008. The second wave: Toward responsible inclusion of pregnant women in research. *International Journal of Feminist Approaches to Bioethics* 1(2):5–22.

Mahajan, R., and K. Gupta. 2010. Adaptive design clinical trials: Methodology, challenges and prospect. *Indian Journal of Pharmacology* 42(4):201–207.

McCoy, T. 2014. Why the brutal murder of several Ebola workers may hint at more violence to come. *The Washington Post*, September 19. https://www.washingtonpost.com/news/morning-mix/wp/2014/09/19/why-the-brutal-murder-of-eight-ebola-workers-may-hint-at-more-violence-to-come/?utm_term=.7b84f596959b (accessed January 11, 2017).

McMillan, J., and C. Conlon. 2004. The ethics of research related to health care in developing countries. *Journal of Medical Ethics* 30(2):204–206.

McWhirter, C., P. Loftus, and D. Hinshaw. 2014. Giving Americans drug for Ebola virus prompts flak. *The Wall Street Journal*, August 4, 2014. http://www.wsj.com/articles/second-ebola-patient-lands-in-u-s-1407256243 (accessed January 12, 2017).

Meltzer, M. I., C. Y. Atkins, S. Santibanez, B. Knust, B. W. Petersen, E. D. Ervin, S. T. Nichol, I. K. Damon, and M. L. Washington. 2014. Estimating the future number of cases in the Ebola epidemic— Liberia and Sierra Leone, 2014–2015. *Morbidity and Mortality Weekly Report* 63(03):1–14.

Copyright © National Academy of Sciences. All rights reserved.

Integrating Clinical Research into Epidemic Response: The Ebola Experience

Moisse, K. 2014. *Experimental Ebola drug's role in Americans' recoveries remains unclear*. http://abcnews.go.com/Health/experimental-ebola-drugs-role-americans-recoveries--remains-unclear/story?id=25070069 (accessed December 21, 2016).

Montgomery, A. A., T. J. Peters, and P. Little. 2003. Design, analysis and presentation of factorial randomised controlled trials. *BMC Medical Research Methodology* 3(1):26.

MRC CTU (Medical Research Center Clinical Trials Unit). 2014. *MAMS design: Design of trials, meta-analyses, and observational studies*. http://www.ctu.mrc.ac.uk/our_research/research_types/methodology1/design/mams_design (accessed January 17, 2017)

MSF (Médicins Sans Frontières). 2014a. *Ebola: The failures of the international outbreak response*. http://www.msf.org/en/article/ebola-failures-international-outbreak-response (accessed January 20, 2017).

MSF. 2014b. *Ebola: Treatments and vaccines could help bring outbreak under control*. http://www.msf.org/en/article/ebola-treatments-and-vaccines-could-help-bring-outbreak-under-control (accessed February 20, 2017).

MSF. 2015. *Ebola: Pushed to the limit and beyond: A year into the largest ever Ebola outbreak*. Geneva, Switzerland: MSF. http://www.msf.org/en/article/ebola-pushed-limit-and-beyond (accessed January 17, 2017).

MSF. 2016. *Ebola: How does MSF care for Ebola patients?* https://www.msf.org.uk/ebola-how-does-msf-care-for-ebola-patients (accessed January 20, 2017).

Nason, M. 2016. Statistics and logistics: Design of Ebola vaccine trials in West Africa. *Clinical Trials* 13(1):87–91.

National Commission for the Protection of Human Subjects of Biomedical and Behavioral Research. 1979. *The Belmont report: Ethical principles and guidelines for the protection of human subjects of research*. DHEW Publication No. (OS) 78-0013 and No. (OS) 78-0014. Washington, DC: U.S. Government Printing Office.

Nelson, R. M., M. Roth-Cline, K. Prohaska, E. Cox, L. Borio, and R. Temple. 2015. Right job, wrong tool: A commentary on designing clinical trials for Ebola virus disease. *American Journal of Bioethics* 15(4):33–36.

NIH (National Institutes of Health). 2016. *Glossary of common site terms*. https://clinicaltrials.gov/ct2/about-studies/glossary#active-comparator-arm (accessed January 17, 2017).

Nuffield Council on Bioethics. 2002. *The ethics of research related to healthcare in developing countries*. http://nuffieldbioethics.org/report/research-developing-countries-2/chapter-downloads (accessed January 20, 2017).

Nuffield Council on Bioethics. 2015. *Children and clinical research: Ethical issues*. https://nuffieldbioethics.org/wp-content/uploads/Children-and-clinical-research-full-report.pdf (accessed March 17, 2017).

Nuremberg Code (1947). 1996. *BMJ* 313(7070):1448.

OHRP (U.S. Department of Health and Human Services Office for Human Research Protections). 2016. *Special protections for children as research subjects*. https://www.hhs.gov/ohrp/regulations-and-policy/guidance/special-protections-for-children/index.html (accessed February 17, 2017).

Oxford English Dictionary. 2016. Placebo effect. Oxford, UK: Oxford University Press.

Partners in Health. 2015. *The tiniest Ebola patients*. http://www.pih.org/blog/the-tiniest-ebola-patients (accessed January 20, 2017).

Presidential Commission for the Study of Bioethical Issues. 2015. *Ethics and Ebola public health planning and response*. http://bioethics.gov/sites/default/files/Ethics-and-Ebola_PCSBI_508.pdf (accessed January 20, 2017).

PREVAIL II Writing Group. 2016. A randomized, controlled trial of ZMapp for Ebola virus infection. *New England Journal of Medicine* 375(15):1448-1456.

Proschan, M. A. L., K. K. Gordan, and Janet Turk Wittes. 2006. *Statistical monitoring of clinical trials*. New York: Springer.

Copyright © National Academy of Sciences. All rights reserved.

Qiu, X., G. Wong, J. Audet, A. Bello, L. Fernando, J. B. Alimonti, H. Fausther-Bovendo, H. Wei, J. Aviles, E. Hiatt, A. Johnson, J. Morton, K. Swope, O. Bohorov, N. Bohorova, C. Goodman, D. Kim, M. H. Pauly, J. Velasco, J. Pettitt, G. G. Olinger, K. Whaley, B. Xu, J. E. Strong, L. Zeitlin, and G. P. Kobinger. 2014. Reversion of advanced Ebola virus disease in nonhuman primates with ZMapp. *Nature* 514(7520):47–53.

R2HC (Research for Health in Humanitarian Crises). 2016. *Research for health in humanitarian crises*. http://www.elrha.org/r2hc/home (accessed January 20, 2017).

Rettner, R. 2014. *Ebola "experimental serum" in limited supply, CDC says*. http://www.livescience.com/47224-experimental-ebola-treatment-supply-limit.html (accessed January 20, 2107).

Rid, A., and E. J. Emanuel. 2014. Compassionate use of experimental drugs in the Ebola outbreak—Authors' reply. *The Lancet* 384(9957):1844.

Sacristán, J. A. 2015. Clinical research and medical care: Towards effective and complete integration. *BMC Medical Research Methodology* 15:4.

Schuklenk, U. 2014. *WHO Ebola ethics panel excluded those most affected*. https://theconversation.com/who-ebola-ethics-panel-excluded-those-most-affected-30429 (accessed December 21, 2016).

Seay, L. 2014. Ebola, research ethics, and the ZMapp serum. *Washington Post*, August 6. https://www.washingtonpost.com/news/monkey-cage/wp/2014/08/06/ebola-research-ethics-and-the-zmapp-serum/?utm_term=.826de62c2cd9 (accessed January 11, 2017).

Shields, K. E., and A. D. Lyerly. 2013. Exclusion of pregnant women from industry-sponsored clinical trials. *Obstetrics and Gynecology* 122(5):1077–1081.

Singh, J. A. 2015. Humanitarian access to unapproved interventions in public health emergencies of international concern. *PLoS Medicine* 12(2):e1001793.

Sissoko, D., C. Laouenan, E. Folkesson, A.-B. M'Lebing, A.-H. Beavogui, S. Baize, A.-M. Camara, P. Maes, S. Shepherd, C. Danel, S. Carazo, M. N. Conde, J.-L. Gala, G. Colin, H. Savini, J. A. Bore, F. Le Marcis, F. R. Koundouno, F. Petitjean, M.-C. Lamah, S. Diederich, A. Tounkara, G. Poelart, E. Berbain, J.-M. Dindart, S. Duraffour, A. Lefevre, T. Leno, O. Peyrouset, L. Irenge, N. F. Bangoura, R. Palich, J. Hinzmann, A. Kraus, T. S. Barry, S. Berette, A. Bongono, M. S. Camara, V. Chanfreau Munoz, L. Doumbouya, H. Souley, P. M. Kighoma, F. R. Koundouno, L. Réné, C. M. Loua, V. Massala, K. Moumouni, C. Provost, N. Samake, C. Sekou, A. Soumah, I. Arnould, M. S. Komano, L. Gustin, C. Berutto, D. Camara, F. S. Camara, J. Colpaert, L. Delamou, L. Jansson, E. Kourouma, M. Loua, K. Malme, E. Manfrin, A. Maomou, A. Milinouno, S. Ombelet, A. Y. Sidiboun, I. Verreckt, P. Yombouno, A. Bocquin, C. Carbonnelle, T. Carmoi, P. Frange, S. Mely, V.-K. Nguyen, D. Pannetier, A.-M. Taburet, J.-M. Treluyer, J. Kolie, R. Moh, M. C. Gonzalez, E. Kuisma, B. Liedigk, D. Ngabo, M. Rudolf, R. Thom, R. Kerber, M. Gabriel, A. Di Caro, R. Wölfel, J. Badir, M. Bentahir, Y. Deccache, C. Dumont, J.-F. Durant, K. El Bakkouri, M. Gasasira Uwamahoro, B. Smits, N. Toufik, S. Van Cauwenberghe, K. Ezzedine, E. Dortenzio, L. Pizarro, A. Etienne, J. Guedj, A. Fizet, E. Barte de Sainte Fare, B. Murgue, T. Tran-Minh, C. Rapp, P. Piguet, M. Poncin, B. Draguez, T. Allaford Duverger, S. Barbe, G. Baret, I. Defourny, M. Carroll, H. Raoul, A. Augier, S. P. Eholie, Y. Yazdanpanah, C. Levy-Marchal, A. Antierrens, M. Van Herp, S. Günther, X. de Lamballerie, S. Keïta, F. Mentre, X. Anglaret, D. Malvy, and JIKI Study Group. 2016. Experimental treatment with favipiravir for Ebola virus disease (the JIKI trial): A historically controlled, single-arm proof-of-concept trial in Guinea. *PLoS Medicine* 13(3):e1001967.

Suresh, K. P. 2011. An overview of randomization techniques: An unbiased assessment of outcome in clinical research. *Journal of Human Reproductive Sciences* 4(1):8–11.

Copyright © National Academy of Sciences. All rights reserved.

Temple, R. 2013. *The regulatory pathway for rare diseases lessons learned from examples of clinical study designs for small populations: U.S. Food and Drug Administration.* http://ctsicn.org/sites/default/files/attachments/%234%20Temple.pdf (accessed January 25, 2017).

Thomas, D. W., J. Burns, J. Audette, A. Carrol, C. Dow-Hygelund, and M. Hay. 2015. *Clinical development success rates 2006–2015*. Bend, OR: Amplion. https://www.bio.org/sites/default/files/Clinical%20Development%20Success%20Rates%202006-2015%20-%20BIO,%20Biomedtracker,%20Amplion%202016.pdf (accessed January 25, 2017).

UNESCO (United Nations Educational, Scientific, and Cultural Organization). 2006. *Universal declaration on bioethics and human rights*. Paris, France: UNESCO. http://unesdoc.unesco.org/images/0014/001461/146180E.pdf (accessed January 25, 2017).

Uyeki, T. M., A. K. Mehta, R. T. J. Davey, A. M. Liddell, T. Wolf, P. Vetter, S. Schmiedel, T. Grünewald, M. Jacobs, J. R. Arribas, L. Evans, A. L. Hewlett, A. B. Brantsaeter, G. Ippolito, C. Rapp, A. I. M. Hoepelman, and J. Gutman. 2016. Clinical management of Ebola virus disease in the United States and Europe. *New England Journal of Medicine* 374(7):636–646.

van Griensven, J., T. Edwards, X. de Lamballerie, M. G. Semple, P. Gallian, S. Baize, P. W. Horby, H. Raoul, N. F. Magassouba, A. Antierens, C. Lomas, O. Faye, A. A. Sall, K. Fransen, J. Buyze, R. Ravinetto, P. Tiberghien, Y. Claeys, M. De Crop, L. Lynen, E. I. Bah, P. G. Smith, A. Delamou, A. De Weggheleire, and N. Haba. 2016. Evaluation of convalescent plasma for Ebola virus disease in Guinea. *New England Journal of Medicine* 374(1):33–42.

Veatch, R. M. 2007. The irrelevance of equipoise. *Journal of Medicine and Philosophy* 32(2):167–183.

Vickers, A. J., and A. J. de Craen. 2000. Why use placebos in clinical trials? A narrative review of the methodological literature. *Journal of Clinical Epidemio*logy 53(2):157–161.

Wahl, B. 2014. *It's time to make experimental drugs available where people really need them*. https://qz.com/247088/its-time-to-make-experimental-drugs-available-where-people-really-need-them (accessed January 17, 2017).

Wainberg, M. A., S. Kippax, M. Bras, and P. S. Sow. 2014. HIV and Ebola: Similarities and differences. *Journal of the International AIDS Society* 17(1):19896.

Waldman, R., and P. Nieburg. 2015. Thoughts on alternative designs for clinical trials for Ebola treatment research. *American Journal of Bioethics* 15(4):38–40.

Wellcome Trust. 2013. *Fund launched to support research for health in humanitarian crises*. https://wellcome.ac.uk/press-release/fund-launched-support-research-health-humanitarian-crises (accessed January 20, 2017).

WHO (World Health Organization). 2009. *Research ethics in international epidemic response: WHO technical consultation*. Geneva, Switzerland: WHO Press. http://www.who.int/ethics/gip_research_ethics_.pdf (accessed January 25, 2017).

WHO. 2014a. *Ethical considerations for use of unregistered interventions for Ebola viral disease: Report of an advisory panel to WHO*. Geneva, Switzerland: WHO Press. http://apps.who.int/iris/bitstream/10665/130997/1/WHO_HIS_KER_GHE_14.1_eng.pdf?ua=1 (accessed January 17, 2017).

WHO. 2014b. *Ethical issues related to study design for trials on therapeutics for Ebola virus disease*. http://apps.who.int/iris/bitstream/10665/137509/1/WHO_HIS_KER_GHE_14.2_eng.pdf?ua=1 (accessed February 20, 2017).

WHO. 2014c. Safety of immunization during pregnancy: A review of the evidence. Geneva, Switzerland: WHO.

WHO. 2014d. *WHO meeting of the Scientific and Technical Advisory Committee on Ebola Experimental Interventions—Briefing note*. http://www.who.int/medicines/ebola-treatment/scientific_tech_meeting/en (accessed December 21, 2016).

Copyright © National Academy of Sciences. All rights reserved.

Integrating Clinical Research into Epidemic Response: The Ebola Experience

WHO. 2015. *Compassionate use of experimental treatments for Ebola virus disease: Outcomes in 14 patients admitted from August to November 2014*. http://www.who.int/medicines/ebola-treatment/outcomes_experimental_therapies/en (accessed January 20, 2017).

WHO Ebola Response Team. 2014. Ebola virus disease in West Africa—The first 9 months of the epidemic and forward projections. *New England Journal of Medicine* 371(16): 1481–1495.

Wilson, B., J. B. Cooper, J. Lysander, K. Bility, J. D. Anderson, J. Endee, H. Kiawu, L. Doepel, and E. Higgs. 2016 (unpublished). *Engaging communities in clinical research during a public health emergency: An ethical norm*.

WMA (World Medical Association). 2013. World Medical Association Declaration of Helsinki: Ethical principles for medical research involving human subjects. *JAMA* 310(20): 2191–2194.

Copyright © National Academy of Sciences. All rights reserved.

3

Assessment of Therapeutic Trials

After the discussions about clinical trial design (as presented in Chapter 2), scientific and ethics committees convened by the World Health Organization (WHO) published their findings regarding suggested research designs. For example, the Scientific and Technical Advisory Committee on Ebola Experimental Interventions found at their meeting in Geneva on November 11–12, 2014, that “it was likely that for anti-Ebola treatments that did not have large effects, randomized concurrently controlled trials may be needed” (WHO, 2014b). The WHO Ethics Working Group (convened on October 20–21, 2014) also noted the pros and cons of various designs. For example, they noted that single-arm studies that use nonrandomized retrospective control data have “a high risk of bias and may lack internal validity” (WHO, 2014a). However, despite their concerns, the Ethics Working Group concluded, “In principle, so long as standard requirements for human research ethics are met, all scientifically recognized methodologies and study designs should be considered as ethically acceptable—whether they are placebo-controlled randomized trials or trials that don’t involve randomization to control groups” (WHO, 2014a). The group added that the reality of the situation—for example, the scarcity of health care providers, research staff, infrastructure, and other resources—should be taken into account in making design decisions (WHO, 2014a).

Ultimately, formal clinical trials were conducted on five investigational therapeutic agents in the three countries most affected by the epidemic. The five therapeutic agents were

Copyright © National Academy of Sciences. All rights reserved.

1. Favipiravir, developed by Fuji/Toyama (Japan) for pandemic influenza (repurposed);
2. Brincidofovir, Chimerix (United States), developed and used for treatment of cytomegalovirus (repurposed);
3. TKM-130803,[1] developed by Tekmira (Canada);
4. Convalescent plasma; and
5. ZMapp, developed by MappBio (United States).

Preparation and planning for the trials started in September 2014, and the trials began enrolling participants between December 2014 and March 2015. While the trials were launched rapidly, most began participant enrollment at the tail end of the epidemic (see Table 3-1 and Figure 3-1; see also Table 3-2 for details on the preclinical and, if available, clinical data on investigational Ebola agents as of October 2015–before the launch of the trials).

JIKI Trial: Favipiravir

The French institut national de la santé et de la recherche médicale (Inserm) funded a study of favipiravir (MSF, 2015), a repurposed medicinal product that was originally developed for pandemic influenza virus infection (Furuta et al., 2013). The trial was conducted at four Ebola treatment units (ETUs) in Guinea that were operated by four different organizations: at Guéckédou, by Médecins Sans Frontières (MSF); at Nzerekore, by the Alliance for International Medical Action; at Macenta, by the French Red Cross; and at Conkary, by the French military health service (Sissoko et al., 2016). The study was designed to rapidly gather standardized preliminary data about favipiravir in order to guide further research.

Study Design

The trial was designed as a multicenter, single-arm, proof-of-concept trial. Initially, the plan was to use historical data to establish target success rates, but as the trial began, information became available from a patient database in Guinea, so these data were used instead of gathering data de novo during the epidemic (Sissoko et al., 2016). The trial team opted for

[1] TKM-130803 is a new formulation of TKM-100802, one of the lead experimental agents prioritized by WHO. "TKM-100802 has been administered to five patients with Ebola medically evacuated to the US and Europe, and to one individual as post-exposure prophylaxis (personal communication, Mark Kowalski, Tekmira Pharmaceuticals). Since the product was administered on a compassionate basis to these individuals and because the patients simultaneously received other experimental products, it has not been possible to assess the efficacy or safety of TKM-100802 in the treatment of [Ebola]" (Dunning et al., 2016b).

Copyright © National Academy of Sciences. All rights reserved.

TABLE 3-1 Timeline of Therapeutic Trials

Trial Name (investigational agent)	Preparation and Planning	Trial Enrollment Start	Trial Enrollment End	No. of Patients Enrolled	Country
JIKI (Favipiravir)	September–December 2014	December 2014	April 2015	126 patients	Guinea
RAPIDE-BCV (Brincidofovir)	September 2014–January 2015	January 2015	February 2015	4 patients	Liberia
TKM-Ebola (TKM-130803)	September 2014–January 2015	March 2015	June 2015	14 patients	Sierra Leone
Ebola Tx (Convalescent plasma)	November 2014–January 2015	February 2015	August 2015	99 patients	Guinea
PREVAIL II (ZMapp)	September 2014–February 2015*	March 2015	November 2015	72 patients	Guinea, Liberia, Sierra Leone, United States

*A sufficient supply of ZMapp was available in September 2015 (Dodd et al., 2016).

Copyright © National Academy of Sciences. All rights reserved.

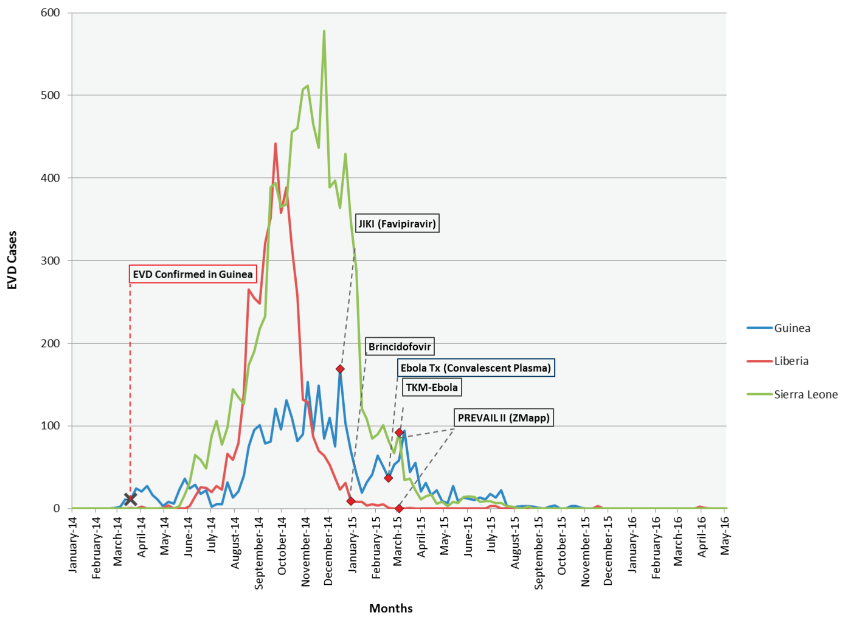

FIGURE 3-1 Clinical trial enrollment dates during the 2014–2015 Ebola outbreak—therapeutic trials. The above figure plots the trial start dates on the time course of the Ebola outbreak (confirmed cases) in each of the respective countries (Guinea, Liberia, and Sierra Leone) where the trials were conducted. The confirmed Ebola case number was obtained from the WHO incident reports.
SOURCES: WHO, 2014a, 2016a,b,c.

a nonrandomized design for two main reasons. First, since Ebola strikes in clusters, the team felt that it was "ethically unacceptable to randomize patients from within the same family or village, who appear together to seek care, to receive or not receive an experimental drug" (Sissoko et al., 2016). Second, investigators, noting the already-existing fear and distrust in the community, worried that a randomized design might exacerbate these tensions and make patients more reluctant to seek care.

Advantages and Disadvantages

The multicenter approach allowed for a large number of participants—in fact, this was the largest Ebola treatment trial and the first to be conducted during the epidemic. Assessment of viral load permitted stratification of patients into risk groups. The rapid initiation of this trial meant that this

Copyright © National Academy of Sciences. All rights reserved.

early experience could potentially inform later efforts: The investigators remarked that the conduct of this trial resulted in lessons learned about how to "quickly set up and run an Ebola trial, in close relationship with the community and nongovernmental organizations," and they learned how to integrate "research into care so that it improved care" (Sissoko et al., 2016).

Results and Discussion

Between December 2014 and April 2015, 126 participants—children, adolescents, and adults—were enrolled. Subsequently, 15 were excluded from the final analysis, 10 because they had received convalescent plasma in another treatment center prior to enrollment in the trial and 5 because they had no available polymerase chain reaction (PCR) data on virus load available at baseline and could not be classified according to the revised stratification (Sissoko et al., 2016). The trial was inconclusive about the efficacy and tolerance of favipiravir in Ebola patients; the investigators noted that their data on tolerance were encouraging but could not be conclusive due to the lack of randomization. However, the study did provide some new information on biomarkers for evaluating patient prognosis and on the course of the disease. For example, investigators found that PCR cycle threshold (C_t)[2] was predictive of patient outcome and served as an effective surrogate of viral load; they suggested that future drug trials should systematically stratify analyses by viral load at baseline C_t value in a semiquantitative Ebola virus reverse transcription (a method the PREVAIL II trial team also used [PREVAIL II Writing Group, 2016]). They also suggested that favipiravir monotherapy "merits further study in patients with medium to high viremia, but not in those with very high viremia" (Sissoko et al., 2016). They reported that a nonsignificant trend in the subgroup of patients with lower viral load might make randomization in a future trial of favipiravir difficult, as the suggestion of possible benefit may limit willingness to allow randomization to an alternative regimen or control group.

Interim data from this trial were released in February 2015 (MSF, 2015); because these data suggested possible benefit, the government of Guinea expanded the use of favipiravir in ETUs (Reuters, 2015). The committee is concerned that the release of interim results could have inappropriately influenced other ongoing clinical trials or caregivers. For example, the coordinator for France's response to Ebola during the outbreak stated that despite concerns over randomizing patients, they would consider sup-

[2] Lower C_t values indicate high amounts of targeted nucleic acid, while higher C_t values mean lower (and even too little) amounts of the targeted nucleic acid.

Copyright © National Academy of Sciences. All rights reserved.

TABLE 3-2 Available Data on Investigational Therapeutic Agents for Ebola When Trials Were Being Planned (November 2014)

Therapeutic Candidate<br>Trial Name<br>(Producer)	Drug Type	Preclinical Evidence	Early Clinical Evidence/<br>Known Safety Issues	Availability and Logistical Considerations (as of November 2014)
Favipiravir<br>JIKI<br>(Fuji/Toyama, Japan)	Small molecule antiviral with activity against many RNA viruses.<br>Functions through inhibiting viral RNA-dependent RNA polymerase. Approved in Japan for treating novel/pandemic influenza.	In vitro inhibition IC50 64 µM; higher than that needed for influenza.<br>Mice: protected at 300 mg/kg.<br>Nonhuman primate (NHP): antiviral effect seen; 2 log reduction in viremia. Model limitation due to frequent need to anesthetize NHP to administer drug orally.	Clinical use in healthy volunteers up to 3.6 g on first day followed by 800 mg twice daily. No safety issues identified.<br>Increased drug exposure in setting of hepatic dysfunction.	200 mg tablets; dosing at 6 g/first day requires 30 tablets—potentially difficult to swallow.<br>1.6 million tablets available free (10,000 treatment courses).<br>Thermostable.
Brincidofovir<br>RAPIDE-BCV<br>(Chimerix, USA)	Small molecule antiviral with activity against dsDNA viruses.<br>Developed and used for treatment of CMV. In theory, should not work on Ebola (RNA virus), mode of action may be different to that for DNA viruses.	In vitro EC50 varies by assay from 120 nM to 1.3 µM. Thought to be a concentration readily achieved in clinic.<br>Selectivity index variable depending on assay.<br>Mice: no therapeutic benefit seen in two separate studies, but no pharmacokinetics (PK); therefore, not known if effective concentration reached.	Testing in >1,000 patients: main symptom GI tolerability, and liver enzymes AST/ALT Elevations.	PO drug. Twice weekly dosing after initial load.<br>22,000×100 mg tablets (>3 500 treatment courses) available.<br>Thermostable.

Copyright © National Academy of Sciences. All rights reserved.

		NHP: Rhesus macaque—not feasible due to PK profile. Guinea pig: study planned to determine PK and efficacy.		
TKM-100802 RAPIDE-TKM; TKM-Ebola (Tekmira, Canada)	Small inhibitory RNA which catalytically cleaves Ebola RNA once inside the cell. Sequence-specific to this strain of Ebola.	NHP: 67–100% (7 rhesus–macaques challenged) efficacy among NHP given 4 to 7 doses with treatment initiated 30 minutes post-challenge (Geisbert et al., 2010).	A Phase 1 safety study found dose-related side effects including dizziness, chest tightness, raised heart rate. A lower dose was better tolerated. A study in healthy volunteers is on partial clinical hold.	Several hundred doses currently available. Several thousand doses could be available in short time period.<br><br>IV infusion.<br><br>Requires refrigeration.
ZMapp PREVAIL II (MappBio, USA)	Cocktail of three monoclonal antibodies produced in tobacco plants.	NHP: 100% survival (18 rhesus macaques) when administered 5 days after virus challenge (Qiu et al., 2014).	Phase 1 safety/PK study conducted in January 2015.	Supply reported to be around 150 treatment courses with another 100 being produced.

SOURCE: This chart is adapted from the WHO's chart "Categorization and Prioritization of Drugs for Consideration for Testing or Use in Patients Infected with Ebola" (WHO, 2015a) and provides a summary of the available Ebola treatments that were under evaluation in formal clinical trials in West Africa as of October 2015. The WHO R&D Landscape (WHO, 2015b) captures a more detailed look at additional investigational therapeutics used during the outbreak, both in formal clinical trials as well as those in compassionate use, historical observational studies, and others that lack sufficient protocol details.
SOURCE: WHO, 2015a.

Copyright © National Academy of Sciences. All rights reserved.

porting the ZMapp trial but "perhaps the standard of care should include favipiravir as part of the control arm in studies of ZMapp and other experimental treatments" (Cohen, 2015). In fact, favipiravir was included as part of optimized standard of care in Guinea for the PREVAIL trial (Davey, 2016), and in June 2016 the Guinean government "formally adopted the administration [of favipriravir] as a part of the standard treatment for [Ebola]" (FujiFilm Corporation, 2016), despite the lack of reliable evidence of efficacy. The JIKI trial experience illustrates the increased risks for biased assessments and prejudgments about interim data occurring when single-arm trials are conducted and when there is early public access to unreliable interim results.

This trial, coupled with the large data set of N >500 patients treated in Guinea early in the course of the outbreak that became available just before the trial's launch, identified important prognostic factors, thus adding value to the evidence base. However, based on the available evidence at this time, there is no conclusive evidence that the drug had a beneficial effect, and therefore favipiravir will still require further evaluation in a randomized controlled trial (RCT) during a future outbreak to resolve the question of efficacy.

Rapid Assessment of Potential Interventions and Drugs for Ebola: TKM-Ebola (TKM-130803)

The Rapid Assessment of Potential Interventions and Drugs for Ebola (RAPIDE) trials, led by investigators from the University of Oxford and the International Severe Acute Respiratory and Emerging Infection Consortium, investigated two agents, brincidofovir and TKM-Ebola, in two separate trials of similar design (Kroll, 2015; Wellcome Trust, 2015). Brincidofovir was prioritized for Ebola trials because of its oral bioavailability and its known safety in seriously ill patients, in addition to its being stable at room temperature and not requiring cold storage (Haque et al., 2015). The brincidofovir study began in Liberia in January 2015, but was terminated after enrolling only four patients due to changing priorities of the drug sponsor as well as the waning of the epidemic (Chimerix Inc., 2015; Dunning et al., 2016a). This terminated trial is probably most valuable as an example of how commercial and other nonhumanitarian considerations can be barriers to successful evaluation of a new treatment in a challenging setting. The TKM-Ebola (TKM-130803) trial was launched in Sierra Leone at an ETU operated by GOAL Global, an Irish nongovernmental organization, and ran from March to June 2015 (Dunning et al., 2016b; Wellcome Trust, 2015).

Copyright © National Academy of Sciences. All rights reserved.

Study Design

The trial used a multistage design (Dunning et al., 2016b). The first stage, in which 100 patients would be evaluated, was a single-arm sequential design with three possible decisions: the treatment is effective, promising, or not promising. A survival probability of 50 percent or less was defined as "not promising." The design had 99 percent power to conclude that a survival probability of 80 percent was effective or promising, with a Type I error rate of 10 percent if the survival proportion was 0.50 (Whitehead et al., 2016). If the treatment was determined to be either effective or promising at the end of Stage 1, it would be subjected to further evaluation: a confirmatory single-arm study if determined effective, or a randomized controlled trial if determined promising. These additional stages provided some protection against both false positive and false negative results; in reality, however, it may have been difficult to do a controlled study of a drug yielding a promising result in Stage 1 as there likely would have been pressure to provide such a drug to everyone if it was not in limited supply. This design was similar to the common approach to drug development for solid tumors, in which a small single-arm Phase 2 trial that sees a response rate greater than a prespecified threshold is followed by a larger randomized Phase 3 trial with a standard treatment comparator (Horby, 2015).

The choice of design was influenced by two factors: (1) a desire to quickly identify highly effective or clearly ineffective treatments due to the high death rate and volatile conditions of the epidemic and (2) a desire to avoid randomization, unless necessary, because of a perception that randomization might not be acceptable or would not be feasible in the setting of a trial. On days that the capacity for trial enrollment was reached, additional patients were to be enrolled into a concurrent observational cohort. This practical approach may have been more acceptable to the community than other approaches to randomization. Patients who died within 48 hours were excluded from the analysis as they were assumed to have been too sick to be potentially responsive to the treatment. A futility bound was established to allow for termination after a small number of patients if the treatment did not appear to be promising, protecting future patients from being exposed to any risks of an ineffective treatment.

Advantages and Disadvantages

The overall strategy of the trial—starting with a single-arm stage and proceeding to a randomized stage if intermediate results were neither clearly positive nor clearly negative—had reasonable operating characteristics (low Type I error rate and high power to identify highly effective treatments). The initial single-arm phase of the trial was likely easier to initiate than a

Copyright © National Academy of Sciences. All rights reserved.

randomized design, as it was simpler to explain to patients and health care workers who had to administer only a single treatment regimen. However, the overall trial strategy may have been difficult to fully implement in an outbreak environment. Once a treatment is labeled as "promising" in this first stage, caregivers and researchers may be inclined to resist randomizing patients to a standard of care arm, as required by the next stage in the testing strategy.

Results and Discussion

After 14 patients had been treated, the study was terminated because 11 of the 14 patients died, an outcome that was inconsistent with a true survival rate of 50 percent or greater (Dunning et al., 2016b). Unfortunately, without a control group and with the small number of patients involved it is difficult to draw strong conclusions from this experience. The trial used historical controls to define a target probability of survival at 14 days greater than 55 percent as promising (and anything less as futile). The selection of this target stemmed from an analysis of individual-level data on 1,820 adult patients with PCR-confirmed Ebola virus infection from earlier in the outbreak. A major problem with this approach was the lack of data from this population on key prognostic factors (e.g., viral load) to stratify the probability of survival. Other reports have suggested that the probability of survival for patients with high viral load might have been closer to 0.10, as opposed to the original estimate of 0.27. "The probability that a TKM-130803 recipient who survived for 48 h will subsequently survive to day 14 was estimated to be 0.27 (95 percent CI = 0.06–0.58)" (Dunning et al., 2016b). Because no estimates of viral load in earlier patients were available, it cannot be determined if the patients in the trial were more or less severely ill than those treated in the past. In addition, given changes in supportive care over time, these historical controls may not have been a relevant comparator.

Ebola-Tx: Convalescent Plasma

The European Union funded the Ebola-Tx project to investigate the safety and efficacy of convalescent plasma (CP) (ITM, 2016). Previous preclinical evidence supported the use of CP; it was shown that nonhuman primates who were challenged with filoviruses survived after being treated with antibodies from previously exposed primates (Dye et al., 2012). The provision of CP is used to achieve short-term immunization, termed passive immunization (PI), against a pathogen through administering pathogen-specific antibodies present in the survivor's plasma. "Although antibiotics

Copyright © National Academy of Sciences. All rights reserved.

have largely supplanted the use of PI in bacterial infections, it remains an important tool in the treatment of many viral infections when vaccines or other specific treatments are not available" (Marano et al., 2016). The Ebola-Tx project was led by the Institute of Tropical Medicine in Antwerp and was conducted at MSF's Donka ETU in Conakry, Guinea (ITM, 2016). The trial was initiated in February 2015. Patient enrollment was stopped in early July 2015, on the advice of the independent Data Safety and Monitoring Board, primarily because the outbreak had slowed in Conakry (ITM, 2016).

Study Design

The trial was a nonrandomized, open-label (nonblinded) study that used patients who had been admitted to the same ETU prior to the start of the study as historical controls. The trial initially planned for a concurrent standard-of-care control arm in the event that there was a shortage of CP; however, a shortage never materialized (Edwards et al., 2016). The trial team made the decision not to randomize patients because they believed it would not be acceptable to patients or health care workers, given the volatile epidemic and high mortality rate, and because it would mean withholding a potentially lifesaving treatment from patients (Adebamowo et al., 2014). For future trials, however, the trial team suggested that "in-depth anthropological studies should also be conducted to gain a better understanding of community acceptability of randomization during outbreaks of diseases with high case fatality rates" (Edwards et al., 2016, p. 20).

Advantages and Disadvantages

The trial had broad entry criteria, enrolling men and women of all ages, including pregnant women, unless CP was contraindicated. These criteria made it possible to enroll 102 patients, of whom 99 were assigned to receive CP. Of the 102 enrolled, 18 were excluded from analysis (3 died before completion of eligibility assessments, 4 died before the third day of diagnosis, 10 received favipiravir as well, and 1 did not have the required PCR cycle-threshold value), resulting in 84 patients in the primary analysis (van Griensven et al., 2016a). Those who received another treatment were excluded from analysis, potentially introducing a bias since these subjects had to survive long enough to get another treatment and excluding them would lead to an underestimation of the average survival time. In terms of the delivery of treatment and explanations to patients, the trial was also relatively straightforward for the clinic to conduct.

Copyright © National Academy of Sciences. All rights reserved.

Results and Discussion

The study found that the transfusion of up to 500 ml of CP, with unknown levels of neutralizing antibodies, in 84 patients with confirmed Ebola was not associated with a significant improvement in survival compared with historical controls (van Griensven et al., 2016a). The results are difficult to interpret in the absence of both randomization and a concurrent control arm, and the fact that antibody titers and evidence of virus neutralization are unknown. It is worth noting that the CP may have actually had a modest to moderate effect in patient outcome; it is the lack of a control group that makes it impossible to identify anything other than a very large effect with reasonable confidence. Because of logistical challenges, the antibody levels in the plasma were not evaluated before administration. Ideally, the donor CP would have been screened for antibody levels, and the plasma with the highest levels would have been used for transfusion. Alternatively, the data could have been stratified on the basis of the neutralizing antibody titer of the administered plasma sample before analysis. The serious consequence of this study design is that the inability of the trial to identify a significant but moderate efficacy may result in a rejection of CP as a treatment, despite the possibility that plasma, especially with a sufficiently high antibody level, may be effective. The most important findings that can be drawn from the study are that treatment with convalescent plasma appears to be safe and that the treatment appeared to have a high level of feasibility and acceptability in the midst of an outbreak.

However, caution should be used in the use of passive immunization due to the potential for "antibodies to enhance viral infections via antibody-dependent enhancement mechanisms" (van Griensven et al., 2016a). This increase in infectivity has been observed in vitro for both Ebola virus and Marburg virus as well as for other viruses, including HIV (Beck et al., 2008; Nakayama et al., 2011).

Partnership for Research on Ebola Vaccines in Liberia (PREVAIL) II–ZMapp

The PREVAIL II trial to investigate the use of ZMapp in the treatment of Ebola was sponsored by the U.S. National Institutes of Health and involved the ministries of health of Guinea, Liberia, and Sierra Leone, along with Mapp Biopharmaceuticals, Inserm, and academic medical centers in the United States. It was conducted in Guinea, Liberia, Sierra Leone, and the United States between March and November 2015 (PREVAIL II Writing Group, 2016).

Copyright © National Academy of Sciences. All rights reserved.

Study Design

The trial was a Phase 1/2, multicenter, randomized, open-label trial. The initial stage of the trial consisted of two arms: ZMapp plus optimized standard of care versus optimized standard of care only. Patients were randomized in a 1:1 ratio to the two groups. ZMapp was given in three intravenous infusions (50 mg per kilogram of body weight) 3 days apart, and optimized standard of care included the provision of intravenous fluids, balancing electrolytes, maintaining oxygen status and blood pressure, and treating concurrent infections. If an investigational treatment were proven to be superior to optimized standard of care alone with respect to survival, it would then become the basis of the new standard of care against which additional investigational Ebola interventions could be tested and compared. The trial also incorporated frequent interim monitoring by an independent data and safety monitoring board to facilitate the early elimination of poorly performing treatments and the introduction of new candidate therapies without influencing those conducting the trial and treating patients. The plan was for each experimental therapy to be studied in up to 100 participants per arm. If investigators were unable to establish a significant benefit of the therapy over optimized standard of care after enrolling 100 participants per arm, then that particular treatment would be declared ineffective, and investigators would begin testing the next therapy.

Advantages and Disadvantages

The antibody combination and dose selection for ZMapp were predicated on strong translational evidence from nonhuman primate studies (Qiu et al., 2014). The clinical study was not blinded because of the burden and potential harm of administering placebo infusions to Ebola patients and because the study outcomes of primary interest—mortality and viral load—were thought to be less susceptible to bias. However, the lack of blinding could have resulted in some bias in interpreting clinical response and adverse events. The use of randomization allowed for an appropriate comparator to assess the safety and efficacy of ZMapp (and other novel interventions that might have been studied later). The trial stratified patients to control for presumed differences in prognosis based on baseline viral burden as well as in potential differences in optimized standard of care based on location. The trial used an innovative barely Bayesian-type design that was more permissive of termination for efficacy or futility than some other standard approaches, without undermining the control of Type I error (Dodd et al., 2016). The study protocol was designed to be adaptive; it included a series

Copyright © National Academy of Sciences. All rights reserved.

of two-arm comparisons of novel interventions (the first being ZMapp) compared with optimized standard of care to establish a framework that could be used to evaluate multiple potential Ebola treatments in the future. The design could be extended to multiple arms if multiple treatment options were simultaneously available and seemed equally promising.

Results and Discussion

The trial enrolled 72 adults and children with confirmed Ebola infection from Guinea (12 patients), Liberia (5 patients), Sierra Leone (54 patients), and the United States (1 patient); the trial was stopped after 72 of the intended 200 patients were enrolled, due to the winding down of the epidemic. In general, those who received ZMapp appeared to do better, regardless of virus levels, but the results were not statistically significant. The observed posterior probability that ZMapp plus the current standard of care was superior to the current standard of care alone was 91.2 percent, falling short of the prespecified threshold of 97.5 percent. Frequentist analyses yielded similar results (absolute difference in mortality with ZMapp, –15 percentage points; 95 percent confidence interval, –36 to 7). From a safety standpoint, ZMapp appeared to be well tolerated. ZMapp showed promise as a possible effective treatment for Ebola, but the data were insufficient to determine definitively whether it is superior to supportive care alone. Although only 72 patients were enrolled, being the only randomized trial of a therapeutic intervention conducted during the outbreak, it added valuable information on the effects of ZMapp on Ebola (PREVAIL II Writing Group, 2016). Prior to PREVAIL, only animal model and nonhuman primate data existed for ZMapp, but the conduct of this trial has provided important safety data and efficacy data in humans showing a trend toward a ~40 percent reduction in mortality.

DISCUSSION

The end result of the therapeutic trials was a “thin scientific harvest” (Cohen and Enserink, 2016) (see Table 3-3 at the end of the chapter for a summary of the therapeutic trials). Because the epidemic began to wane as the trials were being planned in the fall of 2014, most of the trials were unable to enroll enough patients to meet the desired targets. Due to the problem with sample size, none of the therapeutic trials were able to reach definitive conclusions about treatment efficacy. However, even if the trials had been able to enroll to completion, it is highly unlikely that the single-arm studies would have provided conclusive evidence on the effectiveness of the agents in the absence of concurrent controls. Given the limited pre-

Copyright © National Academy of Sciences. All rights reserved.

clinical evidence available on the safety and efficacy of the investigational medicinal products, the changing standard of care for Ebola patients, and variable mortality rates in different settings and population subgroups, the case for randomization providing the most robust evidence was strong, and the committee concludes that randomization should have been more widely used. PREVAIL II demonstrated that an RCT was acceptable in all three countries, despite the doubts expressed earlier in the epidemic; this is in large part due to the evolving circumstances on the ground and the social mobilization efforts made by the research team (see Chapter 6 for more detailed discussion on community engagement). ZMapp, initially hoped to be a highly efficacious therapeutic agent for treating Ebola, did not live up to the publicity, although the limited evidence suggests it might have some benefit, even if it is less than uniformly effective. The investigators concluded that "in the event of another outbreak, that experimental niche should probably be filled by one of a small number of other promising, but unproven, treatments that have emerged since the beginning of the recent crisis" (PREVAIL II Writing Group, 2016, p. 1455). However, it should be noted that PREVAIL II successfully used an adaptive randomized, controlled trial design that could facilitate future trials.

Our understanding of treatment options for Ebola is little better than it was before the outbreak due to the fact that none of the trials yielded conclusive results. There is a legitimate concern that inconclusive trials may actually set back the search for an effective therapy. Single-arm trials may have missed moderate and clearly worthwhile effects and thus discounted a potentially beneficial product for future study. Trials that released preliminary results suggesting the experimental intervention was effective may have contributed to perceptions that overestimated the potential benefits, thereby compromising the ability to perform future controlled trials of these products.

Aside from compromising the ability to conduct a future clinical trial, the adoption of investigational medicinal products (or practices) based on inconclusive or preliminary evidence may lead to medical care that is ineffective or even potentially harmful. In many cases, accepted medical practice (therapies and diagnostics) established without the basis of solid evidence from RCTs may be found to be without value when RCTs are eventually conducted (Prasad et al., 2013). While this "phenomenon should be rare in the age of evidence-based medicine, it is ubiquitous" (Prasad and Cifu, 2011, p. 472). For example, hormone replacement therapy was widely used to prevent cardiovascular disease on the basis of nonrandomized evidence before randomized trials showed that such treatment was more likely harmful than beneficial (ACOG, 2013; Writing Group for the Women's Health Initiative, 2002). These medical reversals can have seri-

Copyright © National Academy of Sciences. All rights reserved.

ous implications, not just regarding suboptimal care for patients but also regarding a loss of patient trust in the medical system (Prasad et al., 2012). For a disease like Ebola, the potential consequences of promoting an ineffective medical practice can be even more severe. The efficacy of a treatment for Ebola can only be tested during an outbreak, so reversing a perceived benefit would require a repetition of the trial during another outbreak; it is clearly better to get it right in the first place with the right design. In addition, Ebola strikes in countries where trust in the government and authority, including the medical system and health care providers, is already low and where research may not be well understood, which results in a situation in which reversing a common practice (e.g., reversing the decision to include favipiravir as standard of care in Guinea) would risk being perceived as even more suspect.

One of the major goals of conducting clinical research is to generate sufficient evidence to lead to product approval. Early consultations with regulators may help researchers select agents for study and develop trial designs that would generate reliable information with the potential to lead to regulatory approval. Outside the U.S. Food and Drug Administration's (FDA's) Investigational New Drug program (FDA, 2016), researchers in the United States and Europe are not required to consult with their regulators, but such consultations for both new investigational drugs and repurposed medicinal products may be beneficial as regulators very often have access to proprietary information that others do not and can use their discretion to inform researchers in a way that can save effort and direct resources to best use. Consultations may take time, but in urgent situations such as the Ebola outbreak, regulators have shown they can be very supportive and responsive in the context of the epidemic. Further, some delay on the front end may result in shorter approval time down the road and provide access to more people more quickly. As was recognized by the regulators involved in the Ebola outbreak, it is essential that regulatory bodies in affected countries are included in these conversations as early as possible.[3]

Regulators in the United States and Europe also have mechanisms for expedited review that can speed up the review timeline. These regulations strongly advise sponsors participate in early and frequent dialogue with regulators (EMA, 2005; HHS, 2014). The FDA, for example, has four main programs "intended to facilitate and expedite development and review of new drugs to address unmet medical need in the treatment of a serious or life-threatening condition"; these are fast track, breakthrough therapy,

[3] Testimony by Robert Hemmings, Medicines and Healthcare Products Regulatory Agency (MHRA) UK; Peter Marks, U.S. FDA; Edward M. Cox, U.S. FDA; and Marco Cavaleri, European Medicine Agency (EMA). Public Webinar of the Committee on Clinical Trials During the 2014–2015 Ebola Outbreak, May 19, 2016.

Copyright © National Academy of Sciences. All rights reserved.

accelerated approval, and priority review designation (HHS, 2014). Products can qualify for one or more of these programs depending on the qualifying criteria. During the Ebola outbreak, both TKM-Ebola and ZMapp were given fast track review. Additionally, regulators may aid in selecting products for investigation if the available preclinical evidence is based on animal models. Animal models can help to prioritize the agents most likely to be efficacious, but only if there are good animal models for the medical condition. In rare cases, efficacy in animals might support licensure of a product under the Animal Efficacy Rule rule; however, the animal rule is only applicable when there are validated animal models for the disease (HHS, 2015). Even with the animal rule, researchers would still have to conduct Phase 1 and Phase 2 trials to obtain sufficient efficacy and safety data in humans to determine safety.

> ***Conclusion 3-1*** *Product regulators can play a useful role in providing advice about trial design and selection of agents to study, and they should be involved in deliberations about these decisions in future epidemic situations.*

Copyright © National Academy of Sciences. All rights reserved.

TABLE 3-3 Summary of Therapeutic Trials Conducted During 2014–2015 Ebola Outbreak

Investigational Product	Sponsoring Organization, Trial Name	Trial Location	Trial Design and Design Considerations	Timeline	Results
Convalescent plasma (CP)<br><br>(ITM, 2016; van Griensven et al., 2016a)	Institute of Tropical Medicine, Belgium; Ebola Tx	Guinea	Trial Design<br>- Safety and efficacy of CP for Ebola in Guinea<br>- Nonrandom, open-label<br>- Historical controls—Control SOC arm planned in the event of limited plasma; however, there was no shortage of CP and controls were all historical<br>- Patients of any age with confirmed Ebola virus disease (including pregnant women) received two units of 200–250 ml from different convalescent donors within 48 hours after laboratory confirmed diagnosis, as recommended by the World Health Organization. The levels of Ebola virus neutralizing antibodies in the plasma were unknown at the time of administration.<br>Design Considerations<br>- Determined that the randomization of patients was locally unacceptable in the volatile setting of the Ebola outbreak (Adebamowo et al., 2014)	February 2015–August 2015	The transfusion of up to 500 ml of convalescent plasma with unknown levels of neutralizing antibodies in 84 patients with confirmed Ebola was not associated with a significant improvement in survival.
Favipiravir<br><br>(MSF, 2015; Sissoko et al., 2016)	Institut national de la santé et de la recherche médicale, France (Inserm); JIKI	Guinea	Trial Design<br>- Multicenter proof-of-concept noncomparative trial<br>- Nonrandom, open-label<br>- Single arm, historical controls	December 2014–June 2015	Efficacy and tolerance inconclusive.

Copyright © National Academy of Sciences. All rights reserved.

Design Considerations

- The trial team decided to rapidly gather preliminary data to guide further research due to the following characteristics of the Ebola outbreak:
    - Urgent need to identify drugs
    - Limited data on the course of the disease
    - Care provision was difficult
- The trial team determined the conditions for running an RCT were not fulfilled due to the following:
    - Course of the outbreak was unpredictable
    - Ebola strikes in clusters
    - "NGOs engaged in Ebola containment and treatment in Guinea and the JIKI trial investigators felt that, given the very high mortality rate of the disease, it was ethically unacceptable to randomize patients from within the same family or village, who appear together to seek care, to receive or not receive an experimental drug" (p. 5)
    - Highly transmissible disease
    - There was the fear that a randomized research design might lead the community to become even more distrustful, and patients more reluctant to seek care

continued

Copyright © National Academy of Sciences. All rights reserved.

TABLE 3-3 Continued

Investigational Product	Sponsoring Organization, Trial Name	Trial Location	Trial Design and Design Considerations	Timeline	Results
Brincidofovir (BCV)<br><br>(Dunning et al., 2016a; Horby, 2015; Whitehead et al., 2016)	University of Oxford; RAPIDE-BCV	Monrovia, Liberia	Trial Design:<br>- The overall design is a multistage strategy that begins with a single-arm sequential design with three stopping boundaries, corresponding to the conclusion that the treatment is effective (powered at 90 percent with n = 140 to detect a survival proportion of 0.90), promising (powered at 95 percent with n = 140 to detect a survival proportion of 0.667), or not promising (Type I error rate of 10 percent with n = 140 if the survival proportion is 0.50)<br>- If the treatment is either effective or promising, the treatment is subjected to further evaluation, i.e., a confirmatory single-arm study if initially effective or a RCT if only promising<br><br>Design Considerations:<br>- A desire to quickly identify highly effective or clearly ineffective treatments<br>- A desire to avoid randomization unless necessary to establish the effectiveness of a treatment, perhaps because of a perception that randomization would not be acceptable or would be infeasible in the trial setting	January 2015	Drug developer Chimerix decided to discontinue a program to test its lead product against the Ebola virus in Liberia (diminishing patient population).<br><br>Chimerix determined to focus on CMV.

Copyright © National Academy of Sciences. All rights reserved.

- The statistician's extensive work on sequential trial designs based on boundaries similar to those used in the trial (e.g., the triangular test of Whitehead), likely facilitating the design and simulation work

Ongoing humanitarian crisis + trial involves risks
- Need to identify useful therapeutic quickly
- Need to discard useless agents quickly

High death rate + volatile conditions
- Unclear if randomization to standard of care would be acceptable

A common approach to developing drugs for patients with solid tumors is a small single-arm phase 2 trial, followed, if promising, by a larger randomized phase 3 comparison with a standard control treatment

continued

Copyright © National Academy of Sciences. All rights reserved.

TABLE 3-3 Continued

Investigational Product	Sponsoring Organization, Trial Name	Trial Location	Trial Design and Design Considerations	Timeline	Results
TKM-130803<br><br>(Dunning et al., 2016b; Horby, 2015; Whitehead et al., 2016)	University of Oxford	Port Loko, Sierra Leone	Trial Design<br>- The overall design is a multistage strategy that begins with a single-arm sequential design with three stopping boundaries, corresponding to the conclusion that the treatment is effective (powered at 90 percent with n = 140 to detect a survival proportion of 0.90), promising (powered at 95 percent with n = 140 to detect a survival proportion of 0.667), or not promising (Type I error rate of 10 percent with n = 140 if the survival proportion is 0.50)<br>- If the treatment is either effective or promising, the treatment is subjected to further evaluation, i.e., a confirmatory single-arm study if initially effective or a RCT if only promising<br><br>Design Considerations:<br>- A desire to quickly identify highly effective or clearly ineffective treatments<br>- A desire to avoid randomization unless necessary to establish the effectiveness of a treatment, perhaps because of a perception that randomization would not be acceptable or would be infeasible in the trial setting	March–June 2015	Early results from the study, demonstrated that TKM-130803 was not effective in increasing the survival fraction above 50 percent; unlikely to demonstrate an overall therapeutic benefit to patients.

Copyright © National Academy of Sciences. All rights reserved.

- The statistician's extensive work on sequential trial designs based on boundaries similar to those used in the trial (e.g., the triangular test of Whitehead), likely facilitating the design and simulation work

Ongoing humanitarian crisis + trial involves risks
- Need to identify useful therapeutic quickly
- Need to discard useless agents quickly

High death rate + volatile conditions
- Unclear if randomization to standard of care would be acceptable

A common approach to developing drugs for patients with solid tumors is a small single-arm Phase 2 trial, followed, if promising, by a larger randomized Phase 3 comparison with a standard control treatment

continued

Copyright © National Academy of Sciences. All rights reserved.

TABLE 3-3 Continued

Investigational Product	Sponsoring Organization, Trial Name	Trial Location	Trial Design and Design Considerations	Timeline	Results
ZMapp<br><br>(PREVAIL II Writing Group, 2016)	National Institute of Allergy and Infectious Diseases (NIAID)–PREVAIL II	Liberia, Guinea, Sierra Leone, United States<br><br>The trial enrolled 72 adults and children with confirmed Ebola infection from Guinea (12 patients), Liberia (5 patients), Sierra Leone (54 patients), and the United States (1 patient)	This intervention trial is a multinational/multicenter, Phase 1/2 randomized, open-label trial consisting of 2 arms—ZMapp + optimized standard of care (oSOC) versus oSOC only.<br>- oSOC included providing intravenous (IV) fluids, balancing electrolytes, maintaining oxygen status and blood pressure, and treating other infections if they were identified/occurred and could be somewhat variable based on site<br>- ZMapp was given as 3 IV infusions of 50 mg/kg 3 days apart<br>- Patients were randomized 1:1 for the two groups<br>- Patients were stratified by<br>• Baseline cycle threshold (CT) value (≤22 versus >22) on PCR<br>▪ Lower CT value = higher viral load<br>▪ Higher CT value = lower viral load<br>• Treatment site (U.S. versus Liberia/Sierra Leone versus Guinea [where favipiravir was part of oSOC]).<br>- Primary endpoint: mortality at 28 days<br>- Secondary endpoints:<br>• Clinical and virology effects of experimental treatment<br>• Adverse events<br>• Plasma viral load changes over time	March 2015–November 2015	A total of 72 patients were enrolled at sites in Guinea, Liberia, Sierra Leone, and the United States. Of the 71 patients who could be evaluated, 21 died, representing an overall case fatality rate of 30 percent. Death occurred in 13 of 35 patients (37 percent) who received the current standard of care alone and in 8 of 36 patients (22 percent) who received the current standard of care plus ZMapp. The observed posterior probability that ZMapp plus the current standard of care was superior to the current standard of care alone was 91.2 percent, falling short of the prespecified threshold of 97.5 percent.

Copyright © National Academy of Sciences. All rights reserved.

	Design Considerations:<br>- The study protocol was designed to be flexible to include a series of two-arm comparisons of novel interventions (the first being ZMapp) compared to oSOC to establish a framework to evaluate multiple potential Ebola treatments in the future. If one investigational treatment were to prove to be statistically more effective (in improving mortality over that achievable through oSOC alone), it would then become the basis of the new SOC against which additional investigational Ebola interventions could be tested and compared<br>- Frequent interim monitoring by an independent data and safety monitoring board to facilitate early elimination of poorly performing treatments as well as the introduction of new candidate therapies was incorporated in the trial<br>- The plan was for each experimental therapy to be examined in up to 100 participants per arm. If investigators were to be unable to establish a significant difference after enrolling 100 participants per arm, then that particular treatment would be declared ineffective and investigators would begin testing the next therapy<br>- The trial was stopped after 72 of 200 patients were enrolled due to the end of the epidemic	Frequentist analyses yielded similar results (absolute difference in mortality with ZMapp, –15 percentage points; 95 percent confidence interval, –36 to 7). Baseline viral load was strongly predictive of both mortality and duration of hospitalization in all age groups.<br><br>ZMapp showed promise as a possible effective treatment agent for Ebola but there were insufficient data to determine definitively whether it is a better treatment for Ebola than supportive care alone.

Copyright © National Academy of Sciences. All rights reserved.

REFERENCES

ACOG (American College of Obstetricians and Gynecologists). 2013. *Hormone therapy and heart disease. Committee opinion no. 565*. Washington, DC: American Congress of Obstetricians and Gynecologists. http://www.acog.org/Resources-And-Publications/Committee-Opinions/Committee-on-Gynecologic-Practice/Hormone-Therapy-and-Heart-Disease (accessed January 17, 2017).

Adebamowo, C., O. Bah-Sow, F. Binka, R. Bruzzone, A. Caplan, J.-F. Delfraissy, D. Heymann, P. Horby, P. Kaleebu, J.-J. M. Tamfum, P. Olliaro, P. Piot, A. Tejan-Cole, O. Tomori, A. Toure, E. Torreele, and J. Whitehead. 2014. Randomised controlled trials for Ebola: Practical and ethical issues. *The Lancet* 384(9952):1423–1424.

Beck, Z., Z. Prohászka, and G. Füst. 2008. Traitors of the immune system—Enhancing antibodies in HIV infection: Their possible implication in HIV vaccine development. *Vaccine* 26(24):3078–3085.

Chimerix Inc. 2015. *Chimerix focusing efforts on CMV and adenovirus pivotal trials*. http://ir.chimerix.com/releasedetail.cfm?releaseid=893927 (accessed December 21, 2016).

Cohen, J. 2015. Results from encouraging Ebola trial scrutinized. *Science Insider*, Feb. 26. http://www.sciencemag.org/news/2015/02/results-encouraging-ebola-trial-scrutinized (accessed January 21, 2017).

Cohen, J., and M. Enserink. 2016. As Ebola epidemic draws to a close, a thin scientific harvest. *Science* 351(6268):12–13.

Davey, R. T. J. 2016. PREVAIL II: A randomized controlled trial of ZMapp™ in acute Ebola virus infection. Paper read at Conference on Retroviruses and Opportunistic Infections 2017, February 22–25, Boston, MA.

Dodd, L. E., M. A. Proschan, J. Neuhaus, J. S. Koopmeiners, J. Neaton, J. D. Beigel, K. Barrett, H. C. Lane, and R. T. Davey, Jr. 2016. Design of a randomized controlled trial for Ebola virus disease medical countermeasures: PREVAIL II, the Ebola MCM study. *Journal of Infectious Diseases* 213(12):1906–1913.

Dunning, J., S. B. Kennedy, A. Antierens, J. Whitehead, I. Ciglenecki, G. Carson, R. Kanapathipillai, L. Castle, R. Howell-Jones, R. Pardinaz-Solis, J. Grove, J. Scott, T. Lang, P. Olliaro, and P. W. Horby, for the RAPIDE- BCV trial team. 2016a. Experimental treatment of Ebola virus disease with brincidofovir. *PLoS ONE* 11(9):e0162199.

Dunning, J., F. Sahr, A. Rojek, F. Gannon, G. Carson, B. Idriss, T. Massaquoi, R. Gandi, S. Joseph, H. K. Osman, T. J. G. Brooks, A. J. H. Simpson, I. Goodfellow, L. Thorne, A. Arias, L. Merson, L. Castle, R. Howell-Jones, R. Pardinaz-Solis, B. Hope-Gill, M. Ferri, J. Grove, M. Kowalski, K. Stepniewska, T. Lang, J. Whitehead, P. Olliaro, M. Samai, and P. W. Horby, for the RAPIDE-TKM trial team. 2016b. Experimental treatment of Ebola virus disease with TKM-130803: A single-arm Phase 2 clinical trial. *PLoS Medicine* 13(4):e1001997.

Dye, J. M., A. S. Herbert, A. I. Kuehne, J. F. Barth, M. A. Muhammad, S. E. Zak, R. A. Ortiz, L. I. Prugar, and W. D. Pratt. 2012. Postexposure antibody prophylaxis protects nonhuman primates from filovirus disease. *Proceedings of the National Academy of Sciences of the United States of America* 109(13):5034–5039.

Edwards, T., M. G. Semple, A. De Weggheleire, Y. Claeys, M. De Crop, J. Menten, R. Ravinetto, S. Temmerman, L. Lynen, E. I. Bah, P. G. Smith, and J. van Griensven, on behalf of the Ebola-Tx Consortium. 2016. Design and analysis considerations in the Ebola-Tx trial evaluating convalescent plasma in the treatment of Ebola virus disease in Guinea during the 2014–2015 outbreak. *Clinical Trials (London, England)* 13(1):13–21.

Copyright © National Academy of Sciences. All rights reserved.

Integrating Clinical Research into Epidemic Response: The Ebola Experience

EMA (European Medicines Agency). 2005. *Guideline on procedures for the granting of a marketing authorisation under exceptional circumstances, pursuant to article 14 (8) of regulation (ec) no 726/2004 release*. http://www.ema.europa.eu/docs/en_GB/document_library/Regulatory_and_procedural_guideline/2009/10/WC500004883.pdf (accessed March 8, 2017).

FDA (U.S. Food and Drug Administration). 2016. *Investigational new drug (IND) application*. http://www.fda.gov/drugs/developmentapprovalprocess/howdrugsaredevelopedandapproved/approvalapplications/investigationalnewdrugindapplication/default.htm (accessed February 20, 2017).

FujiFilm Corporation. 2016. *The anti-influenza drug "Avigan Tablet" selected as one of the supplies to be procured with the Japanese government's emergency grant aid for countering the Ebola virus disease in Guinea*. http://www.fujifilm.com/news/n160614.html (accessed January 17, 2017).

Furuta, Y., B. B. Gowen, K. Takahashi, K. Shiraki, D. F. Smee, and D. L. Barnard. 2013. Favipiravir (T-705), a novel viral RNA polymerase inhibitor. *Antiviral Research* 100(2): 446–454.

Geisbert, T. W., A. C. H. Lee, M. Robbins, J. B. Geisbert, A. N. Honko, V. Sood, J. C. Johnson, S. de Jong, I. Tavakoli, A. Judge, L. E. Hensley, and I. MacLachlan. 2010. Postexposure protection of non-human primates against a lethal Ebola virus challenge with RNA interference: A proof-of-concept study. *The Lancet* 375(9729):1896–1905.

Haque, A., D. Hober, and J. Blondiaux. 2015. Addressing therapeutic options for Ebola virus infection in current and future outbreaks. *Antimicrobial Agents and Chemotherapy* 59(10):5892–5902.

HHS (U.S. Department of Health and Human Services). 2014. *Guidance for industry: Expedited programs for serious conditions – drugs and biologics*. https://www.fda.gov/downloads/drugs/guidancecomplianceregulatoryinformation/guidances/ucm358301.pdf (accessed March 8, 2017).

HHS. 2015. *Product development under the animal rule: Guidance for industry*. Silver Spring, MD: U.S. Department of Health and Human Services, Food and Drug Administration, Center for Drug Evaluation and Research, Center for Biologics Evaluation and Research. http://www.fda.gov/downloads/drugs/guidancecomplianceregulatoryinformation/guidances/ucm399217.pdf (accessed January 17,2017).

Horby, P. 2015. *Trials of antivirals for Ebola ISARIC experience*. http://www.ebolatx.eu/wp-content/uploads/2015/09/Peter-Horby-ECTMIH-Sept-2015.pdf (accessed January 17, 2017).

ITM (Institute of Tropical Medicine). 2016. *Ebola-Tx Project*. http://www.ebolatx.eu (accessed January 17, 2017).

Kroll, D. 2015. Chimerix ends brincidofovir Ebola trials to focus on adenovirus and CMV. *Forbes*, January 31. http://www.forbes.com/sites/davidkroll/2015/01/31/chimerix-ends-brincidofovir-ebola-trials-to-focus-on-adenovirus-and-cmv/#5e8d557069d8 (accessed January 21, 2017).

Marano, G., S. Vaglio, S. Pupella, G. Facco, L. Catalano, G. M. Liumbruno, and G. Grazzini. 2016. Convalescent plasma: New evidence for an old therapeutic tool? *Blood Transfusion* 14(2):152–157.

MSF (Médecins sans Frontières). 2015. *Preliminary results of the JIKI clinical trial to test the efficacy of favipiravir in reducing mortality in individuals infected by Ebola virus in Guinea*. http://www.msf.org/en/article/preliminary-results-jiki-clinical-trial-test-efficacy-favipiravir-reducing-mortality (accessed December 21, 2016).

Nakayama, E., D. Tomabechi, K. Matsuno, N. Kishida, R. Yoshida, H. Feldmann, and A. Takada. 2011. Antibody-dependent enhancement of Marburg virus infection. *Journal of Infectious Diseases* 204(Suppl 3):S978–S985.

Copyright © National Academy of Sciences. All rights reserved.

Integrating Clinical Research into Epidemic Response: The Ebola Experience

Prasad, V., and A. Cifu. 2011. Medical reversal: Why we must raise the bar before adopting new technologies. *Yale Journal of Biology and Medicine* 84(4):471–478.

Prasad, V., A. Cifu, and J. A. Ioannidis. 2012. Reversals of established medical practices: Evidence to abandon ship. *JAMA* 307(1):37–38.

Prasad, V., A. Vandross, C. Toomey, M. Cheung, J. Rho, S. Quinn, S. J. Chacko, D. Borkar, V. Gall, S. Selvaraj, N. Ho, and A. Cifu. 2013. A decade of reversal: An analysis of 146 contradicted medical practices. *Mayo Clinic Proceedings* 88(8):790–798.

PREVAIL II Writing Group. 2016. A randomized, controlled trial of ZMapp for Ebola virus infection. *New England Journal of Medicine* 375(15):1448–1456.

Qiu, X., G. Wong, J. Audet, A. Bello, L. Fernando, J. B. Alimonti, H. Fausther-Bovendo, H. Wei, J. Aviles, E. Hiatt, A. Johnson, J. Morton, K. Swope, O. Bohorov, N. Bohorova, C. Goodman, D. Kim, M. H. Pauly, J. Velasco, J. Pettitt, G. G. Olinger, K. Whaley, B. Xu, J. E. Strong, L. Zeitlin, and G. P. Kobinger. 2014. Reversion of advanced Ebola virus disease in nonhuman primates with ZMapp. *Nature* 514(7520):47–53.

Reuters. 2015. *Guinea to expand use of experimental anti-Ebola drugs*. Reuters, February 7. http://www.reuters.com/article/us-health-ebola-guinea-idUSKBN0LB0Q620150207 (accessed December 21, 2016).

Sissoko, D., C. Laouenan, E. Folkesson, A.-B. M'Lebing, A.-H. Beavogui, S. Baize, A.-M. Camara, P. Maes, S. Shepherd, C. Danel, S. Carazo, M. N. Conde, J.-L. Gala, G. Colin, H. Savini, J. A. Bore, F. Le Marcis, F. R. Koundouno, F. Petitjean, M.-C. Lamah, S. Diederich, A. Tounkara, G. Poelart, E. Berbain, J.-M. Dindart, S. Duraffour, A. Lefevre, T. Leno, O. Peyrouset, L. Irenge, N. F. Bangoura, R. Palich, J. Hinzmann, A. Kraus, T. S. Barry, S. Berette, A. Bongono, M. S. Camara, V. Chanfreau Munoz, L. Doumbouya, H. Souley, P. M. Kighoma, F. R. Koundouno, L. Réné, C. M. Loua, V. Massala, K. Moumouni, C. Provost, N. Samake, C. Sekou, A. Soumah, I. Arnould, M. S. Komano, L. Gustin, C. Berutto, D. Camara, F. S. Camara, J. Colpaert, L. Delamou, L. Jansson, E. Kourouma, M. Loua, K. Malme, E. Manfrin, A. Maomou, A. Milinouno, S. Ombelet, A. Y. Sidiboun, I. Verreckt, P. Yombouno, A. Bocquin, C. Carbonnelle, T. Carmoi, P. Frange, S. Mely, V.-K. Nguyen, D. Pannetier, A.-M. Taburet, J.-M. Treluyer, J. Kolie, R. Moh, M. C. Gonzalez, E. Kuisma, B. Liedigk, D. Ngabo, M. Rudolf, R. Thom, R. Kerber, M. Gabriel, A. Di Caro, R. Wölfel, J. Badir, M. Bentahir, Y. Deccache, C. Dumont, J.-F. Durant, K. El Bakkouri, M. Gasasira Uwamahoro, B. Smits, N. Toufik, S. Van Cauwenberghe, K. Ezzedine, E. Dortenzio, L. Pizarro, A. Etienne, J. Guedj, A. Fizet, E. Barte de Sainte Fare, B. Murgue, T. Tran-Minh, C. Rapp, P. Piguet, M. Poncin, B. Draguez, T. Allaford Duverger, S. Barbe, G. Baret, I. Defourny, M. Carroll, H. Raoul, A. Augier, S. P. Eholie, Y. Yazdanpanah, C. Levy-Marchal, A. Antierrens, M. Van Herp, S. Günther, X. de Lamballerie, S. Keïta, F. Mentre, X. Anglaret, D. Malvy, and JIKI Study Group. 2016. Experimental treatment with favipiravir for Ebola virus disease (the JIKI trial): A historically controlled, single-arm proof-of-concept trial in Guinea. *PLoS Medicine* 13(3):e1001967.

van Griensven, J., T. Edwards, X. de Lamballerie, M. G. Semple, P. Gallian, S. Baize, P. W. Horby, H. Raoul, N. F. Magassouba, A. Antierens, C. Lomas, O. Faye, A. A. Sall, K. Fransen, J. Buyze, R. Ravinetto, P. Tiberghien, Y. Claeys, M. De Crop, L. Lynen, E. I. Bah, P. G. Smith, A. Delamou, A. De Weggheleire, and N. Haba. 2016a. Evaluation of convalescent plasma for Ebola virus disease in Guinea. *New England Journal of Medicine* 374(1):33–42.

Copyright © National Academy of Sciences. All rights reserved.

van Griensven, J., T. Edwards, X. de Lamballerie, M. G. Semple, P. Gallian, S. Baize, P. W. Horby, H. Raoul, N. F. Magassouba, A. Antierens, C. Lomas, O. Faye, A. A. Sall, K. Fransen, J. Buyze, R. Ravinetto, P. Tiberghien, Y. Claeys, M. De Crop, L. Lynen, E. I. Bah, P. G. Smith, A. Delamou, A. De Weggheleire, and N. Haba. 2016b. Evaluation of convalescent plasma for Ebola virus disease in Guinea. *New England Journal of Medicine* 374(1):33–42.

Wellcome Trust. 2015. *New trial of TKM-Ebola treatment to start in Sierra Leone*. https://wellcome.ac.uk/press-release/new-trial-tkm-ebola-treatment-start-sierra-leone (accessed December 21, 2016).

Whitehead, J., P. Olliaro, T. Lang, and P. Horby. 2016. Trial design for evaluating novel treatments during an outbreak of an infectious disease. *Clinical Trials* 13(1):31–38.

WHO (World Health Organization). 2014a. *Ethical issues related to study design for trials on therapeutics for Ebola virus disease.* Geneva, Switzerland: WHO Ethics Working Group Meeting, October 20–21, 2014. http://apps.who.int/iris/bitstream/10665/137509/1/WHO_HIS_KER_GHE_14.2_eng.pdf (accessed January 17, 2017).

WHO. 2014b. *WHO meeting of the Scientific and Technical Advisory Committee on Ebola Experimental Interventions—Briefing note.* http://www.who.int/medicines/ebola-treatment/scientific_tech_meeting/en (accessed December 21, 2016).

WHO. 2015a. *Categorization and prioritization of drugs for consideration for testing or use in patients infected with Ebola*. http://www.who.int/medicines/ebola-treatment/2015_0703TablesofEbolaDrugs.pdf?ua=1 (accessed January 21, 2017).

WHO. 2015b. *Ebola R&D landscape of clinical candidates and trials*. Geneva, Switzerland: World Health Organization. http://www.who.int/medicines/ebola-treatment/EbolaR_D_public-report_updt2015.pdf (accessed January 21, 2017).

WHO. 2016a. *Graph of new confirmed cases per epi week for Guinea*. http://apps.who.int/gho/data/node.ebola-sitrep.ebola-country-GIN-20160511?lang=en (accessed January 17, 2017).

WHO. 2016b. *Graph of new confirmed cases per epi week for Liberia*. http://apps.who.int/gho/data/view.ebola-sitrep.ebola-country-LBR-20160511-graph?lang=en (accessed January 17, 2017).

WHO. 2016c. *Graph of new confirmed cases per epi week for Sierra Leone*. http://apps.who.int/gho/data/view.ebola-sitrep.ebola-country-SLE-20160511-graph?lang=en (accessed January 17, 2017).

Writing Group for the Women's Health Initiative. 2002. Risks and benefits of estrogen plus progestin in healthy postmenopausal women: Principal results from the women's health initiative randomized controlled trial. *JAMA* 288(3):321–333.

Copyright © National Academy of Sciences. All rights reserved.

Copyright © National Academy of Sciences. All rights reserved.

4

Assessment of Vaccine Trials

Overall, the Ebola vaccine trials displayed better coordination and cooperation among international researchers, regulators, manufacturers, funders, and the national authorities and communities of the Ebola affected countries than did the therapeutic trials. In fact, some candidates were already available and had been tested in nonhuman primates in the decade before the West Africa outbreak began. As a result, the trials were designed, approved, and implemented quickly; in one case, "a first-in-human Phase 1 was authorized in 4 working days by regulators in the UK, including initial assessment and time to review responses by the applicant" (WHO, 2015c). However, as with the design of the therapeutic trials, there were also disagreements, competition, and infighting among the organizations that were carrying out the trials (see Chapter 2 for more detail on the disagreements and Chapter 3 for details on the therapeutic trials conducted). Most notably, while there was consensus that clinical trial data would be necessary in order to support the licensure of any investigational vaccine candidate, there was little agreement concerning the preferred specific design or execution of the trials.

On September 29–30, 2014, with the international response just beginning in earnest, the World Health Organization (WHO) convened a meeting to coordinate the planned clinical trials for candidate Ebola vaccines. At this time, the GlaxoSmithKline (GSK) ChAd3 vaccine and the Newlink rVSV vaccine were the only candidates that met the criteria laid out by the

Copyright © National Academy of Sciences. All rights reserved.

WHO the previous month.[1] These criteria required "availability of good manufacturing practice grade vials after lot release for clinical trials, and 100 percent efficacy had been documented in nonhuman primates with acceptable preclinical safety" (WHO, 2015c).

With these vaccine candidates selected, debate shifted to concerns regarding the best trial designs for testing their efficacy. While determining the best designs for vaccine trials involved many of the same issues complicating the design of therapeutic trials—randomization, community perspectives, and access to potential benefit—the vaccine trials also posed distinct ethical issues. First, a person receiving a vaccine would presumably not be infected with Ebola and therefore not at immediate risk of death. Many of the arguments against randomized controlled trials (RCTs) for Ebola therapeutics centered on the ethics of giving an infected person a placebo or only standard of care when the risk of death was so high without an effective intervention. The risks of adverse effects from an unproven investrigational agent were considered by some to be smaller than the risks of not providing one. However, with a vaccine, the risks of adverse effects may outweigh the risks of not receiving the vaccine since the participant may or may not be exposed to Ebola. Giving a potentially harmful agent to a healthy person has different implications than giving a potentially harmful agent to someone who is at a high risk of death, such as a patient suffering from Ebola, as Dawson (2015) noted: "[I]t is not so clear that when not infected [a person] would or should be willing to accept an unknown risk from an unlicensed preventive vaccine, given that other measures such as good quality protective equipment, if properly used, may reduce the risk of infection to an acceptable level" (Dawson, 2015, p. 108). Second, research participants who have received a vaccine or a placebo may believe that they are protected from infection. They may not even consider the possibility that they may have received the placebo or that even if they got the vaccine it might not be effective, and, as a consequence, they may fail to take all proscribed safety precautions, such as the proper donning and doffing of personal protective equipment while caring for Ebola patients. "Known as risk compensation, this behavioral adjustment draws on the theory of 'risk homeostasis,' which has previously been applied to phenomena as diverse as Lyme disease vaccination, insurance mandates, and automobile safety" (Underhill, 2013, p. 115).

At the WHO meeting in September 2014, participants discussed the scientific and ethical issues involved in designing vaccine trials. As reported in *Science*, a researcher with the Ebola vaccine development program at GSK said, "Going into this meeting, we were told the idea of a controlled

[1] J&J (Ad26/MVA) and Novavax (recombinant protein) met this criteria later in the epidemic (WHO, 2015d).

Copyright © National Academy of Sciences. All rights reserved.

trial . . . was not going to be acceptable" (Cohen and Kupferschmidt, 2014b). Yet, the trial design he presented included one-to-one randomization between the investigational vaccine and an active control, which would be an approved vaccine for another disease such as hepatitis B. The GSK representative maintained that this design would determine the efficacy of the vaccine much faster than alternative designs. However, some participants—particularly those from Médecins Sans Frontières (MSF)—disagreed and argued that, as with therapeutic agents, any vaccine trial involving a placebo or active control arm would be unethical (Cohen and Kupferschmidt, 2014b).

Randomization was seen as particularly problematic for health care workers, who were at high risk of contracting Ebola. A representative from the Wellcome Trust asked, "If you were there tomorrow and you were a health care worker, would you be willing to be in a control arm, when the next 3 months you will be looking after patients with Ebola?" (Cohen and Kupferschmidt, 2014a, p. 290). One MSF representative, who oversaw experimental Ebola products for MSF, told *Science*, "Studies on efficacy in affected countries and more so in at-risk populations should not have a placebo or active control arm as this cannot be defended ethically" (Cohen and Kupferschmidt, 2014a). However, at the time there were no in-human data to determine the risk–benefit balance between the benefit of the vaccine and the risk of side effects.

"The meeting was quite tense at moments," said Marie-Paule Kieny, WHO assistant director-general and vaccine expert (Cohen and Kupferschmidt, 2014a), and determining the choice of control arm proved to be one of the most contentious points in designing Ebola vaccine trials. There were three main options: a placebo control, an active vaccination (with a non-Ebola vaccine), or delayed vaccination (Nason, 2016). The placebo-controlled trial was argued by some to be unethical due to a responsibility of researchers to provide something of value to research participants (Cohen and Kupferschmidt, 2014b). While many at the September 29–30 WHO meeting argued that using an active control would be the fastest method for determining the safety and efficacy of the vaccine, this design did not win over all meeting participants. As a representative from the Wellcome Trust put it, "An RCT may yield results faster, but if it's simply unacceptable for trial participants, a stepped-wedge design is preferable" (Cohen and Kupferschmidt, 2014a). The stepped-wedge design became the leading alternative trial design and ultimately best addressed the concerns of the meeting participants (Cohen and Kupferschmidt, 2014a). A stepped-wedge trial rolls out the intervention to participants over time, either as individuals or in clusters. By the time the study ends, all participants will have received the intervention, but they will have received it in a random order and in some cases the intervention will have been delayed.

Copyright © National Academy of Sciences. All rights reserved.

Researchers are then able to learn about efficacy by looking at when participants received the vaccine and if and when they were infected in order to calculate how much protection the vaccine provided (Brown and Lilford, 2006). Stepped-wedge designs do have drawbacks, including an inability to determine long-term harm from vaccination, a difficulty determining how long to wait before vaccine administration to the delay group, and difficulty determining whether an infection-enhancing immune adverse response might be induced, as has been seen with other vaccines, such as respiratory syncytial virus (Openshaw and Tregoning, 2005). On the other hand, since all participants would receive the Ebola vaccine, the design appealed to those opposed to placebos or active controls for ethical reasons. See Box 4-1 for WHO requirements for Ebola vaccine trials.

At the October 23 WHO meeting the U.S. National Institutes of Health (NIH) and the U.S. Centers for Disease Control and Prevention (CDC)

BOX 4-1
WHO Requirements of Vaccine Trials

As reported by WHO, the following set of activities were deemed important and were initiated by the international community:

1. Parallel Phase 1–2 trials had to be launched in sites with optimal first-in-human clinical management facilities, followed as quickly as possible by Phase 1–2 in Africa. These trials were to be conducted on highly expedited timelines. The trials were to be larger than usual for Phase 1 trials in order to allow for simultaneous safety, immunogenicity, and dose-finding evaluations.
2. Given the lack of a standardized assay, centralized laboratory facilities were chosen to allow for head-to-head comparability evaluations between all clinical trial sites and between different vaccines.
3. Data management by investigator-initiated trials was to be promoted, with data transfer to the entities responsible for licensure submission. Independent oversight including data safety monitoring boards as well as good clinical practice[a] training and monitoring needed to be established. All regulatory and ethics oversight steps would need to occur to the same high standards but in greatly compressed timelines.
4. The trial protocols were adapted to take into consideration the safety and immunogenicity results of the Phase 1 trial as they became available and also to take into consideration the evolution of the epidemic.

[a] New guidelines for GCP have been recently released and provide insight into training and implementation (http://www.ich.org/products/gcp-renovation.html, accessed February 20, 2017).
SOURCE: WHO, 2015c.

Copyright © National Academy of Sciences. All rights reserved.

trials, to take place in Liberia and Sierra Leone, respectively (discussed in more detail below), were presented and generally supported. However, at that time there were no trials planned in Guinea (WHO, 2015a). As a result a small group formed at this meeting to discuss options for implementing a vaccine trial in Guinea. A Guinea Ebola vaccine trial working group[2] was formed which determined the trial designs to be used in Guinea. The working group consisted of multiple stakeholders, including the WHO, academics, representatives from U.S. government, and representatives from GSK, Newlink, and Merck (WHO, 2015a).

In selecting trial designs, the NIH determined that in order to test safety and efficacy in the most robust way a traditional placebo-controlled RCT should be implemented while the CDC was more geared toward distributing vaccines to the population, which led to the use of a two-arm immediate and delayed vaccination approach with individual randomization. The main motivation for the Guinea ring vaccination approach was that the working group (formed at the October 23 WHO meeting) saw that there was not sufficient capacity for doing a large population-based trial and therefore decided on two other populations: the rings around new cases and the frontline workers. Individual randomization was considered in the ring vaccination trial, but it was decided that cluster randomization would be more feasible given the logistical and capacity issues.

When the public health emergency of international concern (PHEIC) was declared on August 8, 2014, the WHO convened a meeting to be held that month to discuss how to "fast-track the testing and the deployment of promising vaccines in sufficient numbers to use in the field in 2015 to try and impact the Ebola epidemic curve" (WHO, 2015c). At this meeting it was agreed that Phase 1 trials would launch and that before Phase 1 trials were completed, efficacy trials in the affected countries would be initiated. This decision made it difficult for manufacturers, as they were unsure which dose would be required for Phase 2 trials (Mohammadi, 2015). See Table 4-1 for details on the Phase 1 Ebola vaccine trials initiated during the Ebola outbreak.

ASSESSMENT OF TRIALS

Below is an individual assessment of the vaccine trials that were conducted in the Ebola-affected countries during the Ebola epidemic, including assessments of their study designs and conduct, results, and analyses. In-depth descriptions of the different trials are available in the published

[2] Testimonies of Peter Smith and Ana Maria Henao Restrepo at the Public Workshop of the Committee on Clinical Trials During the 2014–2015 Ebola Outbreak. London, UK; March 2016.

Copyright © National Academy of Sciences. All rights reserved.

TABLE 4-1 October 2015 WHO Summary of the Phase 1 Ebola Vaccine Trials

Product/Company	Phase	Trial Location	Dates
ChAd3-ZEBOV GlaxoSmithKline and PHAC	Phase 1	By VRC at NIH, USA	September 2014
		By Oxford University in the UK	
		By CVD in Mali	October 2014
		At the University of Lausanne, Lausanne, Switzerland	
rVSV-ZEBOV NewLink Genetics and Merck Vaccines USA	Phase 1	By WRAIR in the US	October 2014
		By NIAID in the US	
		By CTC North GmbH in Hamburg, Germany	November 2014
		At Albert Schweitzer Hospital in Lambarene, Gabon	
		At the University of Geneva, Geneva, Switzerland	
		At the IWK Health Center, Halifax, Canada	
		By KEMRI Wellcome Trust in Kilifi, Kenya	December 2014
Ad26.ZEBOV and MVA-BN-Filo Johnson & Johnson and Bavarian Nordic	Phase 1	By University of Oxford in the UK and NIAID, USA	January 2015
		By University of Nairobi, Kenya	Second half of 2015
		By MRC, Uganda Virus Research Institute, Uganda	
		By Mwanza Intervention Trials Unit, United Republic of Tanzania	
Recombinant protein Ebola vaccine candidate Novavax	Phase 1	Australia	February 2015

SOURCE: Adapted from WHO, 2015a.

Copyright © National Academy of Sciences. All rights reserved.

manuscripts for these trials. Similar to the case with the therapeutic trials, preparation and planning for the vaccine trials started in September 2014 and Phase 2 and Phase 3 trials began enrolling participants between February 2015 and October 2015 (see Table 4-2). While the trials were launched rapidly, the Phase 2 and Phase 3 trials began participant enrollment at the tail end of the epidemic (see Figure 4-1).

Ebola ça Suffit–Guinea Ring Vaccination Trial; Guinea (rVSV-ZEBOV)

Among all of the therapeutic and vaccine trials conducted in West Africa during the outbreak, the ring vaccination trial came the closest to fulfilling the hope for a clinical trial "home run" (or a "six," its cricket equivalent). It was a collaboration among the government of Guinea, WHO, MSF, and the Norwegian Institute of Public Health that demonstrated it was possible to perform a type of randomized study during the outbreak, despite apparent substantial opposition to randomized trials by stakeholders involved in the Ebola response (Ebola ça Suffit Ring Vaccination Trial Consortium, 2015). In an example of excellent communication, coordination, and a willingness to compromise between researchers and public health officials, the study took advantage of the public health contact tracing efforts implemented during the outbreak. Index persons in the immediate vaccination clusters were hospitalized, on average, within 3.9 days after symptom onset; clusters defined for the index person were randomized, on average, within 9.7 days of symptom onset in the index person, with similar numbers in the delayed vaccination clusters (Henao-Restrepo et al., 2016). In appreciation of the success in launching this trial, *The Lancet* editors observed, "That such a trial was even possible is a testament not only to the skill of the research teams but also to the commitment of communities to defeating an epidemic that has devastated their nation. Over 90 percent of the study's staff was from Guinea. Before this work, no clinical trial on this scale had ever been performed in the country" (*The Lancet*, 2015).

Study Design

The trial design was a cluster-randomized controlled study modeled on the ring vaccination approach used in the 1970s to eradicate smallpox (WHO, 2015b). Ring vaccination is a measure used to control the spread of an infection that involves vaccinating individuals who are socially or geographically connected to a known case, thereby creating a protective "ring" of immunity around infected individuals to prevent further spread (Rid and Miller, 2016). In the Ebola ring vaccination trial, participants were enrolled and randomized into two groups, one of which was vaccinated immediately and the other of which was assigned to receive the vaccine

Copyright © National Academy of Sciences. All rights reserved.

TABLE 4-2 Timeline of Vaccine Trials

Trial Name (Vaccine)	Phase	Location	Trial Enrollment Start[a]	Trial Enrollment End	Number of Participants
Guinea Ring Vaccine VSV-EBOV	Phase 3	Guinea	April 1, 2015	July 20, 2015	7,284
CDC STRIVE VSV-EBOV	Phase 3	Sierra Leone	April 9, 2015	August 21, 2015	8,673
PREVAIL I VSV-EBOV/ChAd3	Phase 2[b]	Liberia	February 2, 2015	April 30, 2015	1,500
EBOVAC–Salone Ad26.ZEBOV and MVA-BN-Filo	Staged Phase 3	Sierra Leone	October 8, 2015	As of February 2017 this study is currently recruiting participants[c]	

NOTE: CDC = U.S. Centers for Disease Control and Prevention; EBOVAC = Ebola vaccine projects; STRIVE = Sierra Leone Trial to Introduce a Vaccine Against Ebola; PREVAIL = Partnership for Research on Ebola Vaccines in Liberia.

[a] The Phase 2 and Phase 3 trials did not begin earlier in the course of the outbreak because the manufacturers did not have a clear view of the required doses and the investigators were still working on community engagement (Mohammadi, 2015).

[b] In March 2015, Independent DSMB recommended moving to the Phase 2 PREVAIL trial to Phase 3; however, no cases of Ebola were reported for 2 weeks in Liberia and plans were made to move Phase 3 to other countries if possible.

[c] Janssen Vaccines & Prevention B.V. 2017. *Staged Phase 3 Study to Assess the Safety and Immunogenicity of Ebola Candidate Vaccines Ad26. ZEBOV and MVA-BN-Filo During Implementation of Stages 1 and 2 (EBOVAC-Salone)*. https://clinicaltrials.gov/ct2/show/NCT02509494 (accessed February 20, 2017).

Copyright © National Academy of Sciences. All rights reserved.

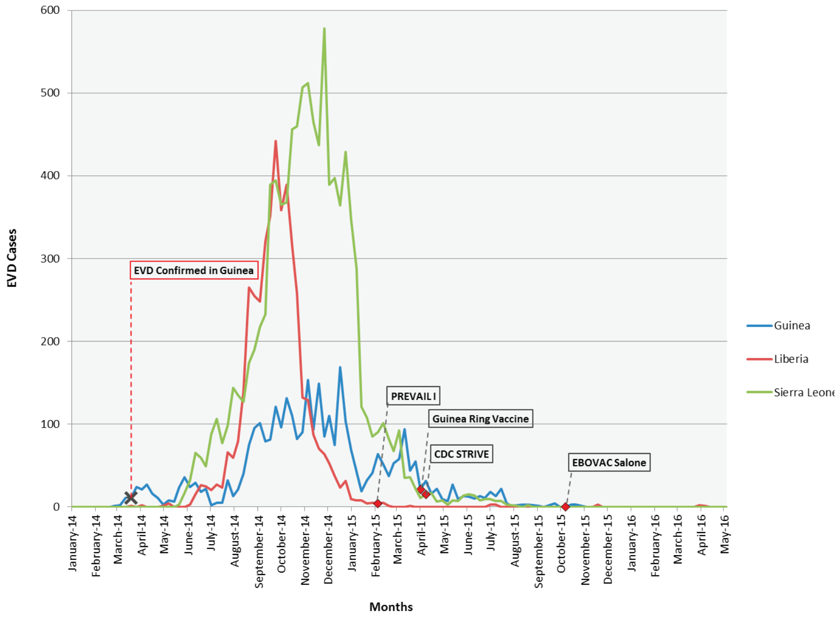

FIGURE 4-1 Clinical trial enrollment start dates during the 2014–2015 Ebola outbreak—vaccine trials.
NOTE: The figure plots the trial start dates on the time course of the Ebola epidemic (confirmed cases) in each of the respective countries (Guinea, Liberia, and Sierra Leone) where the trials were conducted.
SOURCES: The confirmed Ebola case number was obtained from WHO incident reports (2014, 2016b,c,d).

21 days after enrollment. Based on the known incubation period of 2–21 days after infection before symptoms appear and on the fact that it takes some time for vaccine-induced protection to develop (if the vaccine actually works), the period of observation for risk of infection—or, conversely, protection from infection—was set for both groups as the 21-day period from 10 to 30 days post-enrollment (Ebola ça Suffit Ring Vaccination Trial Consortium, 2015). This design was chosen at least in part as a pragmatic solution to address the ethical concerns surrounding the use of an unproven vaccine and an unvaccinated control group. As one researcher noted, "A traditional trial with a placebo control would have been contentious and politically unacceptable, given the known mortality of Ebola and the lack of other options for prevention or treatment. To substitute an inert substance for a potentially life-saving vaccine, given the circumstances, would not

Copyright © National Academy of Sciences. All rights reserved.

have been ethical—but a comparison still needed to be made. So half of the volunteer participants were vaccinated immediately, and the other half after a three-week delay" (Farrar, 2015).

Not all of the scientists and ethicists involved in the conversations agreed with this reasoning, yet to many it appeared to represent an acceptable compromise between scientific rigor and the desire to offer the hoped-for benefits of the vaccine to as many as possible. To others it meant that the results might be difficult to interpret (Rid and Miller, 2016). The investigators planned the primary analysis to "estimate vaccine efficacy against disease [where] vaccine efficacy is defined as . . . the hazard [ratio] of disease for eligible and vaccinated individuals in a ring who receive immediate vaccination and eligible individuals in a ring who receive delayed vaccination" (Ebola ça Suffit Ring Vaccination Trial Consortium, 2015). However, during the course of the trial the data safety monitoring board (DSMB) concluded that the data were sufficiently convincing of vaccine protection and terminated the delayed vaccination arm (see further discussion below). The study continued to enroll additional participants who were all offered immediate immunization; the importance of this, going forward, meant that there was no longer a control arm in the trial (Henao-Restrepo et al., 2016).

Advantages and Disadvantages

The trial focused on persons at elevated risk of contracting Ebola because of contact with an infected individual, such as health care or burial workers—or contacts of such contacts, so fewer persons needed to be enrolled to demonstrate possible efficacy (Henao-Restrepo et al., 2015). However, given that only 21 days passed between the administration of the vaccine to the immediate vaccination group and to the delayed-vaccination control group, there had to be a high enough risk in order for the study to show results—that is, if people in the delayed-vaccination group were not infected soon enough to develop symptoms of Ebola within the 21-day delay period, it would be difficult to show that the vaccine was effective. The delay period began 10 days after the immediate group received vaccine and ended 10 days after the delayed group received the vaccine. The decision on this timing represented a rational attempt to respond to the major challenge to the design and to balance the desire of the investigators to immunize all participants within a reasonable time frame and the desire to have a long enough exposure in the delayed group to increase the likelihood that endpoints might be reached within the 21-day incubation period for Ebola virus (WHO, 2016a). The danger was that not enough events would occur within this window to permit an assessment of vaccine efficacy, the essential goal of the study.

Copyright © National Academy of Sciences. All rights reserved.

Another drawback to the design was that cluster randomization is less efficient than an individually randomized design and therefore that "the sample size must be inflated for the effect of clustering within rings as the members of a ring share a common exposure to the index case and are not statistically independent"(Ebola ça Suffit Ring Vaccination Trial Consortium, 2015). Moreover, the intrinsic risk of transmission within a cluster is assumed to be similar across clusters, but this may not be the case. As indicated in the study results, cases were documented in only 7 of 42 clusters in the delayed arm, and across these 7 clusters, per-person transmission risk also varied markedly (Henao-Restrepo et al., 2015). (See Table 4-3 below for the interim trial data.) Slight imbalances in intrinsic

TABLE 4-3 Calculations of Vaccine Efficacy and Vaccine Effectiveness Based on Different Study Populations—Guinea Ring Vaccine Trial

	All Vaccinated in Immediate Versus All Eligible in Delayed (primary analysis)	All Eligible and Consented	All Eligible (eligible adults, contacts and contacts of contacts)	All (all contacts and contacts of contacts)
**Number of individuals (clusters)**				
Immediate	2014 (48)	2048 (48)	3035 (48)	4123 (48)
Delayed	2380 (42)	1930 (42)	2380 (42)	3528 (42)
**Number of cases at <10 days (affected clusters)**				
Immediate	9 (4)	10 (5)	18 (9)	21 (9)
Delayed	16 (12)	6 (5)	16 (12)	25 (13)
**Number of cases at ≥10 days**				
Immediate	0 (0)	0 (0)	6[a] (3)	8[a] (4)
Delayed	16[b] (7)	11[b] (5)	16[b] (7)	21[b] (7)
Vaccine efficacy/ effectiveness[c] (%; 95% CI)	100% (74.4 to 100)	100% (70.8 to 100)	75.1% (–7.1 to 94.2)	76.3% (–15.5 to 95.1)
p value[d]	0.0036	0.0194	0.1791	0.3351

[a] All cases occurred in unvaccinated individuals.

[b] Four cases were vaccinated and developed symptoms on day 0, 2, 6, or 6 after vaccination.

[c] From fitting a β-binomial distribution to the cluster-level numerators and denominators and using an inverted likelihood ratio test to identify the lower bound for vaccine efficacy (first two columns); from Cox proportional hazards model to estimate vaccine effectiveness (last two columns).

[d] From Fisher's exact test (two-sided).

SOURCE: Henao-Restrepo et al., 2015.

Copyright © National Academy of Sciences. All rights reserved.

Integrating Clinical Research into Epidemic Response: The Ebola Experience

transmission risk across clusters, especially if the number of clusters is low, may lead to the primary comparisons having biased results. The lack of a placebo and the lack of blinding also raise concerns of possible bias in the ascertainment of safety endpoints, which could have significantly influenced the data presented. Additionally, if the study team was convinced that the vaccine was effective, it might at the very least, however unintentional, raise the possibility that efforts might have been less intense to detect and report events in the clusters randomized to immediate vaccination. It should also be noted that the logistical considerations of the ring vaccination trial are complex, with numerous trial sites across a large geographic area (Logistical considerations for the trials are discussed further in Chapter 5.)

Results and Discussion

The results were released in two publications (Henao-Restrepo et al., 2015, 2016). The first, designated the interim analysis, was published in July 2015 and included the data from the original cluster-randomized study design, immediate versus delayed immunization (Table 4-3), which had been collected up to the point at which the DSMB decided to terminate the delayed-immunization arm. The DSMB action was based on the emerging evidence from the trial that the vaccine was safe and effective as well as on the reality that the numbers of new ring-defining index cases were rapidly decreasing, which led the DSMB to conclude that it "would be unethical to deny people access to this life-saving intervention when the interim analysis showed evidence that rVSV-ZEBOV is both safe and effective" (UF, 2015). In the first publication the authors report, "The results of this interim analysis indicate that rVSV-ZEBOV might be highly efficacious and safe in preventing Ebola virus disease and is most likely effective at the population level when delivered during an Ebola outbreak via a ring vaccination strategy" (see data analysis below) (Henao-Restrepo et al., 2015, p. 857).

The second publication, designated the final analysis, appeared in December 2016 and included all data in the interim analysis as well as the additional data collected after the DSMB acted to terminate the delayed arm. Overall, there was a total of 64 laboratory-confirmed Ebola infections among participants eligible for randomization in the 96 randomized clusters. Of these, 41 had symptom onset before day 10 post-randomization (i.e., on days 0–9), including 20 of 3,232 participants in 9 of the 51 clusters randomized to immediate vaccination and 21 of 3,096 participants in 14 of the 47 clusters randomized to delayed vaccination. These data are indicative of a real, though variable, exposure to Ebola infection among contacts of the index person in the clusters (Henao-Restrepo et al., 2016). Among the remaining 23 Ebola cases with symptom onset 10 or more days after randomization (i.e., the primary endpoint of the study), 7 occurred in 4 of

Copyright © National Academy of Sciences. All rights reserved.

the 51 clusters randomized to immediate vaccination and 16 were identified in 7 of the 47 clusters randomized to delayed vaccination. However, in the immediate clusters, all 7 Ebola primary events occurred among eligible participants who actually did not receive the vaccine, whereas none were seen among the 2,108 persons immediately vaccinated. The additional data collected after the delayed arm was terminated supported the finding of an apparent protective effect. Among 1,677 persons in 19 additional nonrandomized clusters that were immediately vaccinated, there were no cases of Ebola with symptom onset 10 or more days after vaccination (Henao-Restrepo et al., 2016) (see Table 4-4). The investigators used multiple analytic strategies to probe the data, and they included these in the two resulting publications (see Tables 4-3 and 4-4).

While the data indicate that this vaccine provides protection from Ebola, there are differing estimates of the vaccine's efficacy depending on the analytical approach employed. If all persons eligible for vaccination within each of the clusters were included in the analysis, consistent with the intention-to-treat principle,[3] the trial was inconclusive (i.e., 7 of 3,212 eligible persons in immediate clusters with a primary endpoint versus 16 of 3,075 in delayed clusters who were eligible for vaccination and ascertainment of the primary endpoint, for a vaccine effectiveness of 65 percent, 95% confidence interval, –47–91%). In the final report, the investigators concluded, "The results add weight to the interim assessment that rVSV-ZEBOV offers substantial protection against Ebola virus disease, with no cases among vaccinated individuals from day 10 after vaccination in both randomised and non-randomised clusters" (Henao-Restrepo et al., 2016, p. 2). However, in the clusters randomized to immediate vaccination, approximately two-thirds (2,108/3,212) of eligible persons actually got the vaccine (Henao-Restrepo et al., 2016). When an "on-treatment" analysis was applied to those in the immediate vaccination clusters who were actually vaccinated and this subset of participants was compared to all eligible in the delayed vaccination clusters, the trial results now showed statistically significant benefits (0 of 2,108 vaccinated persons in immediate clusters with a primary endpoint versus 16 of 3,075 persons in delayed clusters eligible for vaccination and a primary endpoint; vaccine efficacy 100 percent, 95% confidence interval, 69–100%).

[3] Intention-to-treat (ITT) analysis. ITT analysis includes every subject who is randomized according to randomized treatment assignment. It ignores noncompliance, protocol deviations, withdrawal, and anything that happens after randomization. ITT analysis maintains prognostic balance generated from the original random treatment allocation. In ITT analysis, the estimate of treatment effect is generally conservative. A better application of the ITT approach is possible if complete outcome data are available for all randomized subjects. The per-protocol population is defined as the subset of the ITT population who completed the study without any major protocol violations (Gupta, 2011).

Copyright © National Academy of Sciences. All rights reserved.

TABLE 4-4 Effect of Vaccine on Cases of Ebola Virus Disease in Different Study Populations—Guinea Ring Vaccine Trial

	All Clusters[a]			
	1	2	3	4
	All vaccinated in immediate (group A) versus all contacts and contacts of contacts in delayed plus all never-vaccinated in immediate or nonrandomized (group B)	All vaccinated in immediate (group A) versus all eligible in delayed plus all eligible never-vaccinated in immediate (group B)	All contacts and contacts of contacts in immediate (group A) versus delayed (group B)	All vaccinated in immediate (group A) versus all eligible never vaccinated in immediate (group B)
Group A				
Number of individuals (clusters)	3775 (70)	3775 (70)	7241 (70)	3775 (70)
Cases of Ebola virus disease (clusters affected)	0 (0)	0 (0)	12 (7)	0 (0)
Attack rate	0%	0%	0.17%	0%
Group B				
Number of individuals (clusters)	7995 (116)	4507 (104)	4529 (47)	1432 (57)
Cases of Ebola virus disease (clusters affected)	34 (15)	23 (11)	22 (8)	7 (4)
Attack rate	0.43%	0.51%	0.49%	0.49%
Vaccine effect				
Vaccine efficacy/ effectiveness[c] (%, 95% CI)	100% (77.0 to 100.0)	100% (79.3 to 100.0)	70.1% (–4.9 to 91.5%)	100% (–51.5 to 100.0)
p value[d]	0.0012	0.0033	0.2759	0.125

[a] Randomly assigned and nonrandomly assigned individuals who were allocated to immediate vaccination were combined.

[b] Nonrandomized immediate clusters are excluded from this analysis.

[c] From fitting a β-binomial distribution to the cluster-level numerators and denominators and using an inverted likelihood ratio test to identify the lower bound for vaccine efficacy (columns 1, 2, 5, and 6); from a Cox proportional hazards model (columns 3, 7, and 8); from signed test (two-sided): probability of observing endpoints in control groups among

Copyright © National Academy of Sciences. All rights reserved.

Integrating Clinical Research into Epidemic Response: The Ebola Experience

Randomized Clusters[b]			
5	6	7	8
All vaccinated in immediate (group A) versus all eligible and consented on day 0 visit in delayed (group B)	All vaccinated in immediate (group A) versus all eligible in delayed (group B)	All eligible in immediate (group A) versus all eligible in delayed (group B)	All contacts and contacts of contacts in immediate (group A) versus all contacts and contacts of contacts in delayed (group B)
2108 (51)	2108 (51)	3212 (51)	4513 (51)
0 (0)	0 (0)	7 (4)	10 (5)
0%	0%	0.22%	0.22%
1492 (46)	3075 (47)	3075 (47)	4529 (47)
20 (4)	16 (7)	16 (7)	22 (8)
0.7%	0.52%	0.52%	0.49%
100% (63.5 to 100.0)	100% (68.9 to 100.0)	64.6% (–46.5 to 91.4)	64.6% (–44.2 to 91.3)
0.0471	0.0045	0.344	0.3761

treatment–control mismatched pairs and under the null hypothesis that the vaccine has no efficacy (column 4).

[d] From Fisher's exact test (two-sided), which is approximate for columns 1 and 2. From signed test (two-sided): the probability of observing endpoints in control groups among treatment–control mismatched pairs and under the null hypothesis that the vaccine has no efficacy (column 4).

SOURCE: Henao-Restrepo et al., 2016.

Copyright © National Academy of Sciences. All rights reserved.

In late December 2016 the WHO announced, "An experimental Ebola vaccine was highly protective against the deadly virus in a major trial in Guinea, according to results published today in *The Lancet*. The vaccine is the first to prevent infection from one of the most lethal known pathogens, and the findings add weight to early trial results published last year" (WHO, 2016c). Coverage in the press, however, focused on the statistically significant results from the "on-treatment" analyses, which demonstrated 100 percent vaccine efficacy. For example, Donald McNeil of *The New York Times* wrote: "In a scientific triumph that will change the way the world fights a terrifying killer, an experimental Ebola vaccine tested on humans in the waning days of the West African epidemic has been shown to provide *100 percent protection* against the lethal disease" (McNeil, 2016). Echoing the WHO press release, Sarah Boseley of *The Guardian* observed that the vaccine was "highly effective against one of the most lethal known pathogens in existence. Ten days after vaccination, none of the trial subjects developed Ebola virus disease" (Boseley, 2016).

It may appear rational to compare only those who were randomized to the immediate group and actually received the vaccine to the entire delayed group because an individual can only be protected if he or she receives the vaccine. However, while this "as-received" analysis is intended to measure vaccine efficacy, it is likely to be a biased estimate of vaccine efficacy, as discussed below. In contrast, the intention-to-treat analysis, which includes the entire "as-assigned" group, provides an unbiased estimate of efficacy; in this case, however, the estimate is substantially diluted due to the inclusion of those who did not receive the vaccine although this is likely more representative of overall clinical effectiveness. Additional observational analyses reported by the investigators—for example, no cases of Ebola having occurred among those vaccinated in nonrandomized clusters—provide further suggestive evidence of vaccine efficacy, although it is pertinent that the epidemic was already waning by the time the nonrandomized clusters were defined, at which point the risk of infection was substantially reduced.

The committee has devoted considerable attention to these different analyses and what they imply. We concur that, taken together, the results suggest that the vaccine most likely provides some protection to recipients—possibly "substantial protection," as stated in the final report. However, we remain uncertain about the magnitude of its efficacy, which could in reality be quite low or even zero, as the confidence limits around the unbiased estimate include zero. The reason for this uncertainty is that the primary comparison reported by the investigators is no longer protected by randomization because those who accepted vaccination in the immediate clusters are being selected for inclusion post-randomization. The main potential—but unmeasured—bias of this approach is that the individuals who received the vaccine may have had a lower chance of acquisition of

Copyright © National Academy of Sciences. All rights reserved.

infection (e.g., different risk of exposure to the virus) than those who were not immunized. If those who did not get the vaccine were more likely to be exposed, then by excluding them from the analysis but not excluding a comparable subset from the group assigned to delayed vaccination we would bias our results in favor of the immediate vaccination group. This is why the primary analysis in any RCT, including a cluster-randomized RCT, is almost always intention to treat, following the "once randomized, always analyzed" dictum (Hennekens et al., 1987). In addition, as noted earlier, the small proportion of clusters in which Ebola cases were reported raises a concern about the comparability of risk across clusters. Increasing the number of clusters in future similar types of trials will be required to minimize the possibility of disproportional allocation of clusters with different intrinsic transmission probability.

Due to safety and logistical concerns no serologic data were collected during the conduct of the trial, so no immunological correlate of protection from the vaccine can be determined (Henao-Restrepo et al., 2016). This is unfortunate because the establishment of such a correlate of protection would provide a benchmark, and other existing or newly designed vaccines could be compared with the product used in the current study. Long-term follow-up will also be required to ascertain the duration of protection and the potential need for future booster doses. The PREVAIL study is expected to provide data on the immune responses to this vaccine and their persistence, but not on the correlates of protection. Although there were only two serious adverse events (one febrile reaction and one case of anaphylaxis) attributed to the vaccine among the nearly 10,000 subjects vaccinated in the ring trial, the detection of less common adverse events would require a larger sample size (Henao-Restrepo et al., 2016). Fever, arthritis, and rash were associated with the vaccine in several patients in the initial Phase 1 trial, but there were no such reports in the ring trial (Agnandji et al., 2016). Although the safety profile is encouraging, further studies of the rates of these reactions and their potential pathogenesis are needed. It was not possible to compile longer-term safety data comparing the vaccinated and the control groups in the ring trial since all the control subjects were vaccinated. But because the same vaccine was used in the PREVAIL study; the committee believes that additional useful safety information may become available as those results are analyzed over time (Davey, 2016).

The "on treatment" vaccine efficacy estimate of 100 percent has been widely reported, but the reports generally do not acknowledge the fact that no vaccine is—or ever likely will be—100 percent effective, whether because of such host factors as immunodeficiency states or immunogenetics based antigen unresponsiveness or because of extrinsic factors such as a very high infection inoculum size, which can overcome existing immunity (CDC, n.d.). Once the authors were informed by the DSMB that they had

Copyright © National Academy of Sciences. All rights reserved.

documented 100 percent vaccine efficacy in July 2015, randomization was discontinued. Immediate vaccination was thereafter offered to an additional 19 subsequently formed clusters, and reported as an observational study (Henao-Restrepo et al., 2016). None of the vaccinated persons developed Ebola disease. Had the DSMB and the authors applied a more conservative interpretation of the preliminary results, along the lines of what this committee thinks the data demonstrate, and thereby continued to randomize all remaining clusters, the power to demonstrate benefit would have increased. That aside, the high level of expected protection, based on the trial results, may make it more difficult to conduct a confirmatory controlled trial of sufficient size in the event of a future outbreak; we can expect that it will be considered unethical to deny the vaccine or delay its administration to any individuals who are at risk of infection. This will be reinforced if the PREVAIL study demonstrates long-lived antibody responses that are protective when studied in either in vitro or in passive immunization animal studies and also adds to the favorable safety profile in the ring vaccination trial. The latter study included persons with a relatively high exposure to the virus, for whom a greater degree of uncertainty regarding potential adverse effects might be more acceptable than in populations at lower or negligible risk. Additional benefit and risk assessments are important for refining the indications for vaccine use during a future Ebola outbreak because the risk–benefit determination may differ for those at high risk (contacts, health care workers, burial teams) versus members of the general public, who are at considerably lower risk. In addition, this vaccine may not be as effective against a different Ebola virus strain, which is another issue that needs to be evaluated. Given these constraints, future vaccine trials during another outbreak could focus on head-to-head comparisons of different dosing schedules of the rVSV-ZEBOV vaccine or on a comparison with other vaccine candidates for which there is sufficient preliminary safety and immunogenicity data. In such trials, the determination of a surrogate measure for protection and long-term follow-up for continued efficacy and safety assessment should be prioritized; and in the event of an epidemic the immediacy of the protection should also be prioritized.

These considerations aside, the ring vaccination study has provided important new information of value for any future response to an Ebola outbreak. To ensure the further development of the rVSV-ZEBOV vaccine, Gavi (previously the Global Alliance for Vaccines and Immunization) and Merck, the company producing the vaccine used in the ring vaccination trial, announced a partnership in January 2016 in which Gavi committed funding to "help Merck take the vaccine through licensure and WHO prequalification. . . . If approved, it would become one of the world's first licensed Ebola vaccines, and Gavi would be able to begin purchasing the vaccine to create a stockpile for future outbreaks" (Gavi, 2016). This move

Copyright © National Academy of Sciences. All rights reserved.

helps assure a market to manufacturers working in the rare and neglected disease space and ensures the vaccine will be available to those who need it. "Ensuring a vaccine will be available to protect people who might have missed out due to a market failure lies at the heart of what makes Gavi so important in global health," said Gavi Board Chair Dr. Ngozi Okonjo-Iweala. "It is our moral duty to ensure that people do not miss out simply because of where they are born or whether they can afford to pay" (Gavi, 2016).

Partnership for Research on Ebola Vaccines in Liberia—PREVAIL I; Liberia, cAD3-EBOZ, VSV-ZEBOV, Placebo

The PREVAIL vaccine trial was a partnership between the Ministry of Health of Liberia and the National Institute of Allergy and Infectious Diseases of the U.S. National Institutes of Health and was conducted in Liberia in early 2015 to compare the safety, efficacy, and immunogenicity of two candidate vaccines, ChAd3-EBO-Z and VSVDG-ZEBOV, versus a saline placebo (Kennedy et al., 2016). The trial was slated to be the largest trial performed during the epidemic, with a planned enrollment of 28,000 participants—however, only 1,500 patients were ultimately enrolled, and it was the only individually randomized, double-blind, placebo-controlled vaccine trial conducted during the outbreak (Doe-Anderson et al., 2016). A timeline of the PREVAIL I trial can be found in Figure 4-2.

Study Design

The study was designed to allow a seamless transition from Phase 2 to Phase 3, and it used a common control group to assess the efficacy of the two candidate vaccines; i.e., the subjects given ChAd3-EBO-Z, VSVDG-ZEBOV, or a saline placebo were in a one-to-one-to-one ratio. Participant follow-up visits were originally planned at 1 week, 1 month, and 2 months, and then at 2-month intervals until the close of the study (Kennedy et al., 2016). However, with the outbreak rapidly coming under control, it was clear that the Phase 3 study to assess vaccine efficacy as well as safety could not be undertaken. Instead, based on the recommendation of the U.S. Food and Drug Administration (FDA) and with the concurrence of the independent DSMB and the two scientific and ethics review boards of record, the Phase 2 substudy was expanded to 1,500 participants; enrollment in it was completed within 3 months. Shortly before the study ended, the protocol was amended to also include a week 2 follow-up visit to specifically evaluate these participants for joint problems. As of December 2016, the study is still ongoing, although not recruiting additional volunteers (NIH and NIAID, 2016). The researchers state,

Copyright © National Academy of Sciences. All rights reserved.

Integrating Clinical Research into Epidemic Response: The Ebola Experience

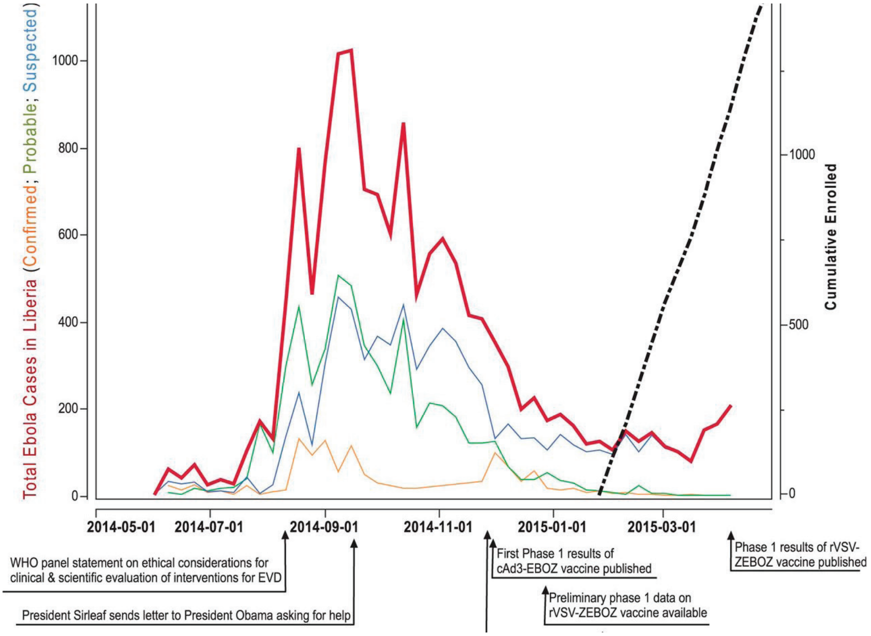

FIGURE 4-2 Timeline of PREVAIL trial. Total Ebola cases per week reported in Liberia are plotted in red, based on the reports from the Ministry of Health. Lines in orange, green, and blue show the breakdown of weekly reported cases that were classified as confirmed, probable, or suspected, respectively. On the right, the black dotted line shows the cumulative enrollment in the PREVAIL I vaccine trial, scaled to the axis on the right side of the graph. Below the figure, important events are shown to give a sense of the timeline as the epidemic unfolded.
SOURCE: Kennedy et al., 2016.

> The plan is to extend the follow-up of the original cohort of PREVAIL study participants to conduct long-term immunogenicity testing and collection of severe adverse events . . . for an additional 1 year after the original 12-month visit with the schedule of these follow-up visits determined from the date of vaccination. In order to understand the durability of the antibody response, follow-up may be continued for an additional 3 years (i.e., 5 total years post-vaccination date) to measure IgG antibody levels against the Ebola surface glycoprotein if after a total of 2 years post-vaccination follow-up, there is evidence that the antibody response has not substantially waned. (PREVAIL, 2016)

An interesting facet of the design is that had the outbreak continued, or if the trial had begun a few months earlier, the Phase 2 trial would have been seamlessly incorporated into a Phase 3 trial. The participants and the

Copyright © National Academy of Sciences. All rights reserved.

data that were already collected on efficacy and safety would have been included in the continuing Phase 3 study with the sample size enlarged to ensure that sufficient power was available to assess efficacy, investigate the possibility of enhancing antibodies, and evaluate both short- and long-term safety. Information from the Phase 2 laboratory evaluation would also be used to guide which data to capture in this larger cohort (Kennedy et al., 2016).

Advantages and Disadvantages

The target population for the trial was the general population rather than groups of higher-risk individuals such as health care workers, burial workers, or contacts of identified cases, as had been the focus of other studies (Henao-Restrepo et al., 2015; Widdowson et al., 2016). This focus on the general population made the study's circumstances similar to how the vaccine might be used in the future, and it allowed adverse effects of the vaccine to be detected more easily. In a situation of a larger Ebola outbreak it is likely that the vaccine will be offered more widely than to those at highest risk of contracting the infection. Additionally, given the collection of routine blood analysis and placebo design, it allowed for easier detection of adverse effects of the vaccine. A downside, however, is the larger sample size required when including an overall lower-risk population.

Only adults were enrolled initially because of concerns about safety. Had the trial continued, the data from this trial and others would have been used to evaluate amending the protocol to allow children to participate. Of note, the Phase 3 section of the trial was event driven, so that if transmission within the trial's cohort was higher than what had been estimated conclusive results might have been achieved with fewer enrollees (Pierson, 2015).

It was decided that the PREVAIL I serology tests would be performed at the Liberian Institute of Biomedical Research (LIBR) as part of a commitment to strengthen research capacity at LIBR. The LIBR laboratory continues to support the PREVAIL research program. PREVAIL analyzed samples with both (1) a commercially available enzyme-linked immunosorbent assay (ELISA) from Alpha Diagnostics International for the detection and quantification of immunoglobulin-G (IgG) against Ebola virus (EBOV) glycoprotein (GP) and (2) the Filovirus Animal Nonclinical Group test for IgG to EBOV GP; however, to date neither of these assays has been validated.[4]

[4] Personal communication, Jerome F. Pierson, Chief, Regulatory Compliance & Human Subjects Protection Branch, National Institute of Allergy and Infectious Disease (NIAID), National Institutes of Health (NIH). October 2016.

Copyright © National Academy of Sciences. All rights reserved.

Integrating Clinical Research into Epidemic Response: The Ebola Experience

Results and Discussion

The data from the PREVAIL I trial indicate that the two tested vaccines are safe and immunogenic. Interim data on the long-term serologic responses were presented at the 8th International Symposium on Filoviruses held in Antwerp, Belgium, on September 12–15, 2016 (Davey, 2016). During the first year of the study the rate of follow-up has consistently exceeded 95 percent; this serves to minimize any bias due to drop-outs and loss of information. The serologic data are summarized in Table 4-5 for the noted time intervals. The antibody response peaked 1 month after vaccination and was sustained over the next 11 months, without any clear evidence of decline for the rVSΔG group; 70 to 80 percent of the cohort responded to the vaccination with an antibody response (i.e., more than two standard deviations of response in the placebo group). Although the immune responses in the rVSΔG group were significantly higher than those to the ChAd3 group at all post-immunization time points, these data have only appeared in an abstract form and will need to be reassessed when published. The two actively vaccinated groups reported an excess risk of injection site reactions at 1 week (29 percent and 30 percent versus 7 percent), but not at the 1 month follow-up visit, compared to placebo. No excess risk of other clinical events was noted (Davey, 2016). The results as outlined document a robust antibody response to both of the vaccines tested that is maintained over a 12-month follow-up period, without evidence of adverse reactions other than the expected local injection site reactions.

Interestingly, at the beginning of the trial, 6.3 percent of enrollees were found to have pre-existing Ebola antibodies, possibly indicative of past Ebola infection (Davey, 2016). Additional investigations will be required to assess whether this is cross reactivity with shared antigens of other viruses or actual asymptomatic infections with Ebola virus and, if so, whether these might confer immunity to Ebola. On the basis of this information, Ebola virus may have been circulating in West Africa in advance of the outbreak, either unrecognized as Ebola or perhaps as asymptomatic infections. The follow-up of this is important for understanding the geographic boundaries of Ebola virus and the possibility of subclinical infection.

Sierra Leone Trial to Introduce a Vaccine Against Ebola (STRIVE); Sierra Leone, VSV-ZEBOV

The STRIVE trial was a collaboration among the College of Medicine and Allied Health Sciences of the University of Sierra Leone, the Sierra Leone Ministry of Health and Sanitation, and the CDC. The study involved health care workers and other frontline workers at greater risk of Ebola infection because of their increased exposure. It was also intended to

Copyright © National Academy of Sciences. All rights reserved.

TABLE 4-5 Preliminary Antibody Responses Following Vaccination—PREVAIL I Trial

	Antibody Responses Following Vaccination			P-value	
	ChAd3	rVSVΔG	Placebo	ChAd3 Versus rVSVΔG	rVSVΔG Versus Placebo
Week 1					
Number of participants	478	477	471		
GMT (95% CI)	88 (82–95)	83 (76–89)	74 (69–80)	<0.001	0.004
Responders (%; 95% CI)	3.6 (1.9–5.2)	2.5 (1.1–3.9)	1.5 (0.4–2.6)	0.06	0.36
Month 1					
Number of participants	476	473	468		
GMT (95% CI)	621 (565–682)	1000 (910–1099)	75 (69–80)	<0.001	<0.001
Responders (%; 95% CI)	70.8 (66.7–74.9)	83.7 (80.4–87.1)	2.8 (1.3–4.3)	<0.001	<0.001
Month 6					
Number of participants	460	447	432		
GMT (95% CI)	598 (547–654)	797 (727–874)	88 (81–96)	<0.001	<0.001
Responders (%; 95% CI)	72.4 (68.3–76.5)	78.5 (74.7–82.3)	6.3 (4.0–8.5)	<0.001	<0.001
Month 12					
Number of participants	452	442	435		
GMT (95% CI)	478 (442–517)	797 (733–867)	90 (84–97)	<0.001	<0.001
Responders (%; 95% CI)	63.3 (58.8–67.7)	78.7 (74.9–82.6)	6.9 (4.5–9.3)	<0.001	<0.001

NOTES: GMT = Geometric Mean Titer.
P-values for group comparisons of GMT based on $\log_{10}$ titer values at visit with baseline $\log_{10}$ titer as a covariate in analysis of covariance.
P-values for group comparisons of % responders based on Fisher's exact test.
Responders defined as change in $\log_{10}$ titer >2×SD of the change in placebo group at month 1, including participants without elevated antibody levels at entry.
SOURCE: Davey, 2016.

Copyright © National Academy of Sciences. All rights reserved.

Integrating Clinical Research into Epidemic Response: The Ebola Experience

strengthen the existing research capacity of institutions in Sierra Leone by providing training and research experience to hundreds of Sierra Leonean staff. Infrastructure was expanded, including renovating existing structures and building new structures to be able to enroll and vaccinate participants, handle data management, and store the vaccine. New technology was also introduced to maintain the cold chain for vaccine storage (Widdowson et al., 2016).

Study Design

The trial was initially designed as a stepped-wedge study, but it shifted to a more traditional individually randomized trial with a delayed vaccination arm. In the initial design the plan was to offer the vaccine to everyone in the study in a sequential manner, using the unvaccinated time as a comparator for the vaccinated time. However, researchers found that this design was too complex to carry out in the local setting and did not allow for the flexibility required to go into new places as the epidemic moved. The project was therefore converted to an unblinded but individually randomized trial, in which individuals were randomized to receive the vaccine immediately or to receive vaccine 18 to 24 weeks later.[5] The primary end point was laboratory-confirmed Ebola infection, and there were no futility stopping rules, although an interim analysis was planned.

Advantages and Disadvantages

Eligible participants included all health care workers or related workers involved in Ebola care and who were 18 years and older; women who were pregnant or breastfeeding were not enrolled. Subjects were followed for 6 months after vaccination. Detailed safety surveillance was prioritized for the first 400 subjects. An additional 500 subjects underwent immunogenicity studies at baseline and at three additional times during the study. During the conduct of the Phase 1 studies in Europe, skin rash and arthralgia were seen in some participants beginning the second week after vaccination (Regules et al., 2017). Given these findings, STRIVE leaders modified the suspected Ebola case definition for trial participants for the first 48 hours to avoid potential confusion with adverse reactions to the

[5] Presentation of Anne Schuchat, CDC, Clinical trial designs for emerging infectious diseases. U.S. Food and Drug Administration, Bethesda, MD. November 9, 2015.

Copyright © National Academy of Sciences. All rights reserved.

vaccine (Widdowson et al., 2016).[6] Given the interim results for the ring vaccination study, 100 individuals who were randomized to the late phase of the study were instead given the vaccine early.

There were several potential ethical issues with the trial, as acknowledged by study staff (Widdowson et al., 2016). First, the widespread fear of Ebola could skew the risk–benefit calculation by health care and frontline workers and push them toward accepting a vaccine of unknown safety and efficacy. Second, participants were reimbursed for participation and received free health care, which could have induced some to enroll. "These ethical and communication concerns were addressed with guidance from Sierra Leone STRIVE leadership and other partners. Active and transparent communication of risks and benefits to participants and the public continued throughout the trial as the risk–benefit balance changed with ebbing Ebola incidence" (Widdowson et al., 2016, p. 100). The rVSV-ZEBOV vaccine is an investigational new drug (IND), and STRIVE was conducted under an approved IND protocol, with the intent to include data from it in a biologics licensing application to the FDA.

Study Results and Discussion

As of November 2016, sera from around 500 STRIVE participants had been collected at baseline and at 1 month, 6 months, and 9–12 months after vaccination, with more than 80 percent follow-up at final time points. As agreed by the CDC and the Sierra Leonean collaborators, these sera have been shipped to the United States for study. Testing of the sera is pending validation of the GP-ELISA assay by the FDA. The CDC decided early on that the STRIVE serology should be conducted using a validated assay so that the results can be included in an application for vaccine licensure.[7]

Although still incomplete, the safety data are reassuring. "The safety sub-study enrolled 453 participants (227 immediate vaccines and 226 deferred vaccines) in April 2015. As of April 28, 2016, a total of 64 participants had illnesses that were investigated as suspected Ebola, of whom

[6] Standard suspected Ebola case definition: temperature ≥38°C (≥100.4°F) and three or more of the following symptoms: headache, loss of appetite, fatigue, muscle/joint pain, diarrhea, unusual bleeding, difficulty breathing, nausea, vomiting, abdominal pain, difficulty swallowing, or hiccups; OR illness after direct, unprotected Ebola contact or a breach in personal protective equipment in the past 21 days. Modified case definition applied to vaccine recipients in the first 48 hours after vaccination: same as for standard suspected Ebola case except that at least one symptom had to be one of the following symptoms not consistent with a vaccine reaction: diarrhea, unusual bleeding, difficulty breathing, nausea, vomiting, abdominal pain, difficulty swallowing, or hiccups (Widdowson et al., 2016).

[7] Personal communication, Barbara Mahon, CDC lead, Sierra Leone Trial to Introduce a Vaccine Against Ebola (STRIVE). October 2016.

Copyright © National Academy of Sciences. All rights reserved.

60 provided specimens for testing, but none were confirmed as Ebola. No serious adverse events related to vaccination have been reported; the data from the safety sub-study are generally consistent with data found in Phase 1 trials of the vaccine, and no association of vaccine with arthritis has been noted" (Widdowson et al., 2016, p. 104). Because cases were already declining when the trial began recruiting participants, and there were no subsequent cases among them, STRIVE was not able to determine vaccine efficacy or draw any conclusions regarding how well the vaccine would work in this population. The major contribution of the study was to expand safety information available for the rVSV-ZEBOV candidate vaccine, creating the largest safety database available on the vaccine and, ultimately, immunogenicity data.

EBOVAC–Salone; Sierra Leone, Ad26.ZEBOV and MVA-BN-Filo

The EBOVAC–Salone trial was originally designed as a large-cluster, randomized study in Sierra Leone to achieve and assess the efficacy of a prime boost vaccine approach. Phase 1 studies on safety and immunogenicity were conducted in Europe starting in late 2014 and in Africa starting in 2015 (EBOVAC, 2016). Phase 2 studies are also under way, with participants in France, the United Kingdom, Burkina Faso, Uganda, Kenya, Cote d'Ivoire, and Rwanda, including an age deescalation study to include young children (EBOVAC2, 2016). The EBOVAC projects are intended to determine the safety and tolerability of a prime boost vaccine regimen that was developed by Janssen Pharmaceutical Companies of Johnson & Johnson (EBOVAC, 2016). The prime boost approach is a two-step vaccination protocol in which participants are first given a dose of Ad26.ZEBOV vaccine to prime their immune system and then a dose of MVA-BN-Filo at a later point to further enhance the immune response and achieve long-lasting protection (EBOVAC, 2016). With enrollment starting in 2015, the study includes an active control arm, using the meningitis Men ACYW vaccine for one-third of the subjects in a randomized manner, to provide a control group for safety and immunogenicity analysis (NIH and Janssen Vaccines & Prevention B.V., 2016).

Study Design

The Phase 1 trial in the United Kingdom (the most comprehensive study site from which data are available) used a randomized, placebo-controlled, observer-blind design, enrolling only adults. Participants were randomized to receive either a placebo or to receive both Ad26.ZEBOV and MVA-BN-Filo in a different order and time interval, thus generating subgroups to evaluate (Milligan et al., 2016).

Copyright © National Academy of Sciences. All rights reserved.

Integrating Clinical Research into Epidemic Response: The Ebola Experience

Study Results and Analysis

The Phase 1 study in the United Kingdom found no serious vaccine related adverse events. All participants had specific IgG detectable at 21 days after the boost vaccine as well as at the 8-month follow-up. In the group that received Ad26.ZEBOV first, 97 percent showed an immune response after the primary immunization (Milligan et al., 2016). While these data do not provide information on the potential efficacy of the approach and the vaccines used, they indicate that the vaccines are promising candidates for further study in a future outbreak.

OVERALL ASSESSMENT OF VACCINES

One of the most remarkable successes of the vaccine trials was how rapidly they were planned, approved, and implemented. Under the pressure of the outbreak, the timelines for scientific and ethics approval were compressed. Protocol development was completed within a few weeks, and to address the requests from clinical trial investigators in Africa, Phase 1 studies were conducted in high-income countries (the United States and European countries) before the vaccine trials were launched in Africa. In fact, "[f]ive Phase 1 trials of ChAd3 and eight Phase 1 rVSV trials were initiated between September and December 2014 in North America, Europe, and Africa" (WHO, 2015c, p. 10). By February 2015, data were available from the Phase 1 trials to select vaccine dosing and to begin implementing Phase 2 and Phase 3 trials in Ebola-affected countries in Africa. These began only 6 months after the WHO declared the epidemic a PHEIC, with the PREVAIL I trial starting in February 2015 in Liberia and both the STRIVE trial in Sierra Leone and the ring vaccination trial in Guinea starting in March 2015 (WHO, 2015c). Conducting Phase 1 and 2 trials in countries not affected by the outbreak was thought to facilitate acceptance of larger trials in affected countries; however, given the persistent belief in the affected countries that foreign medical teams were possibly testing something dangerous it remained imperative that trial teams also focus on community engagement and communication of the clinical trial process. (Community engagement on the part of the trial teams is discussed further in Chapter 6.)

The vaccine trials conducted during the epidemic indicate there are promising Ebola vaccine candidates in terms of safety and immunogenicity. The study designs selected were generally appropriate for the context and question being explored—for example, implementing ring vaccination trials for high-risk populations and individual RCTs for the general population at lower risk in order to more fully assess safety. While the ring vaccination study showed suggestive efficacy, the trial was not designed to

Copyright © National Academy of Sciences. All rights reserved.

document long-term safety and efficacy because all participants were ultimately immunized and the protocol only followed participants out to day 84 (Henao-Restrepo et al., 2016). When the immunogenicity data become available, the results of the PREVAIL trial will provide information on the long-term immunogenicity of the vaccines, including the vaccine used in the ring vaccination study (PREVAIL, 2016). The differences in the study designs and the value of the information generated from each trial highlight the importance of collaboration in future trials. For example, if the ring trial had been the only one conducted during the outbreak, an unfortunate situation could have emerged; because of the initial suggestion of its high degree of efficacy equipoise could have been preemptively eliminated despite the estimate of protection from the intention-to-treat analysis being much lower. Additionally, the results from the ring vaccination trial provide limited safety data and no data on the duration of the immune response beyond 84 days; fortunately, the PREVAIL I trial can address these important gaps in knowledge. (For a summary of the vaccine trials conducted during 2014–2015, see Table 4-6 at the end of the chapter.)

Improving the implementation of vaccine trials in a future outbreak will require a mechanism to assess the pros and cons of the different vaccine trial approaches and a process to prioritize what to study among the available candidates. This is particularly important in advance of the next event because the length and severity of future epidemics cannot be predicted ahead of time and, therefore, rapid trial approval and implementation will be critical in order to generate conclusive results. For future outbreaks, it would be valuable to have a portfolio of trial designs in advance that have already been vetted among the key stakeholders and that are designed to suit different populations, including high-risk populations in direct contact with infected individuals as well as the lower-risk general population. Early in the 2014–2015 epidemic, there was insufficient coordination among the trial teams to prioritize vaccines to test in which population with what protocol, to harmonize data collection, or to select assays to analyze sera. As a consequence, with the outbreak winding down when the trials began, there was competition for enrollment and little standardization of data collection (including data on adverse events); standardized data collection is necessary so that information can be combined for the purpose of analysis.

In the event of a future outbreak, given the practical constraints on the ground, it may be more strategic and easier in practice to quickly launch one type of trial at the start of the epidemic in order to obtain initial information on investigational vaccines. The preliminary findings from the first trial could be used to inform the trial protocols of more robust subsequent trials already in development. To do this effectively would require pre-outbreak planning and coordination and should also include consideration of better and faster ways to undertake the clinical trial review and approval

Copyright © National Academy of Sciences. All rights reserved.

mechanisms within at-risk countries, shared experiences on best practices for fostering community engagement, and a discussion of lessons learned about the context in which randomized double-blind placebo or active-control arms can be accepted by the country and the community. There are also different scenarios in which the course of the outbreak and interim trial results may influence the trial designs and, ultimately, vaccine use during an outbreak. For example, during an epidemic with a new pathogen in which the general population is at high risk of infection and a ring trial shows initial efficacy, it may be reasonable to forgo planning a placebo-controlled trial in order to vaccinate the entire population. Alternatively, it may be preferable to move quickly to implement a placebo-controlled trial as an epidemic begins to wane and it becomes clear that a ring trial may not give definitive answers.

Agreement in principle on diverse issues such as sharing data and resources, intercomparability and interpretation of information, the launch of the trials, and standardization of data collection and assays used for analysis would speed up the design, approval, and initiation of well-thought-out studies. Much of this work can be initiated in advance of the next outbreak, pushing ahead to reach consensus among the key players if at all possible. Other issues to be dealt with in advance of an epidemic (which are beyond the scope of this report) include the manufacture of vaccines; access and distribution; affordability and the source of funding; and how to address liability issues and risk management.

> ***Conclusion 4-1*** *If research during future epidemics is to be conducted in a more efficient and effective manner, funders and sponsors of research need to plan well in advance, ideally during an inter-epidemic period, to coordinate efforts more closely and must agree to initiate clinical research during an outbreak in concordance with an overall research agenda.*

Copyright © National Academy of Sciences. All rights reserved.

TABLE 4-6 Summary of Ebola Vaccine Trials

Investigational Product	Sponsoring Organization, Trial Name	Trial Location	Trial Design and Design Considerations	Timeline	Results
rVSV-ZEBOV<br><br>NewLink Genetics and Merck Vaccines USA<br><br>(Henao-Restrepo et al., 2015, 2016)	WHO, Médecins Sans Frontiéres (MSF) and Government of Guinea in Conakry, Guinea—Guinea Ring Vaccine Trial<br><br>Ebola ça Suffit Trial	Guinea	Trial design<br>• open-label, cluster-randomized ring vaccination trial<br>• immediate versus deferred (21 days) vaccination<br>Design considerations<br>• Because of extensive field operational challenges—including community resistance, difficulty reaching remote field sites, and vaccine transportation at −80°C—the Ebola ça Suffit trial forgoes two of the routine practices of randomized controlled trials.<br>• There are no placebo vaccination visits for double blinding. To reduce the risk of bias arising from behavior changes that might follow vaccination, participants are informed that it is not known if the vaccine works and that they must still take steps to avoid infection.	April 1, 2015, and July 20, 2015	Summary of main findings: Randomization to assess vaccine efficacy was possible to implement during the Ebola outbreak. If cluster was used as unit for analysis, the trial was inconclusive. If individual persons eligible for vaccination within the clusters (intent to treat) were used as unit for the analysis, the trial was inconclusive (the 95 percent CI overlaps with 0). If an on-treatment analysis was applied (with all eligible used in control arm), trial results provide suggestion

Copyright © National Academy of Sciences. All rights reserved.

	• Rings are randomly allocated before individual informed consent is obtained. Although the consent teams are aware of allocation, making this de facto unconcealed, participants are told of their vaccination schedule only at the end of the informed consent process. Monitoring of recruitment to date has not indicated differences between study arms, though selection bias cannot be excluded.	for benefit. Potential harm from vaccination could not be evaluated from a review of the report as safety analyses were ongoing when published.

continued

Copyright © National Academy of Sciences. All rights reserved.

TABLE 4-6 Continued

Investigational Product	Sponsoring Organization, Trial Name	Trial Location	Trial Design and Design Considerations	Timeline	Results
rVSVΔG-ZEBOV<br><br>NewLink Genetics and Merck Vaccines USA<br><br>(FDA, 2015; Widdowson et al., 2016)	U.S. Centers for Disease Control and Prevention and MOH Sierra Leone; Sierra Leone Trial to Introduce a Vaccine Against Ebola (STRIVE)	Sierra Leone	Trial design<br>• Individually randomized, open label<br>• Immediate versus deferred vaccination (18–24 weeks after enrollment)<br>• Subjects were given only one dose of vaccine.<br>Design considerations<br>• Design was originally a step-wedge design in an attempt to design a trial that was a little bit simpler than an individually randomized placebo-controlled trial.<br>• Challenges with the need to enumerate everybody who's going to be in the trial at the beginning of the trial and enumerating health care workers in a place where all the health care facilities are closed limited the flexibility of the stepped-wedge design.<br>• Opted for a design that gave more flexibility—design used allowed the flexibility to not be ready everywhere before starting and permitted less complexity in the cold chain logistics and oversight.<br>• Primary endpoint was laboratory-confirmed EBOLA and there were no futility stopping rules. However, interim analysis was planned. (Include the endpoints of the substudies as secondary endpoints.)	April 9–August 21, 2015	Enrollment complete: 8,673 enrolled<br>• As of October 18, 2015, >8,016 participants vaccinated<br>• Of those vaccinated, 3,826 received delayed vaccination<br>• Safety profile consistent with other published studies<br>○ No safety signals in substudy<br>○ No vaccine-related serious adverse events<br>○ 8 deaths reported to date; none vaccine-related (estimate 43 deaths during study)<br>• A total of 539 participants enrolled in the immunogenicity study.

Copyright © National Academy of Sciences. All rights reserved.

- Of these, 509 provided baseline blood samples, of whom 466 (92 percent) provided a day-28 blood sample and 411 (81 percent) provided a 6-month blood sample. The blood draws for months 9–12 after vaccination began in June 2016.
- Serology is to be performed upon validation of an Ebola GP-ELISA assay.[a]

continued

Copyright © National Academy of Sciences. All rights reserved.

TABLE 4-6 Continued

Investigational Product	Sponsoring Organization, Trial Name	Trial Location	Trial Design and Design Considerations	Timeline	Results
ChAd3-EBOZ GlaxoSmithKline and PHAC<br><br>rVSV-ZEBOV NewLink Genetics and Merck Vaccines USA<br><br>(Davey, 2016; NIH and NIAID, 2016; Pierson, 2015; PREVAIL, 2016[b])	By U.S. NIH and MOH Liberia<br><br>Partnership for Research on Ebola Vaccines in Liberia (PREVAIL I)	Monrovia, Liberia	Trial design:<br>• Randomized, double-blind, placebo controlled<br>• 2 treatment arms (ChAd3-EBOZ or rVSV-ZEBOV), 1 placebo arm<br>• Randomized 1:1:1 to ChAd3-EBO-Z, VSVDG-ZEBOV, or saline placebo<br>• Following randomization, participants' visits were scheduled at 1 week, 1 month, 2 months, and every 2 months thereafter until the close of the study. At these visits, participants were asked questions to assess their health status and any unreported events, and blood samples were periodically collected.<br>• Shortly before the study ended, the protocol was amended to also include a week 2 follow-up visit to specifically evaluate these participants for joint problems.<br>Design considerations:<br>• Randomized, placebo-controlled trial provides most rapid route to identification of a safe and effective vaccine.<br>• Investigational products—thus, placebo-controlled trial allows a rigorous assessment of safety and efficacy.	February 2, 2015–April 30, 2015	The trial enrolled 1,500 men and women ages 18 and older with no reported history of Ebola virus disease at Redemption Hospital in Monrovia from February 2 through April 30, 2015. Three equal-sized groups of 500 received either one of the two vaccine candidates or a saline injection. Both vaccines were well tolerated. At 1 month, 87 percent of the volunteers who received the cAd3-EBOZ vaccine candidate had measurable Ebola antibodies; 94 percent of the volunteers who received the rVSV-ZEBOV vaccine had demonstrable antibodies after 1 month.

Copyright © National Academy of Sciences. All rights reserved.

	• Designs that will allow a rigorous assessment of safety and efficacy will provide confidence for future use if products are later used in wide-scale vaccination programs.	The results as outlined document a robust antibody response to either of the two vaccines tested, that is maintained over a 12-month follow-up period and without evidence of adverse drug reactions other than the expected local injecting site reactions.

continued

Copyright © National Academy of Sciences. All rights reserved.

TABLE 4-6 Continued

Investigational Product	Sponsoring Organization, Trial Name	Trial Location	Trial Design and Design Considerations	Timeline	Results
Ad26.ZEBOV and MVA-BN-Filo<br><br>Johnson & Johnson and Bavarian Nordic<br><br>(EBOVAC2, 2016; EBOVAC, 2016; Milligan et al., 2016; NIH and Janssen Vaccines & Prevention B.V., 2016)	Crucell Holland BV; MoH/ LSHTM<br><br>EBOVAC	Sierra Leone	Trial design<br>• This is a staged Phase 3 study to gather information on the safety and immunogenicity of a heterologous prime-boost regimen. In this regimen, the immune system is primed with the candidate vaccine Ad26.ZEBOV and later boosted with the candidate vaccine MVA-BN-Filo.<br>• The study is taking place in Sierra Leone and consists of a screening phase, an active phase (vaccination), and a follow-up phase. The active phase of the study is being conducted initially in two stages:<br>○ In the first stage approximately 40 adults ages 18 years or older were vaccinated to gain information about the safety and immunogenicity of the prime-boost regimen.<br>○ In stage 2 a larger group of approximately 688 individuals will be vaccinated to further evaluate the safety and immunogenicity of the prime-boost regimen across different age groups. In this stage, children ages 1 year or older, adolescents, and adults are included.	First dose of vaccine was given on October 8, 2015	

Copyright © National Academy of Sciences. All rights reserved.

- Safety data are being collected in stages 1 and 2, 7 days after the initial vaccination and boost vaccination. These data will be reviewed by an independent data monitoring committee to assess whether initiation of vaccination in the next stage or age group can be provided.
- Safety evaluations will include an assessment of adverse events, which will be monitored throughout the study. For stages 1 and 2, the follow-up will be 360 days after prime vaccination.

Design considerations:

- Study was initiated in parallel track to multiple ongoing Phase 1 and Phase 2 studies across United States, Europe, and Africa as part of accelerated development plan for vaccine regimen.
- The EBOVAC-Salone team's goal has been to conduct a study that meets Sierra Leone's Ebola prevention needs, has the support of the Sierra Leonean people, and can play a sustaining role in helping to restore the country's health infrastructure following the Ebola outbreak.
    - Significant investment has been made to build new facilities in Kambia to conduct the study, which will contribute substantially to the strengthening of the local health system.

continued

Copyright © National Academy of Sciences. All rights reserved.

TABLE 4-6 Continued

Investigational Product	Sponsoring Organization, Trial Name	Trial Location	Trial Design and Design Considerations	Timeline	Results
			○ These include establishing the first emergency room at the Kambia District Hospital and building a new vaccine storage facility on the hospital site.<br>○ These efforts are complemented by the employment and training of doctors, nurses and other frontline health care workers who will gain valuable experience while contributing to the clinical study.		

[a] Personal communication, Barbara Mahon, CDC lead, Sierra Leone Trial to Introduce a Vaccine Against Ebola (STRIVE). October 2016.

[b] Personal communication, Jerome F. Pierson, Chief, Regulatory Compliance & Human Subjects Protection Branch, National Institute of Allergy and Infectious Disease (NIAID), National Institutes of Health (NIH). October 2016.

Copyright © National Academy of Sciences. All rights reserved.

Integrating Clinical Research into Epidemic Response: The Ebola Experience

REFERENCES

Agnandji, S. T., A. Huttner, M. E. Zinser, P. Njuguna, C. Dahlke, J. F. Fernandes, S. Yerly, J.-A. Dayer, V. Kraehling, R. Kasonta, A. A. Adegnika, M. Altfeld, F. Auderset, E. B. Bache, N. Biedenkopf, S. Borregaard, J. S. Brosnahan, R. Burrow, C. Combescure, J. Desmeules, M. Eickmann, S. K. Fehling, A. Finckh, A. R. Goncalves, M. P. Grobusch, J. Hooper, A. Jambrecina, A. L. Kabwende, G. Kaya, D. Kimani, B. Lell, B. Lemaître, A. W. Lohse, M. Massinga-Loembe, A. Matthey, B. Mordmüller, A. Nolting, C. Ogwang, M. Ramharter, J. Schmidt-Chanasit, S. Schmiedel, P. Silvera, F. R. Stahl, H. M. Staines, T. Strecker, H. C. Stubbe, B. Tsofa, S. Zaki, P. Fast, V. Moorthy, L. Kaiser, S. Krishna, S. Becker, M.-P. Kieny, P. Bejon, P. G. Kremsner, M. M. Addo, and C.-A. Siegrist. 2016. Phase 1 trials of rVSV Ebola vaccine in Africa and Europe. *New England Journal of Medicine* 374(17):1647–1660.

Boseley, S. 2016. Ebola vaccine is safe and effective, scientists declare after trials. *The Guardian*, December 22. https://www.theguardian.com/world/2016/dec/22/ebola-vaccine-is-safe-and-effective-scientists-declare-after-trials-successful?CMP=share_btn_link (accessed January 27, 2017).

Brown, C. A., and R. J. Lilford. 2006. The stepped wedge trial design: A systematic review. *BMC Medical Research Methodology* 6:54.

CDC (U.S. Centers for Disease Control and Prevention). n.d. *What you need to know about vaccine safety.* https://www.cdc.gov/media/subtopic/matte/pdf/asd-vaccine-safety-matte.pdf (accessed January 25, 2017).

Cohen, J., and K. Kupferschmidt. 2014a. Ebola vaccine trials raise ethical issues. *Science* 346(6207):289–290.

Cohen, J., and K. Kupferschmidt. 2014b. Tough choices ahead in Ebola vaccine trials. *Science*, October 7. http://www.sciencemag.org/news/2014/10/tough-choices-ahead-ebola-vaccine-trials (accessed February 4, 2017).

Davey, R. T. 2016. PREVAIL I: A randomized controlled safety and immunogenicity trial of two different vaccines against Ebola virus. Paper read at 8th International Symposium on Filoviruses, September 12–15, 2016, Antwerp, Belgium.

Dawson, A. J. 2015. Ebola: What it tells us about medical ethics. *Journal of Medical Ethics* 41(1):107–110.

Doe-Anderson, J., B. Baseler, P. Driscoll, M. Johnson, J. Lysander, L. McNay, W. S. Njoh, M. Smolskis, L. Wehrlen, and J. Zuckerman. 2016. Beating the odds: Successful establishment of a Phase II/III clinical research trial in resource-poor Liberia during the largest-ever Ebola outbreak. *Contemporary Clinical Trials Communications* 4:68–73.

Ebola ça Suffit Ring Vaccination Trial Consortium. 2015. The ring vaccination trial: A novel cluster randomised controlled trial design to evaluate vaccine efficacy and effectiveness during outbreaks, with special reference to Ebola. *BMJ* 351:h3740.

EBOVAC. 2016. *Developing a vaccine for Ebola*. http://www.ebovac.org (accessed January 18, 2017).

EBOVAC2. 2016. *1st EBOVAC2 newsletter*. http://www.ebovac2.com/images/EBOVAC2_newsletter_no._1_March_2016.pdf (accessed January 18, 2017).

Farrar, J. 2015. *The Ebola vaccine we dared to dream of is here*. https://www.theguardian.com/commentisfree/2015/aug/03/ebola-vaccine-trials-diseases (accessed December 21, 2016).

FDA (U.S. Food and Drug Administration). 2015. FDA clinical trial designs for emerging infectious diseases. Paper read at FDA Clinical Trail Designs for Emerging Infectious Diseases, November 9, 2015.

Gavi. 2016. *Ebola vaccine purchasing commitment from Gavi to prepare for future outbreaks*. http://www.gavi.org/library/news/press-releases/2016/ebola-vaccine-purchasing-commitment-from-gavi-to-prepare-for-future-outbreaks (accessed January 25, 2017).

Copyright © National Academy of Sciences. All rights reserved.

Integrating Clinical Research into Epidemic Response: The Ebola Experience

Gupta, S. K. 2011. Intention-to-treat concept: A review. *Perspectives in Clinical Research* 2(3):109–112.

Henao-Restrepo, A. M., I. M. Longini, M. Egger, N. E. Dean, W. J. Edmunds, A. Camacho, M. W. Carroll, M. Doumbia, B. Draguez, S. Duraffour, G. Enwere, R. Grais, S. Gunther, S. Hossmann, M. K. Kondé, S. Kone, E. Kuisma, M. M. Levine, S. Mandal, G. Norheim, X. Riveros, A. Soumah, S. Trelle, A. S. Vicari, C. H. Watson, S. Kéïta, M. P. Kieny, and J.-A. Røttingen. 2015. Efficacy and effectiveness of an rVSV-vectored vaccine expressing Ebola surface glycoprotein: Interim results from the Guinea ring vaccination cluster-randomised trial. *The Lancet* 386(9996):857–866.

Henao-Restrepo, A. M., A. Camacho, I. M. Longini, C. H. Watson, W. J. Edmunds, M. Egger, M. W. Carroll, N. E. Dean, I. Diatta, M. Doumbia, B. Draguez, S. Duraffour, G. Enwere, R. Grais, S. Gunther, P.-S. Gsell, S. Hossmann, S. V. Watle, M. K. Kondé, S. Kéïta, S. Kone, E. Kuisma, M. M. Levine, S. Mandal, T. Mauget, G. Norheim, X. Riveros, A. Soumah, S. Trelle, A. S. Vicari, J.-A. Røttingen, and M.-P. Kieny. 2016. Efficacy and effectiveness of an rVSV-vectored vaccine in preventing Ebola virus disease: Final results from the Guinea ring vaccination, open-label, cluster-randomised trial (Ebola Ça Suffit!). *The Lancet* 389:505–518.

Hennekens, C. H., J. E. Buring, and S. L. Mayrent. 1987. *Epidemiology in medicine*. Boston: Little, Brown and Co.

Kennedy, S. B., J. D. Neaton, H. C. Lane, M. W. Kieh, M. B. Massaquoi, N. A. Touchette, M. C. Nason, D. A. Follmann, F. K. Boley, M. P. Johnson, G. Larson, F. N. Kateh, and T. G. Nyenswah. 2016. Implementation of an Ebola virus disease vaccine clinical trial during the Ebola epidemic in Liberia: Design, procedures, and challenges. *Clinical Trials* 13(1):49–56.

The Lancet. 2015. An Ebola vaccine: First results and promising opportunities. *The Lancet* 386(9996): 830.

McNeil, D. G. 2016. New Ebola vaccine gives 100 percent protection. *New York Times*, December 22. https://www.nytimes.com/2016/12/22/health/ebola-vaccine.html?_r=0 (accessed January 25, 2017).

Milligan, I. D., M. M. Gibani, R. Sewell, E. A. Clutterbuck, D. Campbell, E. Plested, E. Nuthall, M. Voysey, L. Silva-Reyes, M. J. McElrath, S. C. D. Rosa, N. Frahm, K. W. Cohen, G. Shukarev, N. Orzabal, W. v. Duijnhoven, C. Truyers, N. Bachmayer, D. Splinter, N. Samy, M. G. Pau, H. Schuitemaker, K. Luhn, B. Callendret, J. V. Hoof, M. Douoguih, K. Ewer, B. Angus, A. J. Pollard, and a. M. D. Snape. 2016. Safety and immunogenicity of novel adenovirus type 26- and modified vaccinia Ankara–vectored Ebola vaccines: A randomized clinical trial. *JAMA* 315(15):1610–1623.

Mohammadi, D. 2015. Ebola vaccine trials back on track. *The Lancet* 385(9964):214–215.

Nason, M. 2016. Statistics and logistics: Design of Ebola vaccine trials in West Africa. *Clinical Trials* 13(1):87–91.

NIH (U.S. National Institutes of Health) and Janssen Vaccines & Prevention B.V. 2016. *Staged Phase 3 study to assess the safety and immunogenicity of Ebola candidate vaccines Ad26.ZEBOV and MVA-BN-Filo during implementation of stages 1 and 2 (EBOVAC-Salone). Clinical Trial No. NCT02509494.* https://clinicaltrials.gov/ct2/show/record/NCT02509494 (accessed January 17, 2017).

NIH and NIAID (National Institute of Allergy and Infection Diseases). 2016. *Partnership for Research on Ebola Vaccines in Liberia (PREVAIL). Clinical trial no. NCT02344407.* https://clinicaltrials.gov/ct2/show/record/NCT02344407 (accessed January 18, 2017).

Openshaw, P. J. M., and J. S. Tregoning. 2005. Immune responses and disease enhancement during respiratory syncytial virus infection. *Clinical Microbiology Reviews* 18(3):541–555.

Copyright © National Academy of Sciences. All rights reserved.

Integrating Clinical Research into Epidemic Response: The Ebola Experience

Pierson, J. F. 2015. *Partnership for Research on Ebola Vaccines in Liberia: Presentation to the Vaccines and Related Biological Products Advisory Committee to the Center for Biologics Evaluation and Research of the Food and Drug Administration*. http://www.fda.gov/downloads/AdvisoryCommittees/CommitteesMeetingMaterials/BloodVaccinesandOtherBiologics/VaccinesandRelatedBiologicalProductsAdvisoryCommittee/UCM448001.pdf (accessed January 17, 2017).

PREVAIL (Partnership for Research on Ebola Vaccines in Liberia). 2016. *PREVAIL protocol* (version 5.0, 09 May 2016). Bethesda, MD: Office of Clinical Research Operations and Regulatory Compliance, Division of Clinical Research, National Institute of Allergy and Infectious Diseases, National Institutes of Health.

Regules, J. A., J. H. Beigel, K. M. Paolino, J. Voell, A. R. Castellano, P. Muñoz, J. E. Moon, R. C. Ruck, J. W. Bennett, P. S. Twomey, R. L. Gutiérrez, S. A. Remich, H. R. Hack, M. L. Wisniewski, M. D. Josleyn, S. A. Kwilas, N. Van Deusen, O. T. Mbaya, Y. Zhou, D. A. Stanley, R. L. Bliss, D. Cebrik, K. S. Smith, M. Shi, J. E. Ledgerwood, B. S. Graham, N. J. Sullivan, L. L. Jagodzinski, S. A. Peel, J. B. Alimonti, J. W. Hooper, P. M. Silvera, B. K. Martin, T. P. Monath, W. J. Ramsey, C. J. Link, H. C. Lane, N. L. Michael, R. T. J. Davey, S. J. Thomas, and the rVSVΔG-ZEBOV-GP Study Group. 2017. A recombinant vesicular stomatitis virus Ebola vaccine. *New England Journal of Medicine* 376(4):330–341.

Rid, A., and F. G. Miller. 2016. Ethical rationale for the Ebola "ring vaccination" trial design. *American Journal of Public Health* 106(3):432–435.

UF (University of Florida). 2015. *Biostat researchers discuss roles in ongoing Ebola vaccine trial*. epi.ufl.edu/news-and-events/epi-news/biostat-researchers-discuss-roles-in-ongoing-ebola-vaccine-trial.html (accessed February 4, 2017).

Underhill, K. 2013. Study designs for identifying risk compensation behavior among users of biomedical HIV prevention technologies: Balancing methodological rigor and research ethics. *Social Science & Medicine* 94:115–123.

WHO (World Health Organization). 2014. *Ebola virus disease in Guinea*. http://www.who.int/csr/don/2014_03_23_ebola/en (accessed January 17, 2017).

WHO. 2015a. *Call for proposals: Mapping of the WHO coordinated international effort to develop Ebola vaccines, treatments and diagnostics and process for the Ebola vaccine trial in Guinea*. http://www.who.int/medicines/ebola-treatment/call-for-proposals-int-effort-ebola-vax-trial/en (accessed February 20, 2017).

WHO. 2015b. *Guinea: Ebola vaccine trial*. http://www.who.int/features/2015/guinea-ebola-vaccine/en (accessed December 21, 2016).

WHO. 2015c. *Report of the SAGE Working Group on Ebola Vaccines and Vaccination with provisional recommendations for vaccination*. Geneva, Switzerland: Section B: Vaccines and vaccination, WHO. http://www.who.int/immunization/sage/meetings/2015/october/2_WHO_SAGE_WG_ebola_vaccines_and_immunization_MPP_VM_AMHR.pdf (accessed January 17, 2017).

WHO. 2016a. *Final trial results confirm Ebola vaccine provides high protection against disease*. http://www.who.int/mediacentre/news/releases/2016/ebola-vaccine-results/en (accessed January 25, 2017).

WHO. 2016b. *Graph of new confirmed cases per epi week for Guinea*. http://apps.who.int/gho/data/node.ebola-sitrep.ebola-country-GIN-20160511?lang=en (accessed January 17, 2017).

WHO. 2016c. *Graph of new confirmed cases per epi week for Liberia*. http://apps.who.int/gho/data/view.ebola-sitrep.ebola-country-LBR-20160511-graph?lang=en (accessed January 17, 2017).

Copyright © National Academy of Sciences. All rights reserved.

WHO. 2016d. *Graph of new confirmed cases per epi week for Sierra Leone*. http://apps.who.int/gho/data/view.ebola-sitrep.ebola-country-SLE-20160511-graph?lang=en (accessed January 17, 2017).

Widdowson, M. A., S. J. Schrag, R. J. Carter, W. Carr, J. Legardy-Williams, L. Gibson, D. R. Lisk, M. I. Jalloh, D. A. Bash-Taqi, S. A. S. Kargbo, A. Idriss, G. F. Deen, J. B. W. Russell, W. McDonald, A. P. Albert, M. Basket, A. Callis, V. M. Carter, K. R. C. C. Ogunsanya, J. Gee, R. Pinner, B. E. Mahon, S. T. Goldstein, J. F. Seward, M. Samai, and a. A. Schuchat. 2016. Implementing an Ebola vaccine study—Sierra Leone. *Morbidity and Mortality Weekly Review* 65(Suppl 3):98–106.

Copyright © National Academy of Sciences. All rights reserved.

5

Strengthening Capacity for Response and Research

Major shifts in our perceptions of our increasingly connected world have occurred over the last several decades as the global health movement has gained traction and the underlying motivations in the international arena have changed from colonial and paternalistic to a shared vision of good health for all, regardless of where you come from. With this, clinical research has also become an increasingly global endeavor, involving populations that have traditionally been underrepresented in research due to the lack of global interest in the health issues they uniquely face, the lack of commercial viability for the products of research, and the dearth of trained local researchers (Ali et al., 2012; Lang and Siribaddana, 2012). As a result of the globalization of clinical trials and accompanying external investment, developing countries have increased their capacity and resources for conducting research, and increasingly they have also tried to ensure the research agenda is relevant to the health challenges they face (Lang and Siribaddana, 2012).

Diseases like Ebola are not highest on the list of targets for research and development, except when there is a global threat such as the Ebola epidemic of 2014–2015 and the need for clinical research to evaluate therapeutics and vaccines seems more urgent. Building clinical research capacity in smaller, poorer developing countries is not a top priority of the international community; it is a particular challenge in the midst of an outbreak when the focus and attention is on helping patients, containing the outbreak, and preventing pandemic spread. However, strengthening research capacity is vital to preventing, responding to, and ending an epidemic. The World Health Organization (WHO) recognizes that "when assessing a new

Copyright © National Academy of Sciences. All rights reserved.

infectious disease outbreak, it is of utmost importance—but enormously difficult—to quickly estimate its key characteristics, such as clinical severity, clinical presentation, the course of the illness, and the risk factors associated with infection. All such information is critical for decision making" (Williams et al., 2011, p. 63). The knowledge that can be produced through research during an epidemic—and sometimes *only* during an epidemic—is not only critical to informing ongoing preparedness and response, but it can also inform revisions in treatment protocols to advance patient care in real time, identify at-risk groups, and inform clinical trial protocols if there are products in development at a stage ready to be tested in humans (Lurie et al., 2013). This information can only be generated if there is the capacity in place to conduct robust research that meets acceptable scientific and ethics standards. The inherent problems with top-down "parachute research" are well documented and the alternative advocated by many in the field is for international researchers to partner with local scientists (Aizenman, 2016; Heymann et al., 2016).

Although there have been many programs over the years to help build research capacity in low-income countries, it is a difficult and long-term effort. Just a few countries in Africa have developed significant local research capacity capable of functioning on its own and ready to fully partner with international colleagues. In 1990 the Commission on Health Research for Development recognized that health research is an essential part of the health system and that it plays a critical role in improving health outcomes. The Commission Report concluded that "for the most vulnerable people, the benefits of research offer a potential for change that has gone largely untapped" (Commission on Health Research for Development, 1990, p. vii; see also Tugwell et al., 2006). With this report as the springboard, the concept of essential national health research (ENHR) was introduced (Evans, 1990). Instead of identifying specific research issues to address, ENHR is an integrated strategy for organizing and managing health-related research so that the research can contribute to health and development within a country (AfHRF, 2014). The programs that have been launched in the past 25 years cover a range of approaches, from ENHR capacity building by the Geneva-based Council on Health Research for Development (COHRED) to specific disease research in low- and middle-income countries supported by a variety of international institutions, including the Fogarty International Center at the U.S. National Institutes of Health (NIH), the Canadian International Development Research Centre, the UK Medical Research Council, the Wellcome Trust, the Pasteur Institutes, the European Developing Country Clinical Trials Partnership, and the Special Programme for Research and Training in Tropical Disease at the WHO, among many others around the world.

Despite these efforts, the majority of African countries, including

Copyright © National Academy of Sciences. All rights reserved.

Guinea, Liberia, and, Sierra Leone have lagged behind. Most capacity-building programs have focused on the training and career development of individual researchers. As important as that is, it is increasingly recognized that a national health research system must be far more than the number of trained researchers in the country and must also include the institutions and activities involved in the generation and dissemination of knowledge. The health research system is an integral part of the health system and should produce evidence to inform the development and strengthening of national health and public health systems[1] (Alliance for Health Policy and Systems Research, 2004; WHO, 2002).

The Ebola-affected countries are by no means the only countries that lack the necessary infrastructure and resources to respond to an outbreak. As Ariel Pablos-Mendez, the assistant administrator for global health at the U.S. Agency for International Development (USAID), said, "The state of the health workforce and health systems of the affected countries hampers the ability of these countries to respond to the Ebola outbreak—but these countries are hardly alone in having inadequate training, support and numbers of health workers, especially in the rural areas where this outbreak took hold" (Pablos-Mendez, 2014). Forging resilient health systems within all developing countries is critical so that these countries can rapidly respond to emergencies and prevent epidemics from occurring. Fragile health systems increase the vulnerability of countries to the risk of future epidemics, as seen in cholera outbreaks in Haiti in 2010 (Ivers et al., 2013); with influenza H1N1 in 2009, which disproportionately affected populations in Africa and Asia (Viboud and Simonsen, 2012); and most recently Zika (WHO, 2016c). But it is not just developing countries that stand to gain from global investments in health. It is now widely agreed that high- and middle-income countries have a defendable self-interest to invest in capacity-building in low-income countries affected by potentially pandemic diseases such as Ebola—not as a luxury, nor an act of charity, but as a necessity for protecting their own people from disease that, given the globalized economy, will inevitably spread to them. It is important to continue capacity-building investments even when the world's attention turns to the next great threat, as Ebola was swept off the front pages of newspapers and funds were diverted to the threat of Zika (Scott, 2016). The case has been clearly made by the Council on Foreign Relations, "Supporting public health worldwide will enhance U.S. national security, increase prosperity at home and abroad, and promote democracy in developing

[1] Public health systems are commonly defined as "all public, private, and voluntary entities that contribute to the delivery of essential public health services within a jurisdiction." This concept ensures that all entities' contributions to the health and well-being of the community or state are recognized in assessing the provision of public health services (CDC, 2014).

Copyright © National Academy of Sciences. All rights reserved.

countries and those in transition. Emerging risks to the health and security of Americans make it prudent policy to grant higher priority to health in these countries. In addition to the threat of the deliberate spread of disease through biological weapons, Americans may now be at greater risk than at any time in recent history from recognized and emerging infectious diseases. These diseases are resurgent everywhere and spread easily across permeable national borders in a globalizing economy" (Kassalow, 2001, p. 4).

CAPACITY CHALLENGES TO CONDUCTING CLINICAL RESEARCH

The highly regulated nature of clinical trials, as well as the scientific and ethical mandates established to ensure that their risks to participants are minimized and the expected benefits are sufficient to justify going forward, can make trials time consuming and expensive to conduct (DiMasi et al., 2016). In the context of an infectious disease outbreak in a low-resource setting it can be even more difficult to meet the logistical, technical, and regulatory requirements of clinical trials, particularly in the narrow window of time that an outbreak affords. In fact, in outbreaks prior to the 2014–2015 Ebola epidemic not a single clinical trial was set up, as evidenced during recent outbreaks of MERS-CoV and influenza H1N1 which originated in middle-income countries—Saudi Arabia and Mexico, respectively (Gray, 2015). In order to conduct a trial in the setting of an outbreak, in addition to the many traditional clinical trial considerations (e.g., hypothesis testing), numerous issues must be considered and solved; the first step is determining whether and when launching a trial would be feasible. This involves (1) predicting when an outbreak can be expected to be large enough in size and long enough in duration to conduct trials, and (2) condensing the time that it takes to design, obtain the necessary approvals, identify the staff and the site, and implement the trials, so that participants can be enrolled while there are still sufficient numbers of new cases occurring to reach an endpoint for analysis. In an ideal situation, this would involve the preparation of an agile trial design in advance of an outbreak, refining the design according to the local circumstances and context, a rapid global response, and extensive collaboration across multiple organizations in multiple countries. In the Ebola epidemic, however, this preplanning did not happen, research was not on the table for the first 6 months of the outbreak, and trial teams were confronted with multiple challenges which they went to great lengths to overcome. The lack of capacity in the affected countries, along with the delayed recognition by the WHO and other key stakeholders of the extent and urgency of the outbreak, also delayed discussions on the need for research until the declaration of the public health emergency of

Copyright © National Academy of Sciences. All rights reserved.

international concern, and frustrated the ability of researchers and responders alike to adequately plan and respond to the emerging outbreak.

Once the magnitude of the outbreak was appreciated by both national and international stakeholders, shortcomings in local capacity became a roadblock to moving forward with research. For example, inexperience with independent scientific and ethics review of proposed research and limited legal experience in evaluating and negotiating research contracts put the countries at a strategic disadvantage, whether actual or just potential. In order to conduct high-quality research in a timely manner in a resource-poor setting during an emerging epidemic, the following challenges experienced during the Ebola outbreak must be addressed if there is to be a more efficient, rapid, and effective research response:

- poor surveillance and a lack of experience with outbreak investigations in the three countries
- a lack of clinical experience with Ebola-infected patients in West Africa
- a lack of health care personnel and basic and health infrastructure
- a small pool of clinical research experts and research infrastructure in countries
- limited prior experience in the conduct of clinical research
- overwhelmed, understaffed, and poorly supported ethics review boards
- limited experience with contract negotiations and large program project management

Each of these challenges can be addressed by strengthening in-country capacity.

Poor Surveillance and a Lack of Experience with Ebola in Country

The ability to rapidly recognize, coordinate, and respond to outbreaks relies on robust surveillance systems that monitor the incidence of communicable and zoonotic diseases and are able to detect increases and warn of outbreaks. Accurate, timely surveillance data are critical before and during public health emergencies because these data can provide the information needed to identify an outbreak at the earliest possible time as well as for appropriate resource allocation, assessment of the success of response, and planning for staffing and resource needs (McNamara et al., 2016). The increases in international travel and trade, recognition of the emergence and reemergence of communicable disease threats and other health risks, and the need for early and accurate identification of these events was a driving force behind development of the 2005 International Health Regulations

Copyright © National Academy of Sciences. All rights reserved.

(IHR 2005), which requires every country to develop core capacities to "a) detect events involving disease or death above expected levels for the particular time and place in all areas within the territory of the State Party; and b) to report all available essential information immediately to the appropriate level of health-care response; and c) to implement preliminary control measures immediately" (WHO, 2005, p. 40). Subsequently, a 2009 Institute of Medicine (IOM) report called for the development of sustainable surveillance capacity for emerging infectious diseases rather than the buildup of surveillance when a new threat occurs and then its dismantling when the threat disappears (IOM and NRC, 2009). As Dr. David Nabarro, then the senior United Nations system coordinator for avian and human influenza, said at a meeting during the development of the report, "we are dealing with things that are likely to emerge at some time and that need attention. We have to persuade decision makers to invest in surveillance systems and other actions to deal with these uncertainties in a flexible and responsive way without being able to tell them, with an absolute precision, when they are going to emerge and what their economic or social cost might be" (IOM and NRC, 2009, p. 27).

Despite the establishment of effective global public health surveillance being a key stipulation in the IHR 2005, as of 2014 only 64 of the member states had achieved the required core capacities and 48 failed even to respond to the WHO (Gostin and Katz, 2016; Katz and Dowell, 2015). This is of paramount concern because it means that only about one-third of the world's health systems are prepared to respond effectively to a public health emergency. At present, there are no enforceable sanctions available to penalize countries for noncompliance past the deadline, which have already been extended several times, or incentives or support for low-income countries to comply. It is also difficult to see where the financial and other support required will come from, both from the countries themselves and from the international community. This process is slow, steady, and not in the public spotlight; mobilizing the necessary resources is, in fact, very difficult. The 2014–2015 Ebola epidemic revealed several weaknesses in the disease surveillance and response systems in the region.

A Lack of Clinical Experience with Ebola-Infected Patients in West Africa

Despite claims that Ebola was new to West Africa there is some evidence that Ebola was present in the region before the 2014–2015 epidemic. In 1994 the Tai Forest strain of Ebola was identified in an Ebola-infected veterinarian in Cote d'Ivoire who was attending to a colony of chimpanzees affected by a fatal outbreak of Ebola; the veterinarian survived (Formenty et al., 1999). There was speculation that the infection might have been acquired in Liberia where a serological diagnosis of Ebola was made in

Copyright © National Academy of Sciences. All rights reserved.

another individual; however, this was not confirmed by virus isolation (United Nations, 1995). A retrospective serosurvey of 672 serum samples collected at the Lassa Diagnostic Laboratory at Kenema Government Hospital, Sierra Leone, between 2007 and 2014, primarily from Sierra Leone, identified 35 samples (5.2 percent) positive for Ebola virus IgG antibodies. Virus isolation was not part of the investigation; however, there was no recognized outbreak of Ebola during this period and the authors suggested this might be "the result of a reservoir maintaining Ebola in the environment" (O'Hearn et al., 2016, p. 5). Without prior appreciation that Ebola virus was present in the region, the appearance of Ebola in the index case in Guinea in late December 2013 and its subsequent spread in early 2014 was a surprise. This first cluster of cases and the missed opportunity to realize that an Ebola outbreak had begun was dubbed a Black Swan event by Osterholm et al. (2015). A Black Swan event is defined by three attributes: "First, it is an outlier, as it lies outside the realm of regular expectations, because nothing in the past can convincingly point to its possibility. Second, it carries an extreme 'impact.' Third, in spite of its outlier status, human nature makes us concoct explanations for its occurrence after the fact, making it explainable and predictable" (Osterholm et al., 2015).

Outbreak Surveillance

While the first string of related cases in Guinea following the death of the initial index subject was quickly noticed as unusual and was reported to the Ministry of Health in Guinea, as required by IHR 2005, the initial investigation by the ministry reached the conclusion that the likely cause was cholera. This misdiagnosis, which determined the initial response, represented the first serious impediment to a quick and effective clinical and public health response to contain and control the disease. Although this misstep was later alleged to be due to the lack of prior experience with Ebola in West Africa, improved outbreak investigation capacity, backed up by access to diagnostic laboratory expertise, either in country or through established collaborations, preferably in the region, could have resulted in the identification of the true cause of these deaths soon after they were spotted. However, clinicians in West Africa "had never managed cases. No laboratory had ever diagnosed a patient specimen. No government had ever witnessed the social and economic upheaval that can accompany an outbreak of this disease. Populations could not understand what hit them or why" (WHO, 2015a). Regardless of the impetus, be it the lack of awareness of the presence of Ebola in West Africa or lack of experience with Ebola as a clinical and public health challenge, the conditions for propagating a firestorm outbreak were present, awaiting the first spark and the subsequent failure to identify and extinguish it quickly. The 2014–2015 epidemic in

Copyright © National Academy of Sciences. All rights reserved.

West Africa quickly and tragically provided the health systems and health care workers of Guinea, Liberia, and Sierra Leone with plenty of experience with the devastating nature of the infection.

The slow response and porous borders between the three epicenter countries allowed Ebola patients and contacts to freely move from one to the others during the critical first weeks, spreading and escalating the outbreak as it moved from Guinea to Liberia and Sierra Leone and from villages into more populous city centers (WHO, 2015a). Through the intervention of Médecins Sans Frontières (MSF) the outbreak was successfully identified as Ebola; however, by this point it was already rapidly spreading. For a variety of political and economic reasons which overrode the public health concerns, WHO and the affected countries were not as transparent as they could have been (Associated Press, 2015b; Taddonio, 2015). The WHO, as a member state organization, can be reticent to act vigorously when the affected country resists full reporting (Cheng et al., 2015).

The U.S. Centers for Disease Control and Prevention (CDC), along with many other organizations, eventually managed to conduct effective surveillance in the affected region during the epidemic, but in the process it faced numerous challenges, including

- case data that were underreported or missing altogether,
- a slow adoption of nationwide standardization of case definitions,
- difficulty in linking laboratory results with epidemiological data,
- mistrust and violence toward contact tracers,
- a lack of information technology equipment and staff,
- a lack of digital systems to track and analyze outbreaks,
- a lack of basic computer skills, and
- a lack of isolation facilities and laboratory capacity for diagnosis (McNamara et al., 2016).

While the surveillance efforts on the part of the CDC and partners were critical during the outbreak, it is also essential that the effort be maintained after the epidemic subsides (and before the next one begins). Diagnostic laboratory capacity was brought into the three countries by various international partners during the outbreak, including Belgium, Canada, China, France, Germany, Italy, the Netherlands, Nigeria, South Africa, Spain, the United Kingdom, and the United States (Abayomi et al., 2016). However, many of these laboratories were dismantled and removed since the outbreak was halted.

There is reason to be concerned about whether a sustainable surveillance system within countries at a similar level of core capacity as Guinea, Liberia, and Sierra Leone can be established, although there is some appreciation of the need and an attempt to do so by the European Union

Copyright © National Academy of Sciences. All rights reserved.

Integrating Clinical Research into Epidemic Response: The Ebola Experience

Chemical Biological Radiological and Nuclear Risk Mitigation Centres of Excellence Initiative (or EU CBRN CoE), the CDC, Expertise France, and Public Health England (Abayomi et al., 2016; House of Commons International Development Committee, 2016). The CDC acknowledges the importance of continuing to support strong public health and surveillance capacity in the region in order to be prepared to respond to future outbreaks: "With the establishment of CDC offices in Guinea, Liberia, and Sierra Leone, the CDC is well-positioned to continue supporting the expansion of public health and surveillance capacity infrastructure to improve the response to future epidemics" (McNamara et al., 2016). An alternative may be establishing capacity at the regional level. The lessons learned through the program to eradicate polio in Nigeria can serve to inform other developing countries' approach to surveillance and emergency public health challenges (Desmarais, 2016; WHO, 2015d). Notably, during the Ebola outbreak, within days of the arrival of the index case in Lagos in July 2014 the well-established African Center of Excellence for Genomics of Infectious Diseases laboratories at Redeemers University in Ogun State, Nigeria, was able to correctly and safely diagnose the index case for the Nigerian outbreak, using polymease chain reaction at biosafety level 2 containment (Salu et al., 2016). The WHO meanwhile is working to strengthen the surveillance systems of the Ebola-affected countries; efforts include

- providing technical support to the West African Health Organization for the establishment of the West African Regional CDC and its network of national coordinating institutions;
- supporting nine West African countries which will participate in the World Bank's West Africa Regional Diseases Surveillance Systems Enhancement (REDISSE) project, with the preparation of their country profiles;
- supporting the three countries in developing and implementing national surveillance strategies and the National Integrated Disease Surveillance and Response guidelines and tools;
- supporting the Ebola-affected countries with the establishment of national public health institutions (or national CDCs), including study visits to existing national public health institutes in selected countries; and
- assisting the Ebola-affected countries with the development and maintenance of their essential health services situation reports, which monitor the health services recovery progress (WHO, 2017).

Building a viable system for public health surveillance and outbreak response requires training individuals and building the necessary infrastructure along with the sustained support to enable the system to continue to

Copyright © National Academy of Sciences. All rights reserved.

function and grow in capability. Building such a system is step 1 in emergency preparedness.

Conclusion 5-1 *In order to better respond to future outbreaks and recognize an emerging epidemic in time to effectively mount a response, including conduct of clinical trials, it is critical that surveillance, outbreak investigation, and diagnostic capacity be strengthened in low- and middle-income countries. The mandate to ensure compliance with IHR 2005 core capacity for surveillance, reporting, and initial response rests with the WHO; however, two-thirds of countries have not yet reached the minimal required standards, which represents a major gap in global readiness.*

Recommendation 1
Support the development of sustainable health systems and research capacities—Inter-epidemic
To better prepare low-income countries to both respond to future outbreaks and conduct foundational research, during the inter-epidemic period (as covered in 2005 International Health Regulations [IHR 2005]), major research funders and sponsors (e.g., U.S. National Institutes of Health and comparable public and private research funders) and development agencies (e.g., U.S. Agency for International Development and comparable public and private development funders) should collaborate with the World Health Organization and regional centers of excellence to

1. **Assist in monitoring and evaluating the development of national and regional core capacities under IHR 2005, and**
2. **Provide financial and technical assistance to the extent possible or establish a financing mechanism, to help build sustainable core capacities at the intersection of health systems and research (e.g., diagnostics, surveillance, and basic epidemiology).**

Lack of Health Personnel and Basic and Health Infrastructure

An effective emergency response relies on the existing health systems having robust capacity before an outbreak occurs. In the specific example of Ebola, the first link in the chain is surveillance and diagnostic capacity, and, as noted above, in the case of the 2014–2015 outbreak it failed at the outset. The second link in the chain is the capacity of the health care system to care for patients and stop transmission. Prior to the 2014–2015 Ebola epidemic, the three epicenter countries most affected by the Ebola outbreak had weak health systems with chronic shortages of human resources, diagnostic capabilities, infection control experience

Copyright © National Academy of Sciences. All rights reserved.

Integrating Clinical Research into Epidemic Response: The Ebola Experience

and supplies, adequate medicines, and basic infrastructure. The epidemic only further strained available resources, and, in turn, this lack of health system capacity dramatically hindered the Ebola response (Kamal-Yanni, 2015). A 2014 survey of health facilities around the world found that over half did not have protocols to deal with an Ebola suspected patient; two-fifths lacked basic infection protection such as gloves, masks, and gowns; and over one-fifth did not have the basic amenities necessary for facility and personnel hygiene, including something as elementary as running water (Wright et al, 2015). This assessment concluded, "There is general agreement that the Ebola crisis was not quickly contained in Guinea, Liberia, and Sierra Leone because their national health systems were dangerously under-resourced, understaffed, and poorly equipped" (Wright et al., 2015, p. 40).

To compound the problem, the outbreak itself was creating further stress, as physicians, health care workers, and ancillary staff became infected and died. In the early phases of the response, the rate of infection in health care workers was 21 to 32 times greater than in the general population. By May 2015, 0.02 percent of Guinea's population had died due to Ebola, compared with 1.45 percent of the country's doctors, nurses, and midwives (Evans et al., 2015). The differences in overall versus health care worker mortality were equally dramatic in Liberia and Sierra Leone. In the former, 0.11 percent of the general population died, versus 8.07 percent of health care workers, while in Sierra Leone the corresponding figures were 0.06 percent of the general population and 6.85 percent of the health care workers, with nurses and nursing aides accounting for more than half of these losses. Given the relative paucity of physicians and nurses or midwives in the three countries at the onset of the outbreak, these numbers translated into a 10 percent reduction in the number of doctors in Liberia; an 8 percent reduction in nurses and midwives, and a 5 percent and 7 percent reduction, respectively, in Sierra Leone; and 2 percent and 1 percent for doctors and nurses in Guinea (Evans et al., 2015). By May 2015 the total loss of health professionals to Ebola in the three countries was 78, 83, and 79 doctors, nurses, and midwives in, respectively, Guinea, Liberia, and Sierra Leone. While these numbers are not big, the WHO ranked Guinea, Liberia, and Sierra Leone as 26th, 1st, and 4th from the bottom, respectively, among 193 countries in terms of doctors per capita; any loss of trained health professionals would have had a huge impact on the ability to care for patients within the three countries (WHO, 2015c). These tragic deaths were particularly critical early in the outbreak, when the case load was increasing exponentially, isolation facilities were insufficient, the international mobilization of volunteers was still in its early stage, and the personal protective equipment, when available, was cumbersome and still unfamiliar to the health care workers (WHO, 2014). To make

Copyright © National Academy of Sciences. All rights reserved.

matters worse, during the outbreak some health care workers in Guinea, Liberia, and Sierra Leone went on strike over salary and incentive pay for the hazardous work they were being asked to do, further interfering with the care of patients at treatment centers (BBC, 2014; Camara, 2015; Telegraph, 2014).

International agencies and nongovernmental organizations (NGOs) can also contribute to human resource crises in Africa, and elsewhere, when they lure government health workers away into more highly paid positions; they may offer 5 to 20 times more than the comparable public-sector salaries (Pfeiffer et al., 2008). While it is difficult to quantify, the internal brain drain of health care workers from local treatment centers providing routine health care to NGOs during the 2014–2015 Ebola outbreak—or, at a later date, to provide skilled local professional and administrative staff to international research projects—would be expected to contribute to an already weakening health care system performance and to adversely affect the environment in which clinical research could be safely conducted (Anderson and Beresford, 2016). Compounded by the realities of migration of trained nationals to richer countries or to higher salaried international positions, what is known is that by the end of 2014 routine hospitalization and health care services in the three countries were dramatically on the decline, including routine immunization campaigns, leading to subsequent outbreaks of preventable childhood diseases such as measles (Bolkan et al., 2014; Brolin Ribacke et al., 2016; Suk et al., 2016; Takahashi et al., 2015). To prevent the negative impacts of siphoning of health care workers out of the national system and into local and international NGOs in the future, it has been suggested that "rather than hiring workers out of the public system to work in a parallel program, NGOs can integrate projects into local systems and fund additional workers in the public system in accordance with local pay structures. NGOs can also support other incentives to retain staff, such as payment for overtime or after-hours service expansion, or stipends for extra training and additional job responsibilities" (Pfeiffer et al., 2008, p. 2137). In addition to the human resource challenges during the epidemic, trial teams also struggled with basic infrastructure needs such as the provision of power, Internet access, and clean water; a reliable cold chain; backup generators; and more—all of which are issues of equal concern for the operation of an effective health care system in nonemergency conditions (Widdowson et al., 2016). It is clear that achieving this level of basal health care infrastructure is neither simple nor inexpensive, but unless it is prioritized during the inter-epidemic period there is little chance that the response to a future epidemic will be any less fraught than it was in 2014.

Copyright © National Academy of Sciences. All rights reserved.

Conclusion 5-2 *To effectively promote the health of a population, every country requires a well-integrated functional health care and health research system. The separation of the responsibility to care for the sick, which is the humanitarian mandate of medicine, from the responsibility to continually learn and improve the quality of care, or the research mandate, adversely affects the potential to fully meet both imperatives. Mechanisms for training (and the stable support of) key personnel, laboratories, and medical care facilities are essential to establishing an effective clinical research environment.*

Logistical Considerations

Logistics, much like public health measures, are frequently discounted when things are going well. In a humanitarian crisis in a low-resource setting, logistics play a crucial role in successfully mounting a response and conducting research. Researchers and responders must assess the limited resources on hand and determine how to use those most effectively in order to have the greatest impact. In an outbreak scenario of a rare or novel pathogen, this task can be made even more challenging. For example, when the Ebola outbreak began, the limited knowledge about patient management and prevention made it "nearly impossible to prioritize [the] limited available resources for those who might benefit the most, especially early in the response" (Roshania et al., 2016, p. 402). This deficit at the start of the outbreak made the data collection efforts of humanitarian organizations like International Medical Corps (IMC) and MSF (discussed below) critical because this information fed back to develop standardized clinical protocols, identify at-risk groups, and determine other epidemiological factors for contracting Ebola; in addition, it enabled humanitarian and trial teams to define how to best manage their limited resources.

Through their logistical support, humanitarian organizations contributed greatly to the launch of clinical trials during the Ebola outbreak. Trials were launched out of Ebola treatment units (ETUs) established and run by a multitude of international NGOs. For example, PREVAIL II (ZMapp) partnered with IMC at two sites in Sierra Leone; MSF collaborated with trial teams on the Guinea ring vaccination trial, brincidofovir, favipiravir (JIKI), and convalescent plasma trials (Ebola-Tx); and GOAL Global partnered with the RAPIDE-TKM trial team (MSF, 2016; NIAID, 2017; Wellcome Trust, 2015). As these treatment units were already established and running in country, it allowed trial teams to benefit from the existing relationships between the humanitarian organizations running the ETUs and the local officials and community members hosting the ETUs (*Georgetown Journal of International Affairs*, 2014; Levine, 2016). "International Medical Corps field staff worked closely with the NIH team, introducing them to local

Copyright © National Academy of Sciences. All rights reserved.

government and community leadership and helping facilitate numerous town hall presentations of the study, in order to ensure community acceptance before beginning the trial" (Levine, 2016, p. 80). This contributed to the trial team's ability to quickly launch trial discussions and gain local support. In addition, trial teams benefited from the already established ETU infrastructure established by the NGO (storage space, equipment, chlorinated water, etc.). While this is helpful to the research team, it may strain the NGOs and constrain their ability to carry out their missions and provide patient care.

> Any research project carried out in a humanitarian context, however small or non-invasive, will always place a burden on the organization providing the logistical infrastructure for the research study. Even if outside researchers are able to provide for their own staff and the food, housing, transportation, and security of those staff (which will be difficult in many humanitarian contexts), they may still siphon off precious resources from their host organization. These resources include tangibles, such as electricity, water, fuel, and space, as well as intangibles, such as staff time and local political capital. Funding to offset these tangible and intangible overhead costs should be built into any research grant and provided to the humanitarian organization as part of the research partnership. (Levine, 2016, p. 81)

Working with experienced care providers also assisted the trial teams as they were able to learn from the humanitarian medical staff running the ETUs. MSF, for instance, which is widely acknowledged as having expertise in treating Ebola, "took a leadership role in the latest epidemic in ways that it had not before. It taught staff from other organizations—including the WHO and the [CDC]—how to treat people with Ebola" (Hayden, 2015, p. 18). The role of humanitarian organizations in the Ebola outbreak and their crucial contributions to the clinical trials conducted should not be understated. Without their support, it is highly unlikely that any trial would have successfully enrolled patients, or even launched.

For the vaccine trials occurring outside of ETUs and in remote villages, meeting the necessary logistical needs required detailed planning and precision. The fact that the basic infrastructure needed to run trials did not exist in the three countries at the start of the outbreak greatly affected the operational and logistical planning necessary for conducting trials. For example, the Kambia District in northern Sierra Leone is not on the national power grid, which led the EBOVAC team to purchase generators to service their vaccine storage facility, which required 24-hour power. The trial team also had to build or refurbish all of the trial clinics and establish a clinical trials laboratory in Kambia (once the epidemic waned they could no longer make use of the laboratory at the Ebola Treatment Center in

Copyright © National Academy of Sciences. All rights reserved.

Port Loko because it had been decommissioned and the local hospital did not have the capacity to process trial samples). Furthermore, the curfews and lockdowns employed to help control the outbreak resulted in limited working hours and restricted movement, which affected the schedules of the trials; specifically, they "contributed to the unpredictability and delays in an already time-sensitive project" (Watson-Jones, 2016). The emergency context did, however, bring "some operational benefits to the project, including a blanket exemption to import goods for the trial which expired when the state of emergency was lifted" (Watson-Jones, 2016). The Sierra Leone Trial to Introduce a Vaccine against Ebola (STRIVE) team faced similar challenges, and to move as rapidly as possible, the CDC Foundation raised donor funds for immediate needs such as infrastructure building, supplies, and hiring staff.

It should be noted, however, that some of the responses to the logistical challenges contributed to the negative perceptions of the trials and influenced community trust. For example, storing vaccine at the U.S. Embassy in Monrovia, Liberia, initially undermined the credibility of the trial teams. Additionally, in Sierra Leone, WHO responders did not have transportation available to them in order to monitor the spread of the virus despite a recently purchased fleet of vehicles that sat, unavailable, at UN headquarters in Freetown, Sierra Leone, fostering mistrust. "One WHO official suggested Sierra Leonean responders requesting motorbikes for travel to villages buy bicycles instead" (Associated Press, 2015a). When tackling logistical barriers, perception matters and community engagement, consultation, and partnership remain of the utmost importance. (See Chapter 6 for further discussion on community engagement.)

Trial teams in affected countries came up with creative solutions to the innumerable challenges they encountered. Table 5-1 below captures the experience of the team carrying out the CDC's STRIVE study—and the challenges and solutions to implement that vaccine trial. The Guinea ring vaccination trial had the complex task of developing remote trial sites at each ring location. This required the tailoring of standard operating procedures to account for challenges that might arise at the different sites. Checklists had to be developed, prepacked boxes of supplies had to be assembled, and generators and backup generators to supply electricity for electronic record monitoring and cold chains had to be purchased and moved into place—these were just a few of the challenges encountered on top of the processes associated with engaging the community and trial conduct (Capital Reporting Company, 2015).

The committee learned that randomized controlled trials are very possible during an outbreak, but it requires funding, logistical support, and a team that reaches far beyond just the researchers and scientists involved, including communication and social mobilization (discussed in more detail

Copyright © National Academy of Sciences. All rights reserved.

TABLE 5-1 Challenges and Solutions of Implementing Sierra Leone Trial to Introduce a Vaccine Against Ebola

Challenge	Solution
No –80°C (–112° F) freezers or method of transport at –80°C (–112°F)	Purchase and international shipping of freezers; phase-change material transporters
No appropriate space for enrollment and vaccination	Identify, negotiate the use of, and renovate some facilities
No space for data entry and management	Build and renovate facilities
No reliable Internet for data entry, storage, and transmission	Installation of satellite-routed Internet and wireless capacity
No reliable power for cold chain, laboratory, and participant follow-up sites	Installation of generators, solar panels, and backup batteries
Health status of population unknown; poor and dispersed health care access	Establish free medical care; provide supplies to upgrade intensive care unit at referral hospital
Misinformation and misconceptions on vaccines and the motives of the trial organizers	Focus groups, key informant interviews, informational sessions, extensive communication materials
Relevant supplies limited in country	Procure and ship supplies internationally
No basic equipment (e.g., centrifuges) in country for serology study	Procure and ship equipment internationally
No staff GCP training; inexperienced research staff	Conduct large-scale, in-person training; repeated retraining on operating procedures

NOTE: GCP = good clinical practice; STRIVE = Sierra Leone Trial to Introduce a Vaccine against Ebola.
SOURCE: Widdowson et al., 2016.

in Chapter 6). Capacity was brought in and trial teams admirably launched trials in the most challenging of circumstances, but the question now is what will be left in place, who will maintain it, what will be improved for the future, and where are the resources needed for sustainability to come from?

> ***Conclusion 5-3*** *Researchers conducting clinical trials during epidemics in low-resource settings will require substantial logistical support from organizations that build and operate treatment centers (including international humanitarian organizations and national health systems), and these organizations should be included in strategic planning for clinical research activities during the inter-epidemic period.*

Copyright © National Academy of Sciences. All rights reserved.

Integrating Clinical Research into Epidemic Response: The Ebola Experience

Small Pool of Clinical Research Experts in Countries

It is difficult to find systematic assessments of research capacity for the West African region, but the data that are available suggest that the capacity is low. In 2013 the Economic Community of the West African States (ECOWAS) reported on the state of health research in ministries of health among ECOWAS countries as of January 2011 (Sombié et al., 2013). It reported that just half of West African countries had established directorates for health research with defined terms of reference, the existing funding mechanisms were inadequate to support the research structures within and outside the ministries or to improve the capacity of researchers, networking and monitoring activities were weak, and "just 7 percent of the directors of research units were trained in research management" (Sombié et al., 2013). While 86 percent of the countries had broader national health policies in place, and 57 percent had some form of policy or strategic document for research development, half of them had not established national research priorities. Specific country assessments were not included in the report; instead the authors concluded that "urgent action to improve the research environment in the Ministries of Health in the West African sub-region" was essential (Sombié et al., 2013). This report was updated recently by an independent evaluation sponsored by the West African Health Organization (WAHO). Although there was evidence of increased regional investment and some progress, "high staff turnover, weak institutional capacities, and ineffective collaboration" remained significant challenges (Aidam and Sombié, 2016).

In the affected countries, the WHO has assessed the policy frameworks that facilitate the conduct of health research, such as the availability of a national health research policy, a health research strategic plan, a health research program, and health research laws in place (WHO, 2016a). None of the three affected countries met all four criteria, and Liberia satisfied just one, an available health research program which was funded by support from WAHO. The WHO also observed that in the Africa region,

> [o]nly a few countries have successfully coordinated the support and involvement of development partners, the private sector and civil society to improve the research policy environment by developing health research policies, strategic plans, legislation, and programs. Policy-makers and decision-makers are not strongly active in national research agenda priority setting. Only half of the health research institutions surveyed reported having a written policy requiring that researchers obtain the informed consent of research participants. Little or no money is allocated to health research in almost all the countries in region. (WHO, 2016b)

Copyright © National Academy of Sciences. All rights reserved.

Integrating Clinical Research into Epidemic Response: The Ebola Experience

Furthermore, the

> [c]ontinued dependence [of the African countries] on external funds for research may not always align to regional priorities and may not be sustainable. Research institutions in the Region have insufficient facilities and infrastructure: less than half have institutional websites, provide email addresses to research staff, and have a library. There is a serious shortage of qualified staff engaged in health research. Although the majority of researchers are full-time staff, significant numbers also leave their institutions for various reasons, leading to shortages of experienced senior researchers. . . . Researchers have also not always been able to push for their evidence to be used to drive policy. (WHO, 2016b)

As international organizations began to plan clinical research on Ebola, the dire lack of broad experience and knowledge in the affected countries became evident. For example, local researchers had limited or no experience in developing collaborative arrangements with international partners, with obtaining approval from local and international authorities, and with negotiating the legal aspects of clinical trial agreements and other legal documents such as clinical trial agreements, material transfer agreements,[2] data sharing, and post-trial benefits. In addition to these responsibilities, local researchers were also under pressure to identify suitable research study locations, obtain funding, recruit research staff, conduct training on how to work safely in the context of containment, and ensure that research did not impair clinical care.

Collecting Patient Data

The lack of robust health systems and personnel dramatically impeded the ability of clinicians to collect patient-level data, which could be used to inform treatment protocols for patients in real time. MSF and IMC, for example, encountered numerous challenges in collecting patient data in the high-risk zone of their treatment centers. Due to the concern over the possibility of transmitting Ebola via paper records, MSF staff "had to shout the results of ward rounds across the fence to staff in the low risk zone on the other side who recorded the information on clean paper" (MSF, 2016). It was not until MSF started using personal digital assistants that patient

[2] A material transfer agreement (MTA) is a contract that governs the transfer of tangible research materials between two organizations, when the recipient intends to use it for his or her own research purposes. The MTA defines the rights of the provider and the recipient with respect to the materials and any derivatives. Biological materials, such as reagents, cell lines, plasmids, and vectors, are the most frequently transferred materials, but MTAs may also be used for other types of materials, such as chemical compounds and even some types of software (UC Regents, 2017).

Copyright © National Academy of Sciences. All rights reserved.

information could be transmitted in real time, thus reducing the staff time spent recording and relaying information. Similarly, IMC noted that ensuring data quality was a complicated process: (1) using data validation settings in Excel reentry documents, (2) using a codebook to ensure that patient data from various types of patient charts were standardized, (3) conducting additional audits by data entry research assistants, and (4) discussing data entry concerns with the principal investigator (Roshania et al., 2016).

For future outbreaks, IMC stresses that "to facilitate data collection and global reporting in future humanitarian responses, standardized data forms and databases, with clear definitions of clinical and epidemiological variables, should be developed and adopted by the international community" (Roshania et al., 2016). With little empirical evidence on Ebola prevention, treatment, or management to guide clinical care, those responding to the Ebola outbreak in West Africa lacked standardized clinical protocols for patient care contributing to the variability of care across ETUs. The lack of standardized protocols combined with the uncertainty regarding how basic supportive critical care could be translated to the setting of an ETU in a limited resource setting in the midst of an outbreak made the collection of patient-level data and real-time learning imperative.

Humanitarian groups made a concerted effort to collect patient data in ETUs during the 2014–2015 Ebola outbreak and as a result of efforts by IMC, MSF, and others, the global community now has a better grasp on Ebola than ever before. Adam Levine, the primary investigator of the IMC's Ebola research team, was quoted as saying, "At a more fundamental level we have proven that with the right partnerships, the right funding, and the right planning, we can do research in this type of emergency—not just research but high-quality research" (Marshall, 2016). Although the committee did not address the fiscal management systems required to ensure that funds provided through international donors, research institutions, or NGOs are used as intended and are not inappropriately diverted, it is essential to have the necessary systems and audits built into these partnerships. As a team effort this needs to be a shared responsibility between the external and the national members in order to build mutual trust and respect.

Conclusion 5-4 *In an epidemic context, particularly with a highly lethal contagious pathogen in a low-resource setting, recording detailed clinical data is a resource-intensive process that may be seen as diverting attention from patient care. However, despite the difficulties, it is imperative to systematically and comprehensively collect basic information on patient characteristics and clinical outcomes in order to document the natural history of the evolving epidemic and to provide clues to better patient management.*

Copyright © National Academy of Sciences. All rights reserved.

Integrating Clinical Research into Epidemic Response: The Ebola Experience

Recommendation 2a
Develop memoranda of understanding[3] to facilitate data collection and sharing—Inter-epidemic
Research funders, sponsors, national governments, and humanitarian organizations should work together with the World Health Organization to develop memoranda of understanding during the inter-epidemic period to improve capacity to collect and share clinical data, with all necessary provisions to protect the privacy of individuals and anonymize data for epidemiological research.

Recommendation 2b
Provide resources to enable data collection and sharing—Epidemic
At the start of an outbreak, developed countries, research funders, and sponsors should work together with national and international health care providers responding to an outbreak, to provide the additional resources and personnel needed to enable systematic data collection on routine care practices and outcomes. Data collection should begin as soon as possible, and data should be shared and coordinated in a central database to advance an understanding of the natural history of the disease and of the best practices for standard of care. This information should also be used to inform protocols for clinical trials.

Overwhelmed Ethics Review Boards[4]

Among the many technical capabilities required for assessing clinical research proposals is the availability of a trained and independent research ethics committee and the administrative support necessary for its members to work efficiently in the country where the trial will be conducted. The Declaration of Helsinki addresses the role of ethics committees in the

[3] Memoranda of Understanding: Documents whereby parties entering into a partnership agree to an intended common purpose or set of goals. This is sometimes seen as more of a moral agreement rather than a legally binding agreement, and thus it is usually not intended to have the enforceability of a legal document. Although useful as an overarching agreement that sets out the working principles between parties, other written agreements are necessary to create binding commitments.

[4] An ethics review board (ERB)—also known around the world as an independent ethics committee, research ethics committee, research ethics board, or institutional review board—is a type of committee used in research that has been formally designated to approve, monitor, and review biomedical and behavioral research involving humans. The purpose of the review board is to ensure that appropriate steps are taken to protect the rights and welfare of humans participating as subjects in a research study (OHRP, 2017a). The committee is sensitive to the fact that the procedures and guidelines for the protection of human subjects may differ internationally and that some review boards may have more or less capacity for scientific or ethics review, or both (OHRP, 2017b).

Copyright © National Academy of Sciences. All rights reserved.

review of human subjects research: "The research protocol must be submitted for consideration, comment, guidance, and approval to a research ethics committee before the trial begins. This committee must be independent of the researcher, the sponsor, and any other undue influence. It must take into consideration the laws and regulations of the country or countries in which the research is to be performed as well as applicable international norms and standards, but these must not be allowed to reduce or eliminate any of the protections for research participants set forth in this Declaration" (WMA, 2013). In the context of a public health emergency, ethics oversight of research can pose numerous challenges due to the rapid turnaround needed to initiate research (discussed in more detail in Chapter 2).

An earlier mapping study of research ethics committees conducted by COHRED identified more than 165 committees operating in 34 African countries, but concluded that there was great variability in skills, membership, capacity, and efficiency (Kasule et al., 2016). Although there had been efforts to train individuals in low- and middle-income countries in research ethics and help promote the establishment of functional mechanisms for ethics review of clinical research, a subsequent 2016 report concluded that "most African research institutions do not have—or allocate—adequate financial resources to strengthen the capacity of their own research ethics committees. Many [ethics committee] administrators may not have defined roles and responsibilities, may lack adequate training, and do not have efficient electronic information management systems to assist with their heavy and often complex workloads" (Kasule et al., 2016). While Guinean, Liberian, and Sierra Leonean ethics review committees are all included in the National Institute of Allergy and Infectious Diseases–operated ClinRegs database of country-specific clinical research regulatory information, there is no accompanying assessment of their functional capacities (NIH, 2016). However, it should be noted that variability in skills is not unique to African countries and can be seen across low- and middle-income countries and in the developed world as well (Bhatt, 2011).

During the Ebola epidemic, the demands on national scientific and ethics review committees taxed them far beyond what they were capable of handling. For example, scientific competition burdened committees as multiple research teams raced to be the first to scientific and ethics review boards, local principal investigators, and treatment units with their product and protocol (Heymann et al., 2016). There was a high volume of research proposals put forth for ethics review, including clinical trials, anthropological qualitative studies, expanded access, and diagnostic studies. In Guinea, for example, the number of research proposals that the ethics committee considered increased threefold from 2014 to 2015 (Djénab, 2016). Moreover, the proposed research was complex, involving contextual consider-

Copyright © National Academy of Sciences. All rights reserved.

Integrating Clinical Research into Epidemic Response: The Ebola Experience

ations such as "a highly vulnerable population faced with a deadly disease; research activities spanned over three low-income countries, with fragile health systems, poor infrastructure and little experience of medical research (and in particular for clinical trials); and some research was carried out in collaboration with academic institutions, which required setting up new collaborative research agreements very quickly" (Schopper et al., 2016). The committee heard testimony that some researchers did not go through the scientific and ethics review process because it was perceived as too time consuming.[5] If true, this is deeply disturbing and such flagrant violations should not happen in the future. However, despite the numerous challenges, with assistance the in-country ethics committees often fulfilled their responsibilities, and at times could act with remarkable speed. For example, the PREVAIL II trial protocol was submitted in Sierra Leone on March 4, 2015; rewritten for a more resource-limited setting on March 18; and approved by the Pharmacy Board, the relevant body in the country, on April 2 (Davey et al., 2016). This was the result of a close collaboration between the sponsor and national researchers on prior research protocols during the outbreak and of an understanding of the local context and requirements; it also demonstrates the impact of effective collaboration and accrued experience.

In addition to these challenges, most proposals required the review not just of one board, but of multiple scientific and ethics committees in the countries where the studies were slated to take place, at the WHO, and in the country of the trial sponsors (Saxena and Gomes, 2016). Carrying out multiple reviews took time and posed a barrier to trial implementation. While multiple reviews can strengthen trial protocols, without coordination and simultaneous reviews this can contribute to major delays in trial approval and implementation—an unfortunate loss of time in a situation where time is of the essence. The WHO tried unsuccessfully to consolidate ethics reviews, and it has been suggested that a supranational ethics committee be formed, although this would require additional resources to establish and operate (Saxena and Gomes, 2016). MSF has suggested alternative methods to encourage coordinated reviews, including

- establishing ethics committee communication mechanisms well before the emergence of the next outbreak; and
- setting up joint pre-review or review mechanisms that could become feasible via upfront planning and a better use of communication technologies for audio- and videoconferences, which have been seriously underused (Schopper et al., 2016).

[5] Testimony of several participants at the Public Workshop of the Committee on Clinical Trials During the 2014–2015 Ebola Outbreak. Monrovia, Liberia, August 15–16, 2016.

Copyright © National Academy of Sciences. All rights reserved.

If a "pre-review meeting" was organized at the start of an outbreak, representatives of the several scientific and ethics committees likely to review the clinical trials protocols could come together—using videoconferencing capacities as necessary to bring the international participants into the discussion—to consider general issues and approaches to foreseeable ethical dilemmas.

As is well documented in the literature, there is a need to boost scientific and ethics review capacity and develop not just the normal capacity required in inter-epidemic periods but also a surge capacity for use during epidemics (Eckstein, 2004). Part of this is to upgrade the administration of ethics committees and their ability to review and track proposals during the ethics and scientific review process, which has the capacity to handle multicenter and multicountry reviews as well. One such system has been developed by COHRED, and it is now being introduced into a number of countries in Africa in general and in West Africa in particular (COHRED, 2012) (see Box 5-1). If this were adopted as the standard tool in a country or in a region with multiple scientific and ethics committees it would greatly facilitate the necessary coordination. During Ebola, efforts were made by trial teams to enhance the capacity for ethics review by national ethics committees. For example, the committee heard at its meeting in Monrovia, Liberia, that NIH worked with Liberians to help them establish a national research ethics board; initially there were two distinct ethics committees, and it was unclear which would have oversight of PREVAIL. The NIH team met with ethics committee leaders and other national thought leaders to establish a board chartered through the Ministry of Health with

BOX 5-1
Council on Health Research for Development (COHRED)

COHRED, an international nongovernmental organization, concentrates on research and innovation system support to low- and middle-income countries and on the identification of bottlenecks to health research, and then works to design solutions. One important product is Research for Health and Innovation Organiser (RHInnO) Ethics, a cloud-based research ethics committee administration and administrative support platform that is now operating in eight countries in Africa involving 29 research ethics committees (COHRED, 2012). "It has demonstrated that it can substantially reduce review time in multi-centre trials, and improve quality of review—to make sure that life-saving interventions get to those who need it sooner. The West African Health Organization is presently working with COHRED to implement RHInnO Ethics in the national research ethics committees in 5 countries in West Africa in 2017, including Sierra Leone, Guinea, and Liberia. Nigeria already has 16 installations in place" (COHRED, 2016).

Copyright © National Academy of Sciences. All rights reserved.

oversight over all proposals to streamline the process and, at the same time, improve the expertise of the members. Additionally, NIH provided basic human research ethics training to national ethics committee members in order to further expand their ethics knowledge (Kennedy et al., 2006). NIH, through the Fogarty International Center, has now been investing in ethics training for developing country professionals for more than 16 years (Millum et al., 2013). With the need for harmonizing multiple international ethics reviews, it is also critical to have host and sponsor country scientific and ethics review committees and regulatory agencies partner and share information to aid the deliberation of each. These efforts were useful and effective in moving proposals through the approval process during the Ebola epidemic. However, there are limits to how much surge capacity is possible particularly if it is above the local capacity and in-country expertise. It seems apparent that advanced planning for outside assistance when a surge is required, while keeping control over decisions within the affected countries, would be an important step to take. The international community and national institutions would benefit from facilitating strong partnerships—such as, for example, NIH's partnership with Liberia—that make global resources available to local review committees without supplanting the local committee. Deference to the local committee is important because international scientific and ethics committees from high-income countries coming in to support local committees may not fully understand the culture and context in a developing country in which clinical research is being proposed, and culture and context matter.

In addition to scientific and ethics committees, international regulatory agencies must also defer to local regulators and their knowledge of their own populations in order to identify and agree on common principles for regulatory approvals. During the 2014–2105 Ebola epidemic, U.S. and African regulators established numerous agreements "to help facilitate communications between the two agencies on medical products used, or proposed to be used, for Ebola-related purposes as part of cooperative regulatory activities" (FDA, 2016). These partnerships included the U.S. Food and Drug Administration, the Ministry of Public Health and Hygiene of Guinea, the Liberian Medicines and Health Products Regulatory Authority, the Pharmacy Board of Sierra Leone, and the World Health Organization Department of Essential Medicines and Health Products (FDA, 2016). Similarly, the European Medicines Agency

> established a type of rolling review to allow experts to continuously assess the data on new medicines as they became available. Through this process, the Agency was able to develop increasingly robust scientific opinions based on additional data provided during the assessment process. The initial review and subsequent updates were shared with health care decision makers in concerned countries. This enabled them to take informed

Copyright © National Academy of Sciences. All rights reserved.

> decisions on whether and how they wanted to use vaccines and medicines during the latest outbreak, taking into account their specific situation. (European Medicines Agency, 2017)

Preexisting mechanisms for regulatory collaboration were also engaged during the epidemic. For example,

> To address the challenge of authorizing clinical trials of Ebola candidate vaccines with limited available data, the WHO African Vaccine Regulatory Forum (AVAREF) [established in 2006] was used as a collaboration platform enabling regulators, ethics committees and sponsors to reach consensus on key ethical and regulatory questions. Given AVAREF's crucial role in speeding up product development through coordinated regulatory efforts to combat Ebola it is essential that necessary resources are allocated to further strengthen its capacity. (Akanmori et al., 2015)

These types of coordinating activities and collaborative agreements are challenging to establish during an outbreak, so the time to build this capacity is primarily during an inter-epidemic period, when planning, training, and implementation in the countries at risk can be systematically organized and executed in collaboration with the countries and assistance from the international community.

In developing international partnerships to build ethics review and regulatory capacity for clinical trials, it would be advantageous to have experts in clinical research and trial design work together with local research staff and representatives of communities that might be enrolled in these studies. The goal of this collaborative partnership would be to develop model protocols during the inter-epidemic period that meet scientific standards and are acceptable to the local researchers and community representatives. Then, in the event of an outbreak, these model protocols would be available to be rapidly adapted to the specific circumstances of the outbreak and local environment; and trials could begin the implementation phase after the normal review process by the relevant local and international scientific and ethics review committees. An additional benefit that is gained from close partnerships between international ethics and regulatory bodies during the inter-epidemic period is the establishment of a corps of ethics and regulatory experts knowledgeable about different regions or countries, their culture, and the context in which trials would be conducted.

Limited Experience with Contract Negotiations

While the clinical trials conducted during the Ebola outbreak moved at record speed once they were prioritized and through ethics reviews, the actual starts of the trials were unnecessarily slowed by bureaucratic barriers

Copyright © National Academy of Sciences. All rights reserved.

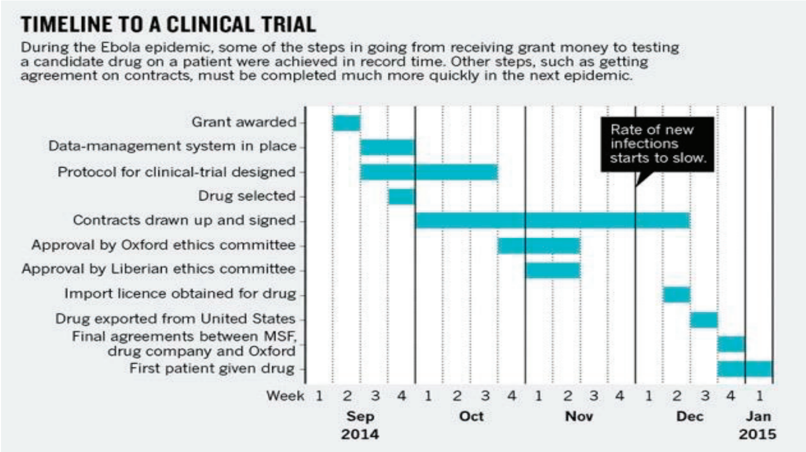

FIGURE 5-1 Timeline to implement a clinical trial.
SOURCE: Lang, 2015.

such as the negotiation and approval process of clinical trial contracts—in other words, they could have been implemented even faster (Lang, 2015). As seen in Figure 5-1, most of the steps required to implement a clinical trial during the Ebola outbreak, such as designing the protocol and obtaining ethics committee approval, could be completed relatively quickly when the partners worked closely together (Lang, 2015). However, developing and signing contracts took up considerable additional time in a process that needed to be very time sensitive, and it seriously delayed the beginning of trials. Speeding up this process would require that countries be "research ready" for the next outbreak and that trial teams, governments, research agencies, relevant NGOs, and the WHO assist countries in acquiring competency in drawing up template contracts (Lang, 2015). Despite the undeniable need for speed in implementation, developing fair research contracts and precluding exploitation is imperative, and trial teams will need to be prepared to overcome the various and complex legal and bureaucratic hurdles in the event of a future outbreak. "The basis for a good collaboration should be trust and openness. A well-negotiated contract will ensure that all partners achieve a fair share of both the benefits and the costs. It is worth spending time on, and will help to ensure minimization of problems in project execution further on" (Edwards et al., 2014).

> ***Conclusion 5-5*** *Helping low- and middle-income countries expand their capacity for the ethics review of human research protocols, regula-*

Copyright © National Academy of Sciences. All rights reserved.

Integrating Clinical Research into Epidemic Response: The Ebola Experience

tory oversight, and the legal review of clinical trial agreements, material transfer agreements, data sharing, and post-trial benefits could reduce bottlenecks in the clinical trial setup process during epidemics and greatly speed up the time to enrollment of the first participant.

Recommendation 3
Facilitate capacity for rapid ethics reviews and legal agreements—Inter-epidemic
Major research sponsors should work with key stakeholders in low- and middle-income countries to

- **Build relationships between local ethics boards and entities that could provide surge capacity for ethics review in the event of an emergency situation. Such efforts would include strengthening networks of ethics boards in a region or connecting local and outside ethics boards, agencies, or experts. Memoranda of understanding setting forth who will provide what services and how decisions will be made should be executed in the inter-epidemic period.**
- **Establish banks of experts in negotiation of clinical trial and material transfer agreements, and other essential components of collaboration, who are willing to offer pro bono advice and support to counterparts in countries affected by outbreaks.**
- **Develop template clinical trial agreements reflecting shared understandings about key issues such as data sharing, post-trial access to interventions, storage and analysis of biospecimens, and investments to build local capacity.**

Those who agree to be available during outbreaks to provide surge capacity would review protocols and provide opinions and advice to local scientific and ethics review committees, which would retain control over and accountability for decisions about protocols. Potential sources of experts in ethics review and negotiation of clinical trial and material transfer agreements are schools of medicine and public health with extensive experience conducting clinical trials in low-resource settings; Public Interest Intellectual Property Advisors, a nongovernmental organization that provides pro bono legal advice to low- and middle-income countries regarding health research and contracts; and COHRED, through its program on fair research contracting (Musolino et al., 2015).

A Clinical Research Document Database

In light of the numerous logistical and operational barriers to implementing clinical trials (discussed above) that confronted Guinea, Liberia, and Sierra Leone it is worth considering what steps might be taken to

Copyright © National Academy of Sciences. All rights reserved.

improve the speed with which research is considered, approved, and implemented. To this end the committee recognizes two capacity-building initiatives that could be useful:

1. The Creation of a Clinical Research Document Database

The database would be meant to provide a framework that can assist national and local researchers in the highly regimented steps required in clinical research. It would be comprised of template documents that are integral parts of the conduct of clinical trials, including trials implemented in low-resource settings—addressing the allocation and provision of scarce resources. The database would include template clinical trial designs for different classes of products (i.e., therapeutics and vaccines); model clinical trial agreements and other contractual arrangements such as material transfer agreements, data sharing, and post-trial expectations; as well as logistical checklists. The numerous logistical tasks that need to be considered and addressed in advance of implementing clinical trials in a low-resource setting and responding to an outbreak are extensive and each task comes with a litany of steps and substeps that must be adapted to the individual situation and followed with precision. These tasks include everything from obtaining sustainable financial assistance, training health care workers and staff, building medical facilities, obtaining reliable power and clean water, to patient monitoring and data collection, cold chain logistics, regulatory document preparation, drug supply accountability, and transportation (road conditions, vehicles, fuel) to name a few. With pre-prepared documents detailing the necessary steps and procedures readily available a country wanting to initiate clinical research would not have to start planning from ground zero and in-country researchers would have a starting point for discussions and planning with international organizations coming in to provide aid. The documents would also be accompanied by explanatory text to help the in-country researcher and Ministry of Health leaders in the affected countries adapt and modify the documents to incorporate pertinent local details, for example, the specific circumstances of the research, the pathogen, and target trial participants.

2. Inter-epidemic Research Partnerships

To be effectively used, the research community and Ministry officials in the countries need to understand firsthand what the documents are and be trained in their use. This can be accomplished best through experience in clinical research. International partners experienced in clinical research can collaborate with low- and middle-income countries to strengthen skills, increase expertise, and prepare in-country experts for rapid, independent

Copyright © National Academy of Sciences. All rights reserved.

Integrating Clinical Research into Epidemic Response: The Ebola Experience

use of the documents in the database when a public health emergency arises. Fogarty, for example, recently launched a program aimed at strengthening research training in Guinea, Liberia, and Sierra Leone, "In the first round of funding, four U.S. institutions received grants to partner with academic centers in two of the West African countries. The support will enable them to design training programs to increase expertise in Ebola, Lassa fever and other emerging viral diseases" (Fogarty International Center, 2016). Initiatives like these, in addition to developing expertise, would help foster trust and partnership between international research stakeholders and would contribute to more rapid implementation of clinical trials.

Developing a readily accessible set of documents for preparedness is not a new idea. A 2012 IOM report detailed a "toolkit" for public engagement in disaster response (IOM, 2012), the U.S. Department of Homeland Security has a Community Preparedness Toolkit to enhance community resilience in an emergency (DHS, 2007), and the WHO regional office in Europe has a toolkit to assess health systems capacity for crisis management (WHO Regional Office for Europe, 2012), while individual institutions have toolkits for clinical researchers interested in doing clinical research in the specific country, on a specific grant, or within a specific institution (National Institute for Health Research, 2017; NIAID, 2015; NIH, 2017; University of Wisconsin, 2017). Having a centralized repository of useful documents to be used on a global scale and linked through inter-epidemic research partnerships between global institutes would be valuable in the event of the next epidemic. The development of this document database paired with training in the application of these pertinent documents would contribute to building national capacity in low- and middle-income countries. The database could help kick-start the research planning phase into motion and focus discussion on the adaptation of generic documents and models to the specific circumstances of the epidemic, what the pathogen is, where it is, who is affected, and which tools should be implemented. Importantly, these templates are only useful if stakeholders know what the tools are, how to use them, and can access an infrastructure to facilitate their application—this is best achieved through training and partnerships with those researchers already well experienced and adept at international trials. The committee has not identified the central repository for this, although it seems to be a WHO function well within their capacity and mandate.

INTEGRATION OF RESEARCH INTO HEALTH SYSTEMS

Building capacity for research cannot—and should not—be separated from building health systems capacity in general; to be most effective clinical research needs to be embedded within the health care system while emphasizing its specialized nature and the need for well-trained practitio-

Copyright © National Academy of Sciences. All rights reserved.

ners. Efforts to improve clinical research in the circumstances of an evolving epidemic setting are interdependent with efforts to improve the overall response, from the initial identification and reporting of emerging infection events, the care of patients and stopping transmission, and the approval and implementation of necessary research, all the way to obtaining regulatory approval for new therapeutics and vaccines—in short, the integration of health care, public health, and health research into a coordinated system with the close collaboration of national and international partners, and full engagement of the community at risk of the disease. Similar conclusions were drawn at a meeting in October 2015, sponsored by the Wellcome Trust, the WHO, the University of Oxford, and the Special Programme for Research and Training in Tropical Diseases (WHO, 2015b). The attendees concluded that research must be included as an "integral and essential component of epidemic preparedness and response," that research should be "integrated with clinical care and public health responses," and that mechanisms "should be established that facilitate efficient and effective joint working" (WHO, 2015b).

The clinical research efforts in the three countries have mobilized resources from the international sponsors to rehabilitate facilities, equip laboratories for clinical and epidemiological research, hire staff, and support salaries, maintain facilities, purchase research supplies and consumables, and upgrade technical and information technology capacity to handle data from collection to storage and on to analysis. Newly refurbished space is often located within deteriorating clinical facilities that remain in dire straits. The difference between the two is obvious, and it is equally obvious that the message it conveys is that research is important but patient care is not. Such observations are not new. In 2015, Daniel Bausch from the Tulane School of Public Health and Tropical Medicine in New Orleans, referring to research projects on Lassa and other hemorrhagic fever viruses in West Africa, noted that externally funded research projects "led to considerable upgrades in the laboratory infrastructure as well as advances in our understanding of Lassa fever, [but] NIH funding restrictions left little room to support patient care. I always felt bad when comparing the shiny new research and diagnostics laboratory at Kenema Government Hospital with the dilapidated, cramped, and poorly resourced Lassa ward only some 50 meters away" (Bausch, 2015, p. 230). Bausch also reflected on ongoing considerations of building a Lassa ward on the part of several international sponsors (European Union, the WHO, and the U.S. Department of Defense); however, frequent logistical issues resulted in lost funding and an unfinished ward in Kenema: "The unfinished shell of the new ward in Kenema still sits collecting rain, a testament to good intentions betrayed by the logistical, bureaucratic, and financial complexities of the world of development, although there are plans now to finally finish it off. . . . How

Copyright © National Academy of Sciences. All rights reserved.

much better and safer might Ebola care have been in Kenema if these projects were seen through to completion?" (Bausch, 2015, p. 230).

To build the health care, public health, and health research systems countries will also need to enhance the quality of the health professional education that they can deliver locally. The committee's visit to the A.M. Dogliotti School of Medicine campus in Monrovia showed how limited the educational resources were for the training of physicians; it is hard to imagine how graduates will be able to deliver competent clinical care for routine illnesses, respond to a crisis, and handle complex situations like the Ebola epidemic of 2014–2015, let alone participate in clinical research, except at the direction of well-trained researchers, primarily from the international community. With increased investments in health research strengthening in the three highly affected countries in West Africa focused on a few facilities and a few people, the divide in the quality and capacity of research facilities versus the quality and capacity of the health care and public health facilities is growing. In parallel, the striking limited capacity of health professional education, at least as observed in Liberia, constrains their ability to educate and train the next generation of health professionals to provide competent health care; these individuals also represent the national talent pool from which future health researchers and leaders will emerge.

Conclusion 5-6 *When conducting research in settings with weak public health, clinical care, and health research infrastructure, efforts to strengthen research capacity without improving the general public health and clinical care infrastructure may inadvertently create the perception that research is more important than care of patients and will ultimately undermine the acceptance of clinical research by the population.*

Recommendation 4
Ensure that capacity-strengthening efforts benefit the local population—Epidemic
When the health care services of a population need to be enhanced or augmented in order to support the conduct of research, development organizations (e.g., USAID), international bodies, and other stakeholders should partner with national governments to ensure that capacity-strengthening efforts are not limited to services that solely benefit study participants.

Because health is now recognized as one of the drivers of economic growth, the concept that improving health contributes to productivity and national wealth generation should be driving country investments to

Copyright © National Academy of Sciences. All rights reserved.

improve the quality of the health care system (WHO, 2001). While there has been a considerable increase in national investment, supplemented by international assistance, the efforts are falling short of the need and often are not well targeted to maximize the impact. As Sir Nigel Crisp observed,

> Even when African health systems' visions are good, they are often poorly embedded within long-term economic growth plans; like most issues of social development, they have to compete with security and economic priorities of leaders who do not realize that their people's health is central to the delivery of economic success. For these reasons, aid funding and the demands attached to it often bypass governments, resulting in aid that focuses on short-term projects rather than an overall resilient system. . . . African government's expenditure on health is mainly spent on recurring costs, such as the training and education of health workers and their salaries, and capital costs such as hospitals, clinics, and transport. Aid funding is mostly focused on the delivery of services, providing life-saving vaccines and basic medicines, and to strengthen accountability processes for the projects they support. . . . The 2008 financial crisis in donor countries has compounded "donor fatigue" among donor countries and their citizens, resulting in the stagnation and reduction of aid to the most vulnerable countries, and increasingly in the combination of aid with trade and military interests which on many occasions prevents it from getting through to the countries that need it the most. (Crisp, 2016, p. 174)

In late 2016 the report of the High-Level Commission on Health Employment and Economic Growth was presented to the Secretary-General of the United Nations. The report argues that there is an urgent need for global investment in the health workforce in low- and lower-middle-income countries to prevent an estimated shortfall of 18 million health workers and maximize the social and economic benefits of improved health, global security, and economic growth (WHO, 2016c,d). Without new commitments from leadership in donor and recipient countries to invest in health systems, facilities, and staff in order to improve health care services and the health status of the population, it is likely that investments in health research systems will not produce the desired sustainable results and that those countries most at risk of emerging infectious disease outbreaks will be no more equipped to deal with the sudden clinical burden and the need to initiate critical research than Guinea, Liberia, and Sierra Leone were in 2014.

Recommendation 5
Enable the incorporation of research into national health systems—Inter-epidemic
National governments should strengthen and incorporate research systems into their emergency preparedness and response systems for

Copyright © National Academy of Sciences. All rights reserved.

epidemic infectious diseases. The multilateral institutions (the World Health Organization and the World Bank Group), regional and international development agencies, and foundations working in global health, should support national efforts by providing expertise and financing.

Financing National Capacity Strengthening

This committee's set of recommendations for actions to strengthen capacity for response and research is intended to provide the basis for cooperative initiatives and a rational partition of primary responsibility among national health authorities, the WHO, and other supranational and international partners involved in health care, public health, and research and development for therapeutics and vaccines, including the academic and private sectors; it is now up to these entities to seize the moment to engage and to invest the critical resources needed to strengthen capacity in low- and middle-income countries for the benefit of all in terms of creating national, regional, and global public goods. There is no doubt that a considerable investment in a sustainable manner will be required and that low-income countries have very limited ability to contribute their own funds to the effort; however, these countries still should be investing partners to claim co-ownership. The committee did not have the mandate or the resources to estimate the amount of funding necessary to get these initiatives off the ground. However, the Commission on a Global Health Risk Framework for the Future has provided an informed benchmark (NAM, 2016). The commission states, "[Their] analysis suggests that expected economic losses from potential pandemics could amount to around $60 billion per year. Implementing [their] recommendations, by contrast, would cost about $4.5 billion per year. This figure has three elements: the cost of upgrading public health systems in low- and middle-income countries, which [their] report puts close to $3.4 billion per year; the cost of enhancing the WHO's pandemic prevention and response capabilities and of financing the WHO and World Bank contingency funds, which [they] assume to be $130 million to $155 million per year; and a proposed incremental investment in research and development of $1 billion per year" (Sands et al., 2016, p. 1284). What seems certain to us is that the actual options are to pay now and prepare in advance, or to pay later when an outbreak occurs, with the likelihood that the cost will be multiple times greater in the latter case.

Many global health initiatives have been financed through global joint funding cooperatives, ranging from the Global Alliance for Vaccines and Immunization on the product procurement side to the International AIDS Vaccine Initiative on the research side, with periodic replenishment of trust accounts to finance a well-thought-out 5-year strategic plan. Generally, these lack meaningful contributions by low-income countries and are sup-

Copyright © National Academy of Sciences. All rights reserved.

ported by just a handful of high- and middle-income countries. However, there are funding models to finance development projects—which is the right way to characterize the effort to build a better, more comprehensive, and more stable health, public health, and health research system—that would engage the usual donors and the usual recipients in a new partnership. One prominent mechanism to accomplish this is overseen by the World Bank Group, which has recently created the Pandemic Emergency Financing Facility (PEF) to rapidly provide funding for the early response to an epidemic, with the goal of moving quickly to gain early and timely control. As the World Bank notes, "While outbreaks are inevitable, pandemics, if addressed early, are for the most part preventable. Money and support delivered at the right time can save lives and economies. . . . Yet as we saw in the recent Ebola crisis in West Africa, there is currently no fast-disbursing financial mechanism to make available significant funds to resource-constrained countries early enough to help them fight an epidemic outbreak that is escalating. Time and again, the world continues to follow the same pattern: money isn't brought to the table until a major outbreak hits an explosive point. Without a strong system in place, the world will simply continue to move from crisis to crisis" (World Bank, 2016a). The PEF is organized to provide funding when a potentially pandemic outbreak occurs that meets the threshold criteria for activation in one of the 77 poorest countries eligible for financing from IDA (the International Development Association component of the World Bank Group), which can also "provide funding to qualified international agencies involved in the response to a major outbreak in affected countries" (World Bank, 2016a). Very briefly, IDA provides grants or loans at zero or near-zero interest rates, with repayment of the principal stretched out over 25–40 years (Mcgroarty et al., 2015). In addition to taking on IDA funding, there is a necessary level of country engagement across sectors; generally the ministry of finance for an IDA-eligible country's government must request the funding and designate its purpose. This means that a health and health research system investment must compete internally for funding with roads, bridges, industrial development, and other national development and investment priorities, requiring the development and articulation of a country strategy to justify the government's choices. What is important is that this represents an internal government-led initiative, in contrast to a donor-specified agenda.

The World Bank Group has other mechanisms of direct relevance to the funding of these initiatives in the form of direct investment projects, including regional (multicountry) investment projects. One such example recently agreed upon is the West Africa REDISSE project. This was developed with financial support from the Bill & Melinda Gates Foundation and technical support from the WHO and the CDC to "address systemic weaknesses within the human and animal health sectors that hinder effective disease

Copyright © National Academy of Sciences. All rights reserved.

surveillance and response" (World Bank, 2016b). Under REDISSE I, Guinea and Sierra Leone have each been approved for $30 million in funding, along with $30 million for Senegal, which the World Bank says

> has shown regional leadership in developing effective disease detection and response capacity. . . . In addition, the West African Health Organization will receive $20 million from IDA and $4 million in trust fund co-financing from the government of Canada to help improve disease surveillance infrastructure, information sharing, and collaboration across the 15 [member] countries that [make up] the Economic Community of West African States (ECOWAS) and across the health, agriculture, and environmental sectors. (World Bank, 2016b)

In addition to making IDA funds available to individual countries that decide to take the opportunity to improve their capacity to respond to another emerging infectious diseases outbreak, the committee strongly supports the development of regional capacity. It is essential to have mechanisms for supporting countries and their governments during inter-epidemic as well as epidemic periods in their efforts to improve the ability of both regional and national systems to respond to epidemic diseases and to provide the necessary infrastructure to support the health research system. The program is expanding, with the announcement of REDISSE II, which will engage Guinea Bissau, Liberia, Nigeria, and Togo, and with plans for REDISSE III to expand to other ECOWAS member states to ensure that by the end of 2017 countries bordering the original nations (Benin, Burkina Faso, Ivory Coast, and Ghana) will be included and will have the means for cross-border collaboration and exchange (PaulClark, 2017). Similar regional investment projects exist in other regions of the world, funded by the World Bank Group or by the regional development banks (African Development Bank, Asian Development Bank, etc.).

Substantial sums of money are involved in the effort to strengthen national health systems, and yet they may not be sufficient to bootstrap the nations of the world to uniformly reach or exceed a minimum standard of capacity under IHR 2005 and to be capable of partnering in clinical research on epidemic infectious diseases. The Ebola epidemic of 2014–2015 demonstrated that a partnership of affected nations and the international community can meet the challenge, but with serious consequences in loss of life, disruption of society, and economic losses. As a result of efforts by the IMC, MSF, and others that provided the initial health care response and also as a result of the subsequent engagement of the larger global community, the epidemic was brought to a close, but not before 11,350 people in 10 countries died. It could have been worse, however, which is why infrastructure in vulnerable countries should be improved—so that, with their participation, insights into the disease and clues to therapeutic and

Copyright © National Academy of Sciences. All rights reserved.

vaccine development gleaned from clinical research can be applied so that countries and international organizations are better prepared to act quickly with better tools the next time there is an outbreak of Ebola.

Whether the recent experience will prove to be a strong enough stimulus for sustained action to elevate the clinical research agenda and national capacity in at-risk countries, as the concern about Ebola no longer raises the specter of pandemic spread, displaced by other crises, remains to be seen. In addition to identifying the funds and partnerships needed, the committee is aware that it has not addressed—nor did it have the requisite expertise to address—the financial management systems, procurement, and logistics required to ensure that the relatively large amounts of funding for clinical research provided through international funders, including the global research institutions, multinational organizations, foundations, and other consortia, are used as intended in efficient and accountable ways. External partners can help by aligning and harmonizing their efforts to strengthen local governance, management, and accountability systems, rather than having donor-specific requirements that further fragment and duplicate efforts to build and use capacity within low- and middle-income countries.

Since most international donors have their own expectations of financial systems to account for grant funds it is important that capacity strengthening is extended to acquiring expertise in these financial systems such that grant or investment funds are managed impeccably, with safeguarding audit processes that allow monies also to be controlled nationally rather than exclusively externally and internationally. It would not hurt if these systems were also harmonized and the burden of effort was on managing the funds properly and not on reporting to different donors using different tools with too little administrative support; it is up to the partner institutions to make this happen (in line with the Paris harmonization agenda[6]).

REFERENCES

Abayomi, A., R. Katz, S. Spence, B. Conton, and S. M. Gevao. 2016. Managing dangerous pathogens: Challenges in the wake of the recent West African Ebola outbreak. *Global Security: Health, Science and Policy* 1(1):51–57.

AfHRF (African Health Research Forum). 2014. *African Health Research Forum Fellowship Program: Module 5: National health research systems*. http://www.ccghr.ca/resources/mentorship-and-leadership/afhrf-leadership-development-learning-modules (accessed January 23, 2017).

[6] The Paris Declaration (2005) is a practical, action-oriented roadmap to improve the quality of aid and its impact on development. It gives a series of specific implementation measures and establishes a monitoring system to assess progress and ensure that donors and recipients hold each other accountable for their commitments (OECD, 2016).

Copyright © National Academy of Sciences. All rights reserved.

Integrating Clinical Research into Epidemic Response: The Ebola Experience

Aidam, J., and I. Sombié. 2016. The West African Health Organization's experience in improving the health research environment in the ECOWAS region. *Health Research Policy and Systems* 14:30.

Aizenman, N. 2016. *Scientists say it's time to end "parachute research."* http://www.npr.org/sections/goatsandsoda/2016/04/02/472686809/scientists-say-its-time-to-end-parachute-research (accessed February 20, 2017).

Akanmori, B., A. Bellah, M. Ward, and L. Rägo. 2015. Regulatory collaboration. *WHO Drug Information* 29(2):127–132.

Ali, R., and A. E. Finlayson, on behalf of the INDOX Cancer Research Network. 2012. Building capacity for clinical research in developing countries: The INDOX cancer research network experience. *Global Health Action* 5(17288).

Alliance for Health Policy and Systems Research. 2004. *Strengthening health systems: The role and promise of policy and systems research*. Geneva, Switzerland: Alliance for Health Policy and Systems Research. http://www.who.int/alliance-hpsr/resources/Strengthening_complet.pdf (accessed January 23, 2017).

Anderson, E.-L., and A. Beresford. 2016. Infectious injustice: The political foundations of the Ebola crisis in Sierra Leone. *Third World Quarterly* 37(3):468–486.

Associated Press. 2015a. *10 critical mistakes in last year's Ebola outbreak*. http://www.businessinsider.com/ap-10-critical-mistakes-in-last-years-ebola-outbreak-2015-9 (accessed February 20, 2017).

Associated Press. 2015b. *Emails: UN health agency resisted declaring Ebola emergency*. http://www.dailymail.co.uk/wires/ap/article-3003593/Emails-UN-health-agency-resisted-declaring-Ebola-emergency.html (accessed February 15, 2017).

Bausch, D. G. 2015. The year that Ebola virus took over West Africa: Missed opportunities for prevention. *American Journal of Tropical Medicine and Hygiene* 92(2):229–232.

BBC (British Broadcasting Corporation). 2014. *Ebola crisis: Sierra Leone health workers strike*. http://www.bbc.com/news/world-africa-30019895 (accessed January 23, 2017).

Bhatt, A. 2011. Quality of clinical trials: A moving target. *Perspectives in Clinical Research* 2(4):124–128.

Bolkan, H. A., D. A. Bash-Taqi, M. Samai, M. Gerdin, and J. von Schreeb. 2014. Ebola and indirect effects on health service function in Sierra Leone. *PLoS Current Outbreaks* 6.

Brolin Ribacke, K. J., D. D. Saulnier, A. Eriksson, and J. von Schreeb. 2016. Effects of the West Africa Ebola virus disease on health-care utilization—A systematic review. *Frontiers in Public Health* 4:222.

Camara, O. 2015. *Guinea labor unions begin general strike over wage demands*. https://www.bloomberg.com/news/articles/2015-01-05/guinea-labor-unions-begin-general-strike-over-wage-demands (accessed January 23, 2017).

Capital Reporting Company. 2015. Transcripts - Clinical Trial Designs for Emerging Infectious Diseases Workshop. Paper read at FDA Clinical Trail Designs for Emerging Infectious Diseases, Bethesda, MD.

CDC (U.S. Centers for Disease Control and Prevention). 2014. *The public health system and the 10 essential public health services*. https://www.cdc.gov/nphpsp/essentialservices.html (accessed February 15, 2017).

Cheng, M., R. Satter, and K. Larson. 2015. *AP investigation: Too few body bags and bad chlorine marred WHO Ebola response in Sierra Leone*. http://www.usnews.com/news/world/articles/2015/09/21/ap-investigation-bungling-by-un-agency-hurt-ebola-response (accessed January 23, 2017).

COHRED (Council on Health Research for Development). 2012. *RHInnO (Research for Health and Innovation Organiser): Your research management process at your fingertips*. Geneva, Switzerland: COHRED. http://www.cohred.org/wp-content/uploads/2012/05/RHInnO.pdf

Copyright © National Academy of Sciences. All rights reserved.

COHRED. 2016. *The WAHO and COHRED collabortation to facilitate reserach for health in West Africa*. http://www.cohred.org/2013/05/the-waho-and-cohred-collaboration-to-facilitate-research-for-health-in-west-africa (accessed January 23, 2017).

Commission on Health Research for Development. 1990. *Health reserach: Essential link to equity in development*. Cambridge, MA: Oxford University Press.

Crisp, N. 2016. *One World Health: An overview of global health*. Boca Raton, FL: CRC Press.

Davey, R., M. Proschan, J. Beigel, and L. Dodd. 2016. *Background and performance of The MCM RCT (PREVAIL* II): A randomized controlled trial of ZMapp™ in acute Ebola virus infection*. Presentation at the Public Workshop of the Committee on Clinical Trials During the 2014–2015 Ebola Outbreak. Washington, DC; June 13–15, 2016.

Desmarais, S. 2016. *Eradicating polio in Nigeria*. http://www.mckinsey.com/industries/health care-systems-and-services/our-insights/eradicating-polio-in-nigeria (accessed February 16, 2017).

DHS (U.S. Department of Homeland Security). 2007. Community Preparedness Toolkit. https://www.ready.gov/community-preparedness-toolkit (accessed February 20, 2017).

DiMasi, J. A., H. G. Grabowski, and R. W. Hansen. 2016. Innovation in the pharmaceutical industry: New estimates of R&D costs. *Journal of Health Economics* 47:20-33.

Djénab, S. N. 2016. *Experiences and lessons learned*. Presentation at the Public Workshop of the Committee on Clinical Trials During the 2014–2015 Ebola Outbreak. Monrovia, Liberia; August 15–16, 2016.

Eckstein, S. 2004. *Efforts to build capacity in research ethics: An overview*. http://www.scidev.net/global/policy-brief/efforts-to-build-capacity-in-research-ethics-an-ov.html (accessed January 27, 2017).

Edwards, D., J. Toohey, and C. IJsselmuiden. 2014. *Negotiating research contracts*. Geneva, Switzerland: Council on Health Research for Development. http://www.cohred.org/wp-content/uploads/2014/06/COHRED-negotiationbookletv-web.pdf (accessed January 23, 2017).

European Medicines Agency. 2017. *Ebola*. http://www.ema.europa.eu/ema/index.jsp?curl=pages/regulation/general/general_content_000624.jsp (accessed February 15, 2017).

Evans, D. K., M. Goldstein, and A. Popova. 2015. Health-care worker mortality and the legacy of the Ebola epidemic. *The Lancet Global Health* 3(8):e439–e440.

Evans, J. R. 1990. Essential national health research. *New England Journal of Medicine* 323(13):913–915.

FDA (U.S. Food and Drug Administration). 2016. *MCM-related cooperative arrangements*. http://www.fda.gov/EmergencyPreparedness/Counterterrorism/MedicalCountermeasures/MCMLegalRegulatoryandPolicyFramework/ucm465378.htm (accessed January 23, 2017).

Fogarty International Center. 2016. *Fogarty provides support to Ebola-affected countries*. https://www.fic.nih.gov/News/GlobalHealthMatters/november-december-2016/Pages/research-capacity-in-countries-affected-by-ebola.aspx (accessed February 20, 2017).

Formenty, P., C. Hatz, B. Le Guenno, A. Stoll, P. Rogenmoser, and A. Widmer. 1999. Human infection due to Ebola virus, subtype Côte d'Ivoire: Clinical and biologic presentation. *Journal of Infectious Diseases* 179(Suppl. 1):S48–S53.

Georgetown Journal of International Affairs. 2014. *NGOs, Ebola, and the future of civil actors in international politics: Five minutes with Sam Worthington*. http://journal.georgetown.edu/ngos-ebola-and-the-future-of-civil-actors-in-international-politics-five-minutes-with-sam-worthington (accessed February 25, 2017).

Gostin, L. O., and R. Katz. 2016. *The International Health Regulations: The governing framework for global health security.* Georgetown Law Faculty Publications and Other Works. http://scholarship.law.georgetown.edu/facpub/1770 (accessed February 15, 2017).

Copyright © National Academy of Sciences. All rights reserved.

Gray, K. A. 2015. *How to conduct a clinical trial during a disease outbreak*. https://blog.wellcome.ac.uk/2015/08/11/how-to-conduct-a-clinical-trial-during-a-disease-outbreak (accessed January 23, 2017).

Hayden, E. C. 2015. Ebola outbreak thrusts MSF into new roles. *Nature* 522:18–19.

Heymann, D. L., J. Liu, and L. Lillywhite. 2016. Partnerships, not parachutists, for Zika research. *New England Journal of Medicine* 374(16):1504–1505.

House of Commons International Development Committee. 2016. Ebola: Responses to a public health emergency. Second Report of Session 2015–16. London, UK: House of Commons. https://www.publications.parliament.uk/pa/cm201516/cmselect/cmintdev/338/338.pdf (accessed February 15, 2017).

IOM (Institute of Medicine). 2012. *Crisis standards of care: A systems framework for catastrophic disaster response: Volume 1: Introduction and CSC framework*. Washington, DC: The National Academies Press.

IOM and NRC (National Research Council). 2009. *Sustaining global surveillance and response to emerging zoonotic diseases*. Washington, DC: The National Academies Press.

Ivers, L. C., J. E. Teng, J. Lascher, M. Raymond, J. Weigel, N. Victor, J. G. Jerome, I. J. Hilaire, C. P. Almazor, R. Ternier, J. Cadet, J. Francois, F. D. Guillaume, and P. E. Farmer. 2013. Use of oral cholera vaccine in Haiti: A rural demonstration project. *American Journal of Tropical Medicine and Hygiene* 89(4):617–624.

Kamal-Yanni, M. 2015. *Never again: Building resilient health systems and learning from the Ebola crisis*. Oxfam Briefing Paper No. 203. Oxford, England: Oxfam International. https://www.oxfam.org/sites/www.oxfam.org/files/file_attachments/bp-never-again-resilient-health-systems-ebola-160415-en.pdf (accessed January 23, 2017).

Kassalow, J. S. 2001. *Why health is important to U.S. foreign policy.* Council on Foreign Relations. http://www.cfr.org/world/why-health-important-us-foreign-policy/p8315 (accessed February 15, 2017).

Kasule, M., D. R. Wassenaar, C. IJsselmuiden, and B. Mokgatla. 2016. Silent voices: Current and future roles of African Research Ethics Committee administrators. *IRB: Ethics & Human Research* 38(1):13–19.

Katz, R., and S. F. Dowell. 2015. Revising the International Health Regulations: Call for a 2017 review conference. *The Lancet Global Health* 3(7):e352–e353.

Kennedy, S. B., A. O. Harris, E. Oudemans, L. Young, J. Kollie, E. S. Nelson, R. A. Nisbett, C. Morris, N. Bartee, E. George-Williams, and J. Jones. 2006. Developing capacity to protect human research subjects in a post-conflict, resource-constrained setting: Procedures and prospects. *Journal of Medical Ethics* 32(10):592–595.

Lang, T. 2015. Ebola: Embed research in outbreak response. *Nature* 524(7563):29–31.

Lang, T., and S. Siribaddana. 2012. Clinical trials have gone global: Is this a good thing? *PLoS Medicine* 9(6):e1001228.

Levine, A. C. 2016. Academics are from Mars, humanitarians are from Venus: Finding common ground to improve research during humanitarian emergencies. *Clinical Trials* 13(1):79–82.

Lurie, N., T. Manolio, A. P. Patterson, F. Collins, and T. Frieden 2013. Research as a part of public health emergency response. *New England Journal of Medicine* 368(13):1251–1255.

Marshall, T. 2016. *Public health experts conclude research conducted at heart of West Africa's Ebola outbreak can provide valuable guidance in preparing for future emergencies*. https://internationalmedicalcorps.org/research-conducted-at-heart-of-west-africas-ebola-outbreak-can-provide-valuable-guidance (accessed January 23, 2017).

Mcgroarty, S., C. E. Rossel, and J. M. Mcfadden. 2015. *The World Bank Group A to Z 2016*. Washington, DC: World Bank Group. http://documents.worldbank.org/curated/en/896931468189267370/The-World-Bank-Group-A-to-Z-2016 (accessed February 15, 2017).

Copyright © National Academy of Sciences. All rights reserved.

McNamara, L. A., I. J. Schafer, L. D. Nolen, Y. Gorina, J. T. Redd, T. Lo, E. Ervin, O. Henao, B. A. Dahl, O. Morgan, S. Hersey, and B. Knust. 2016. Ebola surveillance—Guinea, Liberia, and Sierra Leone. *Morbidity and Mortality Weekly Report* 66(Suppl 3):35–43.

Millum, J., C. Grady, G. Keusch, and B. Sina. 2013. Introduction: The Fogarty International Research Ethics Education and Curriculum Development Program in historical context. *Journal of Empirical Research on Human Research Ethics* 8(5):3–16.

MSF (Médecins Sans Frontières). 2016. *MSF supported research on Ebola*. Brussels, Belgium: MSF. https://www.doctorswithoutborders.org/sites/usa/files/msf_ebola_research_en_web.pdf (accessed January 27, 2017).

Musolino, N., J. Lazdins, J. Toohey, and C. IJsselmuiden. 2015. COHRED Fairness Index for international collaborative partnerships. *The Lancet* 385(9975):1293–1294.

NAM (National Academy of Medicine). 2016. *The neglected dimension of global security: A framework to counter infectious disease crises*. Washington, DC: The National Academies Press.

National Institute for Health Research. 2017. *Clinical trials toolkit*. http://www.ct-toolkit.ac.uk (accessed February 20, 2017).

NIAID (National Institute of Allergy and Infectious Diseases). 2015. *Trans NIAID clinical research toolkit*. https://www.niaid.nih.gov/research/trans-niaid-clinical-research-toolkit (accessed February 20, 2017).

NIAID. 2017. *Putative investigational therapeutics in the treatment of patients with known Ebola infection*. https://clinicaltrials.gov/ct2/show/NCT02363322 (accessed January 15, 2017).

NIH (U.S. National Institutes of Health). 2016. *ClinRegs*. https://clinregs.niaid.nih.gov/index.php (accessed January 27, 2017).

NIH. 2017. *Toolkit resource*. https://www.nhlbi.nih.gov/research/funding/research-support/crg/toolkit-resources.htm (accessed February 20, 2017).

OECD (Organisation for Economic Co-operation and Development). 2016. *Paris Declaration and Accra Agenda for Action*. https://www.oecd.org/dac/effectiveness/parisdeclarationandaccraagendaforaction.htm (accessed February 15, 2017).

O'Hearn, A. E., M. A. Voorhees, D. P. Fetterer, N. Wauquier, M. R. Coomber, J. Bangura, J. N. Fair, J.-P. Gonzalez, and R. J. Schoepp. 2016. Serosurveillance of viral pathogens circulating in West Africa. *Virology Journal* 13:163.

OHRP (U.S. Office of Human Research Protections). 2017a. *Institutional review board guidebook*. https://archive.hhs.gov/ohrp/irb/irb_guidebook.htm (accessed February 20, 2017).

OHRP. 2017b. *International Compilation of Human Research Standards 2017 Edition*. https://www.hhs.gov/ohrp/sites/default/files/internationalcomp2017-part2.pdf (accessed February 20, 2017).

Osterholm, M. T., K. A. Moore, and L. O. Gostin. 2015. Public health in the age of Ebola in West Africa. *JAMA Internal Medicine* 175(1):7–8.

Pablos-Mendez, A. 2014. *Combating the Ebola threat*. http://docs.house.gov/meetings/FA/FA16/20140807/102607/HHRG-113-FA16-Wstate-Pablos-MndezA-20140807.pdf (accessed February 15, 2017).

PaulClark, J. 2017. *Appraisal project information document-integrated safeguards data sheet - Regional Disease Surveillance Systems Enhancement (REDISSE) Phase II - P159040*. Washington, DC: World Bank Group. http://documents.worldbank.org/curated/en/765151485797373436/Appraisal-Project-Information-Document-Integrated-Safeguards-Data-Sheet-Regional-Disease-Surveillance-Systems-Enhancement-REDISSE-Phase-II-P159040 (accessed February 15, 2017).

Pfeiffer, J., W. Johnson, M. Fort, A. Shakow, A. Hagopian, S. Gloyd, and K. Gimbel-Sherr. 2008. Strengthening health systems in poor countries: A code of conduct for nongovernmental organizations. *American Journal of Public Health* 98(12):2134–2140.

Copyright © National Academy of Sciences. All rights reserved.

Roshania, R., M. Mallow, N. Dunbar, D. Mansary, P. Shetty, T. Lyon, K. Pham, M. Abad, E. Shedd, A.-M. A. Tran, S. Cundy, and A. C. Levine. 2016. Successful implementation of a multicountry clinical surveillance and data collection system for Ebola virus disease in West Africa: Findings and lessons learned. *Global Health: Science and Practice* 4(3):394–409.

Salu, O. B., A. B. James, B. O. Oke, M. R. Orenolu, R. A. Anyanwu, M. A. Abdullah, C. Happi, J. Idris, I. A. Abdus-Salam, A.-S. Nasidi, F. T. Ogunsola, O. Tomori, and S. A. Omilabu. 2016. Biosafety level-2 laboratory diagnosis of Zaire Ebola virus disease imported from Liberia to Nigeria. *African Journal of Laboratory Medicine* 5(1).

Sands, P., C. Mundaca-Shah, and V. J. Dzau. 2016. The neglected dimension of global security—A framework for countering infectious-disease crises. *New England Journal of Medicine* 374(13):1281–1287.

Saxena, A., and M. Gomes. 2016. Ethical challenges to responding to the Ebola epidemic: The World Health Organization experience. *Clinical Trials* 13(1):96–100.

Schopper, D., R. Ravinetto, L. Schwartz, E. Kamaara, S. Sheel, M. J. Segelid, A. Ahmad, A. Dawson, J. Singh, A. Jesani, and R. Upshur. 2016. Research ethics governance in times of Ebola. *Public Health Ethics* 1–13.

Scott, D. 2016. *Millions in Ebola funding, a casualty of Zika virus, may not be replenished.* https://www.statnews.com/2016/06/01/ebola-zika-virus-funding (accessed February 16, 2017).

Sombié, I., J. Aidam, B. Konaté, T. D. Somé, and S. S. Kambou. 2013. The state of the research for health environment in the ministries of health of the Economic Community of the West African States (ECOWAS). *Health Research Policy and Systems* 11(1):35.

Suk, J. E., A. P. Jimenez, M. Kourouma, T. Derrough, M. Baldé, P. Honomou, N. Kolie, O. Mamadi, K. Tamba, K. Lamah, A. Loua, T. Mollet, M. Lamah, A. N. Camara, and V. Prikazsky. 2016. Post-Ebola measles outbreak in Lola, Guinea, January–June 2015. *Emerging Infectious Diseases* 22(6):1106–1108.

Taddonio, P. 2015. Outbreak: Inside the troubled early days of Guinea's Ebola response. *PBS Frontline*, May 5. http://www.pbs.org/wgbh/frontline/article/watch-inside-the-troubled-early-days-of-guineas-ebola-response (accessed January 11, 2017).

Takahashi, S., C. J. E. Metcalf, M. J. Ferrari, W. J. Moss, S. A. Truelove, A. J. Tatem, B. T. Grenfell, and J. Lessler. 2015. The growing risk from measles and other childhood infections in the wake of Ebola. *Science* 347(6227):1240–1242.

Telegraph. 2014. *Liberia health workers strike over Ebola "danger money."* http://www.telegraph.co.uk/news/worldnews/ebola/11159217/Liberia-health-workers-strike-over-Ebola-danger-money.html (accessed January 23, 2017).

Tugwell, P., C. Sitthi-Amorn, J. Hatcher-Roberts, V. Neufeld, P. Makara, F. Munoz, P. Czerny, V. Robinson, Y. Nuyens, and D. Okello. 2006. Health research profile to assess the capacity of low- and middle-income countries for equity-oriented research. *BMC Public Health* 6:151.

UC Regents. 2017. *A quick guide to material transfer agreements at UC Berkeley*. http://www.spo.berkeley.edu/guide/mtaquick.html (accessed February 16, 2017).

United Nations. 1995. *Suspect cases of Ebola in Liberia: Press release H/2888*. http://www.un.org/press/en/1995/19951211.h2888.html (accessed February 15, 2017).

University of Wisconsin. 2017. *Clinical research toolkit*. https://ictr.wisc.edu/clinical-research-toolkit (accessed February 20, 2017).

Viboud, C., and L. Simonsen. 2012. Global mortality of 2009 pandemic influenza A H1N1. *The Lancet Infectious Diseases* 12(9):651–653.

Copyright © National Academy of Sciences. All rights reserved.

Integrating Clinical Research into Epidemic Response: The Ebola Experience

Watson-Jones, D. 2016. *The Ebola outbreak: The challenge of establishing a clinical trial during an epidemic*. http://www.clinicaltrialsarena.com/news/operations/the-ebola-outbreak-the-challenge-of-establishing-a-clinical-trial-during-an-epidemic-4925139 (accessed February 15, 2017).

Wellcome Trust. 2015. *New trial of TKM-Ebola treatment to start in Sierra Leone*. https://wellcome.ac.uk/press-release/new-trial-tkm-ebola-treatment-start-sierra-leone (accessed December 21, 2016).

WHO (World Health Organization). 2001. *Macroeconomics and health: Investing in health for economic development: Report of the Commission on Macroeconomics and Health*. Geneva, Switzerland: WHO. http://www1.worldbank.org/publicsector/pe/PEAMMarch2005/CMHReport.pdf (accessed January 27, 2017).

WHO. 2002. *National health research systems: Report of an international workshop*. Geneva, Switzerland: WHO. http://www.who.int/rpc/summit/documents/en/national_health_research_systems.pdf (accessed February 15, 2017).

WHO. 2005. *International Health Regulations (2005),* third ed. Geneva, Switzerland: WHO Press, World Health Organization.

WHO. 2014. *Barriers to rapid containment of the Ebola outbreak*. http://www.who.int/csr/disease/ebola/overview-august-2014/en (accessed January 23, 2017).

WHO. 2015a. *Factors that contributed to undetected spread of the Ebola virus and impeded rapid containment*. http://www.who.int/csr/disease/ebola/one-year-report/factors/en (accessed January 27, 2017).

WHO. 2015b. *Generating evidence for infectious diseases with epidemic potential*. Meeting report, October 20, 2015, London, UK. Jointly organized by the World Health Organization, the Wellcome Trust, the University of Oxford, and the Special Programme for Research and Training in Tropical Diseases. http://www.who.int/medicines/ebola-treatment/meetings/MeetingReport_GEIDEP.pdf (accessed January 23, 2017).

WHO. 2015c. *Health workforce: Density of physicians (total number per 1,000 population)*. http://gamapserver.who.int/gho/interactive_charts/health_workforce/PhysiciansDensity_Total/atlas.html (accessed January 23, 2017).

WHO. 2015d. *WHO removes Nigeria from polio-endemic list*. http://www.who.int/mediacentre/news/releases/2015/nigeria-polio/en (accessed February 16, 2017).

WHO. 2016a. Atlas of African health statistics 2016: Health situation analysis of the African region. Brazzaville, Congo: Africa Regional Office,WHO. https://www.aho.afro.who.int/sites/default/files/publications/5266/Atlas-2016-en.pdf (accessed January 27, 2017).

WHO. 2016b. *The health of the people: What works. Chapter 6: Improving access to health care*. http://www.who.int/bulletin/africanhealth2014/improving_access_to_health_care/en (accessed January 23, 2017).

WHO. 2016c. *Making health systems resilient to changing needs and threats must be a top priority, says PAHO director*. http://www.paho.org/hq/index.php?option=com_content&view=article&id=12755%3Amaking-health-systems-resilient-changing-needs-threats-top-priority-paho-director&Itemid=135&lang=en (accessed January 23, 2017).

WHO. 2016d. *Working for health and growth: Investing in the health workforce*. Geneva, Switzerland: WHO Press. http://apps.who.int/iris/bitstream/10665/250047/1/9789241511308-eng.pdf?ua=1 (accessed January 27, 2017).

WHO. 2017. *National and regional surveillance systems*. http://www.who.int/csr/disease/ebola/health-systems-recovery/surveillance/en (accessed February 20, 2017).

WHO Regional Office for Europe. 2012. Strengthening health-system emergency preparedness: Toolkit for assessing health-system capacity for crisis management. Copenhagen, Denmark: The Regional Office for Europe of the World Health Organization. http://www.euro.who.int/__data/assets/pdf_file/0008/157886/e96187.pdf?ua=1 (accessed February 20, 2017).

Copyright © National Academy of Sciences. All rights reserved.

Integrating Clinical Research into Epidemic Response: The Ebola Experience

Widdowson, M., S. Schrag, R. Carter, W. Carr, J. Legardy-Williams, L. Gibson, D. R. Lisk, M. I. Jalloh, D. A. Bash-Taqi, S. A. Sheku Kargbo, A. Idriss, G. F. Deen, J. B.W. Russell, W. McDonald, A. P. Albert, M. Basket, A. Callis, V. M. Carter, K. R. Clifton Ogunsanya, J. Gee, R. Pinner, B. E Mahon, S. T. Goldstein, J. F. Seward, M. Samai, and A. Schuchat. 2016. Implementing an Ebola vaccine study—Sierra Leone. *Morbidity and Mortality Weekly Report* 65(Suppl 3):98–106.

Williams, S., J. Fitzner, A. Merianos, and A. Mounts. 2011. The challenges of global case reporting during pandemic A(H1N1) 2009. *Bulletin of the World Health Organization* 92:60–67. Geneva, Switzerland: WHO Press.

WMA (World Medical Association). 2013. World Medical Association Declaration of Helsinki: Ethical principles for medical research involving human subjects. *JAMA* 310(20):2191–2194.

World Bank. 2016a. *Pandemic emergency facility: Frequently asked questions*. http://www.worldbank.org/en/topic/pandemics/brief/pandemic-emergency-facility-frequently-asked-questions (accessed February 15, 2107).

World Bank. 2016b. *World Bank contributes to improved disease surveillance and health systems in West Africa following Ebola epidemic*. http://www.worldbank.org/en/news/press-release/2016/06/29/world-bank-contributes-to-improved-disease-surveillance-and-health-systems-in-west-africa-following-ebola-epidemic (accessed February 15, 2017).

Wright, S., L. Hanna, and M. Mailfert. 2015. *A wake-up call: Lessons from Ebola for the world's health systems*. London, UK: Save the Children. https://www.savethechildren.net/sites/default/files/libraries/WAKE%20UP%20CALL%20REPORT%20PDF.pdf (accessed January 23, 2017).

Copyright © National Academy of Sciences. All rights reserved.

Copyright © National Academy of Sciences. All rights reserved.

6

Engaging Communities in Research and Response

Clinical research entails a special relationship between researchers and the communities of the research participants. The research participants volunteer themselves to consent to and take part in the research. Participants at the time of consent can not only be ill, but often fearful, hopeful, vulnerable, expectant, reluctant, and at times confused about goals, benefits, and risks—and sometimes several or all of these simultaneously. Response to an emergency of any nature, be it health or a natural disaster, is difficult, demanding, dangerous, and often unravels under conditions that preclude effective dialogue between those in need and those providing help. Conducting clinical research in the midst of a public health emergency like the Ebola epidemic in 2014–2015 involved most, if not all, of these issues and concerns. This is the fundamental reason why engaging affected communities in all facets of epidemic response is critical to ensuring that the response to the emergency is successful—for example, that community members not only receive and understand public health messages, but that they seek out and trust clinical care, and become engaged to help shape the epidemic response and actively contribute to the efforts to change behaviors in order to protect people from exposure and facilitate getting those infected into care, as well as to become active participants in research. To create the special relationship required for clinical research to go forward, there must be trust and respect built between the researchers, the community, and the individual participants (Ahmed and Palermo, 2010; Nyika et al., 2010).

Community engagement is as much an essential component of the clinical research process when research is being conducted in developed

Copyright © National Academy of Sciences. All rights reserved.

countries—especially among marginalized populations—as it is in developing country settings, but research in the latter particularly when working with marginalized populations may be complicated by different social and cultural perceptions; differences in language and meaning; and asymmetry in authority, expertise, and resources between the researchers and the local participants (Berndtson et al., 2007; CIOMS, 2016; McMillan and Conlon, 2004; NBAC, 2001; Wellcome Trust, 2011). In West Africa there is a vivid history of exploitation by government and international actors, from the slave trade and colonialism of the past to more modern-day civil wars, and disputes over trade policy and resource extraction. There is also a history of unethical and paternalistic medical research on the population by international researchers. Community suspicions that researchers' actions are hiding malicious intent is supported by a collective memory of a lack of informed consent during past research, and this needs to be recognized and overcome (Okonta, 2014).

Communities need to be engaged from the onset of an outbreak response, have access to accurate information about what is happening to individual members of the community and what is happening to them collectively, and have a way to get reliable answers to questions (Kickbusch and Reddy, 2015). There are a variety of ways to share information with community members, depending on the urgency and gravity of the event, including but not limited to one-on-one interviews, town meetings, focus group discussions, community surveys, dissemination of information via the media, and setting up Community Advisory Boards (Nyika et al., 2010). To truly engage communities, they should also be invited and encouraged to be involved in planning and strategy committees for outbreak response and participate in monitoring and evaluation of the outbreak response and clinical trials.

The range of meaningful community engagement activities includes *informing, consulting, involving, collaborating,* and *partnering* with communities and also contributing to *citizen-led* strategies (DELWP, 2013). Community engagement strategies generally involve structured approaches to sharing information, collaborative problem solving, collective action, participation in decision making and transparent accountability with community leaders and stakeholders, along with formal government authorities (George et al., 2015). These steps are not necessarily familiar to researchers who more likely than not have limited experience in community engagement and lack relevant training in the subject. Nonetheless, in order to effectively engage the community, research teams need to successfully link with community members and leaders; this may require bringing someone onto their team who has the necessary skillset and experience, for example, an anthropologist or other social scientist.

Engaging the community serves a number of purposes for epidemic response and research. These include building the knowledge base and

Copyright © National Academy of Sciences. All rights reserved.

capabilities of the community so they understand what the emergency is, what can be done, and what needs to be learned. Incorporating the priorities and perspectives of the community into epidemic response and research plans builds trust and bolsters community confidence in the clinical researchers, both local and international. This serves as a prelude to successfully recruiting them to become involved in clinical trials (Kickbusch and Reddy, 2015; Laverack and Manoncourt, 2015) (see Box 6-1).

During the Ebola epidemic, a lack of early and sustained community engagement efforts hampered both the response and research efforts (Bedrosian et al., 2016). However, as community engagement improved and communities became more knowledgeable and involved, both of these endeavors benefited. For example, communities came to accept randomized controlled trials, an outcome that had seemed impossible in the beginning (Doe-Anderson et al., 2016). A critical factor in this acceptance was the connection and dialogue between health care and research communities, traditional and religious leaders, civil society organizations, women's groups, survivors, and other trusted members of communities who could effectively communicate within the community and who understood how

BOX 6-1
Purposes of Community Engagement During an Epidemic

- Listen to and respect the opinions of people living in affected communities.
- Provide information about the epidemic and epidemic control, and the purpose and nature of research during the epidemic, to community leaders, those directly affected, and the public at large.
- Learn from communities and individuals about their knowledge, experiences, resources, needs, concerns, fears, trusted sources of information, and care—both for epidemic preparedness and response as well as for research during an epidemic.
- Include community members in committees and other activities to plan, prepare, and implement critical response and research efforts.
- Participate in contact tracing, isolation, transport, dead body management, and psychosocial support for families and patients, including for those participating in clinical trials research.
- Support or lead behavior change to improve health and reduce transmission.
- Identify and organize prevention and response activities.
- Identify and organize communications for epidemic preparation and response and for the research during an epidemic.
- Identify and assess rumors and misinformation to address fear and mistrust, including those related to research.
- Build capabilities in the community to participate in clinical trials research, and more broadly to better prevent, identify, and respond to epidemics.

Copyright © National Academy of Sciences. All rights reserved.

to shape culturally sensitive messages and explain response measures and research activities. The experience in West Africa has clearly demonstrated that in any future outbreaks, disasters, or other public health emergencies in which research needs to be conducted, it will be critical that community engagement is begun early and done well.

ENGAGING COMMUNITIES IN RESPONSE

Empirical studies from cholera, shigellosis, dengue, and other outbreaks demonstrate the centrality that communities play in outbreak response and control (Kickbusch and Reddy, 2015). They reveal that understanding local communities' customs, beliefs, knowledge, and practices is essential to the success of disease prevention and treatment interventions as well as of biomedical approaches (Chang et al., 2011; Connolly et al., 2004; Faruque et al., 1985; Mohle-Boetani et al., 1995). The Ebola outbreak was particularly difficult to initially control because it took place in an environment of preexisting mistrust of external responders (both medical care providers and researchers) as well as national and local political authorities (Mukpo, 2015). Rumors began to spread that Ebola was "deliberately propagated as a way for entrenched interests to pocket money donated for the response" (Dhillon and Kelly, 2015, p. 788). It led some community members who were ill to avoid seeking care at health care facilities or Ebola treatment units (ETUs) and instead to visit traditional healers to treat their illness, in part because some believed Ebola was caused by witchcraft (Bedrosian et al., 2016). One study in Liberia revealed that the majority of the population surveyed were afraid of ETUs; individuals reported a fear that they would not be allowed to see their families if they were admitted to an ETU or that they would die if they sought care at one (a concern often confirmed given the high mortality rates in the ETUs) (Kobayashi et al., 2014). It is not surprising that community members at times "defied recommendations from public health authorities because of fear that those authorities were responsible for spreading Ebola" (Bedrosian et al., 2016). A news analysis article in *The New York Times* reported that "The notion, for example, that health officials are conspiring with Big Pharma to consciously spread—and then cure—Ebola as a profit-making venture might sound like the plot to a cheesy summer thriller, but in fact it touches on a genuine aspect of our health care system, said Mark Fenster, a professor at the University of Florida's Levin College of Law and the author of *Conspiracy Theories: Secrecy and Power in American Culture*" (Feuer, 2014). Others noted that "While health workers are struggling to contain the outbreak, conspiracy theories about the deadly pandemic are proliferating on the internet, with people deeming the virus a creation of the West to annihilate Africans or as the result of bioterrorism activities" (Iaccino, 2014). The trust in

Copyright © National Academy of Sciences. All rights reserved.

the Ebola response was further eroded when patients in ETUs, isolated from family and friends, encountered health care workers in their eerie yellow personal protective equipment, which served to hide every visible clue to their humanity except their eyes, leaving little to no possibility for an empathetic connection (Fast, 2015). A redesign of personal protective equipment, with a clear circumferential head cover so the provider's face is visible and with a place for the individuals name on the front and the back, is long overdue (King, 2014).

Early missteps in community engagement and communication exacerbated these issues of mistrust, rumors, and fear. Despite prior knowledge of the effectiveness of community engagement in outbreak response, national authorities and international responders were slow to involve communities in the planning of public health interventions and in developing and implementing communication and social mobilization strategies during the Ebola outbreak (Laverack and Manoncourt, 2015; Marais et al., 2016) The initial response strategy was reported to be "top-down and driven by epidemiological data and the perceived need to treat Ebola patients" (Laverack and Manoncourt, 2015, p. 2) and the initial control measures did not take into account deep-rooted community traditions and beliefs or basic community needs in the West African setting (Laverack and Manoncourt, 2015). For example, mandatory cremation policies countered deeply held religious beliefs about proper burial of the dead and quarantine requirements led to food shortages and disrupted trade (Abramowitz et al., 2015).

However, over the course of the epidemic, communication and social mobilization improved, and with that the situation on the ground improved. As Laverak and Manoncourt observed, "The lead agencies did learn from their earlier mistakes in the present outbreak and have made a genuine attempt to better engage with communities" (Laverack and Manoncourt, 2015, p. 82). There were many examples of improved engagement. A group of anthropologists working in the region, for example, developed the Ebola Response Anthropology Platform to share knowledge and information about the affected communities and community-led responses in real time (Ebola Response Anthropology Platform, 2017). In Liberia, "Community leaders set up response teams (Ebola task forces) to lead contact tracing, case investigation, and reporting, as well as surveillance. The community-based Ebola task force also instituted quarantine measures and provided food and water for those confined to their homes" (Wilson et al., 2016). When the Ministry of Health in Liberia recognized how successful this community-based approach was it provided formal support to the efforts. An important, perhaps critical, step was consultations with traditional and religious leaders to incorporate faith elements into public health messages and providing examples from religious texts to support them. With these types of collaborations, there was significant improvement of community

Copyright © National Academy of Sciences. All rights reserved.

perceptions of key messages. For example, the committee heard from a local reverend who helped control efforts by assuring his community that the traditional practice of "laying of hands" believed to help cure the sick could be done spiritually at a safe distance and still meet religious requirements.[1] The National Traditional Council in Liberia, with support from the U.S. Centers for Disease Control and Prevention (CDC), engaged in efforts to persuade communities to heed public health messaging (Global Communities, 2015). Provided with bullhorns, buckets, and bleach, the local tribal chiefs and elders went from village to village to demonstrate how people can help to stop the transmission of Ebola (Act Alliance, 2014). Another community-based approach was the creation of Community Care Centers (CCCs) led by Liberian county health officials and nongovernmental organizations (NGOs). CCCs were established so that patients who were awaiting Ebola diagnostic tests or entry into ETUs could be admitted and provided with basic care, but also helped to destigmatize Ebola and to encourage persons with illness to seek care rather than remain at home. In addition, these centers facilitated contact tracing of exposed family members (Logan et al., 2014). These modifications to the response were intended to convey critical information and engender trust from the community and thereby improve the community's participation in the efforts to slow the spread of Ebola. It seems likely that if such initiatives to engage and share information were commenced earlier, and community participation in planning and implementing response and research programs were prioritized, it might have greatly affected the communities' receptivity to clinical trials. The fear and mistrust generated by poor initial community engagement in response activities had a direct impact on the real and perceived feasibility of conducting clinical trials during the epidemic.

Marais et al. have proposed an eight-step process for engaging communities during outbreaks, which could be adapted for future scenarios (Marais et al., 2016). Many of their steps focus on the need to identify and partner with key trusted leaders, both men and women, including village or traditional chiefs, religious leaders, traditional healers, community health workers, and others who have the respect of community members. They provide a critical observation: "Low-resource communities around the world are accustomed to meetings called by outsiders, in which they are informed of a new health threat and the need to comply with directions. Such meetings are often poorly attended and sometimes promote fear or further feelings of disconnect between health authorities and local residents" (Marais et al., 2016, p. 444). The EBOVAC-Salone team discovered this in the process of implementing their vaccine study when they were approached by a small group of stakeholders from the community who had

[1] Presentation of Reverend John Sumo. Public Workshop of the Committee on Clinical Trials During the 2014–2015 Ebola Outbreak, Monrovia, Liberia, August 16, 2016.

Copyright © National Academy of Sciences. All rights reserved.

attended their information meetings but did not feel they could express their concerns in public (Enria et al., 2016). They recommend instead having community leaders organize meetings with a few medical or research team members invited as guests. At these meetings, community members could map assets (e.g., faith-based groups, traditional healers, local radio stations, schools, health centers, youth leaders, and elders) that can help promote public health measures or research activities and identify gaps in community resources and risks. It was essential to recognize that "community members may help facilitate a process of turning problems into assets, [for example] when nursing and medical students in Sierra Leone whose schools were closed went on bicycles to find new Ebola cases" (Marais et al., 2016, p. 444).

> ***Conclusion 6-1*** *At the beginning of an outbreak it is critical that national and international agencies engage key community representatives, religious and traditional leadership, and others working in the community, such as nongovernmental organizations, faith-based organizations, and civil society organizations, to establish communication to foster mutual trust and a partnership for response and research activities.*

ENGAGING COMMUNITIES IN RESEARCH

During the Ebola outbreak some community members believed they were being used as "guinea pigs" for foreign researchers. "A local radio reporter asked whether signing a consent form was tantamount to a 'death warrant' for volunteers. A daily newspaper said simply, 'Liberians are not animals.' Scientists have been left scrambling to win over the trust of the Liberian people on the ground" (Onishi and Fink, 2015). In this environment, the importance of community engagement cannot be overstated. Wilson et al. comment that "When communities are not involved from the beginning, they feel like objects of the research rather than partners in the process, thereby leading to distrust, poor communication, rumors, and misconceptions, all of which negatively impact the process and ultimately the outcome of the research" (Wilson et al., 2016).

As discussed in earlier chapters, research activities were not considered in the early months of the epidemic, and once they were, there was tremendous pressure to launch trials as rapidly as possible. This urgency did not leave adequate time for research teams to engage communities in the initial phase of trial planning and led to disagreements about what communities would or would not accept at the WHO meetings, particularly in relation to trial design. Folayan et al. observed of the plans in place to conduct trials on therapeutics and vaccines that "the timelines are so short that the prospect for effective community engagement is dismally low despite the now strong recognition to effectively engage local communities in the clinical research

Copyright © National Academy of Sciences. All rights reserved.

process" (Folayan et al., 2015, p. 1). Research teams had varying knowledge and experience on community engagement prior to the outbreak, but all quickly understood the importance of consulting with communities on all aspects of the research proposal prior to initiating trials. The below section describes the engagement activities of several of the teams and the lessons they learned in the process.

Community Input on Research

Research teams seeking to investigate Ebola therapeutics or vaccines initially sought approval from the appropriate regulatory authorities and advisory bodies, but in some cases did not obtain local community opinions or input in the research planning phase. According to Nyika et al., one reason that community engagement is so important is that "relying solely on the approvals from local ethics committees and regulatory authorities and high-level technical advisory bodies without any practical efforts to interact directly with ordinary communities may in the long run prove to be unsatisfactory to the communities concerned and other stakeholders" (Nyika et al., 2010, p. 3). This point was illustrated when the Partnership for Research on Ebola Vaccines in Liberia (PREVAIL) trial was launched (NIAID, 2015). The trial was initially requested by Liberia's Minister for Health and Social Welfare in an official letter to the U.S. Secretary of Health and Human Services. The response was rapid, and the U.S. and Liberian government agencies quickly established a working partnership (see Figure 6-1). The resulting research proposal received the required regulatory and ethics approvals in country. But it was subsequently held up due to a challenge by a group of Liberian politicians, lawyers, human rights activists, ethicists, journalists, and academicians who "were opposed to the concept of conducting clinical research with inadequate health care facilities, in a research naïve population, during an ongoing public health crisis" (Doe-Anderson et al., 2016, p. 70). According to Dr. Vuyu Golokai, at the time the Dean of the Dogliotti College of Medicine in Monrovia, the fact that research authorization came from high-ranking political authorities (i.e., the President) inhibited local scientists and community members from voicing dissent (Sendolo, 2016). Stephen B. Kennedy, the coordinator for Ebola research and the Incident Management System in the Liberian Ministry of Health, admitted at a press conference in Monrovia that several actions were missed along the way before the trials began. Kennedy said, "We failed to carry out (comprehensive) consultations. For example, we left out the media, the legislature, women and other important groups in our consultation process during the planning stage. . . . We are not politicians; we are medical people, and so we were not sensitive enough to these procedures. We only took into consideration the medical community during

Copyright © National Academy of Sciences. All rights reserved.

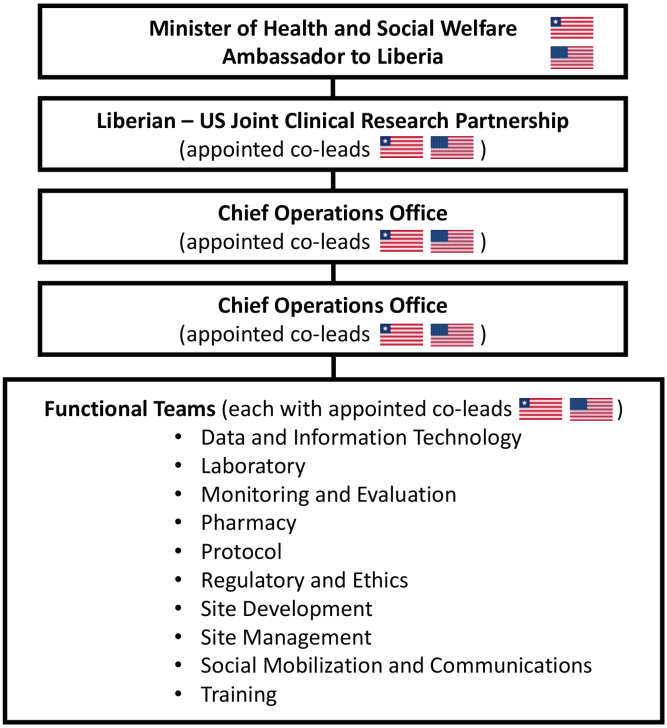

FIGURE 6-1 Organizational structure of the Liberian–U.S. research partnership.
SOURCE: Doe-Anderson et al., 2016.

the initial process. However, we will do all we can to meet those concerns that are being raised" (Yates, 2015). Dr. Clifford Lane, the deputy director for clinical research and special projects and the director of the Division of Clinical Research at the National Institute of Allergy and Infectious Diseases, reflected in the context of clinical research initiatives in Liberia: "This concept of social mobilization, I had not heard that term before. But I came to realize it is one of the most critical things for success in this country" (Onishi and Fink, 2015).

Subsequently, the PREVAIL team held a series of meetings initiated by the Liberian vice president, where concerns were expressed about issues such as informed consent, inadequate testing of the vaccines in humans, the possibility of giving false hope to an at-risk population, and the potential that participants were being exploited or coerced (via compensation) to help develop a lucrative vaccine for the manufacturer. While dissenting voices remained, the study was ultimately approved through hard work and after extensive dialogue in the community to address the many questions

Copyright © National Academy of Sciences. All rights reserved.

and concerns raised, and through crafting simple yet comprehensive messages about informed consent and the risks and benefits of participating. Additional stakeholder meetings resulted in an agreement that research participants would have post-trial access to any vaccines and treatments that were proven effective (Doe-Anderson et al., 2016).

Even with these insights, the PREVAIL trial team reported that it took 3 months of intense engagement with communities to achieve the goal of 1,500 consented participants. This entailed a multipronged approach including meetings with national stakeholders and local leaders within target communities to collect information about community concerns and to better understand cultural, religious, and traditional norms. For example, team members learned that they should use the word "study" instead of "trial," because the latter connoted experimentation on animals in local understanding and thus would be negatively received. The trial also hired participant trackers from the community to assist with follow-up; as Wilson et al. (2016) commented, "the role played by these trackers in promoting behavior change to build lasting trust and sustainable relationships within these communities cannot be overemphasized" (Wilson et al., 2016).

The CDC-supported Sierra Leone Trial to Introduce a Vaccine against Ebola (STRIVE) team also spent significant time and resources engaging with communities (CDC, 2015). Prior to presenting the study to the full government and the media, leaders from the Ministry of Health in Sierra Leone and the College of Medicine and Allied Health Sciences "conducted numerous outreach sessions with traditional and religious leaders of selected chiefdoms, district health leaders, and professional organizations to explain the proposed trial, to understand concerns, and to garner support and feedback. Study team members also met with leaders of every eligible health facility" (CDC, 2016). The STRIVE team reported holding 175 informational sessions in sites where the vaccine trial was held, using materials developed during the initial engagement phase. Participants were presented with consent forms at the information session, but they also had 24-hour access to a hotline with trained staff to answer questions about the trial and procedures (Widdowson et al., 2016).

In the case of the Guinea ring vaccination trial (also known as Ebola ça Suffit), social anthropologists provided advice to the trial team on appropriate communication channels and methods to approach communities. The team employed community facilitators, who spoke the local language, to explain the purpose of the trial and answer questions regarding concerns about potential harm from the vaccine.[2]

[2] Testimony of Ana Maria Henao Restrepo, Medical Officer, Department of Immunization Vaccines and Biologicals, WHO. Public Workshop of the Committee on Clinical Trials During the 2014–2015 Ebola Outbreak, London, UK, March 22–24, 2016.

Copyright © National Academy of Sciences. All rights reserved.

The EBOVAC-Salone trial first trained a community liaison team, comprised of locally recruited staff, on the basics of clinical trials. The initial phase of the community engagement strategy was a 2-month process that included individual consultations with key stakeholders (e.g., elected leaders, traditional leaders), and a series of public meetings hosted with local civil society members and traditional leaders. Additionally, the team conducted "house-to-house sensitization visits" and participated in a local call-in radio show. Finally, the team recruited four local research assistants "to examine community and participant perceptions and experiences of the EBOVAC-Salone vaccine study, including any rumors and concerns about the trial and vaccine" (Enria et al., 2016, p. 4). A subsequent paper published about the EBOVAC-Salone trial underscores the important role of social science and community liaison teams to shape engagement and communication strategies. It also highlighted that community engagement and communication needs—including risk and rumor management—be anticipated, and strategies and funding be included in research plans (Enria et al., 2016).

Community engagement can help facilitate community understanding of key research objectives and concepts and reduce misunderstandings about research. It is clear from the experiences of the researchers involved in clinical trials during the Ebola outbreak that community engagement requires extensive dialogue with key community representatives on complex issues such as study design, the potential benefits and risks of investigational vaccines or therapeutics, and fair distribution of benefits to participants and communities (see Table 6-1). But by giving communities the opportunity to share perspectives and provide input on these issues, researchers can adapt study protocols and clinical trial agreements to address community concerns when possible, and when such adaptations are not technically feasible can continue the conversation to improve understanding.

As detailed earlier in this report, there was much debate among researchers and stakeholders over what would or would not be considered acceptable to the communities that would be participating in the clinical trials, without necessarily understanding the basis for community perceptions. According to a report from the Institut national de la santé et de la recherche médicale (Inserm)[3] and the French Institute for Development Research, national health officials, Médecins Sans Frontières, and national caregivers argued that communities would not understand or accept certain design features, such as randomization (Botbol-Baum et al., 2015). However, the committee heard testimony in Liberia that local community representatives were largely not included in early discussions about trial design. For example, Mandy Kader Konde, a professor at the University

[3] Inserm is the French National Institute of Health and Medical Research.

Copyright © National Academy of Sciences. All rights reserved.

TABLE 6-1 Determining Research Benefits for Study Participants and/or Communities Involved in Research

Consulting the Community to Negotiate Research Benefits	Steps and Considerations
Which community?	Identify the community according to community characteristics.<br>Identify degree of community involvement in research.<br>Study the chosen community with regard to sociocultural structure and political/traditional leadership.
Which community representatives?	Identify legitimate representatives of the community, and do not reinforce existing inequitable structures and relationships, such as gender inequities.
How to negotiate?	Provide information about the research.<br>Assess risks, burdens, and benefits for individual participants, the community, and sponsors.<br>Provide information about previous benefit agreements.<br>Provide support for negotiations.<br>Recognize that benefit negotiations are dynamic.
What comes next?	Make benefit agreements publicly available.

SOURCE: Schulz-Baldes et al., 2007.

of Conakry and chairman of the Department of Public Health as well as chairman of the Guinea Ebola Research Commission and executive director of the Center of Research on Diseases (CEFORPAG), stated that time was lost in high-level discussions around facets of trial design, such as randomization and the use of placebos, without ever discussing these features directly with the community. With effective community engagement and articulation of aspects of trial designs, community buy-in is possible. This has been clearly demonstrated by the community acceptance of and participation in the PREVAIL randomized controlled trial. The PREVAIL team reported successfully describing randomization using flip charts and local terms, after consulting with local people to determine the common parlance for randomization, for example "lucky ticket" or "eeny-meeny-miny-moe," and they reported that through using local colloquial phrasing, participants had an increased understanding of the process.[4]

Respect for communities requires that communities be engaged in a process of dialogue about the need for research, the nature of the uncer-

[4] Testimony of Elizabeth Higgs, global health science advisor, National Institute of Allergy and Infectious Diseases, National Institutes of Health. Public Workshop of the Committee on Clinical Trials During the 2014–2015 Ebola Outbreak, Washington DC, June 13–15, 2016.

Copyright © National Academy of Sciences. All rights reserved.

tainty to be addressed, what is known about the status of the interventions to be used, including benefits and risks, and the merits and limits of possible trial designs. In a context of scarcity, need, and heightened mistrust, as in the midst of the epidemic, these conversations can be challenging—but they are an indispensable component of ethically sound research and are critical to treating study communities as full partners in the effort to find ways to improve the community members' health.

> ***Conclusion 6-2*** *National-level agreements with international researchers for the conduct of clinical trials during an epidemic do not necessarily indicate local acceptance or understanding of the research activities. Affected communities may have legitimate concerns about research that national authorities do not fully recognize.*

> ***Conclusion 6-3*** *Community engagement and social mobilization efforts are essential for public understanding and acceptance of research, and they need to be linked to other aspects of the epidemic response. International response and research teams would be strengthened by the inclusion of social scientists and others with expertise in community engagement. In addition, and ideally, researchers and responders should receive training in (1) cultural competency, (2) rapid appraisal techniques to identify key individuals and groups who influence local opinion, and (3) methods to assess affected populations' understanding of and concerns regarding clinical research and how they can participate in the research.*

Individual and Community Consent

A key component of clinical research is obtaining the informed consent of participants. While U.S. requirements for informed consent focus almost entirely on individual autonomy and individual consent, some cultures consider the community's perspective to be a "fundamental aspect of individual decisions" (Diallo et al., 2005, p. 255). International research guidelines have increasingly reflected this cultural difference, noting that researchers should respect local customs such as "obtaining permission from a community leader, a council of elders, or another designated authority" (Diallo et al., 2005, p. 255). However, it should also be clear that while community consent may be an important or necessary first step to obtaining individual consent, it cannot replace individual consent. The U.S. Food and Drug Administration guidance states, "Community consent is not a substitute for individual informed consent required under the IND/IDE regulations, nor can the community consent on behalf of individual members to permit their participation in a study" (HHS, 2016).

Copyright © National Academy of Sciences. All rights reserved.

The committee considered the following questions on consent pertinent to its charge: first, what if any type of community consent was obtained; second, were the individual consent processes designed to enable the population to understand the nature of the research; third, to what extent was obtaining meaningful informed consent difficult because of the conditions under which the process was carried out; and fourth, in future, similar epidemics, is it appropriate to alter or waive any consent requirements? Ideally, researchers would receive consent from both communities and individuals, in whatever manner is appropriate for the specific cultural traditions and understanding of the community. For example, a malaria vaccine trial in Mali sought the consent of the community in addition to individual consent before initiating research (Diallo et al., 2005). They held introductory meetings with health authorities and government officials, followed by formal meetings with neighborhood and religious leaders and traditional practitioners. The researchers visited community leaders in their homes to further explain the study and to answer any questions, and these leaders in turn transmitted information to the general population. In keeping with community traditions about formal agreements, researchers documented the community consultation and consent process through meeting minutes, which were signed by top community leaders. The researchers found that obtaining community consent through this process had a number of practical and ethical benefits: it ensured widespread knowledge about the research project, avoided potential resistance from local leaders, facilitated referrals of patients through traditional practitioners, and reassured community members that their leaders were comfortable with the project. With the consent of the community, obtaining individual consent became easier. During the Ebola epidemic, it was unclear to the extent in which community consent was obtained, though as detailed above, trial teams did hold group sessions and meetings to address community concerns prior to enrolling participants. For example, the PREVAIL trial team held group information sessions in which they discussed the plans for the study. Following those sessions, those who were interested in participating went through an individual consent process, and, as in the STRIVE trial, they were provided with 24-hour access to a hotline with trained staff to answer questions about the trial and procedures (Widdowson et al., 2016).

Participant comprehension is critical to the informed consent process, which means that while written documentation of consent is required, it is not enough to merely obtain a signature. The researcher must ensure that participants are adequately informed, have voluntarily agreed to participate, and understand that their consent may be withdrawn at any time without affecting their access to care (HHS, 2016). There are well-recognized issues with obtaining informed consent that may particularly occur with complex research designs and low-literate populations. Dur-

Copyright © National Academy of Sciences. All rights reserved.

ing the Ebola epidemic these issues were especially pronounced given the distinctive (though not necessarily unique) circumstances detailed above—the pervasive sense of urgency; fear and distrust of authority and foreign researchers; little prior experience with clinical trials; critically ill patients that were occasionally too sick or delirious to give consent; and lack of time for caregivers to spend with patients. Long multipage forms using technical language and long lists of potential adverse effects, which are often used for consent for clinical research in high-income countries, are difficult to navigate, even by literate, educated trial participants.[5] The Nuffield Council addresses these points, noting that "consent forms often appeared to be designed to protect researchers and their sponsors rather than participants. The forms [are] frequently too long and complex, making them inaccessible to participants" (Nuffield Council on Bioethics, 2002, p. 15). During her discussions with the committee, Luciana Borio, acting chief scientist at the U.S. Food and Drug Administration, welcomed the suggestion of shortened consent forms that use clear and simple language, reducing the unnecessary jargon and lists that are so often used to cover liability concerns and satisfy legalistic institutional review board (IRB) imposed requirements in affluent countries.[6] The consent forms from the trials the committee reviewed were generally three to four pages in length.[7]

The consent process needs to be conducted in the language of the participants. However, the translation of forms into local languages may add to the confusion when concepts are not clearly stated and mistranslations result in a different meaning than intended (Chaisson et al., 2011; Samandari et al., 2011). Back translation to ensure that the original meaning has been maintained is therefore beneficial (Jhanwar and Bishnoi, 2010). In low-literacy settings, consent form language may be accompanied by the use of pictorial aids to convey the complex processes that are described. For example, in one study, a multimedia informed consent tool demonstrated improved comprehension and retention among low-literate study participants (Afolabi et al., 2015). The EBOVAC-Salone trial developed a flipchart to facilitate discussion between researchers and potential participants which included a number of pictures that illustrated the points in the consent

[5] Testimony of Ana Maria Henao Restrepo, Medical Officer, Department of Immunization Vaccines and Biologicals, WHO. Public Workshop of the Committee on Clinical Trials During the 2014–2015 Ebola Outbreak, London, UK, March 22–24, 2016.

[6] Testimony of Luciana Borio, acting chief scientist, U.S. Food and Drug Administration. Public Workshop of the Committee on Clinical Trials During the 2014–2015 Ebola Outbreak, Washington, DC, February 22–23, 2016.

[7] Consent forms were obtained through personal communication with several trial teams: Peter Horby, University of Oxford (RAPIDE-BCV and TKM-Ebola); Dennis Malvy, University of Bordeaux (JIKI); James Neaton, University of Minnesota (PREVAIL).

Copyright © National Academy of Sciences. All rights reserved.

form.[8] In the Guinea ring vaccination trial, the team ensured that illiterate participants had an independent literate witness to provide consent in writing. While informed consent processes should be flexible and appropriate for the population and situation, it should also be recognized that some potential participants (e.g., those who may be forcibly confined or noncompetent patients without an available proxy) may simply not be suitable candidates for research studies because they are not able to give consent.

In emergency situations, however, it may be worth considering when an exception of informed consent may apply. Exception from informed consent allows researchers to enroll patients in certain emergency situations where consent cannot be given in advance, available treatments are unproven or unsatisfactory, and the research in question cannot be carried out without this waiver of informed consent. While this exemption facilitates the ease of enrollment of patients, it also requires the commitment of time and resources by the investigator, sponsor, and scientific and ethics review boards to ensure that potential host communities are openly and honestly informed about the risks and potential benefits associated with participation and given the opportunity to accept or decline to host the study in question. Even when communities are willing to host such studies, individuals may not wish to be included. Opt-out mechanisms, such as a wristband or bracelet, are often an ethics board or sponsor-required component of these studies and provide community members with the opportunity to indicate their prospective refusal to give consent to participation (HHS, 2013).

THE ROLE OF COMMUNICATION

Truthful, clear communication during an outbreak or epidemic is critical to successfully conveying public health messages, implementing infection control measures, and engaging communities in the entire process of response. There are a variety of ways to convey messages to communities, including radio, television, community meetings, and social media. Each of these has benefits and limitations, and the characteristics of the community should be taken into account when choosing a communication method (WHO, 2012). The success of response and research activities is contingent on community understanding of diseases, their mode of transmission and spread, public health control strategies, the availability of a proven therapy, and the research process. Experiences from previous

[8] Personal communication with Christopher McShane, Janssen Research & Development, LLC. COMAHS (Sierra Leone College of Medicine and Allied Health Sciences) and MoHS (Sierra Leone Ministry of Health and Sanitation). 2015. *Ebola flipcharts: A community Ebola marklate study in Sierra Leone.*

Copyright © National Academy of Sciences. All rights reserved.

Integrating Clinical Research into Epidemic Response: The Ebola Experience

outbreaks have shown that a paternalistic view of how to affect human behavior through provision of one-size-fits-all messaging is insufficient and ineffective, as "community understanding of diseases and their spread is complex, context dependent, and culturally mediated" (WHO, 2009, p. 6; see also Sugg, 2016). Communication programs targeted at low- and middle-income countries by Western, expert-led campaigns run the risk of casting individuals in those countries in the role of passive objects, rather than agents of their own change. The Ebola communication response ultimately went well beyond "messaging." Community dialogue, listening, and discussion—both face-to-face and through the media—were essential to bringing about change. "Communication is not something that happens to people," observed Bernhard Schwartlander of the World Health Organization. "You need to engage those that you want to reach in such a way that those communities take up the responsibility for communicating themselves" (Sugg, 2016, p. 16).

During the Ebola outbreak, early outreach and messaging efforts were confusing and counterproductive; the "public was initially inundated with complex information about Ebola transmission" (Bedrosian et al., 2016). A report by the All Party Parliamentary Group for Africa states that in Guinea, Liberia, and Sierra Leone in 2014, communication initially amplified the terrifying impact of the disease: "Initial communication campaigns focused on raising awareness about Ebola, informing people of the signs, symptoms, and how to seek help, but there was little effort to build the capacity of local journalists to spread accurate information and raise awareness" (Polygei, 2016, p. 38). Many messaging attempts by response workers were not informed by communication and behavioral sciences and did little to address underlying beliefs, including a pervasive idea that Ebola was not real. Many community members were convinced of this; in fact one small qualitative study in Sierra Leone revealed that nearly all of the participants did not think that Ebola was real (Yamanis et al., 2016). Furthermore, most feared that calling the national hotline for someone suspected of having Ebola would result in the person's death, and many said they would self-medicate if they developed a fever (Yamanis et al., 2016). Initial communications of the governments and international agencies included dramatic and fear-inducing messages such as "Ebola kills," "There is no cure for Ebola," and "Don't touch," which stigmatized those with the disease and deterred people from seeking care (Polygei, 2016). There were also reports that research messages may have occasionally interfered with other public health or response goals. For example, the Liberian Civil Society Organizations' Ebola Response Task Force expressed some concerns about the rollout of the PREVAIL vaccine trial in February 2015. The task force stated that the PREVAIL trial team, in spite of its efforts to engage the community in discussion, did not do an adequate job educating the public about

Copyright © National Academy of Sciences. All rights reserved.

the difference between the Ebola experimental vaccine and the standard childhood vaccination program and, consequently, was partly responsible for the low turnout of children to receive the standard vaccines to prevent childhood communicable disease (CSO-Ebola, 2015).

These missteps, however small, exacerbated community mistrust of responders as well as researchers and hampered efforts to treat patients or to recruit volunteers for clinical trials (Sugg, 2016). However, by the end of 2014, the increasing emphasis on communication interventions had contributed to a shift in public attitudes and understanding about Ebola. Public health professionals began empowering community organizers with insights and practical advice. Health professionals started to engage with local leaders and communities more systematically, listening to their concerns and ideas. They also began training local journalists and media hosts to provide accurate information. The CDC reported that "recognizing the need to simplify and coordinate messaging, CDC partners worked with the Sierra Leone National Ebola Response Centre to launch the Ebola Big Idea of the Week campaign. Approximately 80 radio, television, and print journalists from across the country were trained by experts on critical communication topics from CDC and other partner organizations. . . . In Guinea, coordinated communication strategies addressed cultural differences and focused on identifying trusted local spokespersons and Ebola survivors who could relate to diverse communities. . . . Central to the response was collaboration with these partners to deliver coordinated messages and avoid duplication of effort while respecting individuals and communities" (Bedrosian et al., 2016, p. 70).

In another example of effective communication, the Liberia DeySay project ("DeySay" is a reference to how people speak about rumors in Liberian English) attempted to identify and dispel rumors in real time (Iacucci, 2015). Hundreds of health workers, NGO staff, and volunteers on the ground were given a phone number and asked to send a text message when they became aware of a rumor circulating. A central coordination hub collected and analyzed the messages and sent information to local media partners, who could use their influence to help to dispel the rumors. DeySay also produced a weekly newsletter that highlighted the most critical rumors in circulation and advised media with insights on information gaps and health reporting (Iacucci, 2015).

Effective communication between different stakeholders and communities is critical to the epidemic response, and the success of research efforts is contingent upon successful communication within the broader epidemic response. Social and behavior change communication encompasses a range of approaches and tools, including interpersonal communication, work with mass media and other information and communication technologies, and social mobilization. Communication and social mobilization are dis-

Copyright © National Academy of Sciences. All rights reserved.

tinctive skill sets and require the participation of experienced experts. Early in an outbreak, all stakeholders should collaborate closely and harmonize the messages going to the public—for example, there should be no inconsistencies in the information or advice given to the public by the public health, clinical care, and research messages.

> ***Conclusion 6-4*** *Communication as part of the epidemic response is vitally important to the success of health care and public health measures as well as of clinical research. Increased funding for training and research into the science of culturally relevant communication to facilitate research and response during epidemics would lay the groundwork for better health and public health messaging to the general public both between and during epidemic emergencies.*

SUMMARY

Community engagement is a lengthy process, and outbreak response and clinical trial teams not only need to reach the community and provide information during an epidemic, but also must deal with preexisting knowledge and beliefs, whatever their origins or basis. A research team may bring new information and insights, but it never starts from zero when entering a new community during an epidemic. There are existing perceptions and beliefs that will influence how a community views the research and the researcher; in these contexts, history is not negligible. To gain community trust and to conduct valid, high-quality research, researchers must establish relationships with members and leaders throughout a community; this is even more challenging to do during an epidemic, when the fear of disease and death, rumors, and restrictions on movement interfere with the normal means of communication. However, to use resources wisely, respond most rapidly, and contain the epidemic quickly, there must be community engagement and a community-led response.

> **Recommendation 6a**
> ***Prioritize community engagement in research and response—Epidemic***
> **International and national research institutions, public health agencies, and humanitarian organizations responding to an outbreak should engage communities in the research and response by**
> 1. **Identifying social science experts in community engagement and communications to lead their efforts to effectively engage and connect with communities affected by the epidemic.**
> 2. **Consulting with key community representatives from the outset of an outbreak to identify a range of local leaders who can participate in planning research and response efforts, help to map community**

Copyright © National Academy of Sciences. All rights reserved.

assets, articulate how to infuse cultural and historical context into presentations, and identify gaps and risks in developing public health measures and designing research protocols. Consultations should be continued throughout the implementation phase by relevant actors to provide information as the outbreak evolves, provide feedback about progress and results, and inform and recommend changes to strategies based on feedback from the community.

3. **Coordinating within and across sectors, with national authorities, and with each other to ensure alignment of social mobilization and communication activities with the overall response and research strategies, and that there is sufficient support and training to local leaders and organizations to engage communities in research and response.**

As discussed in this chapter, successful community engagement and effective communication during a public health emergency or epidemic depend on the context, the specific community, and the particular goals of engagement and communication. This would no doubt be easier and less fraught with problems of trust if, during inter-epidemic periods, stakeholders invested more time, training, research, and funding into developing frameworks and strategies for community engagement and communication about health and public health that could be translated to the circumstances of an epidemic. Certainly in West Africa, the post-outbreak period is an opportunity to build on the successful efforts from later in the epidemic to connect with and engage communities in the research and response. This would be an ideal time to learn more about what did and did not work and about how to improve on the communication of health, public health, and health research messages. While there are many ongoing health concerns to address and there is a need to build better capacity and expertise, it is also an excellent time to continue to engage in dialogue about Ebola and, in particular, to share what was learned in each country from the clinical research that was done and how this information could be productively used in a future outbreak. Partnerships established during the outbreak can be leveraged to engage in this work and to solicit support for the dissemination of Ebola information and to develop a network between the newly established and strengthened public health units and the media and communication channels used by and in the communities.

Recommendation 6b

Fund training and research into community engagement and communication for research and response—Inter-epidemic

The World Health Organization, international research institutions, governments, public health agencies, and humanitarian organizations

Copyright © National Academy of Sciences. All rights reserved.

should actively collaborate together to fund training and research for developing frameworks, networks, strategies, and action plans for community engagement and communication on public health and research that could inform and be mobilized during an epidemic.

REFERENCES

Abramowitz, S. A., K. E. McLean, S. L. McKune, K. L. Bardosh, M. Fallah, J. Monger, K. Tehoungue, and P. A. Omidian. 2015. Community-centered responses to Ebola in urban Liberia: The view from below. *PLoS Neglected Tropical Diseases* 9(4):e0003706.

Act Alliance. 2014. *Appeal: Sierra Leone—Ebola sensitization and prevention in Sierra Leone—SLE141*. September 24, Geneva, Switzerland. http://reliefweb.int/sites/reliefweb.int/files/resources/SLE141_SLeone_Ebola.pdf (accessed January 25, 2017).

Afolabi, M. O., N. McGrath, U. D'Alessandro, B. Kampmann, E. B. Imoukhuede, R. M. Ravinetto, N. Alexander, H. J. Larson, D. Chandramohan, and K. Bojang. 2015. A multimedia consent tool for research participants in the Gambia: A randomized controlled trial. *Bulletin of the World Health Organization* 93:320–328A.

Ahmed, S. M., and A.-G. S. Palermo. 2010. Community engagement in research: Frameworks for education and peer review. *American Journal of Public Health* 100(8):1380–1387.

Bedrosian, S., C. Young, L. Smith, J. D. Cox, C. Manning, L. Pechta, J. L. Telfer, M. Gaines-McCollom, K. Harben, W. Holmes, K. M. Lubell, J. H. McQuiston, K. Nordlund, J. O'Connor, B. S. Reynolds, J. A. Schindelar, G. Shelley, and K. L. Daniel. 2016. Lessons of risk communication and health promotion—West Africa and United States. *Morbidity and Mortality Weekly Report* 65(Suppl 3):68–74.

Berndtson, K., T. Daid, C. S. Tracy, A. Bhan, E. R. M. Cohen, R. E. G. Upshur, J. A. Singh, A. S. Daar, J. V. Lavery, and P. A. Singer. 2007. Grand challenges in global health: Ethical, social, and cultural issues based on key informant perspectives. *PLoS Medicine* 4(9):e268.

Botbol-Baum, M., M. Brodin, A. Desclaux, F. Hirsch, C. Longuet, A.-M. Moulin, and B. Taverne. 2015. *From HIV to Ebola: Ethical reflections on health research in the global south and recommendations from Inserm and IRD*. Inserm Ethics Committee and IRD CCDE.

CDC (U.S. Centers for Disease Control and Prevention). 2015. *Launch of STRIVE Ebola vaccine trial*. https://www.cdc.gov/media/releases/2015/a0413-strive.html (accessed January 25, 2017).

CDC. 2016. CDC's response to the 2014–2016 Ebola epidemic—West Africa and United States. *Morbidity and Mortality Weekly Report* 65(3) [entire issue]. https://www.cdc.gov/mmwr/volumes/65/su/pdfs/su6503.pdf (accessed January 30, 2017).

Chaisson, L. H., N. E. Kass, B. Chengeta, U. Mathebula, and T. Samandari. 2011. Repeated assessments of informed consent comprehension among HIV-infected participants of a three-year clinical trial in Botswana. *PLoS ONE* 6(10):e22696.

Chang, M. S., E. M. Christophel, D. Gopinath, and R. M. Abdur. 2011. Challenges and future perspective for dengue vector control in the Western Pacific region. *Western Pacific Surveillance and Response Journal* 2(2):9–16.

CIOMS (Council for International Organizations of Medical Sciences). 2016. *International ethical guidelines for biomedical research involving human subjects*. http://www.cioms.ch/ethical-guidelines-2016 (accessed January 20, 2017).

Connolly, M. A., M. Gayer, M. J. Ryan, P. Salama, P. Spiegel, and D. L. Heymann. 2004. Communicable diseases in complex emergencies: Impact and challenges. *The Lancet* 364(9449):1974–1983.

Copyright © National Academy of Sciences. All rights reserved.

Integrating Clinical Research into Epidemic Response: The Ebola Experience

CSO–Ebola (Civil Society Ebola Response Task Force). 2015. *Liberia Civil Society Organizations Ebola Response Task Force: Policy brief*. February. http://lmcliberia.org/sites/default/files/Policy%20Brief%20on%20Vaccine%20Trials%20%2B%20Financial%20Tracking%20%20(final).pdf (accessed January 25, 2016).

DELWP (Department of Environment, Land, Water and Planning). 2013. *Types of engagement*. http://www.dse.vic.gov.au/effective-engagement/developing-an-engagement-plan/types-of-engagement (accessed January 25, 2017).

Dhillon, R. S., and J. D. Kelly. 2015. Community trust and the Ebola endgame. *New England Journal of Medicine* 373(9):787–789.

Diallo, D. A., O. K. Doumbo, C. V. Plowe, T. E. Wellems, E. J. Emanuel, and S. A. Hurst. 2005. Community permission for medical research in developing countries. *Clinical Infectious Diseases* 41(2):255–259.

Doe-Anderson, J., B. Baseler, P. Driscoll, M. Johnson, J. Lysander, L. McNay, W. S. Njoh, M. Smolskis, L. Wehrlen, and J. Zuckerman. 2016. Beating the odds: Successful establishment of a Phase II/III clinical research trial in resource-poor Liberia during the largest-ever Ebola outbreak. *Contemporary Clinical Trials Communications* 4:68–73.

Ebola Response Anthropology Platform. 2017. *About the network*. http://www.ebola-anthropology.net/about-the-network (accessed February 20, 2017).

Enria, L., S. Lees, E. Smout, T. Mooney, A. F. Tengbeh, B. Leigh, B. Greenwood, D. Watson-Jones, and H. Larson. 2016. Power, fairness and trust: Understanding and engaging with vaccine trial participants and communities in the setting up the EBOVAC–Salone vaccine trial in Sierra Leone. *BMC Public Health* 16:1140.

Faruque, A., A. Eusof, A. Q. Islam, S. Akbar, and M. Sarkar. 1985. Community participation in a diarrhoeal outbreak: A case study. *Tropical and Geographic Medicine* 37(3):216–222.

Fast, L. 2015. *On the importance of human connection: Fear, Ebola and security*. https://humanitarianhealthethics.net/2015/03/27/on-the-importance-of-human-connection-fear-ebola-and-security (accessed January 25, 2017).

Feuer, A. 2014. The Ebola conspiracy theories. *New York Times*. https://www.nytimes.com/2014/10/19/sunday-review/the-ebola-conspiracy-theories.html?_r=2 (accessed February 20, 2017).

Folayan, M. O., K. Peterson, and F. Kombe. 2015. Ethics, emergencies and Ebola clinical trials: The role of governments and communities in offshored research. *The Pan African Medical Journal* 22(Suppl 1):10.

George, A. S., V. Mehra, K. Scott, and V. Sriram. 2015. Community participation in health systems research: A systematic review assessing the state of research, the nature of interventions involved and the features of engagement with communities. *PLoS ONE* 10(10):e0141091.

Global Communities. 2015. *Assisting Liberians with Education to Reduce Transmission (ALERT)—Quarterly report*. April 30. Silver Spring, MD: Global Communities. http://pdf.usaid.gov/pdf_docs/PA00KN8V.pdf (accessed January 25, 2017).

HHS (U.S. Department of Health and Human Services). 2013. *Guidance for institutional review boards, clinical investigators, and sponsors: Exception from informed consent requirements for emergency research*. Silver Spring, MD: U.S. Food and Drug Administration. https://www.fda.gov/downloads/RegulatoryInformation/Guidances/UCM249673.pdf (accessed February 20, 2017).

HHS. 2016. *Informed consent information sheet: Draft guidance. July 2014*. Silver Spring, MD: U.S. Department of Health and Human Services. http://www.fda.gov/RegulatoryInformation/Guidances/ucm404975.htm (accessed January 25, 2017).

Iaccino, L. 2014. *Ebola "caused by Red Cross" and other conspiracy theories*. http://www.ibtimes.co.uk/ebola-caused-by-red-cross-other-conspiracy-theories-1469896 (accessed February 20, 2017).

Copyright © National Academy of Sciences. All rights reserved.

Iacucci, A. A. 2015. *Combatting rumors about Ebola: SMS done right*. https://medium.com/local-voices-global-change/combatting-rumors-about-ebola-sms-done-right-da1da1b222e8#.1ui1vbs1x (accessed January 25, 2017).

Jhanwar, V. G., and R. J. Bishnoi. 2010. Comprehensibility of translated informed consent documents used in clinical research in psychiatry. *Indian Journal of Psychological Medicine* 32(1):7–12.

Kickbusch, I., and K. S. Reddy. 2015. Community matters—Why outbreak responses need to integrate health promotion. *Global Health Promotion* 23(1):75–78.

King, N. 2014. *Empathetic and efficient PPE*. https://challenges.openideo.com/challenge/fighting-ebola/impact/empathetic-and-efficient-ppe (accessed January 25, 2017).

Kobayashi, M., K. D. Beer, A. Bjork, K. Chatham-Stephens, C. C. Cherry, S. Arzoaquoi, W. Frank, O. Kumeh, J. Sieka, A. Yeiah, J. E. Painter, J. S. Yoder, B. Flannery, F. Mahoney, and T. G. Nyenswah. 2014. Community knowledge, attitudes, and practices regarding Ebola virus disease—Five counties, Liberia, September–October, 2014. *Morbidity and Mortality Weekly Report* 64(26):714–718.

Laverack, G., and E. Manoncourt. 2015. Key experiences of community engagement and social mobilization in the Ebola response. *Global Health Promotion* 23(1):79–82.

Logan, G., N. M. Vora, T. G. Nyensuah, A. Gasasira, J. Mott, H. Walke, F. Mahoney, R. Luce, and B. Flannery. 2014. Establishment of a community care center for isolation and management of Ebola patients—Bomi County, Liberia, October 2014. *Morbidity and Mortality Weekly Report* 63(44):1010–1012.

Marais, F., M. Minkler, N. Gibson, B. Mwau, S. Mehtar, F. Ogunsola, S. Banya, and J. Corburn. 2016. A community-engaged infection prevention and control approach to Ebola. *Health Promotion International* 31(2):440–449.

McMillan, J., and C. Conlon. 2004. The ethics of research related to health care in developing countries. *Journal of Medical Ethics* 30(2):204–206.

Mohle-Boetani, J. C., M. Stapleton, R. Finger, N. H. Bean, J. Poundstone, P. A. Blake, and P. M. Griffin. 1995. Communitywide shigellosis: Control of an outbreak and risk factors in child day-care centers. *American Journal of Public Health* 85(6):812–816.

Mukpo, A. 2015. *Surviving Ebola: Public perceptions of governance and the outbreak response in Liberia*. London: International Alert. http://reliefweb.int/sites/reliefweb.int/files/resources/Liberia_SurvivingEbola_EN_2015.pdf (accessed January 25, 2017).

NBAC (National Bioethics Advisory Commission). 2001. *Ethical and policy issues in international research: Clinical trials in developing countries. Volume I: Report and recommendations of the National Bioethics Advisory Commission*. Bethesda, MD: National Bioethics Advisory Commission. https://bioethicsarchive.georgetown.edu/nbac/clinical/Vol1.pdf (accessed January 25, 2017).

NIAID (National Institue of Allergy and Infectious Diseases). 2015. *Liberia–U.S. clinical research partnership opens trial to test Ebola treatments*. https://www.niaid.nih.gov/news-events/liberia-us-clinical-research-partnership-opens-trial-test-ebola-treatments (accessed January 25, 2017).

Nuffield Council on Bioethics. 2002. *The ethics of research related to healthcare in developing countries*. http://nuffieldbioethics.org/report/research-developing-countries-2/chapter-downloads (accessed January 20, 2017).

Nyika, A., R. Chilengi, D. Ishengoma, S. Mtenga, M. A. Thera, M. S. Sissoko, J. Lusingu, A. B. Tiono, O. Doumbo, S. B. Sirima, M. Lemnge, and W. L. Kilama. 2010. Engaging diverse communities participating in clinical trials: Case examples from across Africa. *Malaria Journal* 9:86–86.

Okonta, P. I. 2014. Ethics of clinical trials in Nigeria. *Nigerian Medical Journal* 55(3):188–194.

Copyright © National Academy of Sciences. All rights reserved.

Onishi, N., and S. Fink. 2015. Vaccines face same mistrust that fed Ebola. *New York Times*, March 13. http://www.nytimes.com/2015/03/14/world/africa/ebola-vaccine-researchers-fight-to-overcome-public-skepticism-in-west-africa.html?_r=0 (accessed January 30, 2017).

Polygei. 2016. *Lessons from Ebola affected communities: Being prepared for future health crises*. Cambridge, UK: Polgeia. http://www.royalafricansociety.org/sites/default/files/reports/AAPPG_report_3.10_sngls_web.pdf (accessed January 25, 2017).

Samandari, T., T. B. Agizew, S. Nyirenda, Z. Tedla, T. Sibanda, N. Shang, B. Mosimaneotsile, O. I. Motsamai, L. Bozeman, M. K. Davis, E. A. Talbot, T. L. Moeti, H. J. Moffat, P. H. Kilmarx, K. G. Castro, and C. D. Wells. 2011. 6-month versus 36-month isoniazid preventive treatment for tuberculosis in adults with HIV infection in Botswana: A randomised, double-blind, placebo-controlled trial. *The Lancet* 377(9777):1588–1598.

Schulz-Baldes, A., E. Vayena, and N. Biller-Andorno. 2007. Sharing benefits in international health research. Research-capacity building as an example of an indirect collective benefit. *EMBO Reports* 8(1):8–13.

Sendolo, J. 2016. Dr. Golakai decries discrepancies in Ebola research. *Liberian Daily Observer*, August 18. http://www.liberianobserver.com/news/dr-golakai-decries-discrepancies-ebola-research (accessed January 25, 2017).

Sugg, C. 2016. *Coming of age: Communication's role in powering global health*. Policy Briefing No. 18. London, UK: BBC Media Action. http://www.bbc.co.uk/mediaaction/publications-and-resources/policy/briefings/role-of-communication-in-global-health (accessed January 25, 2017).

Wellcome Trust. 2011. *Community engagement—Under the microscope*. June 12–15, 2011. London, UK: Wellcome Trust. https://wellcome.ac.uk/sites/default/files/wtvm054326_0.pdf (accessed January 25, 2017).

WHO (World Health Organization). 2009. *Social mobilization in public health emergencies: Preparedness, readiness, and response: Report of an informal consultation*. Held on December 10–11, 2009. Geneva, Switzerland: WHO Press. http://apps.who.int/iris/bitstream/10665/70444/1/WHO_HSE_GAR_BDP_2010.1_eng.pdf (accessed January 25, 2017).

WHO. 2012. *Communication for behavioural impact (COMBI): A toolkit for behavioural and social communication in outbreak response*. Geneva, Switzerland: WHO Press. http://apps.who.int/iris/bitstream/10665/75170/1/WHO_HSE_GCR_2012.13_eng.pdf?ua=1 (accessed January 25, 2017).

Widdowson, M., S. Schrag, R. Carter, W. Carr, J. Legardy-Williams, L. Gibson, D. R. Lisk, M. I. Jalloh, D. A. Bash-Taqi, S. A. Sheku Kargbo, A. Idriss, G. F. Deen, J. B.W. Russell, W. McDonald, A. P. Albert, M. Basket, A. Callis, V. M. Carter, K. R. Clifton Ogunsanya, J. Gee, R. Pinner, B. E Mahon, S. T. Goldstein, J. F. Seward, M. Samai, and A. Schuchat. 2016. Implementing an Ebola vaccine study—Sierra Leone. *Morbidity and Mortality Weekly Report* 65(Suppl 3):98–106.

Wilson, B., J. B. Cooper, J. Lysander, K. Bility, J. D. Anderson, J. Endee, H. Kiawu, L. Doepel, and E. Higgs. 2016 (unpublished). *Engaging communities in clinical research during a public health emergency: An ethical norm*. Funded by National Institute of Allergy and Infectious Diseases, National Institutes of Health.

Yamanis, T., E. Nolan, and S. Shepler. 2016. Fears and misperceptions of the Ebola response system during the 2014–2015 outbreak in Sierra Leone. *PLoS Neglected Tropical Diseases* 10(10):e0005077.

Yates, D. A. 2015. PREVAIL admits neglect in promoting vaccine trials. *Liberian Daily Observer*, February 5. http://liberianobserver.com/columns-health/prevail-admits-neglect-promoting-vaccine-trials (accessed January 25, 2017).

Copyright © National Academy of Sciences. All rights reserved.

7

Facilitating International Coordination and Collaboration

The clinical trials conducted during the 2014–2015 Ebola epidemic were done in an atmosphere and on a timeline immensely different from most clinical trials. The fact that trials were conducted at all is a demonstration of the ability of researchers, regulators, scientific and ethics review boards, and communities to work together around the clock when the need is pressing—but despite this success, it was not without avoidable conflict along the way. The trial teams should be praised for overcoming the complex and intertwined logistical obstacles encountered while trying to design and implement trials in West Africa in the midst of a rapidly spreading and highly dangerous contagious disease epidemic. The limited health care, public health, and health research infrastructure; the bureaucracy; fear, rumors, and lack of trust; and supply chain hurdles were just some of the barriers that had to be addressed and overcome. Despite the many challenges, much was learned about conducting clinical trials in this type of environment—lessons that may help future trials be more successful. The clinical studies also succeeded in contributing to the base of scientific knowledge about Ebola, including the importance of physiological support and the identification of sequelae that had not been clearly delineated in past outbreaks (Chiappelli et al., 2015).

Despite the successes, however, the overall scientific harvest of the trials was described as "thin" (Cohen and Enserink, 2015). As discussed in Chapter 3, none of the therapeutic trials ended with conclusive results concerning product efficacy, although the limited evidence from the ZMapp trials did trend toward a possible benefit (PREVAIL II Writing Group, 2016). And, as discussed in Chapter 4, there were two Ebola vaccine can-

Copyright © National Academy of Sciences. All rights reserved.

Integrating Clinical Research into Epidemic Response: The Ebola Experience

didates studied that current data indicate may be safe and immunogenic, and one that is most likely protective, although further data on safety and efficacy are needed. However, given the resources, time, and effort that were put into these trials, they were not as successful as they could have been. The reasons for this are multiple and varied. First, when the initial serious discussions about pursuing clinical research were held by the World Health Organization (WHO) and other stakeholders in August 2014 they produced a long list of potential investigational agents, with various degrees of evidence to support their consideration. This led to trial teams independently selecting several different agents and ultimately competing for trial sites and patients rather than coordinating efforts and triage to focus on the most promising agents. Second, there was a lack of baseline information about the natural history of Ebola, clinical outcomes, and biomarkers that could inform patient care and clinical research. Third, several of the trials, as discussed in more detail in Chapters 3 and 4, had design issues limiting their chances of generating robust scientific data. Finally, many trials started too late in the course of the epidemic, launching as the outbreak was winding down. As a consequence even well-designed trials were unable to enroll a large enough participant population or to collect sufficient data to reach clear conclusions. One researcher reflecting on the experience stated that the "challenges . . . faced in the design, implementing, and reporting of the Ebola drug trials were not scientific, but political and administrative" (House of Commons Science and Technology Committee, 2016, p. 21). The committee concurs with the conclusion by the UK House of Commons Science and Technology Committee's report *Science in Emergencies: UK Lessons from Ebola*: "The failure to conduct therapeutic trials earlier in the outbreak was a serious missed opportunity that will not only have cost lives in this epidemic but will impact our ability to respond to similar events in the future" (House of Commons Science and Technology Committee, 2016, p. 25).

The fact that clinical trials began a few months after the epidemic peaked was in part due to the nature of dealing with an unpredictable, unprecedented outbreak; the initial focus on ramping up response to meet immediate need rather than research; delays in recognizing how rapidly the outbreak was expanding (despite alerts to the international community on the part of Médecins Sans Frontières [MSF]); and, additionally, problems of coordinating research and response on the part of international organizations also contributed to the delay. When the outbreak was first identified as Ebola in March 2014, it was unknown how far it would spread or how long it would last—previous outbreaks had been brought under control in just a few months after infecting at most a few hundred people. The initial priority in 2014 was to provide patient care and prevent further spread through public health measures, and the idea to do clinical

Copyright © National Academy of Sciences. All rights reserved.

trials for therapeutics or vaccines was not on the radar screen of the early responders and thus was overlooked.[1] Despite the warning signs on the ground, with historical precedent in mind it was difficult to foresee how the epidemic would actually unfold and whether trials would be possible. Nevertheless, had certain mechanisms been in place before the epidemic struck, clinical trials could likely have begun before the epidemic began to wane. Starting trials earlier would have potentially allowed them to enroll a sufficient number of patients to permit the full analysis intended, thus increasing the likelihood that the trials would result in the identification of one or more safe and effective treatments or vaccines for Ebola or at least an incremental increase in the knowledge base that could lead to better products in the future.

With these challenges in mind, the committee recognizes that several things will need to happen, often simultaneously, in order to execute clinical trials more efficiently in a future epidemic (see Figure 7-1). Substantial planning will be required in advance of the next epidemic in order to best position the international community to tackle these tasks, in partnership with affected countries. To enable a rapid prioritizing of investigational therapeutics and vaccines and the coordination of research efforts, the committee recommends a mechanism that will (1) foster collaborative investment in research and development (R&D) and (2) establish social trust and facilitate coordination among stakeholders.

INTER-EPIDEMIC PERIOD

The mobilization of a rapid and robust research response during the next epidemic will depend not just on what happens during the epidemic, but on what happens before and between epidemics—the inter-epidemic period. Building collaborative mechanisms, improving stakeholder relationships, and expanding investment into and planning of research during the inter-epidemic period will pay dividends when the next epidemic—of Ebola or another disease—strikes, regardless of where in the world it emerges.

[1] To prevent research from being overlooked in the future the United Kingdom is supporting a promising model by allocating funding to support a public health rapid support team to be jointly run by Public Health England and the London School of Hygiene & Tropical Medicine, with the University of Oxford and King's College London as academic partners. It will have the ability to deploy within 48 hours to anywhere in the world that requests assistance. The team's mission is to support national health systems to rapidly investigate and respond to disease outbreaks, and the team includes epidemiologists, microbiologists, experts in infectious disease control, social scientists, and experts in clinical research, thereby ensuring that research considerations including clinical research are included in response planning from the very beginning (PHE, 2016).

Copyright © National Academy of Sciences. All rights reserved.

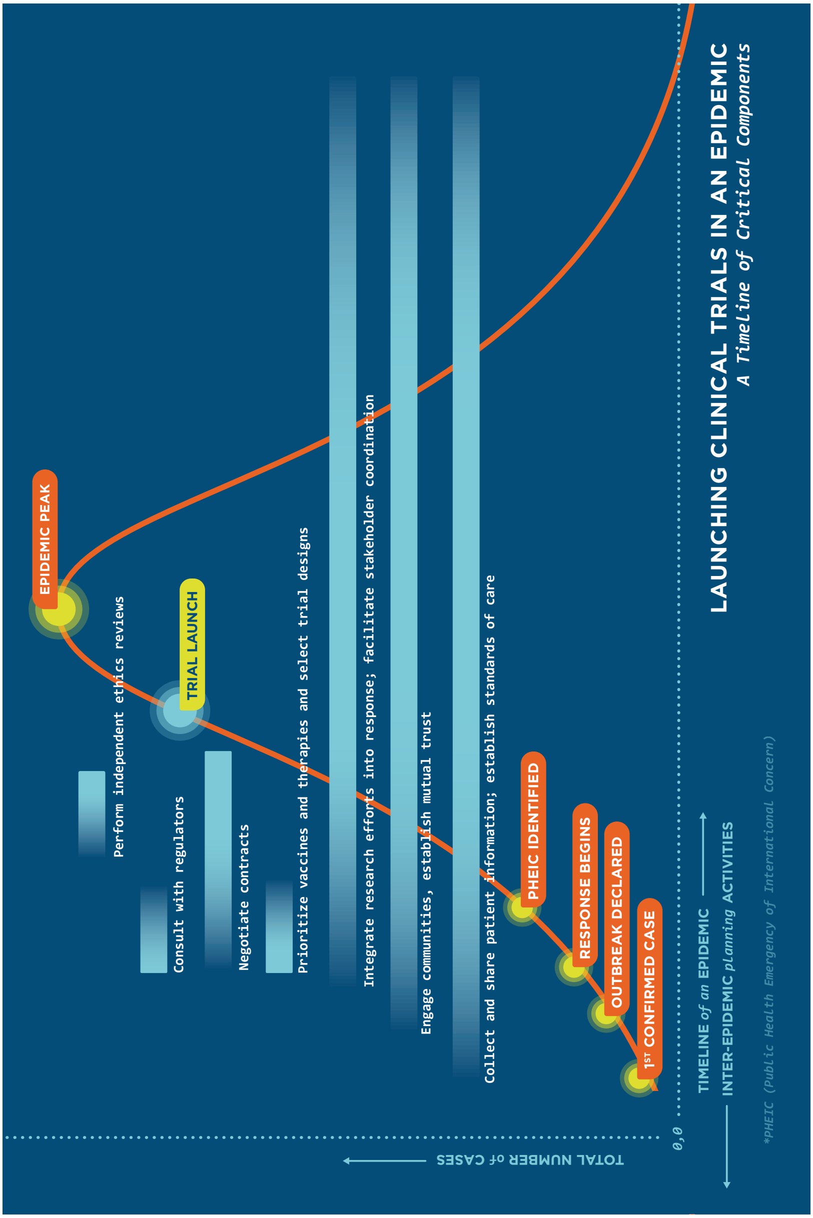

Copyright © National Academy of Sciences. All rights reserved.

FIGURE 7-1 A timeline of critical components: Launching clinical trials in an epidemic. The above figure represents an idealized timeline of activities necessary to launch a clinical trial and their relation to an epidemic curve. It should also be noted that each of these endeavors has inter-epidemic planning activities—if properly planned for, these inter-epidemic activities will contribute to faster trial implementation. (1) Patient information should begin to be collected upon identification of the first confirmed case. While this information contributes to pathogen identification and surveillance it is also critical as the outbreak progresses to collect data on routine care practices and outcomes to help establish standards of care. (2) Local communities must be engaged with both response and research efforts from the beginning of the outbreak. Response and research requires mutual trust and partnership with the community, and this trust and partnership is critical in carrying out public health tasks such as contact tracing and the prevention of disease transmission as well as to informing research protocols and obtaining informed consent from research participants. This should last throughout the epidemic response and research. (3) As the international response is launched clinical research experts should be engaged and research opportunities should be integrated and considered as part of the response planning from the very beginning. To accomplish this will require social trust and coordination among stakeholders. (4) A small working group of clinical research experts should objectively prioritize vaccine and therapeutic candidates with the most promising preclinical and clinical data to use in clinical trials in the affected region. The group should also select the most scientifically robust clinical trial designs for trial implementation and coordinate the research efforts to initiate research in concordance with an overall research agenda. (5) As investigational agents and trial designs are being prioritized the Rapid Research Response Workgroup (see Recommendation 7b) should engage with international regulators. In discussions with regulators the Workgroup should discuss pathways for expedited review, discuss pertinent data which regulators may be privy to that would influence the prioritization process (e.g., known toxicity data), prepare regulatory documents, and plan further consultations to facilitate licensure and approval of investigational products moved forward into clinical trials. (6) Negotiating contracts is a time-consuming process; as soon as pertinent institutions, countries, pharmaceutical firms, and manufacturers are identified template contracts should be adapted and negotiated. This includes everything from drug supply chain, customs documents, clinical trial agreements, post-trial access, and more. (7) Independent ethics review by the affected countries' ethics boards, the trial teams' institutions, and others are critical before any trial launch. If these seven steps are done in an efficient, coordinated, and timely manner, trials should be able to be launched before the peak of the epidemic.

Copyright © National Academy of Sciences. All rights reserved.

Research and Development

When the 2014–2015 epidemic began, there were a few Ebola-specific products in various stages of preclinical R&D, some of which had shown evidence of efficacy in animal models, including nonhuman primates. This preepidemic research was largely supported by a small set of funders, including civilian and military medical research and research funding agencies, albeit in line with the priority afforded to Ebola virus at the time—funding for Ebola R&D was limited. For example, a review of research funding in the United Kingdom from 1997 through 2013 reported zero Ebola research support out of £3.7 billion spent by public and philanthropic sources (Head et al., 2016). The U.S. National Institutes of Health (NIH) did not report disease-specific funding until 2010, but during the period 2010–2013, some $540 million was allocated to Ebola and other hemorrhagic fever virus research (Kliff, 2014). Several Canadian government agencies invested a total of around $25 million in the decade before the outbreak to support seminal work on what became ZMapp and the VSV-EBOV vaccine, representing an exceptionally good return on these early investments (Grant, 2014). G-FINDER[2] has recently provided, for the first time, an estimate of global research funding on Ebola during 2014 and of the proportional contribution of the U.S. government.

> Virtually all reported funding for Ebola R&D in 2014 came from the top 12 funders ($164 million, 99.7 percent). Apart from aggregate industry and the Gates Foundation, all of these were public sector institutions from North America and Europe. Three of the top five were U.S. government agencies: the NIH ($64 million, 39 percent), the U.S. HHS [Department of Health and Human Services] ($26 million, 16 percent), and the U.S. Department of Defense: Defense Threat Reduction Agency (DTRA, $11 million, 6.6 percent). Collectively, these three U.S. organizations provided 78 percent of all non-industry investment in Ebola R&D. (Moran et al., 2015, p. 31)

If not for these initial investments in research and early preclinical development, it is very unlikely that any products would have been any-

[2] G-FINDER is a uniquely informative data source, providing policy makers, funders, researchers, and industry with objective, previously unavailable information on the state of investment, trends, and patterns

- in 35 neglected diseases;
- across 142 product areas for these diseases including drugs, vaccines, diagnostics, microbicides and vector control products; and
- in platform technologies (e.g., adjuvants, delivery technologies, diagnostic platforms).

The data include all types of product-related R&D, including basic research, discovery and preclinical, clinical development, Phase IV and pharmacovigilance studies, and baseline epidemiological studies (Policy Cures, 2017).

Copyright © National Academy of Sciences. All rights reserved.

where near ready for clinical trials when the outbreak occurred. It should also be noted that in the most recent G-FINDER survey results released February 2017, with the notable exception of Ebola R&D, spending on neglected disease is at its lowest level since 2007 (Ross, 2017)—a dismal prospect if the global community hopes to be prepared in the event of a future outbreak.

The severity and rapid escalation of transmission of Ebola in West Africa during 2014 motivated the initiation of clinical trials, as the situation was so desperate and the epidemic would be the only opportunity to evaluate efficacy in humans. The usual process of drug development from bench to bedside[3] is estimated to, on average, take at least 10 years and cost $2.6 billion, with fewer than 12 percent of the products under development likely to be eventually licensed (DiMasi et al., 2016). Given the length of the typical Ebola outbreak and the length of time it takes to conduct drug discovery and assess safety and efficacy, the odds that a new compound could be discovered and fully evaluated during an outbreak are vanishingly small. Even with preliminary evidence, a drug in development with limited or no human safety and efficacy data would be very unlikely to gain regulatory approval on the basis of data generated during the outbreak and in time to be deployed during the same outbreak. Unless the data were especially promising, the likely best case scenario for a new drug or vaccine would be provisional approval for use in clinical trials or possibly for expanded access to high-risk groups, but not approval for the general population. Even with a limited expanded access approval, manufacturers would have to ramp up rapidly to make the product available before the epidemic waned.

The R&D of products—including therapeutics, vaccines, assays, and diagnostic tests—during the inter-epidemic period is the most likely pathway to ensure that promising candidates are available to study during an epidemic. Conducting Phase 1 safety trials during the inter-epidemic period (either in the country in which the product originated or in countries with populations at risk of the disease, or possibly both) could considerably facilitate the approval process and more rapid implementation of efficacy trials at the occurrence of an outbreak. The decision of whether to conduct Phase 1 trials in populations who have a near-zero risk of infection (i.e., in the country in which the product originated) will depend on a number of factors, including the specifics of the pathogen and of the investigational agent. For example, if the investigational product is suspected to have high toxicity or a vaccine is a live-attenuated vaccine, it may not be reasonable to perform this research in healthy participants in countries with a near-

[3] The term *bench to bedside* is used to describe the process by which the results of research done in the laboratory are directly used to develop new ways to treat patients (NCI, 2017).

Copyright © National Academy of Sciences. All rights reserved.

zero risk of infection. However, there are advantages to conducting Phase 1 studies in high-income settings; for example, given the greater resources and better infrastructure available, it may be easier to track and detect adverse events. During the Ebola epidemic it proved to be possible to recruit volunteers in high-income settings. A major advantage of conducting initial Phase 1 research in the country of product origin is that it may alleviate some of the distrust of the affected populations, diminish the concerns that their population is being used as "guinea pigs," and speed the approval and implementation of clinical trials during the outbreak. To conduct these types of activities during the inter-epidemic period will depend first on the existence of a vigorous R&D agenda; second, on a system for ongoing surveillance of known and potentially new and emerging pathogens; third, on a continuous process to assess priorities for research support, perhaps including incentives for private-sector R&D; and fourth, on sufficient vision and commitment from leaders inside and outside of government and science. The committee agrees with the assessment of the Ebola Vaccine Team B[4]—and for therapeutics as well—that the "need for Ebola vaccines (including multivalent filovirus vaccines) remains an urgent public health priority. Renewed and continued global leadership is required to complete the task of licensing and delivering safe, effective, and durable multivalent Ebola vaccines for prophylactic and reactive use. Achieving this outcome is critical not only for Ebola preparedness, but also for proof of concept that vaccines to protect against other neglected or emerging infectious diseases can be successfully developed in the future" (Ebola Vaccine Team B, 2017).

The WHO and its member states have recognized the importance of R&D in preparing for and responding to outbreaks. In October 2015, as part of the Oslo consultation, Financing of R&D Preparedness and Response to Epidemic Emergencies, participants proposed that stakeholders should aim to do the following (WHO, 2015b):

- Increase their overall investment in R&D for emergency preparedness.
- Align their different R&D efforts to address the global priorities identified in the WHO R&D blueprint under preparation as well as other ongoing processes.

[4] The Wellcome Trust and the Center for Infectious Disease Research and Policy (CIDRAP) at the University of Minnesota established the Ebola Vaccine Team B in November 2014 to support international efforts to stop the rapid spread of Ebola virus disease in West Africa. The group's purpose is to provide a complementary and creative review of all major aspects of developing and delivering effective and safe Ebola vaccines, including funding, research, development, vaccine efficacy and effectiveness determination, licensure, manufacturing, and vaccination strategies. The Wellcome Trust–CIDRAP Ebola Vaccine Team B includes 25 international subject-matter experts involved in one or more areas of vaccine work (Ebola Vaccine Team B, 2017).

Copyright © National Academy of Sciences. All rights reserved.

Integrating Clinical Research into Epidemic Response: The Ebola Experience

- Ensure efficient use of existing funding mechanisms by avoiding duplication of efforts.

The failure to support R&D for emerging infectious diseases can have severe consequences, as participants at the Oslo consultation discussed: "The lack of vaccines, drugs, and diagnostic tests for infectious diseases with epidemic or pandemic potential is a severe threat to global public health. [Ebola] in West Africa has taken a devastating human toll . . . [and] has also had a significant direct economic impact and continues to weaken the economies of the three hardest-hit countries with a projected $2.2 billion in lost GDP [gross domestic product] for 2015. Experiences with previous disease outbreaks (e.g., severe acute respiratory syndrome [SARS]) paint similar grim pictures" (WHO, 2015b). The idea that the international community should work together to address the gaps in R&D for priority pathogens that place populations at risk of epidemics seems obvious, particularly in hindsight. However, the historical problem remains: how to keep the focus of global leadership on the threat of future pandemic outbreaks and on supporting the vision of innovators in R&D, when there are so many different threats, even just for global health.

Operational Considerations

Conducting clinical trials requires addressing a litany of bureaucratic, legal, and ethical issues in addition to the scientific considerations. While Ebola trials were launched during the epidemic in record time, the lack of preplanning for research, and the sole focus on ramping up the critical humanitarian response in the early months of the international response resulted in the first patients not being enrolled in trials until the epidemic was already waning. At the time that the epidemic was still rapidly growing and spreading, researchers had to complete operational tasks such as establishing legal agreements, arranging for specimen analysis, and solving logistical issues. These challenges were compounded by the scarcity of resources and minimal research experience available in the affected countries, which at times led to perceptions of imbalances and mistrust between foreign sponsors and host countries. The imbalance in experience negotiating clinical trial contracts led to the perception, if not the reality, that foreign sponsors had much greater influence over the contracts than the in-country negotiators.[5] Additionally, because the lack of highly technical laboratory capacity present in country led to some of the clinical specimens

[5] Testimony of several workshop participants. Public Workshop of the Committee on Clinical Trials During the 2014–2015 Ebola Outbreak, August 15–17, 2016, Monrovia, Liberia.

Copyright © National Academy of Sciences. All rights reserved.

being exported for analysis (Schopper et al., 2016), the signing of material transfer agreements between host countries and researchers had "the potential of creating a lot of suspicion and mistrust if not well handled and documented" (Folayan et al., 2015, p. 2). Further, the fact that the Ebola-affected countries had relatively few and relatively inexperienced lawyers available to help execute material transfer agreements to get samples out of country and get investigational products into the country reportedly slowed response time on the part of the West African authorities and, consequently, resulted in additional delays in getting trials going. To address this common situation in low- and middle-income countries, the Council on Health Research for Development developed a Fair Research Contracting Initiative as a model program for low- and middle-income countries to adopt and enhance local competence (Marais et al., 2013).

These logistical and operational tasks "need to be done in days rather than weeks or months," says a researcher involved in the clinical trials conducted during the Ebola outbreak. She added that in order to address this issue, "research has to be embedded in the immediate response to an outbreak and not come as an afterthought" (Kelland, 2015). The committee strongly endorses this perspective. If emergency and epidemic response plans include ways to address these operational and logistical challenges, clinical researchers can overcome these hurdles more quickly and begin to evaluate potential agents to stop the outbreak more expeditiously than before, particularly if much of the general clinical trial planning work is done during the inter-epidemic period.

> ***Conclusion 7-1*** *Research and development is a complex and lengthy process that cannot be compressed into the course of a rapidly progressing outbreak. Prior investment in R&D is required during the inter-epidemic period for priority known pathogens and for the development of new approaches to speed the discovery and development of investigational products for emerging but still unknown or unrecognized pathogens.*

> ***Conclusion 7-2*** *Clinical trials can be more rapidly planned, approved, and implemented during an outbreak if (1) promising products have already been studied through Phase 1 or Phase 2 safety trials (when possible), particularly if there are preliminary efficacy data in a relevant animal model; and (2) if emergency response planning includes clinical research considerations and clinical researchers in the discussions from the beginning.*

Copyright © National Academy of Sciences. All rights reserved.

International Coordination

As discussed in Chapters 2–4 of this report, fortunately, there were a few Ebola-specific therapeutic and vaccine candidates in the R&D pipeline available for clinical research at the beginning of the outbreak. However, there was no a priori agreed-upon approach to prioritizing these candidates for clinical trials. The therapeutic category included not only untested Ebola-specific products, but also already-licensed drugs that could potentially be repurposed for Ebola and a variety of other proposed agents with little, if any, evidence or theory for their selection. There were also a few vaccine candidates in the pipeline at the time, with limited safety and efficacy information. With the long list of investigational agents competing for attention, the WHO-convened meetings aimed at harmonizing efforts were frequently tense and contentious as stakeholders not only disagreed on how to prioritize what to study, but also disagreed on how to design trials and debated issues such as randomization and the use of control groups. No infrastructure for the conduct of the trials was in place in the affected countries before the outbreak, nor was there a plan to coordinate across multiple studies to ensure that the available resources were used optimally to generate as much data as quickly as possible during the outbreak. The lack of coordination fostered competition among the trial teams over trial locations and trial participants, particularly as the epidemic waned and the number of new patients began to drop (Kupferschmidt, 2015). In its account of the Ebola outbreak, the UK House of Commons Science and Technology Committee (2016) reported the conclusions of Professor Trudie Lang of the University of Oxford about this lack of coordination: "[H]aving 'five different groups testing five different things' was 'not an overly sensible approach' since it resulted in an 'absurd situation' whereby a disorganised and 'unorchestrated throng of researchers' were each 'negotiating for access to patients' on the ground. [Professor Lang] stressed that 'better co-ordination' was needed in the future, combined with a more obvious prioritisation of research studies" (House of Commons Science and Technology Committee, 2016). Gelinas et al. (2017) recently explored the consequences of competition among similar clinical trials for participants, using as an example a hypothetical cancer center with multiple trials intended for the same patient population. Their conclusion was that "such a competition is a predictor of low study accrual, with increased competition tied to increased recruitment shortfalls . . . [and a] policy that prioritises some trials for recruitment ahead of others is ethically permissible and indeed prima facie preferable to alternative means of addressing recruitment competition" (Gelinas et al., 2017, p. 1).

In the future, in order to better gain stakeholder buy-in and increased cooperation in a coordinated research plan, it seems advisable to engage

Copyright © National Academy of Sciences. All rights reserved.

Integrating Clinical Research into Epidemic Response: The Ebola Experience

experienced meeting facilitators at stakeholder meetings, both in the inter-epidemic period and particularly at the start of an outbreak, to introduce and facilitate neutral and productive discussions among stakeholders and determine an agreed-upon process to adopt. Future meetings would also benefit from real-time stakeholder feedback to ensure the processes and goals are acceptable to all. According to former NIH ombudsman, Howard Gadlin, "ongoing assessment of process factors in teams is essential at the very beginning, at the midpoint, and at the end. . . . It's important for any group that's meeting to put aside time, even if it's just 5 minutes at the end of the meeting, to talk about how we did. What we handled well, what did we not handle well, what should we do differently in the future, paying attention to the emergence of group norms that may be somewhat counter-productive."[6]

It would be valuable and advantageous for the principal stakeholders, during the inter-epidemic period, to engage in early planning that is focused on the development of a list of priority pathogens to target for R&D, the creation of generic protocols, to establish memoranda of understanding, and to discuss material transfer agreements and other administrative details that would suddenly become high priority during an emerging infectious disease outbreak such as a central data repository. For example, in the case of protocols, the coalition of stakeholders discussed below would seek consensus about specific trial design issues for different priority pathogens, such as the population to be studied, the trial's primary endpoint including the potential role of surrogate measures, the use of individual versus cluster randomization, the feasibility of blinding the randomization, and approaches to improve efficiency by simultaneously evaluating complementary interventions such as through the use of factorial designs. These templates can be adapted to the specific circumstances of a particular outbreak and will speed up the planning, coordination, and approval process. This could become a part of an emergency outbreak document database (discussed in Chapter 5), perhaps posted and maintained by the WHO, and freely and openly available to everybody with interest. These tasks are time consuming, but they are expected necessities in launching clinical trials. Given the litany of bureaucratic tasks required for trials, creating an interactive social setting to develop these documents and reach agreements in advance of the next epidemic could result in a global community whose members are more comfortable with one another and who are better able and more willing to prioritize and collaborate in order to more quickly launch trials when necessary. These issues should be enlightened by the participation of experts from clinical and statistical science areas, from

[6] Testimony of Howard Gadlin, retired NIH ombudsman. Public meeting of the Committee on Clinical Trials During the 2014–2015 Ebola Outbreak, Washington, DC, June 15, 2016.

Copyright © National Academy of Sciences. All rights reserved.

academia, government, and industry, from ethics and regulatory bodies, from humanitarian nongovernmental organizations, and from foundations and at risk local communities.

Another outbreak similar to the Ebola epidemic will surely occur in the future. It is uncertain which pathogen will be the cause of the outbreak or in which geographical location the outbreak will occur, but it will happen. To enable research to begin much more quickly in this situation, it is essential to consider how to bring the various stakeholders together and to do it now. To this end, the committee recommends that an international coalition of stakeholders (ICS) be convened in order to improve inter-epidemic planning and coordination. Events on a global scale generally require a global solution, which in turn requires international coordination and cooperation. There are no events for which this is more applicable than emerging infectious diseases outbreaks, for even when initially localized within a country's borders such outbreaks can quickly become global. Within our recent memory, outbreaks due to severe acute respiratory syndrome, Middle East respiratory syndrome (MERS), Ebola, and now Zika have amply demonstrated the truth of this view. What remains is to determine how to most effectively make this process real. That is why the committee sought to consider existing models or organizations that could lead this effort, rather than recommending an entirely new entity be formed. The below discussion considers the WHO, the Global Health Security Agenda (GHSA), and the Coalition for Epidemic Preparedness Innovations (CEPI), a new multistakeholder organization created to "stimulate, finance, and coordinate the development of vaccines against epidemic infectious diseases, especially in cases in which market incentives alone are insufficient" (Røttingen et al., 2017).

> ***Conclusion 7-3*** *It is unrealistic to assume that all of the necessary planning and coordination activities for efficiently conducting clinical trials during an epidemic and avoiding unnecessary delays can take place after an outbreak begins and while it is ongoing. Activities that build relationships and address foreseeable problems in implementing a research program—such as determining how to evaluate competing trial proposals, deciding what should be included in clinical trial contracts, and educating national researchers and review boards in study conduct—must begin in the inter-epidemic period.*

> ***Conclusion 7-4*** *To increase the likelihood of success there is a need for an international coordination and collaboration mechanism to guide investment decisions, encourage broad participation of the global R&D community, and steward the process from early discovery to the registration of safe and effective products.*

Copyright © National Academy of Sciences. All rights reserved.

Recommendation 7a
Coordinate international efforts in research and development for infectious disease pathogens—Inter-epidemic
An international coalition of stakeholders with representation from governments, foundations, academic institutions and researchers, pharmaceutical companies, humanitarian organizations, and the World Health Organization (such as the Coalition for Epidemic Preparedness Innovations) should work on the following planning activities to better prepare for and improve the execution of clinical trials conducted during infectious disease events:

1. **Advise on and invest in priority pathogens to target for research and development, and promote a process to ensure that, whenever possible, interventions should be brought through Phase 1 or Phase 2 trials prior to an outbreak.**
2. **Develop generic clinical trial design templates for likely outbreak scenarios. The reasoning and rationale behind the designs and the situations in which each would be best utilized should be discussed with representatives of ethics review boards, major humanitarian organizations, and at-risk local communities to promote buy-in from stakeholders in advance of an outbreak.**
3. **Develop a list of key experts in clinical research from different agencies and organizations who could be rapidly seconded to the coalition of stakeholders and deployed anywhere in the world when an outbreak is first identified.**

DURING AN EPIDEMIC

While inter-epidemic planning and coordination may set stakeholders up for success, it is when an epidemic strikes that the rubber hits the road. Regardless of how much planning has been done before the epidemic, the steps that are taken in the early days of the outbreak set the course for the response and the potential for robust research. An epidemic presents the best—and sometimes only—opportunity to study a pathogen, the natural course of a disease, and the efficacy of investigatory treatments or vaccines. Care must be taken to conduct research efficiently and effectively and to use research designs that are most likely to produce reliable results, while considering feasibility and acceptability in the context of the epidemic. Each aspect of conducting research during an epidemic—following a research agenda, prioritizing agents for study, choosing trial designs, engaging with the community—can be best accomplished through a coordinated international effort. This committee recommends that upon the emergence of an epidemic, the ICS designate a rapid research response workgroup (R^3W) to coordinate the research response. This working group would appraise and

Copyright © National Academy of Sciences. All rights reserved.

prioritize the most promising agents to study, select the trial designs that are best suited to the context of the epidemic, and monitor and evaluate the clinical trials that are conducted during the outbreak. The workgroup should include stakeholders with expertise in areas such as the pathogen of concern, R&D of investigational interventions, clinical trial design, and ethics and regulatory review, and include representatives from the affected communities (see the following section for more details).

Recommendation 7b
Establish and implement a cooperative international clinical research agenda—Epidemic
In the event of an emerging epidemic the international coalition of stakeholders in Recommendation 7a should designate an independent multistakeholder rapid research response workgroup with expertise in the pathogen of concern, research and development of investigational interventions, clinical trial design, and ethics and regulatory review, and including representatives from the affected communities, to

1. **Rapidly appraise and prioritize a limited set of vaccine and therapeutic products with the most promising preclinical and clinical data for clinical trials.**
2. **Select a portfolio of trial designs that are best suited to the investigational agent(s) and the manifestation of the epidemic:**
   a. **The trial designs used should lead to interpretable safety and efficacy data in the most reliable and fastest way.**
   b. **Randomized trials are the preferable approach, and unless there are compelling reasons not to do so, every effort should be made to implement randomized trial designs.**
3. **Monitor and evaluate clinical trials conducted during an outbreak to enhance transparency and accountability.**

Stakeholder Coalition

In order to develop the international collaborative mechanisms described in Recommendations 7a and 7b (see Figure 7-2 for a diagram of Recommendations 7a and 7b), an ICS for product R&D and implementation of clinical research, including prioritization of products to be evaluated in clinical trials, will need to be identified. First, in order for it to be available to serve at the outset of an epidemic, the ICS must be organized in the inter-epidemic period and include all relevant stakeholders. This list is long and should include, for example, the WHO, research organizations in regions of the world where outbreaks are likely to occur, regional scientific groups and academic centers, large research organizations from developed countries with experience in global health and emerging infectious diseases

Copyright © National Academy of Sciences. All rights reserved.

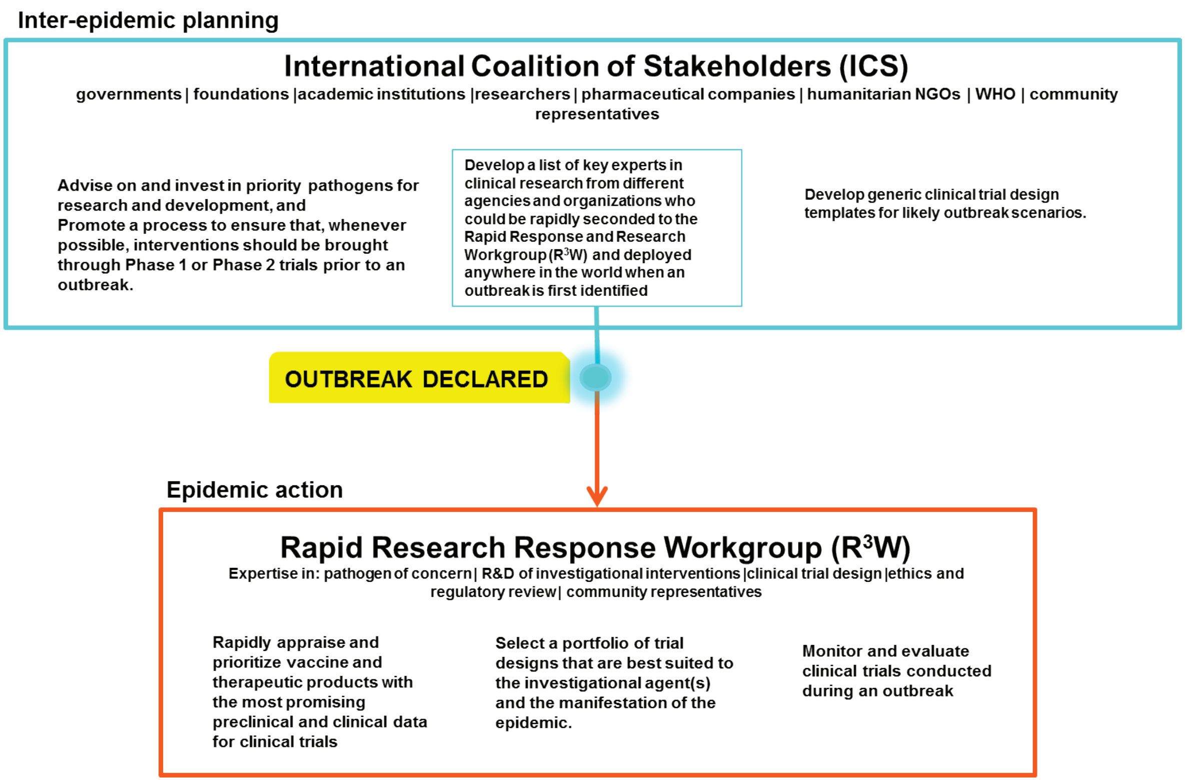

FIGURE 7-2 A visual representation of how the international coalition of stakeholders and rapid research response workgroup relate to one another, as suggested in Recommendations 7a and 7b.
NOTE: NGO = nongovernmental organization; R&D = research and development; WHO = World Health Organization.

Copyright © National Academy of Sciences. All rights reserved.

research, major research funders (e.g., NIH, Inserm, Wellcome Trust), pharmaceutical companies, regional research collaborations (e.g., Global Research Collaboration for Infectious Disease Preparedness [GLOPID-R], REACTing: REsearch and ACTion targeting emerging infectious diseases), as well as pan-African, Asian, and South American public health and research organizations. Through its interactions with WHO and its focus on the capacity for response, the ICS would involve international organizations providing ongoing care in locations where outbreaks are likely to happen (e.g., MSF, International Medical Corps, GOAL Global), as well as ethicists, and regulators.[7] The skillsets required are broad, but the number of people involved needs to be small enough for the ICS to work efficiently and be able to reach thoughtful consensus, perhaps by working through smaller subgroups or committees. Second, to be functional and efficient there is need for an autonomous expert working group, free of significant conflicts of interest, which has the mandate to make rapid decisions to shape and guide the clinical research agenda and prioritize which trials can go forward. This is the R^{3}W proposed in Recommendation 7b.

The ICS could play a very important strategic role in coordinating the interests of the research community and its potential to generate valuable new knowledge during an outbreak. It should work in tandem with the WHO, which has responsibilities for oversight and improvement of the 2005 International Health Regulations (IHR 2005) core capacities, and coordinates emergency response partnerships (Gostin and Katz, 2016). For example, as a part of its functions the ICS would need the input of R&D experts to help guide and focus the R&D agenda during the inter-epidemic period. It would then be ready to participate with WHO in the global emergency response planning from the very beginning of an outbreak and the declaration of a public health emergency of international concern. The ICS would also be in a preferred position to identify and delegate a smaller, highly expert group to form the R^{3}W, together with representatives from affected countries, to prioritize the products to go into clinical trials during the outbreak and coordinate and monitor their implementation. The R^{3}W could also provide support to the health research system in those countries as proposals are developed as a collaboration of international and national research institutions together with pharmaceutical and biotechnology companies, and are submitted for national scientific and ethical review, and, if accepted, implemented as soon as possible. Its

[7] Although regulators' role is not to choose the agents that should be investigated, they have a wealth of expertise in research design to contribute, and they may inform and broaden discussion and debate within the scientific community. In addition, regulators may be able to work with researchers in offering more flexible regulatory pathways and enabling rapid review during the time-sensitive part of an epidemic.

Copyright © National Academy of Sciences. All rights reserved.

hallmarks would be that it has R&D expertise, it is connected to but independent of both the coalition and the humanitarian emergency response team that convenes to guide the mobilization of global resources, and it is able to help integrate the clinical research opportunities into the planning process from the beginning. It would require broad-based connections to the international community, and the endorsement of the ICS, the WHO, and other relevant UN agencies, regulatory agencies in the countries of origin of products and in the countries experiencing the outbreak, and the research and manufacturing sectors. There would need to be explicit agreement forged during the inter-epidemic period that the decisions of the R^3W on priorities for moving a product into clinical research would be binding on all of the stakeholders.

The committee examined three possible entities that could take the lead in establishing such mechanisms for research governance, including the ICS and the R^3W: (1) the WHO, (2) the Global Health Security Agenda, and (3) the Coalition for Epidemic Preparedness Innovations.

World Health Organization

R&D for emerging infectious diseases is a vast challenge, and it requires depth in basic, translational, and clinical research expertise; focus; and a big budget. The WHO has many tasks to address, has limited technical R&D expertise among its staff, and is dependent on donor funds for its research activities. The various shortcomings of the WHO's performance over the 5 months from the time Ebola was confirmed in March 2014 until the WHO declared the public health emergency of international concern (PHEIC) on August 8, 2014, have subsequently been acknowledged by the organization (WHO, 2015c). This triggered a deep internal review, resulting in a plan to substantially improve the organization's future performance "to ensure that WHO maintains appropriate levels of organizational readiness, supports country-level capacity building and preparedness, deploys efficiently and effectively to respond to outbreaks and emergencies at national and subnational levels, and engages effectively with partners and stakeholders throughout" (WHO, 2015a, p. 1). The document discusses six major items and issues for WHO to adopt or address in order to improve performance:

- A unified platform for readiness and response to outbreaks and emergencies.
- A global health emergency workforce.
- Country-level IHR 2005 core capacities.
- The function, transparency, effectiveness, and efficiency of the IHR 2005.

Copyright © National Academy of Sciences. All rights reserved.

- A framework for R&D preparedness and enabling R&D during outbreaks or emergencies.
- International financing.

This is an enormous responsibility which will require substantial staff time and expertise to carry out, established and well-used communication mechanisms up and down the chain between the country offices and WHO Geneva, and process checks to ensure that the information flow is working and that there are enough well-trained staff available to carry out these responsibilities effectively and efficiently. WHO's analysis recognizes that "the world, including WHO, is ill-prepared for a large and sustained disease outbreak. . . . We have taken note of the constructive criticisms of WHO's performance and the lessons learned to ensure that WHO plays its rightful place in disease outbreaks, humanitarian emergencies and in global health security" (WHO, 2015c). After this reflection, WHO identified five key steps it needed to take in order to improve its performance in the future: first, take disease threats seriously; second, remain vigilant; third, help to reestablish the devastated services, systems, and infrastructure in Guinea, Liberia, and Sierra Leone; fourth, be transparent in reporting; and fifth, invest in research and development for the neglected diseases. It is worth considering whether the WHO ought to be the responsible party for all of the above tasks.

Without doubt, the WHO is an essential part of the international response to outbreaks of emerging infectious diseases. The effectiveness of the international response depends on how well the WHO focuses its attention for action and how well it partners with—and, when appropriate, cedes responsibility to—other organizations in order to harness their particular strengths, experience, and resources. For the WHO to cede responsibility for aspects of the broad international response required will be difficult unless the boundaries of responsibility among the various partners are clearly delineated in advance and effective mechanisms for communication and data sharing among the partners are established before an outbreak. In the committee's view, the WHO is not the optimal organization to shepherd the research and development of drugs and vaccines for emerging infectious diseases because it lacks the depth of expertise and the resources needed to support and undertake clinical research. The WHO must be at the table, but not as the chair, as it has enough to do already and needs to focus on doing that right as well. In this determination the committee finds itself in agreement with the Ebola Vaccine Team B analysis that "Despite the WHO's leadership role, it is not in a position to manage and fund all of the complexities associated with bringing Ebola vaccines to market. While the WHO can generate guidance documents, lead collaborations, and convene stakeholders through workshops and other platforms,

Copyright © National Academy of Sciences. All rights reserved.

the organization lacks the authority and extensive resources necessary to surmount some of the biggest remaining challenges associated with Ebola vaccine development" (Ebola Vaccine Team B, 2017). In recognition of these various concerns, and in a very positive step forward, the WHO has recently refined its role in the global R&D arena for emerging infectious diseases. "To fulfil its mandate, WHO has a core responsibility in the area of research and coordination of research. WHO will use its convening capacity to fulfil this responsibility. Although WHO is not a funding agency nor in general a major implementer of research activities, it has a global mandate to set evidence-based priorities and standards for research, ensuring that all voices are heard and avoiding conflicts of interests. Success of the R&D Blueprint will certainly depend on the concerted efforts of all stakeholders" (Kieny et al., 2016).

Global Health Security Agenda

Another model the committee considered is the Global Health Security Agenda (GHSA), a recent initiative to connect relevant parts of the U.S. government[8] with partners around the world on emerging infectious disease threats. The specific goal envisioned for GHSA is

> to advance a world safe and secure from infectious disease threats, to bring together nations from all over the world to make new, concrete commitments, and to elevate global health security as a national leaders-level priority . . . [and promote a] multilateral and multi-sectoral approach to strengthen both the global capacity and nations' capacity to prevent, detect, and respond to infectious diseases threats whether naturally occurring, deliberate, or accidental—capacity that once established would mitigate the devastating effects of Ebola, MERS, other highly pathogenic infectious diseases, and bioterrorism events." (GHSA, 2016b)

GHSA was launched in February 2014 (see Box 7-1, Global Health Security Agenda for GHSA's major commitments at the time of its launch) just as the Ebola outbreak was beginning to escalate but was still unrecognized. It was created as an expansion of the 2009 USAID Emerging Pandemic Threats program, which was designed to

> aggressively pre-empt or combat . . . diseases that could spark future pandemics. . . . [It is] composed of four complementary projects operating in 20 countries—PREDICT, PREVENT, IDENTIFY, and RESPOND—with

[8] Including HHS, the U.S. Department of State, the U.S. Agency for International Development (USAID), the U.S. Department of Defense (which includes the medical research organizations of the U.S. military), and the U.S. Department of Agriculture (which includes the agricultural research enterprise for animal diseases).

Copyright © National Academy of Sciences. All rights reserved.

BOX 7-1
Global Health Security Agenda

Over the next 5 years the United States commits to working with at least 30 partner countries to advance model systems to advance the Global Health Security Agenda.

The U.S. Centers for Disease Control and Prevention and the U.S. Department of Defense will work with other U.S. agencies and partner countries to establish emergency operations centers, build information systems, and strengthen laboratory security to mitigate biological threats and build partner capacity. In 2014 we will expand this effort to 10 additional partner nations.

In 2014, to effectively respond to outbreaks of disease with pandemic potential, the United States, in partnership with Canada and Mexico, will implement trilateral emergency communication protocols for information sharing among the health, agriculture, security, and foreign affairs sectors (CDC, 2014).

In 2014 the U.S. Department of Agriculture will partner with OIE [World Organisation for Animal Health, originally the Office International des Epizooties], FAO [Food and Agriculture Organization], and other nations to rapidly detect, diagnose and manage especially dangerous animal diseases in affected and high-risk countries.

In 2014 the U.S. Agency for International Development will launch its new Emerging Pandemic Threats (2) Program in 20 countries—providing technical and operational support for "preventing, detecting and responding to" new emerging zoonotic disease threats (USAID, 2016). In 2014, under the IHR (2005) framework, the United States will work with partners to strengthen National IHR Focal Point–related capacities, including the development of formal processes for the rapid assessment and notification of potential public health emergencies of international concern.

SOURCE: CDC, 2014.

> technical assistance from the U.S. Centers for Disease Control and Prevention (CDC). The Emerging Pandemic Threats global program draws on expertise from across the animal and human health sectors to build regional, national, and local 'One Health' capacities for early disease detection, laboratory-based disease diagnosis, rapid response and containment, and risk reduction. (USAID, 2016)

While GHSA is relevant to the goal of responding to emerging infectious diseases threats through international cooperation and collaborations, it is not an R&D program for therapeutics and vaccines. The principal basic and translational health research component of the U.S. government, NIH, and other similar research focused institutions internationally, are not significant partners in GHSA. Even without R&D, the rest of the GHSA

Copyright © National Academy of Sciences. All rights reserved.

is complex enough to fully occupy the attention of the involved agencies. GHSA currently lists 55 partner nations, including Guinea, Liberia, and Sierra Leone, as well as a number of international organizations and nongovernmental stakeholders such as the WHO, the UN Food and Agriculture Organization (FAO), the World Organisation for Animal Health, Interpol, the Economic Community of West African States, the UN Office for Disaster Risk Reduction, and the European Union (GHSA, 2014, 2016a). It now operates a set of 11 agreed-upon action packages which range across the themes of prevent, detect, and respond to emerging pandemic threats in order to "translate political support into action and to guide countries toward achieving the GHSA targets . . . by building capacity at a national, regional, and/or global level" (GHSA, 2014). Unfortunately, there is no indication on the GHSA website that any of the three West African nations affected by Ebola are contributing countries under any of the 11 action packages. The committee looks forward to GHSA addressing this very important agenda, but it does not consider GHSA the right structure to entrust with the R&D and clinical research agenda; furthermore, GHSA is driven by one country, and its priorities and commitments may change with changes in national leadership.

Coalition for Epidemic Preparedness Innovations

There are other new concepts for international coordination and cooperation more specifically targeted to R&D, including vital clinical research, for emerging epidemic diseases. A particularly interesting new entity is the Coalition for Epidemic Preparedness Innovations (CEPI), which was formally launched in January 2017 (Brende et al., 2017; CEPI, 2017). CEPI is being driven by five founding partners—the Norwegian Ministry of Foreign Affairs, the government of India, the Wellcome Trust, the Bill & Melinda Gates Foundation, and the World Economic Forum—with an expanding list of coalition partners (CEPI, 2016b). For example, it has also received large financial contributions from Japan and Germany (Brende et al., 2017). It is rapidly becoming operational under the leadership of an experienced interim chief executive officer, John-Arne Røttingen, previously the executive director of infection control and environmental health at the Norwegian Institute of Public Health and a professor of health policy at the Department of Health Management and Health Economics, Institute of Health and Society, University of Oslo, and chaired by Professor K. VijayRaghavan, Secretary of the Indian Department of Biotechnology. Because of the resources of the partners and the focus of its mission, CEPI has the potential for significant investments that can be used to dramatically speed up the development of vaccines for emerging infectious diseases, with the goal of raising $1 billion for its first 5 years of operation. According to CEPI,

Copyright © National Academy of Sciences. All rights reserved.

> The R&D response to the Ebola epidemic in West Africa was both a success and a failure. Never before have industry, government agencies, academia and NGOs [nongovernmental organizations] collaborated so effectively to plan and conduct more than a dozen clinical vaccine trials in less than a year. But it also showed that the R&D system is not prepared for these threats: we had not done the right research before the epidemic, causing needless delay and loss of life. CEPI will build on the spirit of working together that was ignited by Ebola to create a new R&D system for epidemics that several international panels have demanded. This partnership will give us the new vaccines we need for a safer world. (CEPI, 2016b)

A recent editorial in *Nature* stated, "At a time when short-termism and shortsightedness are rife, and political rhetoric often prevails over action, CEPI's founders are offering vision and foresight—it's an insurance policy that more governments, including the United States, would be well advised to back" (*Nature*, 2017). CEPI's approach to vaccine development is innovative, designed as

> an end-to-end approach—we won't take on discovery research or vaccine delivery, but we will work through all the steps in between. We will stay abreast of new discoveries and technologies, and we'll work with other organizations to make sure any vaccines that are developed reach those who need them. Equitable access will be a founding principle of CEPI, so that vaccines developed with its support are available to all who need them—price should not be a barrier—and they are available to populations with the most need. We expect that many of the vaccines CEPI helps to develop will not be profit-making, and we will work with our partners to ensure that the risks, costs and benefits of development are shared proportionately. (CEPI, 2016a)

CEPI's intent is to build on the WHO R&D Blueprint for Action to Prevent Epidemics, which is a good starting point to address the need for improved R&D preparedness for diseases of epidemic potential and for the ability to conduct responsive R&D in emergencies, to prioritize the pathogens of greatest interest and identify the R&D priorities, and to explore funding models for R&D preparedness and response (WHO, 2016). With the global recognition and significant financial and scientific resources of the founding partners, CEPI is already taking steps to lead international coordination and cooperation in vaccine development for emerging infectious diseases. For example, it organized a scientific conference which took place in February 2017 in collaboration with Inserm to assess progress in vaccine R&D for the WHO priority pathogens and other unknown pathogens with epidemic potential and to update the goals for vaccine R&D, manufacturing, and clinical development (CEPI, 2016c; Røttingen, 2016).

Copyright © National Academy of Sciences. All rights reserved.

CEPI has the power of the founding coalition and its resources to function as an independent scientifically driven clearinghouse for vaccines, and while the WHO is a CEPI partner, it is the rest of the coalition that brings the scientific and R&D strengths and resources.

Major prospective co-funders include NIH and the Biomedical Advanced Research and Development Authority within the Office of the Assistant Secretary for Preparedness and Response in HHS; the European Community and the European Union's Innovative Medicines Initiative and the European & Developing Countries Clinical Trials Partnership; public- and private-sector implementers and innovators, such as multinational corporations, research institutes, and product development partnerships; regulators and normative bodies (e.g., the U.S. Food and Drug Administration, the European Medicines Authority, the WHO PreQualification Programme, and the African Vaccine Regulatory Forum); national academies of medicine or science; and procurement and distribution partners such as the Global Alliance for Vaccines and Immunization. The committee recognizes that CEPI is a model that is still early in its development and is focused on vaccine development, but it also recognizes that if CEPI is successful in the vaccine arena, it could in the future tackle the need to coordinate and cooperate on the development of new safe and effective therapeutics. It has the "right DNA for the job," and we are hopeful that it will quickly evolve and be willing to take on the broader role envisioned by the committee.

EMBEDDING RESEARCH INTO RESPONSE

There will be a need to connect the proposed ICS and R^{3}W with other international response agencies during an epidemic and with the leadership of national governments affected by an outbreak from its very onset in order to ensure that the affected population has a partnership position in the response. Together, the response and research agencies and organizations can share the responsibility and allocate resources efficiently and effectively so that the goals of the response and research activities are clear and agreed upon, and that community engagement and communication strategies are aligned. One way to get research at the table from the beginning would be to include representation from the proposed ICS on the WHO IHR Emergency Committee constituted under IHR 2005 which is responsible for advising the WHO director-general whether an outbreak should be identified as a PHEIC; it is this that triggers the international response to contain the outbreak and help to care for infected individuals (WHO, 2017).

Because the tasks and burdens at the beginning of an outbreak are complex and involve multiple stakeholders, there should be thoughtful consideration given in the inter-epidemic period to developing an epidemic

Copyright © National Academy of Sciences. All rights reserved.

response stakeholder engagement strategy that includes a process for rapid mapping of key stakeholders at multiple levels (i.e., national to international, and national to local leaders and opinion formers) at the onset of an epidemic. The goal is to encourage an open dialogue among all relevant stakeholders to achieve a better understanding of the nature of the crisis, each stakeholders' interests, and resources available for addressing the epidemic, inclusive of the potential for research in the response.

SUMMARY

From the outset of the committee's work, we have focused on the goal of identifying ways to improve the speed and effectiveness of clinical research during an epidemic of an emerging infectious disease. This has involved the committee considering the many complex issues that are at the core of good clinical research. We have been aware of the multiplicity of issues that impinge on the task of optimizing clinical research in these circumstances. As discussed in greater detail in the preceding chapters, clinical trials require a diverse range of expertise, from scientific and medical experts to those who are adept at law, ethics, and community engagement. It is not possible to consider how to improve the speed and efficiency of clinical research on an emerging infectious disease without reflecting on the need to determine that an outbreak is beginning or that a new or neglected agent is emerging; the first step in the chain is to have effective and sustainable surveillance in place within countries, connected to a global community with expertise and resources to deploy once the need is identified and a response is triggered. The world can certainly do better in this regard than it has up until now. The next step in the chain is to be certain that there is the vision, expertise, and resources to support essential early research on priority pathogens spanning the spectrum from discovery, pathogenesis, and early R&D on diagnostics, drugs, and vaccines.

Due to the complexity of the activities involved in the design and conduct of trials, their implementation in the midst of a rapidly progressing outbreak requires quick action and immense coordination and collaboration among stakeholders, from the countries affected to the international community involved in the global response. Developing a document database for research (as discussed in Chapter 5) that includes model documents for all of the administrative processes required for approval and implementation of clinical research in these circumstances, that has a variety of model research designs available to be adapted to the particular attributes of the agent and the outbreak, and that includes the tools for ethical and legal review would help to strengthen research systems and guide affected countries to more quickly understand the lifespan of the research process and be better equipped to act as effective partners. In order to be rapidly

Copyright © National Academy of Sciences. All rights reserved.

effective when an outbreak is recognized, the health care, public health, and health research communities will require training in the nature and use of these tools. This is why the committee is convinced that coordinated planning and capacity strengthening for clinical research must start in the inter-epidemic period and why research must continue to be considered a critical part of the response as an outbreak begins and the initial response teams enter the affected communities. Engaging the community at every step is essential in order to avoid conflicts, to establish trust, and to prevent problems that may lead to premature trial closure, or prevent them from ever beginning (Folayan et al., 2015).

If national and international researchers can work together on a collaborative and coordinated research agenda, and include input from the population at risk, the global community has the best chance at being prepared for the next outbreak. As Louis Pasteur said a long time ago, "Chance favors the prepared mind" (Pasteur, 1854). It can also be said that preparation is not without cost, in fact significant sums are required; however, considered as an investment in global health and security these amounts pale in the comparison to the cost of confronting an epidemic and the potentially catastrophic loss of life and global resources if we are unprepared, uncoordinated, and without global participation.

REFERENCES

Brende, B., J. Farrar, D. Gashumba, C. Moedas, T. Mundel, Y. Shiozaki, H. Vardhan, J. Wanka, and J.-A. Røttingen. 2017. CEPI - A new global R&D organisation for epidemic preparedness and response. *The Lancet* 389(10066):233–235.

CDC (U.S. Centers for Disease Control and Prevention). 2014. *U.S. commitment to the global health security agenda: Toward a world safe & secure from infectious disease threats*. https://www.cdc.gov/globalhealth/security/pdf/ghs_us_commitment.pdf (accessed January 25, 2017).

CEPI (Coalition for Epidemic Preparedness Innovations). 2016a. *CEPI: Approach*. http://cepi.net/approach (accessed January 25, 2017).

CEPI. 2016b. *CEPI: Partners*. http://cepi.net/partners (accessed January 25, 2017).

CEPI. 2016c. *CEPI newsletter*, November 21. http://cepi.net/sites/default/files/52016.pdf.

CEPI. 2017. *Global partnership launched to prevent epidemics with new vaccine*. http://cepi.net/cepi-officially-launched (accessed February 20, 2017).

Chiappelli, F., A. Bakhordarian, A. D. Thames, A. M. Du, A. L. Jan, M. Nahcivan, M. T. Nguyen, N. Sama, E. Manfrini, F. Piva, R. M. Rocha, and C. A. Maida. 2015. Ebola: Translational science considerations. *Journal of Translational Medicine* 13:11.

Cohen, J., and M. Enserink. 2015. Special report: Ebola's thin harvest. *Science*, December 31. http://www.sciencemag.org/news/2015/12/special-report-ebolas-thin-harvest (accessed January 26, 2017).

DiMasi, J. A., H. G. Grabowski, and R. W. Hansen. 2016. Innovation in the pharmaceutical industry: New estimates of R&D costs. *Journal of Health Economics* 47:20–33.

Copyright © National Academy of Sciences. All rights reserved.

Ebola Vaccine Team B. 2017. *Completing the development of Ebola vaccines: Current status, remaining challenges, and recommendations*. Regents of the University of Minnesota. http://www.cidrap.umn.edu/sites/default/files/public/downloads/ebola_team_b_report_3-011717-final_0.pdf (accessed February 20, 2017).

Folayan, M. O., K. Peterson, and F. Kombe. 2015. Ethics, emergencies and Ebola clinical trials: The role of governments and communities in offshored research. *The Pan African Medical Journal* 22(Suppl 1):10.

Gelinas, L., H. F. Lynch, B. E. Bierer, and I. G. Cohen. 2017. When clinical trials compete: Prioritising study recruitment. *Journal of Medical Ethics* 0:1–7.

GHSA (Global Health Security Agency). 2014. *Global Health Security Agenda: Action packages*. https://www.ghsagenda.org/packages (accessed January 26, 2017).

GHSA. 2016a. *Global Health Security Agenda: Membership*. https://www.ghsagenda.org/members (accessed January 26, 2017).

GHSA. 2016b. *Global Health Security Agenga: About*. https://www.ghsagenda.org/about (accessed January 26, 2017).

Gostin, L. O., and R. Katz. 2016. The International Health Regulations: The governing framework for global health security. *Milbank Quarterly* 94:264–313.

Grant, K. 2014. How Canada developed pioneer drugs to fight Ebola. *The Globe and Mail*, August 22. http://www.theglobeandmail.com/life/health-and-fitness/health/how-canada-developed-pioneer-drugs-to-fight-ebola/article20184581 (accessed January 26, 2017).

Head, M. G., J. R. Fitchett, V. Nageshwaran, N. Kumari, A. Hayward, and R. Atun. 2016. Research investments in global health: A systematic analysis of UK infectious disease research funding and global health metrics, 1997–2013. *EBioMedicine* 3:180–190.

House of Commons Science and Technology Committee. 2016. *Science in emergencies: UK lessons from Ebola: Second report of session 2015–16*. Report No. HC 469. London, UK: House of Commons. http://www.publications.parliament.uk/pa/cm201516/cmselect/cmsctech/469/46902.htm (accessed January 26, 2017).

Kieny, M. P., J.-A. Rottingen, and J. Farrar. 2016. The need for global R&D coordination for infectious diseases with epidemic potential. *The Lancet* 388(10043):460–461.

Kelland, K. 2015. INSIGHT–MERS, Ebola, bird flu: Science's big missed opportunities. *Reuters*, October 26. http://www.reuters.com/article/health-epidemic-research-idUSL8N12831D20151026 (accessed January 27, 2107).

Kliff, S. 2014. No, budget cuts aren't the reason we don't have an Ebola vaccine. *Vox*, October 16. http://www.vox.com/2014/10/16/6987825/ebola-budget-nih-collins-vaccine (accessed February 1, 2017).

Kupferschmidt, K. 2015. Scientists argue over access to remaining Ebola hotspots. *Science*, March 26. http://www.sciencemag.org/news/2015/03/scientists-argue-over-access-remaining-ebola-hotspots (accessed January 26, 2017).

Marais, D., J. Toohey, D. Edwards, and C IJsselmuiden. 2013. *Where there is no lawyer: Guidance for fairer contract negotiation in collaborative research partnerships*. Geneva, Switzerland: Council in Health Research for Development. http://www.cohred.org/wp-content/uploads/2012/04/Fair-Research-Contracting-Guidance-Booklet-e-version.pdf (accessed January 25, 2017).

Moran, M., N. Chapman, L. Abela-Oversteegen, V. Chowdhary, A. Doubell, C. Whittall, R. Howard, P. Farrell, D. Halliday, and C. Hirst. 2015. *Neglected disease research and development: The Ebola effect*. Sydney, Australia: Policy Cures. http://policycures.org/downloads/Y8%20GFINDER%20full%20report%20web.pdf (accessed January 25, 2017).

Nature. 2017. Vaccine initiative marks bold resolution. 436.

NCI (National Cancer Institute). *NCI Dictionary of Cancer Terms*. https://www.cancer.gov/publications/dictionaries/cancer-terms?cdrid=561321 (accessed March 8, 2017).

Copyright © National Academy of Sciences. All rights reserved.

Integrating Clinical Research into Epidemic Response: The Ebola Experience

Pasteur, L. 1854. *Inaugural address as newly appointed professor and dean (Sep 1854) at the opening of the new Faculté des Sciences at Lille (7 Dec 1854)*. https://todayinsci.com/P/Pasteur_Louis/PasteurLouis-Quotations.htm (accessed January 26, 2017).

PHE (Public Health England). 2016. *UK team of health experts to tackle global disease outbreaks*. https://www.gov.uk/government/news/uk-team-of-health-experts-to-tackle-global-disease-outbreaks (accessed January 26, 2017).

Policy Cures. 2017. *G-FINDER*. http://policycures.org/gfinder.html (accessed February 20, 2017).

PREVAIL II Writing Group. 2016. A randomized, controlled trial of ZMapp for Ebola virus infection. *New England Journal of Medicine* 375(15):1448–1456.

Ross, E. 2017. Ebola funding surge hides falling investment in other neglected diseases. *Nature*. doi:10.1038/nature.2017.21505.

Røttingen, J.-A. 2016. *Coalition for Epidemic Preparedness Innovations (CEPI): Presentation to the WHO*. Geneva, Switzerland: World Health Organization. http://www.who.int/medicines/ebola-treatment/TheCoalitionEpidemicPreparednessInnovations-an-overview.pdf (accessed January 25, 2017).

Røttingen, J.-A., D. Gouglas, M. Feinberg, S. Plotkin, K. V. Raghavan, A. Witty, R. Draghia-Akli, P. Stoffels, and P. Piot. 2017. New vaccines against epidemic infectious diseases. *New England Journal of Medicine* 376(7):610–613.

Schopper, D., R. Ravinetto, L. Schwartz, E. Kamaara, S. Sheel, M. J. Segelid, A. Ahmad, A. Dawson, J. Singh, A. Jesani, and R. Upshur. 2016. Research ethics governance in times of Ebola. *Public Health Ethics* phw039. doi: 10.1093/phe/phw039. https://academic.oup.com/phe/article-abstract/doi/10.1093/phe/phw039/2548977/Research-Ethics-Governance-in-Times-of-Ebola?redirectedFrom=fulltext (accessed February 1, 2017).

USAID (U.S. Agency for International Development). 2016. *Emerging Pandemic Threats 2 Program*. https://www.usaid.gov/news-information/fact-sheets/emerging-pandemic-threats-program (accessed January 25, 2017).

WHO (World Health Organization). 2015a. *Follow up to the World Health Assembly decision on the Ebola virus disease outbreak and the Special Session of the Executive Board on Ebola: Roadmap for Action*. Geneva, Switzerland: WHO. http://www.who.int/about/who_reform/emergency-capacities/WHO-outbreaks-emergencies-Roadmap.pdf (accessed January 25, 2017).

WHO. 2015b. *Outcome document: Financing of R&D preparedness and response to epidemic emergencies*. October 29–30, 2015. Oslo, Norway, and Geneva, Switzerland: WHO. http://www.who.int/medicines/ebola-treatment/Outcome.pdf (accessed January 25, 2017).

WHO. 2015c. *WHO leadership statement on the Ebola response and WHO reforms*. http://www.who.int/csr/disease/ebola/joint-statement-ebola/en (accessed January 25, 2017).

WHO. 2016. *A research and development blueprint for action to prevent epidemics: Plan of action. May 2016*. Geneva, Switzerland: WHO. http://www.who.int/csr/research-and-development/r_d_blueprint_plan_of_action.pdf?ua=1 (accessed January 26, 2017).

WHO. 2017. *IHR Committees and expert roster*. http://www.who.int/ihr/procedures/ihr_committees/en (accessed February 20, 2017).

Copyright © National Academy of Sciences. All rights reserved.

Appendix A

Study Approach and Methods

In response to a request by the Office of the Assistant Secretary for Preparedness and Response, the National Institute of Allergy and Infectious Disease, and the Food and Drug Administration of the U.S. Department of Health and Human Services, the National Academies of Sciences, Engineering, and Medicine's Committee on Clinical Trials During the 2014–2015 Ebola Outbreak was charged with exploring the scientific and ethical issues related to clinical trials conducted in Guinea, Liberia, and Sierra Leone between 2014 and 2015. The committee's final report will inform guidelines and best practices for the design, conduct, and reporting of clinical trials in response to a future outbreak.

COMMITTEE EXPERTISE

The National Academies formed a committee of 16 experts to conduct an 18-month study to deliberate and respond to the statement of task. The committee was composed of individuals with expertise in clinical trial investigations and ethics review committees experience, international health law, regulatory and health systems oversight, public engagement and local/community and cultural perspectives, biostatistics and clinical trial design, clinical infectious disease science and case management, crisis management, and emergency preparedness and response. Representation included U.S., European, and African participants as well as a consultant on clinical trial methodology, Janet Darbyshire.

Copyright © National Academy of Sciences. All rights reserved.

MEETINGS AND INFORMATION-GATHERING ACTIVITIES

The committee deliberated from January to December 2016, during the course of which it held six in-person meetings (February, March, June, August, September, and November). The February, March, June, and August meetings included portions open to the public, and the open session agendas for those sessions appear below. The committee meetings in September and November were held only in closed session.

To inform its deliberations the committee gathered information through a variety of mechanisms: (1) three 3-day workshops with open public sessions; (2) one 2-hour webinar in May with international regulators; (3) one open public comment session during its June meeting; (4) systematic literature reviews of the scientific, ethical, social, and anthropological issues and other pertinent background research; (5) solicitation and consideration of written statements from stakeholders and members of the public through the committee's Current Projects System website and committee e-mail; and (6) personal communication between committee members and staff and individuals who have been directly involved in or have special knowledge of the issues under consideration.

IDENTIFYING WEST AFRICAN EXPERTS FOR A LOCAL PERSPECTIVE

The committee held a 2-day public workshop in August 2016 in Monrovia, Liberia, where it spoke with local experts from Guinea, Liberia, and Sierra Leone knowledgeable about scientific research, ethical review and pharmaceutical regulations, Ebola clinical care, survivors of Ebola, and social mobilization and gathered their input on a wide range of topics related to the committee's charge, including (1) the clinical trials conducted during the Ebola outbreak, (2) ethical and social implications of clinical trials being conducted in the three countries, (3) the role of the community in implementing clinical trials, and (4) inter-epidemic planning. Participants for this public workshop were identified through conversations with committee members, anthropologists who worked in country, suggestions from trial sponsors, and published news and literature discussing pertinent players during the Ebola outbreak. This meeting included an open public comment session during which input was invited from any interested parties.

TRIAL ANALYSIS

As part of the statement of task, the committee was asked to assess the scientific validity of the information that arose as a result of the clinical trials conducted during the 2014–2015 Ebola outbreak. While numerous

Copyright © National Academy of Sciences. All rights reserved.

attempts to study investigational agents occurred in West Africa during this time including through compassionate use, observational studies, and other investigations of therapies that lacked sufficient detail on protocol or results—the trials assessed by the committee were chosen based on the trial location (Guinea, Liberia, or Sierra Leone) and on whether or not a formal clinical trial was conducted. The committee reviewed the trials based on available trial results in the literature as well as on other publications or presentations that addressed the scientific, ethical, and logistical considerations of each trial team.

LITERATURE AND PRESS REVIEW

The committee and staff conducted a literature search that was concentrated on journals found in the following databases: Medline, Embase, PubMed, Scopus, Web of Science, Anthropology Plus, Proquest, African Journals Online, African Index Medicus, ClinicalTrials.gov, and Northern Light. Broad search terms were used to cast as wide a net as possible. The articles obtained by use of the search terms were reviewed for their relevance to the committee's charge. Other targeted literature reviews were conducted throughout the committee's deliberations as novel issues arose.

Clinical Trial Design and Conduct

Search Parameters:
- Date range: all years
- International, English only

Databases:
- Scopus
- Web of Science
- Embase and Medline
- Proquest
- Northern Light

Search Strategy:

Adaptive Clinical Trials
- TITLE-ABS ("clinical trial" AND ("adaptive randomized trial" OR "adaptive trial" OR "platform trial*" OR "new Bayesian" OR "Bayesian adaptive" OR "Bayesian meta-analysis"))

Historical Controls
- TITLE (("historical") AND ("randomized clinical trial" OR "randomized trial" OR rct OR "clinical trial"))

Copyright © National Academy of Sciences. All rights reserved.

Clinical Trials During Outbreaks

- TITLE-ABS((“infectious disease” or ebola or cholera or AIDS or HIV or “avian flu” or “avian influenza” or MERS or “Middle East respiratory syndrome” or “Marburg virus” or “viral haemorrhagic fever” or Legionnaires or “meningococcal disease” or “acute haemorrhagic fever syndrome” or SARS or “severe acute respiratory syndrome”)) w/10 outbreak and “clinical trial*”)

Ethics of Randomization

- TITLE ((ethics OR ethical) AND randomization) OR ABS ((ethics OR ethical) AND randomization) AND TITLE-ABS-KEY (“high risk” OR “infectious disease” OR “high mortality”))

Informed Consent

- (informed consent or consent or informed decision).mp. AND (understanding or comprehension or retention or knowledge or awareness or recall).mp. AND (biomedical research or clinical research or clinical trials or randomi*ed controlled clinical trials or random allocation trials or intervention trials).mp. AND Africa/ OR (lowincome countr* or developing countr* or vulnerable populations or disadvantaged populations or underserved populations).mp.

Clinical Trials in Developing Countries

- randomization and ethics and (Asia or Africa or Thailand or “developing countr*”)

Anthropology

Search Parameters:

- Date range: all years;
- International, English only

Databases:

- PubMed
- Anthropology Plus
- AnthroSource
- ClinicalTrials.gov

Search Strategy:

Compassionate Use

- Search terms: compassionate use, undue inducement, standard of care Ebola, community engagement, informed consent

Community Acceptance of Clinical Trials

- Search terms: Ebola, therapeutic misconception, consent, community engagement, clinical trial, Ebola treatment unit, vaccination, consent, surveillance, quarantine

Copyright © National Academy of Sciences. All rights reserved.

PUBLIC COMMITTEE MEETING AGENDAS

Meeting 1: Washington, DC; February 22–23

Public Workshop of the Committee on Clinical Trials During the 2014–2015 Ebola Outbreak

First Committee Meeting
February 22–23, 2016
Keck Center: 500 Fifth Street, NW
Washington, DC 20001
Room 100

Day 1
Monday, February 22

CLOSED COMMITTEE SESSION
8:00 a.m.–4:00 p.m.

OPEN SESSION

4:00 p.m. **Opening Remarks to Public Audience**

- **Gerald Keusch,** *Committee Co-Chair*, Professor of Medicine and International Health, Boston University Schools of Medicine and Public Health

- **Keith McAdam,** *Committee Co-Chair*, Emeritus Professor of Clinical and Tropical Medicine, London School of Hygiene & Tropical Medicine

4:05 p.m. **Delivery of Study Charge and Q&A/Discussion with Committee**

Objectives:
- Receive study background and charge from the U.S. National Institutes of Health, National Institute of Allergy and Infectious Diseases (NIH–NIAID).
- Discuss task with the sponsor and determine scope of committee's work (i.e., what is in and what is out).
- Clarify issues identified by the committee and seek answers to questions.
- Discuss report audience and expected products.

Copyright © National Academy of Sciences. All rights reserved.

Moderators: Gerald Keusch and Keith McAdam, *Committee Co-Chairs*

Sponsor Panelists:
- **Tony Fauci**, Director, NIAID
- **Cliff Lane**, Deputy Director for Clinical Research and Special Projects; Director, Division of Clinical Research, NIAID

5:00 p.m. ADJOURN Open Session

Day 2
Tuesday, February 23

OPEN SESSION

9:00 a.m. **Opening Remarks to Public Audience**

- **Gerald Keusch,** *Committee Co-Chair*, Professor of Medicine and International Health, Boston University Schools of Medicine and Public Health

- **Keith McAdam,** *Committee Co-Chair*, Emeritus Professor of Clinical and Tropical Medicine, London School of Hygiene & Tropical Medicine

9:05 a.m. **Delivery of Study Charge and Q&A/Discussion with Committee**

Objectives:
- Receive study background and charge from the Office of the Assistant Secretary for Preparedness and Response (ASPR) and the U.S. Food and Drug Administration (FDA).
- Discuss task with the sponsor and determine scope of committee's work (i.e., what is in and what is out).
- Clarify issues identified by the committee and seek answers to questions.
- Discuss report audience and expected products.

Moderators: Gerald Keusch and Keith McAdam, *Committee Co-Chairs*

Copyright © National Academy of Sciences. All rights reserved.

Sponsor Panelists:

- **Nicole Lurie**, Assistant Secretary for Preparedness and Response, Office of the Assistant Secretary for Preparedness and Response (ASPR)

- **Luciana Borio**, Acting Chief Scientist, U.S. Food and Drug Administration (FDA)

10:00 a.m. ADJOURN Open Session

CLOSED COMMITTEE SESSION
10:00 a.m. – 4:00 p.m.

Copyright © National Academy of Sciences. All rights reserved.

Meeting 2: London, UK; March 22–24

Public Workshop of the Committee on Clinical Trials During the 2014–2015 Ebola Outbreak

Second Committee Meeting
March 22–24, 2016
British Medical Association
BMA House
Tavistock Square
London WC1H 9JP
United Kingdom

Day 1
Tuesday, March 22, 2016

Meeting Objectives:

- Explore the design and implementation of clinical trials during the 2014–2015 Ebola outbreak.
- Examine the cultural, public health, and ethical context surrounding the respective designs of Ebola clinical trials; highlight important takeaways for future trials in a similar emergency context.
- Discuss the scientific and public health gains from clinical trials during the Ebola outbreak and identify lessons learned to improve a future international response to a public health emergency in a low-resourced country.
- Consider the role of international bodies (governments, regulatory agencies, nongovernmental organizations, academicians, and others) in a rapid, robust, and sustained response.

9:45 a.m. Welcome by Committee Co-Chairs

- **Keith McAdam,** *Committee Co-Chair*, Emeritus Professor of Clinical and Tropical Medicine, London School of Hygiene & Tropical Medicine
- **Gerald Keusch,** *Committee Co-Chair*, Professor of Medicine and International Health, Boston University Schools of Medicine and Public Health

Copyright © National Academy of Sciences. All rights reserved.

10:00 a.m. Opening Presentation: *Presentation and Q&A*

Bridging the Divide: Connecting Clinician, Patient, and Researcher

- **Ian Crozier,** Infectious Disease Specialist, Physician

SESSION I – PERSPECTIVES ON THE COMMUNICATION OF CLINICAL RESEARCH DURING AN EMERGENCY

(*60 min*; *10-min panelist presentations followed by 30-min discussion and Q&A*)

10:30 a.m. Objectives:

- Explore the role of public trust and rumor management in the communication and implementation of clinical trials.
- Examine how local understanding of existing clinical care and clinical research influence community acceptance of trials.

Moderator: **Sheila Davis**, Chief Nursing Officer, Partners In Health

Panelists:

- **James Fairhead,** Chair, Social Anthropology, University of Sussex
- **Heidi Larson,** Senior Lecturer, London School of Hygiene & Tropical Medicine

SESSION II – CLINICAL TRIAL DESIGN AND IMPLEMENTATION: REFLECTIONS ON THE CLINICAL TRIALS CONDUCTED DURING THE 2014–2015 EBOLA OUTBREAK

(*Session II will consist of three panels and extend after lunch; speakers are encouraged to stay throughout the entire session*)

Objectives:

- Discuss the considerations that were taken into account in the design of the trial (i.e., meeting scientific and ethical standards, health systems infrastructure, time to trial launch, public opinion, need of the affected population, etc.).

Copyright © National Academy of Sciences. All rights reserved.

- Discuss any alternative trial designs considered leading up to implementation of the trial; explore why particular designs were selected.
- Explore the role of the trialist, if any, in selecting the interventions used in the EVD trials; discuss the considerations that go into advancing experimental compounds into clinical trials.
- Discuss the trial results, where available, and explore the scientific and public health value in the data derived from each study. What, if anything, would you do differently next time to achieve greater gains from trials?

Moderators: **Janet Darbyshire,** Emeritus Professor of Epidemiology, University College London; and **Abdel Babiker,** Professor of Epidemiology and Medical Statistics, Medical Research Council Clinical Trials Unit, University College London

11:30 a.m. Overview Presentation (*20 mins*):

- **Peter Smith,** Professor, London School of Hygiene & Tropical Medicine

11:50 a.m. **Panel 2A. Vaccine Trials Conducted During the Ebola Outbreak**

(*60 min; 10-min panelist presentations followed by 40-min discussion and Q&A*)

Panelists:

- **Johan van Hoof,** Global Therapeutic Area Head, Infectious Diseases and Vaccines, Janssen Research & Development, LLC – *EBOVAC-Salone*
- **Ana Maria Henao-Restrepo,** Medical Officer at the Initiative for Vaccine Research (IVR), Department of Immunization Vaccines and Biologicals, WHO – *Guinea Ring Vaccine*

12:50 p.m. LUNCH

Copyright © National Academy of Sciences. All rights reserved.

1:50 p.m. **Panel 2B. Therapeutic Trials Conducted During the Ebola Outbreak**
(*85 min*; *10-min panelist presentations followed by 45-min discussion and Q&A*)

Panelists:
- **Trudie Lang,** Professor, University of Oxford
- **John Whitehead,** Emeritus Professor, Lancaster University – *RAPIDE-BCV, TKM-Ebola*
- **Annick Antierens,** Medical Department, Médecins Sans Frontières
- **Johan van Griensven,** Professor, Institute of Tropical Medicine–Antwerp – *Ebola-Tx*
- **France Mentre,** Professor of Biostatistics, Université Paris Diderot, Paris, France – *JIKI*

3:15 p.m. BREAK

3:30 p.m. **Panel 2C. Panel Reflections and Considerations for the Design of Clinical Trials**
(*60 min; 10-min panelist presentations followed by 40-min discussion and Q&A*)

Objectives:
- Discuss lessons learned and explore how future approaches to clinical trials in a public health emergency may be similar and/or different.
- Identify innovative approaches to research in emergency contexts; consider options that facilitate flexible and accelerated approaches.
- Consider whether adjustments to research standards in an outbreak are appropriate.

Panelists:
- **Peter Smith,** Professor, London School of Hygiene & Tropical Medicine
- **Geneviève Chêne,** Professor, University of Bordeaux

Copyright © National Academy of Sciences. All rights reserved.

SESSION III – PUBLIC HEALTH CONTEXT

(30 min; 10-min panelist presentation followed by 20-min discussion and Q&A)

4:30 p.m. Objectives:

- Explore strategies for how different stakeholders (for example, nongovernmental organizations, clinicians, health ministers, and international researchers) could work together to address a public health emergency.
- Consider how best incorporate research into the public health response in the event of an outbreak in a low-resource setting.
- In the context of a public health emergency in a low-resource setting, examine where international organizations can best cooperate and invest to build sustainable in-country clinical research systems.
- Discuss lessons learned from other outbreak situations (e.g., severe acute respiratory syndrome [SARS]) and explore how those experiences could have informed the Ebola response, reflect on strategies for applying lessons learned in the future.

Moderator: **David Peters,** Professor, Johns Hopkins Bloomberg School of Public Health

Panelist:

- **David Heymann,** Head of the Centre on Global Health Security, Chatham House

5:00 p.m. ADJOURN

Copyright © National Academy of Sciences. All rights reserved.

Day 2
Wednesday, March 23, 2016

SESSION IV – ETHICAL CONSIDERATIONS IN THE CONDUCT OF CLINICAL TRIALS DURING AN EMERGENCY
(*60 min; 10-min presentations followed by 40-min discussion*)

10:00 a.m. Objectives:

- Explore how the ethical principles for clinical trial conduct are applied in a low-resource outbreak setting, consider:
  - Scientific validity and health value of the study
  - Fair subject selection and subject respect
  - Risk–benefit ratio; equipoise
  - Informed consent
- Discuss how a public health emergency may impact the ethical considerations involved in clinical trial design and conduct—explore what, if any of the principles are inviolable.

Moderator: **Olayemi Omotade,** Professor of Pediatrics and Child Health, University of Ibadan

Panelists:

- **François Hirsch,** Senior Research Director, Institut national de la santé et de la recherche médicale (Inserm)
- **Jonathan Montgomery,** Professor of Health Care Law, University College London, Chair, Nuffield Council on Bioethics

CLOSED SESSION – COMMITTEE ONLY
11:15 a.m.–1:25 p.m.

SESSION V – PREPARING FOR AND FINANCING CLINICAL TRIALS
(*60 min; 10-min panelist presentations followed by 40-min discussion and Q&A*)

1:30 p.m. Objectives:

- Explore how the broader research community can work together during the inter-epidemc period to prepare for and improve the execution of clinical trials.

Copyright © National Academy of Sciences. All rights reserved.

- Identify the biggest local and international roadblocks in designing and implementing clinical trials in West Africa. Discuss how international bodies be better situated to respond next time.
- Consider methods to develop a sustainable research system, e.g., standard implementable clinical trial protocols, training local research staff, establishing regional health technologies and infrastructure.

- Moderator: **Fred Wabwire-Mangen,** Associate Professor of Epidemiology and Public Health, Makerere University

Panelists:

- **Jimmy Whitworth,** Professor, London School of Hygiene & Tropical Medicine
- **Marguerite Koutsoukos,** Director Ebola and HIV programs, GlaxoSmithKline (GSK)

CLOSED SESSION – COMMITTEE ONLY
2:35 p.m.–3:15 p.m.

OPEN SESSION

3:30 p.m. **Q&A with Jeremy Farrar,** Director, Wellcome Trust

4:00 p.m. ADJOURN

Copyright © National Academy of Sciences. All rights reserved.

Day 3
Thursday, March 24, 2016

SESSION VI – ETHICAL AND SCIENTIFIC CONSIDERATIONS FOR PRIORITIZING RESEARCH DURING OUTBREAKS

(*60 min; 10-min panelist presentations followed by 40-min Q&A*)

10:00 a.m. Objectives:

- Explore what evidence is needed when evaluating potential treatment options to determine the most viable candidates for further development and advancement to clinical trials.
- Discuss how, in the context of an international emerging or re-emerging infectious disease event, clinical trials can best be prioritized.
- Explore the common goals and trade-offs in health care and clinical research.

Moderator: **Alex John London,** Professor, Carnegie Mellon University

Panelists:

- **Miles Carroll,** Head of Research Microbiology Service, Public Health England (PHE)
- **Carel IJsselmuiden,** Executive Director, Council on Health Research for Development (COHRED) Group, South Africa

11:00 a.m. ADJOURN

Copyright © National Academy of Sciences. All rights reserved.

Integrating Clinical Research into Epidemic Response: The Ebola Experience

Meeting 3: Webinar; May 19

Public Webinar of the Committee on Clinical Trials During the 2014–2015 Ebola Outbreak

May WebEx Session: 2-Hour Webinar

May 19, 2016

REGULATORY CONSIDERATIONS

Meeting Objectives:

- Discuss product regulators' thinking about standards of evidence for approval of experimental products in a rapidly progressing infectious disease epidemic.
- In situations involving multiple experimental agents at relatively early stages of development, consider how regulators should prioritize which agent to advance.
- Identify the key considerations for prioritizing and implementing clinical trials when there is a limited supply of product available or (as in the waning of an outbreak) the potential for insufficient participants for a statistically valid analysis. Furthermore, is randomization imperative in this context?
- Explore whether and how regulatory agencies, key funders, and other stakeholders in different countries can coordinate the assessment and implementation of clinical trials for experimental products during an infectious disease outbreak.

Panelists:

- **Robert Hemmings,** Unit Manager, Statistics and Pharmacokinetics Unit, Medicines and Healthcare Products Regulatory Agency (MHRA), UK
- **Peter Marks,** Director, Center for Biologics Evaluation and Research, U.S. FDA
- **Edward M. Cox,** Director of Antimicrobial Products (OAP), U.S. FDA
- **Marco Cavaleri,** Head of Anti-Infectives and Vaccines, European Medicine Agency

Copyright © National Academy of Sciences. All rights reserved.

Hour 1: **Welcome by Moderator and Speaker Introductions** (*5 min*)

Moderator: **Michelle Mello,** Professor of Law and Professor of Health Research and Policy, Stanford University

Discussion: Standards of Evidence (*45 min*)

- Each agency has 5 minutes for opening remarks followed by committee discussion and Q&A

Hour 2: **Discussion: Prioritization and Collaboration** (*45 min*)

- Each agency has 5 minutes for opening remarks followed by committee discussion and Q&A

Open Discussion and Q&A with Committee (*25 mins*)

ADJOURN

Copyright © National Academy of Sciences. All rights reserved.

Meeting 4: Washington, DC; June 13–15

Public Workshop of the Committee on Clinical Trials During the 2014–2015 Ebola Outbreak

Third Committee Meeting
June 13–15, 2016
Keck Center: 500 Fifth Street NW
Washington, DC 20001
Room 208

Day 1
Monday, June 13, 2016

Meeting Objectives:

- Consider how to best align the missions and values of international stakeholders (governments, regulatory agencies, nongovernmental organizations, academic and industry researchers) to engender a rapid, robust, and sustained public health and research response.
- Explore strategies and identify resources needed to effectively conduct clinical trials during an emergency without negatively impacting the public health and humanitarian response.
- Discuss the ethical and scientific considerations in the design and implementation of clinical trials during the 2014–2015 Ebola outbreak; identify challenges and lessons learned, including issues around consent, community engagement, managing data, etc.
- Explore the full economic impact of outbreaks, and discuss how sustainable funding for clinical research during public health outbreaks can be established and managed.

SESSION I – FOSTERING INTERNATIONAL COORDINATION AND COLLABORATION

Objectives:

- Explore how nations with strong response capacity can work more effectively together under the leadership of international organizations like the WHO.
- Consider how U.S. and other international institutions can cede the role of lead coordinating organization

Copyright © National Academy of Sciences. All rights reserved.

for emergency response while still maintaining their autonomy.

8:30 a.m. Welcome by Committee Co-Chairs
- **Gerald Keusch,** *Committee Co-Chair*, Boston University Schools of Medicine and Public Health
- **Keith McAdam,** *Committee Co-Chair*, London School of Hygiene & Tropical Medicine

8:45 a.m. Opening Presentation:
(*15- to 20-min presentation followed by Q&A*)

Lessons from Past Epidemics
- **Adel Mahmoud,** Woodrow Wilson School of Public and International Affairs and Department of Molecular Biology, Princeton University

9:15 a.m. Moderator: **Kathryn M. Edwards,** Vanderbilt University School of Medicine

Panelists:
(*90 min; 10-min opening remarks by each panelist, followed by discussion and Q&A)*
- **Margaret A. Hamburg,** Foreign Secretary, National Academy of Medicine
- **Inger K. Damon,** Ebola Response Team Incident Commander, Centers for Disease Control and Prevention

10:45 a.m. BREAK

SESSION II – THE FEASIBILITY OF CLINICAL RESEARCH DURING HUMANITARIAN EMERGENCIES

Objectives:
- Explore strategies and identify resources needed to effectively conduct clinical trials during an emergency without overburdening clinical care givers.
- Consider the feasibility of using existing clinical care facilities established by NGOs/non-research-based organizations for research activities during an emergency.
- Discuss approaches to bridge the divide between clinical care and medical research staff to find commonalities and improve the research response.

Copyright © National Academy of Sciences. All rights reserved.

- Consider how resources to support clinical trials in humanitarian emergencies might be prepositioned. Could there be a team of neutral ethics experts assembled to help low-resource countries review and approve trials when a myriad of requests are received?

11:00 a.m. Moderator: **Janice Cooper,** Liberia Mental Health initiative

Panelists:
(*90 min; 10-min opening remarks by each panelist, followed by discussion and Q&A*)

- **Nahid Bhadelia,** Assistant Professor of Medicine, Director of Infection Control, National Emerging Infectious Disease Laboratories (NEIDL), Boston University
- **Peter Kilmarx,** Deputy Director, Fogarty International Center, National Institutes of Health
- **Matthew Barnhart,** Senior Science Advisor, Bureau for Global Health, USAID

12:30 p.m. LUNCH

SESSION III – THE ECONOMIC IMPLICATIONS OF OUTBREAKS

1:00 p.m. Objectives:

- For low-resource countries with fragile economies, explore the full economic impact of outbreaks, including how low-income countries can best cope and how international assistance can be provided for recovery in the short term as well as the long term.
- Discuss how sustainable funding for clinical research during public health outbreaks can be established and what an efficient mechanism for their allocation and use might be. How could the promising therapeutic and vaccine interventions be delivered, and who should pay?
- Is there a reasonable source of sustainable funding for stockpiling interventions for emerging infectious diseases? How would such a fund be managed and by whom? What would be a workable mechanism for decision making about which products to store and when to release them?

Copyright © National Academy of Sciences. All rights reserved.

Moderator: **Gerald Keusch,** *Committee Co-Chair*, Boston University Schools of Medicine and Public Health

Panelists:
(60 min; 10-min opening remarks by each panelist, followed by discussion and Q&A)

- **Ok Pannenborg,** Retired Chief Health Advisor, World Bank
- **Mead Over,** Senior Fellow, Center for Global Development

Copyright © National Academy of Sciences. All rights reserved.

Day 2
Tuesday, June 14, 2016

SESSION IV – CLINICAL TRIALS CONDUCTED DURING THE 2014–2015 OUTBREAK

Objectives:

- Discuss the considerations that were taken into account in the design of the trial (i.e., meeting scientific and ethical standards, health systems infrastructure, time to trial launch, public opinion, need of the affected population, etc.).
- Discuss any alternative trial designs considered leading up to implementation of the trial; explore why particular designs were selected.
- Explore the role of the trialist, if any, in selecting the interventions used in the EVD trials; discuss the considerations that go into advancing experimental compounds into clinical trials.
- Discuss the trial results, where available, and explore the scientific and public health value in the data derived from each study. What, if anything, would you do differently next time to achieve greater gains from trials?

8:30 a.m. Welcome by Committee Co-Chairs

- **Gerald Keusch,** *Committee Co-Chair*, Boston University Schools of Medicine and Public Health
- **Keith McAdam,** *Committee Co-Chair*, London School of Hygiene & Tropical Medicine

8:45 a.m. Opening Presentation:
(*15- to 20-min presentation followed by Q&A*)

Looking Forward: Principles for Conducting Research during Emergencies, Lessons Learned through the Liberia–U.S. Joint Clinical Research Partnership

- **Elizabeth Higgs,** Global Health Science Advisor, Division of Clinical Research, National Institute of Allergy and Infectious Disease (NIAID), National Institutes of Health (NIH)

Copyright © National Academy of Sciences. All rights reserved.

9:15 a.m. Moderator: **Jens Lundgren,** University of Copenhagen

Panel 1: Vaccine Trials Conducted During the Ebola Outbreak
(*90 min; 10-min opening remarks by each trial team followed by discussion and Q&A*)

- PREVAIL I
  - **Jerome F. Pierson,** Chief, Regulatory Compliance & Human Subjects Protection Branch, NIAID, NIH
  - **James Neaton,** Professor of Biostatistics, Adjunct Professor of Medicine, Distinguished International Professor, University of Minnesota
- STRIVE:
  - **Anne Schuchat,** Principal Deputy Director, U.S. Centers for Disease Control and Prevention (CDC)

10:45 a.m. BREAK

11:00 a.m. Moderator: **Roger J. Lewis,** Harbor–UCLA Medical Center

Panel 2: Therapeutic Trials Conducted During the Ebola Outbreak
(*90 min; 10-min opening remarks followed by discussion and Q&A*)

- PREVAIL II:
  - **Richard T Davey,** Senior Investigator, Laboratory of Immunoregulation, NIAID, NIH
  - **John Beigel,** Leidos Biomedical Research, Inc., in support of Clinical Research Section, LIR, NIAID, NIH
  - **Mike Proschan,** Mathematical Statistician, Biostatistics Research Branch, NIAID, NIH
  - **Lori Dodd,** Mathematical Statistician, Biostatistics Research Branch, NIAID, NIH

12:30 p.m. Closing Presentation
(*15- to 20-min presentation followed by Q&A*)

Fostering International Cooperation and Collaboration
- **Gray Handley,** International Office Director at NIAID

1:00 p.m. ADJOURN Public Session Day 2

Copyright © National Academy of Sciences. All rights reserved.

Day 3
Wednesday, June 15, 2016

SESSION V – DECISION MAKING DURING EMERGENCIES

Objectives:

- Discuss the ethical imperatives present during an international humanitarian emergency and the role of local and international health officials, regulatory agencies, and research and clinical staff in determining an ethical course of action.
- Explore how ethical and human rights considerations regarding clinical research can be assessed in the midst of an emerging outbreak.
- Identify the appropriate role of international organizations and national/district-level health, research, and regulatory agencies in decision making.

8:30 a.m. Welcome by Committee Co-Chairs

- **Gerald Keusch,** *Committee Co-Chair*, Boston University Schools of Medicine and Public Health
- **Keith McAdam,** *Committee Co-Chair*, London School of Hygiene & Tropical Medicine

8:45 a.m. Opening Presentation:
(*15- to 20-min presentation followed by Q&A*)

Aligning Regulatory, Public Health, and Clinical Care Goals During an Epidemic Crisis

- **Jesse Goodman,** Professor and Director, Center on Medical Product Access, Safety and Stewardship (COMPASS), Georgetown

9:15 a.m. Moderator: **Charles D. Wells,** Sanofi

Panelists:
(*90 min; 10-min opening remarks by each panelist, followed by discussion and Q&A*)

- **Ross Upshur,** Canada Research Chair in Primary Care Research; Professor, Department of Family and Community Medicine and Dalla Lana School of Public Health, University of Toronto

Copyright © National Academy of Sciences. All rights reserved.

- **Christine Grady,** Chief, Clinical Center's Department of Bioethics, National Institutes of Health Clinical Center
- **John David Pringle,** Postdoctoral Fellow in Humanitarian Health Ethics, McGill University; Vice Chair, MSF Ethics Review Board, 2015

10:45 a.m. BREAK

SESSION VI – MANAGING GROUP DYNAMICS DURING CRISES

11:00 a.m. Objectives:

- Examine how global institutions can respectfully prioritize and align the interests and expertise of organizations (including public health professionals, clinical care providers, and academic/medical research staff) to design and implement a coordinated course of action to achieve the greatest benefit, while respecting the opinions of local institutions and communities.
- Provide examples of best practices used to address, prevent, and overcome disagreements within and between large institutions in order to reach agreeable compromises.

Moderator: **Keith McAdam,** *Committee Co-Chair*, Emeritus Professor of Clinical and Tropical Medicine, London School of Hygiene & Tropical Medicine

Panelists:
(*90 min; 10-min opening remarks by each panelist followed by discussion and Q&A*)

- **David Cooperrider,** Fairmount Santrol–David L. Cooperrider Professor of Appreciative Inquiry at the Weatherhead School of Management, Case Western
- **Howard Gadlin,** Retired Ombudsman and Director of the Center for Cooperative Resolution, NIH

SESSION VII – PUBLIC COMMENT

12:30 p.m. Open Public Comment (*30 min*)

- Members of the public are invited to sign up to provide comments geared toward the session topic.

1:00 p.m. ADJOURN Public Session

Copyright © National Academy of Sciences. All rights reserved.

Meeting 5: Monrovia, Liberia; August 14–17

Public Workshop of the Committee on Clinical Trials During the 2014–2015 Ebola Outbreak

Fourth Committee Meeting
August 15–16, 2016
Bella Casa Hotel
2nd Street Sinkor Tubman Blvd.
Monrovia, Liberia

Day 1
Monday, August 15, 2016

SETTING RESEARCH PRIORITIES DURING EMERGENCY INFECTIOUS DISEASE EVENTS

Day 1 Meeting Objective:

- Explore lessons learned from the 2014–2015 Ebola outbreak to best prioritize, design and implement clinical research during public health emergencies.

8:30 a.m. **Meeting Registration**

9:00 a.m. Welcome by Committee Co-Chairs

- **Gerald Keusch,** *Committee Co-Chair*, Boston University Schools of Medicine and Public Health
- **Keith McAdam,** *Committee Co-Chair*, London School of Hygiene & Tropical Medicine

Welcome and Perspectives from the Ministries of Health

- Discuss the top lessons learned from the Ebola outbreak. How can research best be incorporated into national response efforts in the event of future outbreaks?

Co-Moderators: **Janice Cooper**, Carter Center Liberia
M. Bailor Barrie, Wellbody Alliance

Speakers (*10-min prepared remarks each, followed by Q&A*):

- **Hon. Bernice Dahn,** Minister of Health and Social Welfare, Ministry of Health and Social Welfare of the Republic of Liberia

Copyright © National Academy of Sciences. All rights reserved.

- **Hon. Zulianatu Cooper,** Deputy Minister of Health and Sanitation II, Ministry of Health and Sanitation of the Republic of Sierra Leone

10:00 a.m. BREAK

10:15 a.m. **Panel 1: Prioritizing Research in Outbreak Response**

- Describe national capacity over time to respond to the outbreak. What were the key challenges and lessons learned?
- Discuss the process by which research proposals were prioritized.
- Discuss how the numerous and varied institutional pressures influenced decision-making priorities.
- Consider how to facilitate the incorporation of clinical trials in the public health and care response during future emergency infectious disease events.

Co-Moderators: **Janice Cooper,** Carter Center Liberia
M. Bailor Barrie, Wellbody Alliance

Panelists (*10-min prepared remarks each*):

- **Tolbert Nyenswah,** Legal and Senior Public Health Specialist, Deputy Minister Health for Disease Surveillance and Epidemic Control, Liberia
- **Alie Wurie,** Case Management Lead, National Emergency Response, Ministry of Health and Sanitation, Republic of Sierra Leone
- **Alpha Mahmoud Barry,** Public Health Specialist, Researcher, University of Gammal, Conakry, Guinea

Respondents (*5 min each, reaction to panelists*):

- **Moses Massaquoi,** National Case Manager, Ebola Response, Ministry of Health/IMS; Country Director, Clinton Health Access Initiative (CHAI); Chair, Sub-Regional Consortium on Ebola Virus Vaccine and Therapeutic Trials in Guinea, Liberia, and Sierra Leone
- **Vuyu Kanda Golakai,** Professor, College of Health and Life Sciences, University of Liberia

Moderated Discussion with Committee and Participants

11:45 a.m. LUNCH

Copyright © National Academy of Sciences. All rights reserved.

Integrating Clinical Research into Epidemic Response: The Ebola Experience

12:45 p.m. **Panel 2: Perspectives from the Research and Training Community**

- Discuss lessons learned from the international research partnerships during the Ebola outbreak. How would you apply those lessons to future research collaborations?
- Examine the research capacity that was acquired by the national researchers as a result of the international research partnerships.
- Discuss the process by which research proposals for therapeutic and vaccine candidates were prioritized for clinical trials. How can this process be improved?
- Describe challenges with designing and implementing scientifically and ethically robust vaccine and therapeutic trials during the Ebola outbreak.
- Explore new ideas and innovative approaches for accelerating future clinical trials in emergency contexts; identify pragmatic methods for building community support, speeding data collection, and assessing the safety, efficacy, and effectiveness of therapeutics and vaccines.

Moderator: **Fred Wabwire Mangen,** Makerere University–Uganda

Panelists (*10-min prepared remarks each*):

- **Mandy Kader Konde,** Professor and Chair, Department of Public Health, University of Conakry; Chairman Guinea Ebola Research Commission; Executive Director, Center of Research on Diseases (CEFORPAG) – *Guinea Ring Vaccine*
- **Mohamed Samai,** PI STRIVE Vaccine Study; Acting Provost of College of Medicine and Allied Health Sciences (COMAHS); Deputy Director for Research, Ministry of Health and Sanitation, Freetown, Sierra Leone – *STRIVE Vaccine Trial*
- **Stephen B. Kennedy,** Co-Principal Investigator, PREVAIL & Coordinator, EVD Research, Incident Management System (IMS), Liberia – *PREVAIL Trials*

Respondents (*5 min each, reaction to panelists*):

- **Abdoul Habib Beavogui,** Director, National Center for Training and Research in Rural Health (CNFRSR) "Jean SENECAL" of Maferinyah, Republic of Guinea – *JIKI* (*Favipiravir*)

Copyright © National Academy of Sciences. All rights reserved.

- **Bartholomew Wilson,** Social Mobilization, Communication and Community Engagement (SMC) Lead of the Partnership for Research on Ebola Virus in Liberia – *PREVAIL Trials*

Moderated Discussion with Committee and Participants

2:45 p.m. BREAK

3:00 p.m. **Panel 3: Perspectives from Regulatory Authorities**

- Describe the mandate of your agency and its role in research, development, and procurement of therapeutic and vaccine products.
- Discuss the lessons learned and practical challenges encountered during the Ebola outbreak.
- Identify key capacity-building needs to improve local regulatory capabilities; consider the availability of resources and regulatory protocols to enable the rapid review of investigational medical products.

Moderator: **Susan Ellenberg,** University of Pennsylvania

Panelists (*10-min prepared remarks each*):

- **Beno Yakubu Nyam,** Chief Regulatory Officer, Clinical Trial Unit, Drug Evaluation and Research Directorate, National Agency for Food and Drug Administration and Control (NAFDAC)
- **Wiltshire C. N. Johnson,** Registrar, Pharmacy Board of Sierra Leone
- **David Sumo,** Managing Director, Liberian Medicines Health Products Regulatory Authority (LMHRA)

Respondent (*5 min, reaction to panelists*):

- **Onome Thomas Abiri,** Head of Pharmacovigilance and Clinical Trial Department, Pharmacy Board of Sierra Leone, Ministry of Health and Sanitation

Moderated Discussion with Committee and Participants

4:00 p.m. **Panel 4: Perspectives from the Ethics Review Board (ERB)**

- Describe the procedures for review of research proposals during the Ebola outbreak. Discuss lessons learned,

Copyright © National Academy of Sciences. All rights reserved.

practical challenges encountered, and identify approaches for more efficient reviews in the future.

- Discuss the role of the ERB in helping shape the clinical trial design decisions and in negotiating terms of the trial.
- In the event of a future outbreak, discuss any best practices to achieve community understanding of key trial design components (such as randomization) if they are determined to be required for valid trial results.

Moderator: **Olayemi Omotade,** University of Ibadan

Panelists (*10-min prepared remarks each*):

- **Hector Morgan,** Professor, Department of Microbiology, College of Medicine and Allied Health Sciences, University of Sierra Leone; Director, Research Ethics Committee, Freetown, Sierra Leone
- **Fatorma K. Bolay,** Director, Liberia Institute of Biomedical Research (LIBR); Chairperson, Liberia Institute for Biomedical Research Ethics Committee
- **Nnah Djenab Sylla**, Secretary General, National Ethics Committee on Health Research, Guinea

Respondents (*5 min each, reaction to panelists*):

- **Gloria Mason,** Coordinator, National Research Ethics Board (NREB), Liberia
- **Tumani Corrah,** Director (MRC UK) Africa Research Development, Director Africa Research Excellence Fund; Emeritus Director, MRC Unit, The Gambia

Moderated Discussion with Committee and Participants

5:30 p.m. ADJOURN

6:15 p.m. **Wine Reception and Dinner at the Bella Casa Restaurant "Suave"**

- Hosted by the National Academy of Medicine's Independent Commission for a Global Health Risk Framework
- Remarks by Dr. Oyewale Tomori, President, Nigerian Academy of Science

Copyright © National Academy of Sciences. All rights reserved.

Day 2
Tuesday, August 16, 2016

ENGAGING COMMUNITIES IN RESEARCH DESIGN AND IMPLEMENTATION DURING OUTBREAKS

Day 2 Meeting Objective:

- Explore lessons learned from the 2014–2015 Ebola outbreak to best engage communities in the design and implementation of clinical research during future outbreaks.
- Discuss opportunities for community involvement in planning activities to better prepare and build local research capacity for future epidemics.

8:30 a.m. **Meeting Registration**

9:00 a.m. Welcome by Committee Co-Chairs

- **Gerald Keusch,** *Committee Co-Chair*, Boston University Schools of Medicine and Public Health
- **Keith McAdam,** *Committee Co-Chair*, London School of Hygiene & Tropical Medicine

Panel 5: Community Mobilizers' Perspectives

- Explore challenges and lessons learned during the Ebola outbreak to overcome fear, rumors, and stigma in the community; consider key groups to engage to ensure effective and far-reaching community engagement.
- Identify best practices for community engagement during a future outbreak and explore methods to gauge individual and community comprehension, acceptance, and adherence to key messages, such as those conveyed during the communication of vaccine or therapeutic trials.

Moderator: **Charles Wells**, Sanofi

Panelists (*10-min prepared remarks each*):

- **Reverend John Barclay Sumo,** Director, National Health Promotion Division; Chair, Social Mobilization Pillar, Ministry of Health
- **Mohammad Bailor Jalloh,** Chief Executive Officer, Focus1000

Copyright © National Academy of Sciences. All rights reserved.

- **Alpha Mahmoud Barry,** Public Health Specialist, Researcher, University of Gammal, Conakry, Guinea

Respondents (*5 min each, reaction to panelists*):

- **Musa Sangarie,** Program Manager, BBC Media Action Sierra Leone
- **Luke Bawo,** Coordinator for Health Management Information Systems (HMIS), Research and Monitoring and Evaluation (M&E), National Ministry of Health in Liberia

Moderated Discussion with Committee and Participants

10:30 a.m. BREAK

10:45 a.m. **Panel 6: Patient and Clinician Perspectives**

- Discuss your experiences during the Ebola outbreak; consider the clinical care provided in Ebola treatment units and explore lessons learned to overcome fear, rumors, and stigma in the community.
- Discuss the role of research during the Ebola outbreak and explore how research should be done during a future outbreak, both during the crisis and once the crisis has passed. How can researchers best address survivors' concerns?
- In the event of a future outbreak, examine your community's understanding of and expectations from clinical care and clinical trials.

Moderator: **David Peters,** Johns Hopkins University

Panelists (*10-min prepared remarks each*):

- **Achille Diona Guemou,** Chairman, Ebola Association Network; Physician with Association pour la Réinsertion des Personnes Guéries et Affectées d'Ebola en Guinée (Association for Rehabilitation of Persons Affected and Cured of Ebola in Guinea)
- **Abdul Karim Bah,** Chief Executive Officer, Sierra Leone Association of Ebola Survivors (S.L.A E.S)
- **Patrick Faley,** Survivor's Consultant – PREVAIL Research Program; Former President, National Ebola Survivors Network Liberia

Copyright © National Academy of Sciences. All rights reserved.

Moderated Discussion with Committee and Participants

12:15 p.m. LUNCH

1:15 p.m. **Panel 7: Perspectives from Civil Society**

- Discuss lessons learned and greatest challenges during the Ebola outbreak, and explore the engagement of civil society in the Ebola clinical trials.
- In the event of a future outbreak, discuss how civil society can best be involved in outbreak response and clinical research.

Moderator: **Abdel G. Babiker,** Medical Research Council Clinical Trials Unit, UCL

Panelists (*10-min prepared remarks each*):

- **Ambassador Juli Endee,** Culture Ambassador of the Republic of Liberia, traditional Queen, UNICEF Goodwill Ambassador for Children in Liberia and Executive Director of the Liberia Crusaders for Peace
- **Shiekh Ahmad Tejan Sillah,** United Nations Goodwill Ambassador, Chief Imam of the Freetown Central Mosque, Founding Member of the Inter-Religious Council of Sierra Leone
- **Abdoulaye Touré,** Associate Professor of Epidemiology, Conakry University
- **Chief Zanzan Kawa,** Chairman of the Council of Chiefs, Liberia

Moderated Discussion with Committee and Participants

2:30 p.m. BREAK

2:45 p.m. **Breakout Groups with Facilitated Discussion**

- Further explore strategies to engage communities in advance of and during outbreaks so that future research is designed to meet the communities' needs.

3:45 p.m. **Reconvene in Plenary Session**

- Recap breakout group discussions.

Copyright © National Academy of Sciences. All rights reserved.

4:15 p.m. **Panel 8: Building Local Research Capacity to Meet Community Needs**

- Explore planning activities during the inter-epidemic period to better prepare for and improve the execution of clinical trials during future infectious disease public health emergencies.
- Identify collaborative opportunities to achieve long-term ethical and scientific gains from clinical trials conducted during emerging infectious disease events.

Panelists and Group Leads (*10 minutes prepared remarks followed by breakout groups with facilitated discussion*):

Moderator: **Roger Lewis,** Harbor–UCLA Medical Center

- **Oyewale Tomori,** President, Nigerian Academy of Science
- **Mosoka Fallah,** Ebola Emergency-Response Program Manager, Action Contre la Faim (ACF) – Liberia
- **Tumani Corrah,** Director (MRC UK) Africa Research Development, Director Africa Research Excellence Fund; Emeritus Director, MRC Unit, The Gambia

Moderated Discussion with Committee and Participants

5:15 p.m. **Open Comment Period and Workshop Wrap-Up**

- Members of the public are invited provide comments geared toward the topics covered in the panel discussions over the course of the 2 days.

5:30 p.m. ADJOURN

Copyright © National Academy of Sciences. All rights reserved.

Day 3
Wednesday, August 17, 2016

LIBERIA SITE VISITS

1:00 p.m. Committee Liberia site visits

- University of Liberia, A.M. Dogliotti College of Medicine
- John F. Kennedy Medical Center
- ELWA-2 (Eternal Love Winning Africa) Ebola Treatment Unit
- Liberian Institute for Biomedical Research (LIBR)

Copyright © National Academy of Sciences. All rights reserved.

Copyright © National Academy of Sciences. All rights reserved.

Appendix B

Clinical Trial Designs

TABLE B-1 Brief Summary of Some Advantages and Disadvantages of Various Clinical Trial Designs

Design	Structure	Advantages	Disadvantages
Traditional RCT<br><br>(Evans, 2010; Glasziou et al., 2007; Suresh, 2011)	• A group of subjects with the target disease is identified and randomized to two or more treatments (e.g., active treatment versus placebo).<br>• A randomized participant receives only one treatment (or treatment strategy) during the duration of the trial.<br>• Participants are then followed over time and the responses are compared between groups.	• Allows for valid treatment group comparisons.<br>• Provides an estimate of effect that is unbiased and consistent.	• Can require large sample sizes due to the existence of both within- and between-subject variation.<br>• Sample sizes can also be large when the desired effect size to detect is small.<br>• Can be expensive, lengthy.

continued

Copyright © National Academy of Sciences. All rights reserved.

TABLE B-1 Continued

Design	Structure	Advantages	Disadvantages
Cluster Randomized Trials<br><br>(Campbell et al., 2004; Donner and Klar, 2004; Edwards et al., 1999)	• Intact groups of individuals are randomized to receive different interventions.	• The ability to study interventions that cannot be directed toward selected individuals.<br>• Avoids treatment group contamination.<br>• Enhances subject compliance.	• More complex to design.<br>• Requires more participants to obtain equivalent statistical power.<br>• Requires more complex analysis.<br>• Observations on individuals in the same cluster tend to be correlated (non-independent), and so the effective sample size is less than the total number of individual participants.<br>• After randomization, individuals in the clusters may be approached for consent, which raises the possibility of post-randomization selection bias, or they may not, which raises ethical concerns.

Copyright © National Academy of Sciences. All rights reserved.

TABLE B-1 Continued

Design	Structure	Advantages	Disadvantages
Stepped Wedge<br><br>(Brown and Lilford, 2006; Hughes, 2007)	• Sequential roll-out of an intervention to participants (individuals or clusters) over a number of time periods.<br>• The order in which the different individuals or clusters receive the intervention is determined at random, and, by the end of the random allocation, all individuals or groups will have received the intervention.<br>• Stepped-wedge designs incorporate data collection at each point where a new group (step) receives the intervention.	• Particularly useful when it is not feasible to provide the intervention to everyone or every community at once.<br>• For evaluating the effectiveness of interventions that have been shown to be efficacious in a more limited, research setting and are now being scaled up to the community level.<br>• This design is also useful for evaluating temporal changes in the intervention effect.<br>• Two key (non-exclusive) situations in which a stepped-wedge design is considered advantageous are:<br>1. If there is a prior belief that the intervention will do more good than harm, rather than a prior belief of equipoise, it may be unethical to withhold the intervention from a proportion of the participants or to withdraw the intervention as would occur in a cross-over design.	• Likely to lead to a longer trial duration than a traditional parallel design, particularly where effectiveness is measured immediately after implementation.<br>• Imposes some practical implementation challenges, such as preventing contamination between intervention participants and those waiting for the intervention and ensuring that those assessing outcomes are blind to the participants' statuses as intervention or control in order to help guard against information bias.

continued

Copyright © National Academy of Sciences. All rights reserved.

TABLE B-1 Continued

Design	Structure	Advantages	Disadvantages
		2. There may be logistical, practical, or financial constraints that mean the intervention can only be implemented in stages.	

Copyright © National Academy of Sciences. All rights reserved.

TABLE B-1 Continued

Design	Structure	Advantages	Disadvantages
Multiarm, Multistage Trial with a Common Control<br><br>(Jaki, 2015; Wason et al., 2016)	• Consist of simultaneously testing several experimental treatments against a common control.<br>• Interim analyses are used in order to decide which treatments should continue.	• Advantages over running separate controlled trials for each experimental treatment are<br>1. A shared control group can be used, instead of a separate control group for each treatment;<br>2. A direct head-to-head comparison of treatments is conducted, minimizing biases that can be introduced from making comparisons between treatments tested in separate trials;<br>3. The use of interim analyses allows ineffective treatments to be dropped early or allows an early stopping of the trial if one treatment is clearly superior (although this advantage applies also in the case of separate trials of each treatment through use of group-sequential designs).	• Different trials comparing a single treatment against control are often initiated and conducted by different centers. As a result, they have different inclusion and exclusion criteria and may use different primary and secondary endpoints and possibly a different comparator treatment. All of these must be standardized for a multiarm trial that requires negotiations and compromises between investigators.<br>• Need to ensure that no bias in the evaluation is introduced in multi-center multiarm studies through imbalances between allocations to treatments at different centers/regions. It is therefore paramount that randomization to all arms (including the control arm) is stratified by center or region to ensure that the risk of bias is minimized.<br>• Using standard analysis methods for this purpose will result in an overly enthusiastic (upward-biased) estimate of the effect. Specialized methods that lead to unbiased estimators or reduce the bias are therefore necessary.

continued

Copyright © National Academy of Sciences. All rights reserved.

TABLE B-1 Continued

Design	Structure	Advantages	Disadvantages
Delayed Start<br><br>(D'Agostino 2009; Velengtas et al., 2012)	• One group receives active treatment and another group receives placebo during the first period of the trial.<br>• Both groups receive active treatment during the second period of the trial.	• Delayed-start study design separates the disease-modifying effects of administered treatment from short-term beneficial effects on symptoms.<br>• The study design also addresses ethical concerns raised with respect to RCTs. More patients receive the active intervention as than in a traditional trial. All participants eventually receive the potentially beneficial medical intervention, while a control group is maintained in the initial phase.	• Delayed-start design requires sufficient understanding of the study design and clinical progression of the disease to define adequate Phase I and Phase II durations and of the statistical methodology to address analytical considerations.<br>• Only the first half of the study is considered double blind; the second half is open label, a limitation that may introduce bias through unblinding.<br>• The delayed-start design study may encounter enrollment issues; it needs to recruit patients who are willing to be off the symptomatic therapy for the first half of the study if they are randomized to the control arm.<br>• Only patients with mild, early, and more slowly progressive disease may be eligible for this type of study.<br>• The studies are susceptible to high dropout rates and patient discontinuation in the Phase I placebo group because these patients do not experience any treatment effects. Differential baseline characteristics between patients in Phase II and discontinued patients may introduce confounding, and compromise results.

Copyright © National Academy of Sciences. All rights reserved.

TABLE B-1 Continued

Design	Structure	Advantages	Disadvantages
Adaptive Platform<br><br>(Quinlan et al., 2010; Saville and Berry, 2016)	• A clinical trial with a single master protocol in which multiple treatments are evaluated simultaneously.<br>• Adaptive platform designs offer flexible features such as dropping treatments for futility, declaring one or more treatments superior, or adding new treatments to be tested during the course of a trial.	• Provides the flexibility to redesign clinical trials at interim stage.<br>• Enables faster, cheaper drug development by enabling real-time learning and terminating a trial or treatment arms at the earliest time point, enabling the choice of the correct dose(s) for Phase III, and by enabling the selection of the population responding best to treatment.	• Requires more work and additional effort during planning, implementation, execution, and reporting.<br>• Barriers to implementation include<br>o Technical concerns<br>o Perceptions of regulatory risk<br>o Challenges related to change management

continued

Copyright © National Academy of Sciences. All rights reserved.

TABLE B-1 Continued

Design	Structure	Advantages	Disadvantages
Single Arm with Comparisons to Historical Controls<br><br>(Evans, 2010)	• A sample of individuals is given experimental therapy and followed over time.<br>• Design may be desirable when the patient pool is limited.<br>• Used to obtain preliminary efficacy evidence (not confirmatory).<br>• Best used when the natural history of the disease is well understood, when placebo effects are minimal or nonexistent, and when a placebo control is not ethically desirable.	• May be the only (or one of few) options for trials evaluating therapies for which placebos are not ethical and options for controlled trials are limited.	• There is an inability to distinguish between the effect of the treatment, a placebo, and the effect of natural history.<br>• It is difficult to interpret the response without a frame of reference for comparison.

Copyright © National Academy of Sciences. All rights reserved.

TABLE B-1 Continued

Design	Structure	Advantages	Disadvantages
Uncontrolled Case Series<br><br>(Ford, 2010; Kempen, 2011)	A group or series of case reports involving patients who were given similar treatment. Reports of case series usually contain detailed information about the individual patients before and after an intervention but with no control group.<br>• Should have clear definitions of the phenomena being studied.<br>• These same definitions should be applied equally to all individuals in the series.<br>• All observations should be reliable and reproducible (consider blinding).	• Informs patients and physicians about natural history and prognostic factors.<br>• Easy and inexpensive to do in hospital settings.<br>• Helpful in hypothesis formation.<br><br>Some appropriate settings for the use of the case series study design:<br>• Proof (or disproof) of concept for a new hypothesis<br>• Reporting of sentinel events<br>o Toxicities of therapies<br>o Recognition of epidemics<br>o Initial identification of previously unrecognized syndromes<br>• Studying outcomes of rare diseases or new treatments (limited usefulness)	• Cases may not be representative.<br>• Outcome may be a chance finding, not characteristic of disease.<br>• Cannot easily examine disease etiology.<br>• Exposure reflects the underlying population, not the outcome.<br>• Begs the question "Compared to what?"

Copyright © National Academy of Sciences. All rights reserved.

Integrating Clinical Research into Epidemic Response: The Ebola Experience

REFERENCES

Brown, C. A., and R. J. Lilford. 2006. The stepped wedge trial design: A systematic review. *BMC Medical Research Methodology* 6(1):54.

Campbell, M. K., D. R. Elbourne, and D. G. Altman. 2004. CONSORT statement: Extension to cluster randomised trials. *BMJ* 328(7441):702–708.

D'Agostino, R. B. 2009. The delayed-start study design. *New England Journal of Medicine* 361(13):1304–1306.

Donner, A., and N. Klar. 2004. Pitfalls of and controversies in cluster randomization trials. *American Journal of Public Health* 94(3):416–422.

Edwards, S. J. L., D. A. Braunholtz, R. J. Lilford, and A. J. Stevens. 1999. Ethical issues in the design and conduct of cluster randomised controlled trials. *BMJ* 318(7195):1407–1409.

Evans, S. R. 2010. Clinical trial structures. *Journal of Experimental Stroke & Translational Medicine* 3(1):8–18.

Ford, D. E. 2010. *Study design case series and cross-sectional.* Johns Hopkins Institute for Clinical and Translational Research. http://ictr.johnshopkins.edu/wp-content/uploads/import/1274-Ford%20Final%20Rev%20Cross%20Sectional%20July%2012%202011.pdf (accessed January 25, 2017).

Glasziou, P., I. Chalmers, M. Rawlins, and P. McCulloch. 2007. When are randomised trials unnecessary? Picking signal from noise. *BMJ* 334(7589):349–351.

Hughes, J. P. 2007. Stepped wedge design. In *Wiley Encyclopedia of Clinical Trials: 1–8*. Hoboken, NJ: John Wiley & Sons, Inc.

Jaki, T. 2015. Multi-arm clinical trials with treatment selection: What can be gained and at what price? *Clinical Investigation* 5(4):393–399.

Kempen, J. H. 2011. Appropriate use and reporting of uncontrolled case series in the medical literature. *American Journal of Ophthalmology* 151(1):7–10.e11.

Quinlan, J., B. Gaydos, J. Maca, and M. Krams. 2010. Barriers and opportunities for implementation of adaptive designs in pharmaceutical product development. *Clinical Trials* 7(2):167–173.

Saville, B. R., and S. M. Berry. 2016. Efficiencies of platform clinical trials: A vision of the future. *Clinical Trials* 13(3):358–366.

Suresh, K. P. 2011. An overview of randomization techniques: An unbiased assessment of outcome in clinical research. *Journal of Human Reproductive Sciences* 4(1):8–11.

Velengtas, P., P. Mohr, and D. A. Messner. 2012. *Making informed decisions: Assessing the strengths and weaknesses of study designs and analytic methods for comparative effectiveness research*. Washington, DC: National Pharmaceutical Council. http://www.npcnow.org/publication/making-informed-decisions-assessing-strengths-and-weaknesses-study-designs-and-analytic (accessed January 25, 2017).

Wason, J., D. Magirr, M. Law, and T. Jaki. 2016. Some recommendations for multi-arm multistage trials. *Statistical Methods in Medical Research* 25(2):716–727.

Copyright © National Academy of Sciences. All rights reserved.

Appendix C

Ethical Principles for Research with Human Subjects

TABLE C-1 Basic Ethical Principles That Should Govern Research with Human Subjects

Seven Key Requirements Used to Review Trials Conducted During the 2014–2015 Ebola Outbreak	Source Guidance Documents	Key Principles
1. *Scientific and Social Value*	CIOMS Ethical Guidelines, 2016<br>Nuremburg Code, 1947<br>WMA Declaration of Helsinki, 2013<br>HHS Common Rule, 2009<br>Convention on Human Rights and Biomedicine, 1997	• The knowledge to be gained through the conduct of the research must be of direct or incremental value to the development of clinical or public health advancements.<br>• The value of the research must be such that it (1) justifies any direct or indirect risks and burdens to participants and their communities and (2) justifies the allocation of resources away from other emergency response activities.

continued

Copyright © National Academy of Sciences. All rights reserved.

TABLE C-1 Continued

Seven Key Requirements Used to Review Trials Conducted During the 2014–2015 Ebola Outbreak	Source Guidance Documents	Key Principles
2. *Respect for Persons*	CIOMS Ethical Guidelines, 2016<br>UNESCO Declaration, 2005<br>Belmont Report, 1979<br>Nuremburg Code, 1947<br>WMA Declaration of Helsinki, 2013<br>HHS Common Rule, 2009<br>Convention on Human Rights and Biomedicine, 1997	• Research must honor the rights and welfare of participants by (1) providing prospective participants with clear and accessible information on the possible benefits and risks to participation and the research purpose; and (2) obtaining voluntary consent and ensuring that participants understand that they are able to withdraw consent at will and without reprisal.
3. *Community Engagement*	CIOMS Ethical Guidelines, 2016	• Research activities must by centered on an ongoing commitment to sustaining community engagement focusing on communication about the research purpose, design, and possible risks and benefits at the individual and societal level and elicitation of community concerns and preferences.
4. *Concern for Participant Welfare and Interests*	CIOMS Ethical Guidelines, 2016<br>UNESCO Declaration, 2005<br>Belmont Report, 1979<br>Nuremburg Code, 1947<br>WMA Declaration of Helsinki, 2013<br>HHS Common Rule, 2009	• Gratuitous risks to participants cannot be justified, and protections must be taken to limit violation of privacy and potential stigma associated with participation.<br>• Efforts must be made to increase benefits to participants to the extent possible, including access to interventions that are found to be efficacious.

Copyright © National Academy of Sciences. All rights reserved.

TABLE C-1 Continued

Seven Key Requirements Used to Review Trials Conducted During the 2014–2015 Ebola Outbreak	Source Guidance Documents	Key Principles
5. *Favorable Risk–Benefit Ratio*	CIOMS Ethical Guidelines, 2016<br>UNESCO Declaration, 2005<br>Belmont Report, 1979<br>WMA Declaration of Helsinki, 2013<br>HHS Common Rule, 2009<br>Convention on Human Rights and Biomedicine, 1997	• The expected knowledge to be gained by the research must be justified in relation to the expected benefits and burdens associated with participation.
6. *Justice in the Distribution of Benefits and Burdens*	CIOMS Ethical Guidelines, 2016<br>Belmont Report, 1979<br>HHS Common Rule, 2009	• The research must not focus inequitably on the health needs of a specific group, and, relatedly, a specific group should not disproportionately bear the burden and risks associated with the research.
7. *Post-Trial Access*	CIOMS Ethical Guidelines, 2016, UNESCO Declaration, 2005<br>WMA Declaration of Helsinki, 2013	• There is an obligation to provide the communities that supported research with access to post-trial investigational products.

Copyright © National Academy of Sciences. All rights reserved.

REFERENCES

CIOMS (Council for International Organizations of Medical Sciences). 2016. *International ethical guidelines for biomedical research involving human subjects*. http://www.cioms.ch/ethical-guidelines-2016 (accessed January 20, 2017).

COE (Council of Europe). 1997. Convention for the protection of human rights and dignity of the human being with regard to the application of biology and medicine: Convention on Human Rights and Biomedicine. *Kennedy Institute of Ethics Journal* 7(3):277–290.

HHS (U.S. Department of Health and Human Services). 2009. *45 CFR 46, Common Rule for the Protection of Human Subjects in Research*.

National Commission for the Protection of Human Subjects of Biomedical and Behavioral Research. 1979. *The Belmont report: Ethical principles and guidelines for the protection of human subjects of research*. DHEW Publication No. (OS) 78-0013 and No. (OS) 78-0014. Washington, DC: U.S. Government Printing Office.

Nuremberg Code (1947). 1996. *BMJ* 313(7070):1448.

UNESCO (United Nations Educational, Scientific and Cultural Organization). 2006. *Universal declaration on bioethics and human rights*. Paris, France: UNESCO. http://unesdoc.unesco.org/images/0014/001461/146180E.pdf (accessed January 25, 2017).

WMA (World Medical Association). 2013. World Medical Association Declaration of Helsinki: Ethical principles for medical research involving human subjects. *JAMA* 310(20):2191–2194.

Copyright © National Academy of Sciences. All rights reserved.

Appendix D

Biographical Sketches of Committee Members and Staff

COMMITTEE

Gerald T. Keusch, M.D., FRCP (*Co-Chair*), is a graduate of Columbia College and Harvard Medical School. He has been involved in academic medicine for his entire career, currently as a professor of medicine and global health at Boston University, where he serves as an associate director of the National Emerging Infectious Diseases Laboratory. Prior to this he was the chief of the Division of Geographic Medicine and Infectious Diseases at Tufts Medical Center in Boston from 1979 to 1998 and the associate director for international research and the director of the Fogarty International Center at the U.S. National Institutes of Health from 1998 to 2004. Dr. Keusch is a fellow of the Infectious Diseases Society of America and an elected member of the American Society for Clinical Investigation, the Association of American Physicians, and the National Academy of Medicine, where he has served on the Board on Global Health and the Forum on Microbial Threats and co-chaired an Institute of Medicine/National Research Council report, *Sustaining Global Surveillance and Response to Emerging Zoonotic Diseases*, released in September 2009. He has experience in both laboratory and clinical field research on infectious diseases in Latin America, Africa, and Asia. He has been a member of multiple committees for the Tropical Diseases Research Program at the World Health Organization and the Wellcome Trust, including a recent review committee for the Wellcome Trust on Global Clinical Trials.

Copyright © National Academy of Sciences. All rights reserved.

Keith McAdam, DL, MB BCh, FRCP, FWACP (*Co-Chair*), is the founding director of the Infectious Diseases Institute (2004–2007) at Makerere University in Kampala, Uganda. He is an emeritus professor of clinical tropical medicine at the London School of Hygiene & Tropical Medicine, where he was a professor of clinical tropical medicine from 1985 to 2004. From 1994 to 2003 Dr. McAdam was seconded to West Africa to serve as director of the UK Medical Research Council Laboratories in the Republic of The Gambia. Dr. McAdam grew up in Uganda, where his father, Sir Ian McAdam, was a professor of surgery at Makerere. He did his schooling in Kenya and went on to study medicine at Cambridge University and the Middlesex Hospital in London. After training in internal medicine in London, he spent 3 years at the Institute of Medical Research in Papua New Guinea, working on leprosy, malaria, and filariasis as causes of secondary amyloidosis. For 2 years, from 1975 to 1977, Dr. McAdam developed his laboratory and clinical research focus on inflammation, acute phase proteins, and cytokines at the Immunology Branch of the National Cancer Institute in Bethesda, Maryland, and he continued this focus over the next 7 years in Boston as a clinical scientist in the Department of Medicine at Tufts New England Medical Center. Dr. McAdam was medical advisor to the UK Parliamentary Select Committee on AIDS in 1987 and a member of the Nuffield Council on Bioethics working party that produced an authoritative publication, *The Ethics of Healthcare Related Research in Developing Countries*. He has been associate international director at the Royal College of Physicians in London and is currently its special advisor on East Central and Southern Africa. He has just rotated off the International Board of the African Medical and Research Foundation and is currently on the board of trustees of the charity BBC Media Action.

Abdel Babiker, Ph.D., received his doctoral degree in mathematical analysis from the University of London. In the 1980s he worked on a number of cancer studies at the Institute of Cancer Research and with the Imperial Cancer Research Fund. He joined the MRC HIV Clinical Trials Centre (HIV CTC) as deputy head in 1992 and was directly responsible for overseeing all statistical aspects of the center's research program. When the HIV CTC became part of the MRC Clinical Trials Unit in 1998, he was appointed head of the HIV Group. HIV research has expanded greatly since 1998, through wider national and international collaborations addressing key questions in treatment and prevention of HIV, and has affected international guidelines for the treatment of HIV. Dr Babiker is a member of the executive committee of the International Network for Strategic Initiatives in Global HIV Trials (INSIGHT) and co-chair of the START study. He is a fellow of the Royal Statistical Society and the World Academy of Sciences for the advancement of science in the developing world and has served as

Copyright © National Academy of Sciences. All rights reserved.

associate editor for *Sexually Transmitted Infections* and *Controlled Clinical Trials*.

Mohamed Bailor Barrie, M.B.Ch.B., grew up in poverty in rural Sierra Leone. After finishing secondary school, he received one of two scholarships in the country to study medicine at the College of Medicine and Allied Health Sciences in Freetown, Sierra Leone. Dr. Barrie trained in general medicine, building his skills in all areas of medicine. The civil war in Sierra Leone forced him to suspend his studies, and, after 1 year as a refugee in neighboring Guinea, he graduated with his degree in medicine in 2004. After obtaining his degree Dr. Barrie worked as a medical officer at a rural nonprofit hospital and was one of four physicians in his graduating class to continue to practice medicine in Sierra Leone. He has also acted as a consultant for UNICEF and the World Health Organization. In 2006 Dr. Barrie co-founded and became the executive and medical director of Wellbody Alliance, a nonprofit health care organization based in Kono District, Sierra Leone. His current role there is as chief strategic officer. Dr. Barrie is the recipient of the 2013 Grace Humanitarian Award from Thomas Jefferson University. For 2013–2015, Dr. Barrie was awarded a Fulbright Fellowship to study global health delivery at Harvard University.

Janice Cooper, Ph.D., M.P.A., is the country lead for the Liberia Mental Health Initiative. She oversees a national training, policy, and support program to expand capacity for mental health services delivery. She also is responsible for interacting with national and international colleagues and partners of the program. During the Ebola outbreak in Liberia she led the psychosocial pillar for the Incident Management System, the national Ebola virus disease response system. A native Liberian and health services researcher specializing in children's mental health, Dr. Cooper has worked in the private, public, and nonprofit sectors in the United States and Liberia. Prior to joining The Carter Center in 2010, Dr. Cooper was the interim director of the National Center for Children in Poverty as well as an assistant clinical professor in health policy and management at Columbia University's Mailman School of Public Health. From 2005 to 2009, she also served as the center's director of child health and mental health, receiving the distinguished Calderone Prize for Junior Faculty in 2007. Dr. Cooper received her Ph.D. in health policy from Harvard University. She was a 2001 fellow in medical ethics at Harvard Medical School and a 1999 Archibald Bush Foundation Leadership Fellow. She holds additional undergraduate and graduate degrees from the University of Essex in Colchester, England, and Columbia and Harvard Universities in the United States. In 2016 she received the Beyond Health Award from Boston University.

Copyright © National Academy of Sciences. All rights reserved.

Sheila Davis, D.N.P., ANP-BC, FAAN, is the chief of Ebola response and the chief nursing officer at Partners In Health (PIH), for which she led the Ebola response efforts in Sierra Leone and Liberia. At peak of the effort, PIH was operating in collaboration with the ministries of health at over 20 facilities for the screening and treatment of Ebola. Currently she is leading the effort to transition from Ebola response to health system strengthening in Liberia and Sierra Leone as part of PIH's long-term commitment to both countries. Dr. Davis has been a nursing leader in the field of HIV/AIDS since its emergence in the mid-1980s, and she served on the national board of the Association of Nurses AIDS Care (ANAC). She entered the global health arena in 1999 when she began working for Partners AIDS Research Center as part of Massachusetts General Hospital on community outreach and HIV treatment efforts. Partnering with global nursing colleagues, she co-founded a small nongovernmental organization that worked in South Africa and Boston from 2004 to 2010 on health projects including a rural village nurse clinic and an urban vulnerable-children feeding program.

Dr. Davis received her B.S.N. from Northeastern University in 1988, her masters in nursing as an adult nurse practitioner from the MGH Institute of Health Professions in 1997, and her doctorate in nursing practice with a concentration in global health in 2008 also from the MGH Institute of Health Professions. She was a faculty member at the School of Nursing at the MGH Institute of Health Professions for 4 years and an adult nurse practitioner at MGH Infectious Diseases outpatient practice for over 15 years. She is currently adjunct faculty at the University of California, San Francisco, School of Nursing.

Inducted as a fellow of the American Academy of Nursing in 2008, Dr. Davis is a frequent national speaker on global health, clinical topics including Ebola and HIV/AIDS, and the role of nursing in human rights. In 2009 she was inducted as one of the inaugural class of 12 Carl Wilken's Fellows working on antigenocide global efforts as part of the Genocide Intervention Network. Dr. Davis has published in a number of domestic and global journals and is on the editorial board of *Health and Human Rights: An International Journal*. She was part of the 2012 cohort of the Robert Wood Johnson Executive Nurse Fellowship, a 3-year fellowship that prepares 20 national nursing leaders to contribute to the national health care strategy.

Kathryn Edwards, M.D., is the Sarah H. Sell and Cornelius Vanderbilt Professor of Pediatrics at the Vanderbilt University School of Medicine. She graduated from the University of Iowa College of Medicine and completed her pediatric residency and infectious disease fellowship at Northwestern University and her postdoctoral training in immunology at Rush Medical

Copyright © National Academy of Sciences. All rights reserved.

School in Chicago. Dr. Edwards joined the Vanderbilt Vaccine Program in 1980. She has had an extensive experience in leading National Institutes of Health (NIH)-funded and Centers for Disease Control and Prevention (CDC)-funded multicenter investigations and in conducting pivotal Phase I, II, and III clinical trials on vaccines and therapeutics. In 1998 Dr. Edwards was awarded a contract from the CDC to conduct active population-based surveillance to monitor the impact of newly licensed vaccines, which evolved into the existing New Vaccine Surveillance Network. She has also led the CDC-funded Center for Immunization Safety Assessment (CISA) to monitor the safety of vaccines. In 2012 Dr. Edwards conducted comprehensive pneumonia surveillance studies in children and adults.

Dr. Edwards has served on many CDC, NIH, World Health Organization, and Infectious Diseases Society of America (IDSA) committees. She received the IDSA Mentor Award in 2006, the Distinguished Physician Award from the Pediatric Infectious Diseases Society in 2011, the Maureen Andrew Mentoring Award from the Society for Pediatric Research in 2014, and the Charles Mérieux Award in Vaccinology from the National Foundation for Infectious Diseases in 2016. In 2008 she was elected to the National Academy of Medicine.

Susan Ellenberg, Ph.D., joined the biostatistics faculty at the University of Pennsylvania as a professor of biostatistics in the fall of 2004. She also has a secondary appointment in the Department of Medical Ethics and Health Policy. Dr. Ellenberg directs the Biostatistics Core for the Penn Center for AIDS Research and is also collaborating on projects in pulmonary research, breast cancer, anesthesiology, endocrinology, and HIV. Prior to arriving at Penn, Dr. Ellenberg held leadership positions at the U.S. National Institutes of Health and the U.S. Food and Drug Administration. Her areas of research have included surrogate endpoints for treatment effects in clinical trials, operational issues for data monitoring committees, clinical trial designs, adverse event monitoring, vaccine safety, and special issues in cancer and AIDS trials. Dr. Ellenberg is a fellow of the American Statistical Association, the Society for Clinical Trials, and the American Association for the Advancement of Science and is an elected member of the International Statistical Institute. She has served as the president of the Eastern North American Region of the International Biometric Society and of the Society for Clinical Trials and also as the chair of the board of trustees of the National Institute of Statistical Sciences. She is an associate editor of *Clinical Trials* and the *Journal of the National Cancer Institute*. Her book *Data Monitoring Committees in Clinical Trials: A Practical Perspective*, co-authored with Drs. Thomas Fleming and David DeMets, was named Wiley Europe Statistics Book of the Year for 2002.

Copyright © National Academy of Sciences. All rights reserved.

Integrating Clinical Research into Epidemic Response: The Ebola Experience

Roger Lewis, M.D., Ph.D., received a doctorate in biophysics and a medical degree from Stanford University. He is a professor at the David Geffen School of Medicine at the University of California, Los Angeles (UCLA), and the chair of the Department of Emergency Medicine at Harbor–UCLA Medical Center. Dr. Lewis's expertise centers on adaptive and Bayesian clinical trials, including platform trials; translational, clinical, health services, and outcomes research; interim data analysis; data monitoring committees; and informed consent in emergency research studies.

In 2009 Dr. Lewis was elected to membership in the National Academy of Medicine. He is a past president of the Society for Academic Emergency Medicine, a current member of the board of directors for the Society for Clinical Trials, and the senior medical scientist at Berry Consultants, LLC, a group that specializes in adaptive clinical trials.

Dr. Lewis has served as a grant reviewer for the Agency for Healthcare Research and Quality, the Canadian Institutes of Health Research, the U.S. Centers for Disease Control and Prevention, the National Cancer Institute of France, the U.S. National Institutes of Health, the Patient-Centered Outcomes Research Institute, and foundations. He is also a member of the Medicare Evidence Development & Coverage Advisory Committee of the Centers for Medicare & Medicaid Services. Dr. Lewis serves as the chair of data and safety monitoring boards for both federally funded and industry-sponsored clinical trials, including international trials. He is a research methodology reviewer for *JAMA* and an editor of the *JAMA* series titled "JAMA Guides to Statistics and Methods." He has served as a content reviewer for many other peer-reviewed journals. He has authored or co-authored more than 200 original research publications, reviews, editorials, and chapters.

Alex John London, Ph.D., is a professor of philosophy and the director of the Center for Ethics and Policy at Carnegie Mellon University. Professor London is an elected fellow of the Hastings Center and a recipient of the Distinguished Service Award from the American Society of Bioethics and Humanities.

Dr. London's research focuses on foundational ethical issues in human-subjects research, issues of social justice in the transnational context, and on methodological issues in theoretical and applied ethics. His papers have appeared in *Mind*, *Science*, *The Lancet*, *PLoS Medicine*, *Statistics in Medicine*, *The Hastings Center Report*, and numerous other journals and collections. He is co-editor of *Ethical Issues in Modern Medicine*, one of the most widely used textbooks in medical ethics.

In 2012 he joined the working group on the revision of the Council for International Organizations of Medical Sciences 2002 International Ethical Guidelines for Biomedical Research Involving Human Subjects, and in 2011

Copyright © National Academy of Sciences. All rights reserved.

he was appointed to the steering committee on forensic science programs for the International Commission on Missing Persons. Since 2007 he has served as a member of the ethics working group of the HIV Prevention Trials Network. He has testified before the Presidential Commission for the Study of Bioethical Issues and has been commissioned to write papers for the Centers for Disease Control and Prevention and the Institute of Medicine. He has served as an ethics expert in consultations with numerous national and international organizations including the U.S. National Institutes of Health, the World Health Organization, the World Medical Association, and the World Bank.

Jens Lundgren, M.D., D.M.Sc., is a professor of infectious diseases and a practicing infectious disease specialist. He founded and directs the Centre of Excellence for Health, Immunity and Infections at the Department of Infectious Diseases, based at the Copenhagen University Hospital (Rigshospitalet), University of Copenhagen, where he also directs the Centre of Excellence for Personalized Medicine of Infectious Complications in Immune Deficiency, serves as a member of the executive committee of the NIH/NIAID-funded International Network for Strategic Initiatives in Global HIV Trials (INSIGHT) network, chairs its scientific steering committee, and is co-principal investigator for the START study. He is co-editor-in-chief of the *HIV Medicine* journal and was awarded the European AIDS Clinical Society Award for Excellence in HIV Medicine in 2015. He is a member of the American Society of Clinical Investigation and the Association of American Physicians. His list of publications over the past three decades in the scientific literature is extensive, and he has mentored several younger colleagues in their research development.

Michelle M. Mello, J.D., M.P.H., is a professor of health research and policy at Stanford University School of Medicine and a professor of law at Stanford Law School. She holds doctoral degrees in law and health policy and conducts research on issues at the intersection of health policy, law, and bioethics. Ms. Mello's scholarship includes work on ethical issues arising in industry-sponsored clinical trials, legal and ethical barriers to clinical trial data sharing, legal concerns as a hindrance to clinical volunteerism during the Ebola epidemic, and a range of legal and ethical issues in pharmaceutical regulation and human-subjects research. For 7 years she served as the chair of the institutional review board at the Harvard School of Public Health, which is responsible for oversight of numerous clinical trials in African countries.

Olayemi Omotade, M.B.B.S., M.A., FMCPaed, FRCPCH, is a professor of pediatrics and child health at the Institute of Child Health, College of Medicine, University of Ibadan. He is a consultant pediatrician to the Uni-

Copyright © National Academy of Sciences. All rights reserved.

versity College Hospital, Ibadan, Nigeria. As part of his residency training in pediatrics at the University College Hospital, he was on attachment to the University Hospital of Wales, Cardiff, where he was trained as a clinical geneticist.

At the Institute of Child Health, his research interests span community/preventive pediatrics, and while combining this with his clinical genetics, he has been able to carry out research on infectious and communicable diseases. He has been involved in program planning and monitoring with international organizations including the World Health Organization (WHO), the United Nations International Children's Fund, and the United Nations Development Programme both at the country and international levels.

Through a Fogarty Fellowship (2001–2002) he was able to undertake a course of study leading to the award of a master of arts at Case Western Reserve University, Cleveland, Ohio, during which he was instrumental to the drawing up of the initial works for Nigeria's national ethical guidelines. He was for some time a member of the National Ethics Review Board for Nigeria as well as for the Joint IRC University of Ibadan/University College Hospital. He has more than 80 publications in international and regional journals, and he is a member of many professional associations including the Paediatrics Association of Nigeria, American Society for Bioethics and Humanities, Puebla Group of Networks Collaborating on Health Research for Development, Clinical Genetics Society of Great Britain, and International Association for Tropical Paediatrics. He is the foundation director for the Centre for HIV/AIDS Intervention, Nigeria (CEHAIN). He has also coordinated several studies for the Nigerian Academy of Sciences, and he has been a reviewer for several books and journals, including reviewing some chapters of two books for the Institute of Medicine.

He is also a member of the task force Multilateral Initiative on Malaria (MIM)/Special Programme for Research and Training in Tropical Diseases (TDR)/WHO, the scientific review committee for the European & Developing Countries Clinical Trials Partnership (EDCTP), for the EDCTP Senior Fellowships Training Awards, and the WHO/MIM/TRD Task Force on Malaria Research Capability Strengthening in Africa. Dr. Omotade is also a member of several expert technical groups, including the WHO expert technical group on Intermittent preventive treatment in infants, and the international advisory board of the Institute for Research on Unlimited Love.

He has been on the University College Hospital board of management (2010–2015), and he is a member of the National Child Health Technical Working Group (2015 to date). He has been the chairman of the postgraduate committee at the College of Medicine, University of Ibadan, since 2010.

David H. Peters, M.D., M.P.H., Dr.P.H., is a specialist in international health systems who has worked as a researcher, policy advisor, educator,

Copyright © National Academy of Sciences. All rights reserved.

bureaucrat, manager, and clinician in a number of developing countries over the past 25 years and has been on faculty at Johns Hopkins University since 2001. At Johns Hopkins he oversees a department of more than 150 full-time faculty and about 300 graduate students who are involved in more than 250 projects around the world. He is the research director for the Future Health Systems research consortium, which is working to improve access to and the affordability and quality of health services for the poor, with field sites in five countries in Africa and Asia. He led the development and implementation of the first national Balanced Scorecard to assess and manage health services (in Afghanistan) and conducted research that directly led to the ending of user fees in primary care facilities. He is currently leading a program to strengthen public health systems in Liberia in the wake of the Ebola epidemic. He has written 7 books and more than 100 scientific articles, mostly focusing on health systems in low- and middle-income countries. His teaching and research focus on the performance of health systems; implementation research methods; poverty and health systems; innovations in organization, technology, and financing of health systems; the role of the private sector; human resource management; and ways to use donor assistance to strengthen local capacity in low-income countries.

While at the World Bank as a senior public health specialist, he pioneered the development of sector-wide approaches (SWAps) in health, with the purpose of improving national leadership and coherence over health strategies and improving coordination and accountability of policy implementation. In India he led a research program that included local researchers, government, and civil society in examining health systems and inequities and which was used as a basis for new policies and major programs to improve access and financing for health, notably the Rural Health Mission.

He is the chair of the board of the World Health Organization Alliance for Health Policy and Systems Research, is a member of the scientific advisory board for the President's Emergency Plan for AIDS Relief (PEPFAR), and has served on advisory and technical bodies for the Canadian Institutes of Health Research–Institute of Population and Public Health; Gavi (The Vaccine Alliance); the Global Fund to Fight AIDS, Tuberculosis and Malaria; and the World Economic Forum.

Fred Wabwire-Mangen, M.B.Ch.B., DTM&H, M.P.H., Ph.D., was trained in human medicine at Makerere University, in tropical medicine at Liverpool University, and in immunology and infectious diseases and infectious disease epidemiology at Johns Hopkins University, where he obtained a Ph.D. in 1994. He is an associate professor of epidemiology and public health at the Makerere University School of Public Health, where he teaches infectious disease epidemiology, intervention trials, and health services research. He

Copyright © National Academy of Sciences. All rights reserved.

also has a secondary appointment as a senior research scientist and executive chair at the Makerere University Walter Reed Project (MUWRP). MUWRP is one of the few projects that is conducting Ebola and Marburg vaccine trials in Uganda. Dr Wabwire-Mangen has more than 25 years of conducting research on emerging and reemerging diseases of public health importance in Uganda, including malaria, sexually transmitted infections, HIV/AIDS, influenza, and other emerging viral infections. He also has demonstrated experience leading and managing multidisciplinary research teams. He served as a co-investigator on a cluster randomized trial on sexually transmitted disease control for AIDS prevention and on an individual randomized controlled trial on male circumcision for HIV prevention while working at the Rakai Health Sciences Project between 1994 and 2008, and also served as co-investigator of a Phase 2 and a Phase 2a HIV vaccine trial at MUWRP. As principal investigator of the Surveillance of Influenza Viruses among Human and Non-Human Hosts in Uganda study and the Antimicrobial Resistance Surveillance in Uganda study, funded by Global Emerging Infections Surveillance, Dr. Wabwire-Mangen leads a team of medical doctors, laboratorians, epidemiologists, veterinarians, ornithologists, and other scientists. Dr. Wabwire-Mangen has published widely on public health issues in peer-reviewed journals.

Charles D. Wells, M.D., currently serves as the head of development and the associate vice president for the Infectious Diseases Therapeutic Unit at Sanofi, based in Bridgewater, New Jersey, having joined the organization in September 2015. Prior to joining Sanofi he served as the senior medical director for the Novel Product Opportunities group at Otsuka Pharmaceuticals in Rockville, Maryland. He joined Otsuka in May 2007 to provide the medical and clinical leadership for developing Otsuka's antituberculosis compound, delamanid, which was successfully registered as Deltyba® in 2014 in the European Union, Japan, and Korea for treatment of multidrug resistant (MDR) tuberculosis (TB). In his role at Otsuka he oversaw the clinical development program for delamanid, including clinical operations charged with conducting the global clinical trials in 14 countries across 5 continents, and served on the regulatory submission team responsible for the product's registration. Additionally, he led the publication strategy for reporting results from the clinical development trials for delamanid and led the data submission process to the World Health Organization required for development of interim global guidelines for the use of delamanid in MDR-TB treatment.

Prior to joining Otsuka, he served as the chief of the International Research and Programs Branch of the Division of Tuberculosis Elimination at the U.S. Centers for Disease Control and Prevention (CDC) during 2000–2007. The branch he led at CDC conducted extensive epidemiologic,

Copyright © National Academy of Sciences. All rights reserved.

clinical, and diagnostics research on TB which fed supportive data into evolving global policy and provided direct technical assistance internationally for implementation and scale-up of public health programs for control of TB, HIV-associated TB, and MDR-TB in sub-Saharan Africa, Southeast and South Asia, Eastern Europe, and South America. During this time he also served as CDC's lead representative on the strategic advisory group for the STOP-TB Department at the World Health Organization (WHO) and also the U.S. Agency for International Development (USAID)-supported TB Coalition for Technical Assistance. Additionally, he served as a technical expert on disease control program reviews in numerous countries for the WHO, USAID, and the President's Emergency Plan for AIDS Relief (PEPFAR).

Early in his career he began work in clinical development serving as a research associate at Burroughs Wellcome and Glaxo in Research Triangle Park, North Carolina, in the late 1980s and as an associate medical director at PathoGenesis Corporation in Seattle, Washington, in the late 1990s working on clinical development for anti-infectives, including new drugs for TB.

He is a native of North Carolina and attended North Carolina State University where he received a bachelor of science degree in chemical engineering in 1987. He then completed his medical studies at the University of North Carolina at Chapel Hill in 1992 and his postgraduate medical training in internal medicine and infectious diseases at Emory University and the CDC in Atlanta from 1992 to 1998.

CONSULTANTS

Janet Darbyshire, CBE FMedSci, joined the UK Medical Research Council Tuberculosis and Chest Diseases Unit, after training in respiratory medicine, to coordinate a program of clinical trials and observational epidemiological studies in East Africa and the United Kingdom which led to the short-course chemotherapy regimens which are now the basis of tuberculosis treatment worldwide. She subsequently moved into HIV research at the time when the first antiretroviral drugs were becoming available and led the MRC HIV Clinical Trials Centre, developing a program of clinical trials and observational studies in the United Kingdom and in collaboration with research groups across Europe, Australia, and North and South America and subsequently in Africa.

In 1998 Ms. Darbyshire became the Director of the newly established MRC Clinical Trials Unit (CTU) which incorporated the HIV program and the MRC Cancer Trials Office. The remit of the CTU also extended into other disease areas where there was no strong tradition of clinical trials, such as arthritis and blood transfusion. She retired as director of the CTU in March 2010 but the Unit continues directed by Professor Max Parmar.

Copyright © National Academy of Sciences. All rights reserved.

In 2005 with Professor Peter Selby she became Joint Director of the UK Clinical Research Network (UKCRN) coordinated jointly between the MRC CTU and the University of Leeds. The UKCRN (which became the NIHR CRN) was set up to support both commercial and noncommercial research in the United Kingdom by providing clinical infrastructure in the NHS. The aim was to increase the quality and quantity of clinical research with the overall goal of improving both the health and wealth of the United Kingdom. They retired as Joint Directors in September 2010 and Dr. Jonathan Sheffield has been appointed as Chief Executive.

She has been involved in drug regulation for many years initially on the Committee on Safety of Medicines and then on the Commission on Human Medicines which replaced it. She has served on many research and funding committees and advisory boards and on the World Health Organization and other expert committees as well as numerous trial oversight, data monitoring, and scientific advisory committees. Although she has never lived in Africa she has spent much time there as much of her career has involved collaborative research in resource-poor countries to improve the treatment initially of tuberculosis and subsequently of HIV infection although the two are inextricably linked.

Erin Hammers Forstag, J.D., M.P.H., is a writer, consultant, and attorney in the public health and nonprofit arenas. She received her law degree from Georgetown University Law Center, and her master's of public health from Columbia University. She currently serves as the executive director of Common Good Consulting, which she founded in order to provide small nonprofits with legal, policy, and strategic guidance. She has worked on issues including school food, factory farming, and disaster recovery, and has authored several papers on the intersection between public health and the First Amendment. Erin served as a health volunteer in the Peace Corps in Uzbekistan in 2003.

STUDY STAFF

Michael J. Berrios is a senior program assistant on the Board of Health Sciences Policy of the National Academies of Sciences, Engineering, and Medicine. Mr. Berrios joined the National Academies in 2014 and has worked on the consensus studies *Sharing Clinical Trial Data: Maximizing Benefits, Minimizing Risk* and *Mitochondrial Replacement Techniques: Ethical, Social, and Policy Considerations*. He is currently working on the Forum on Drug Discovery, Development, and Translation. Mr. Berrios received a B.A. in international relations from Michigan State University and is currently a candidate for a master's in Asian studies from the George Washington University.

Copyright © National Academy of Sciences. All rights reserved.

Emily R. Busta, M.S., is an associate program officer on the Board on Health Sciences Policy of the National Academies of Sciences, Engineering, and Medicine. Ms. Busta joined the National Academies staff in October 2014 as staff on the Forum for Drug Discovery, Development, and Translation. Prior to joining the National Academies, she held positions as a research assistant in a placentology lab at the University of Colorado and as a Toxicology Review Fellow at the Center for Food Safety and Applied Nutrition (CFSAN), U.S. Food and Drug Administration. At CFSAN she helped develop and test predictive computational toxicology models and assisted in the safety review of new food contacts. Ms. Busta holds a master's of science degree in biomedical basic sciences from the University of Colorado at Denver–Anschutz Medical Campus and a bachelor of science degree in molecular toxicology from the University of California, Berkeley.

Anne B. Claiborne, J.D., M.P.H., is a senior program officer in the Board on Health Sciences Policy of the National Academies of Sciences, Engineering, and Medicine, where she is staff director of the Forum on Drug Discovery, Development, and Translation and was the responsible staff officer for *Integrating Clinical Research into Epidemic Response: The Ebola Experience*. She has advised or worked on numerous studies and projects relating to drug discovery and development, clinical research, and biomedical ethics, including *Mitochondrial Replacement Techniques: Ethical, Social, and Policy Considerations*; *Global Health Risk Framework: Research and Development of Medical Products: Workshop Summary*; and *Sharing Clinical Trial Data: Maximizing Benefits, Minimizing Risk*. Before joining the National Academies in April 2010, Ms. Claiborne was a practicing health care attorney in the Washington, DC, office of an international law firm. Ms. Claiborne received her bachelor of arts degree, with distinction, from Stanford University; her juris doctorate, cum laude, from Harvard Law School, where she was an editor of the *Harvard Law Review*; and her M.P.H. from the Johns Hopkins Bloomberg School of Public Health, where she was elected to the Delta Omega honorary society. Prior to her graduate studies, Ms. Claiborne spent several years working in public health planning and health services research at the San Francisco Department of Public Health and at the University of California, San Francisco.

Patricia A. Cuff, M.S., M.P.H., is a senior program officer for the Board on Global Health within the Health and Medicine Division of the National Academies of Sciences, Engineering, and Medicine. Her roles involve directing the Global Forum on Innovation in Health Professional Education, and co-directing the study on Clinical Trials During the 2014–2015 Ebola Outbreak. She was the Country Liaison to the Uganda National Academy

Copyright © National Academy of Sciences. All rights reserved.

of Sciences where she worked for 11 years with African academy staff and members in developing their capacity to provide evidence-based science advice to their governments and to their nations. Prior to her role with the African academies, she was the Study Director for the Committee on the Options for Overseas Placement of U.S. Health Professionals and with the Board on Neuroscience and Behavioral Health. Ms. Cuff joined the National Academies staff to work on the report *Emerging Microbial Threats to Health in the 21st Century* under the Board on Global Health. Before coming to Washington, DC, Ms. Cuff worked at St. Luke's-Roosevelt Hospital Center in New York City in the field of HIV nutrition as a counselor, researcher, and lecturer on topics of adult and pediatric HIV. She received an M.S. in nutrition and an M.P.H. in population and family health from Columbia University, and performed her undergraduate studies at the University of Connecticut.

Michelle Mancher, M.P.H., is a program officer on the Board on Health Sciences Policy of the National Academies of Sciences, Engineering, and Medicine. She served as staff co-Director for the *Integrating Clinical Research into Epidemic Response: The Ebola Experience* report and liaison for the Sharing Clinical Trial Data Action Collaborative. Ms. Mancher joined the National Academies in 2009, and has since worked on many consensus studies and workshops related to health care services delivery, clinical trial data sharing, and medical product research and development, including *Initial National Priorities for Comparative Effectiveness Research*; *Clinical Practice Guidelines We Can Trust*; *Variation in Health Care Spending: Target Decision Making Not Geography*; *Sharing Clinical Trial Data: Maximizing Benefits, Minimizing Risk*; and *Global Health Risk Framework: Research and Development of Medical Products: Workshop Summary.* Prior to joining the National Academies, Ms. Mancher held positions at the Arthritis Foundation: Metro DC Chapter, Clinton Foundation's Alliance for a Healthier Generation and the New York City Health and Hospital Corporation's office of managed care. Ms. Mancher holds a master's in public health in health care management and policy from Columbia University, and a bachelor of arts in international relations from the George Washington University.

Julie Pavlin, M.D., Ph.D., M.P.H., is the director, Board on Global Health, Health and Medicine Division, the National Academies of Sciences, Engineering, and Medicine. Prior to joining the National Academies, she was the Research Area Director for Emerging Infectious Diseases and Antimicrobial Resistance and Deputy Research Area Director for HIV at the Infectious Disease Clinical Research Program, part of the Uniformed Services University, and before that the Deputy Director of the Armed Forces

Copyright © National Academy of Sciences. All rights reserved.

Health Surveillance Center. She is a retired Colonel in the U.S. Army and previous assignments included the Chief of the Global Emerging Infections Department at the Armed Forces Research Institute of Medical Sciences in Bangkok, Thailand, where she developed surveillance programs for infectious diseases in Asia, the Chief of the Field Studies Department at the Walter Reed Army Institute of Research where she played a pivotal role in developing the Electronic Surveillance System for the Early Notification of Community-based Epidemics (ESSENCE), the U.S. Department of Defense real-time surveillance system, and Assistant Chief of the Operational Medicine Division at the U.S. Army Medical Research Institute for Infectious Diseases. Dr. Pavlin received her A.B. from Cornell University, her M.D. from Loyola University, her M.P.H. from Harvard University, and her Ph.D. in emerging infectious diseases at the Uniformed Services University.

Andrew M. Pope, Ph.D., is director of the Board on Health Sciences Policy of the National Academies of Sciences, Engineering, and Medicine. He has a Ph.D. in physiology and biochemistry from the University of Maryland and has been a member of the National Academies staff since 1982 and of the Health and Medicine Division staff since 1989. His primary interests are science policy, biomedical ethics, and environmental and occupational influences on human health. During his tenure at the National Academies, Dr. Pope has directed numerous studies on topics that range from injury control, disability prevention, and biologic markers to the protection of human subjects of research, National Institutes of Health priority-setting processes, organ procurement and transplantation policy, and the role of science and technology in countering terrorism. Since 1998, Dr. Pope has served as Director of the Board on Health Sciences Policy, which oversees and guides a program of activities that is intended to encourage and sustain the continuous vigor of the basic biomedical and clinical research enterprises needed to ensure and improve the health and resilience of the public. Ongoing activities include Forums on Neuroscience, Genomics, Drug Discovery and Development, and Medical and Public Health Preparedness for Disasters and Emergencies. Dr. Pope is the recipient of the Health and Medicine Division's Cecil Award and the National Academy of Sciences' President's Special Achievement Award.

Olivia C. Yost, M.S., is a research associate on the Board on Health Sciences Policy of the National Academies of Sciences, Engineering, and Medicine. She provided research support to the consensus report *Integrating Clinical Research into Epidemic Response: The Ebola Experience*, as well as the Committee on Preventing Dementia and Cognitive Impairment and the Committee on Issues in Organ Donor Intervention Research. Prior to joining the National Academies in 2015, Ms. Yost worked as a research officer

Copyright © National Academy of Sciences. All rights reserved.

for ARCHIVE Global, a global health organization based in New York City, where she oversaw the implementation of monitoring and evaluation programs and field studies focused on the deployment of environmental strategies for malaria, tuberculosis, and gastrointestinal infection control in Haiti, Cameroon, and Bangladesh. Ms. Yost received her M.S. in the control of infectious diseases from the London School of Hygiene & Tropical Medicine in 2012. Her graduate research focused on developing alternative methodologies for assessing small-scale wastewater infrastructure decay in rural Alabama. She received her B.A. in history and communications from Franklin University in Switzerland in 2011.

Copyright © National Academy of Sciences. All rights reserved.
